# Synthesis of a Series of Diaminoindoles

**DOI:** 10.1021/acs.joc.1c00652

**Published:** 2021-08-05

**Authors:** James S. Martin, Claire J. Mackenzie, Ian H. Gilbert

**Affiliations:** Wellcome Centre for Anti-Infectives Research, Division of Biological Chemistry and Drug Discovery, University of Dundee, Dundee DD1 5EH, U.K.

## Abstract

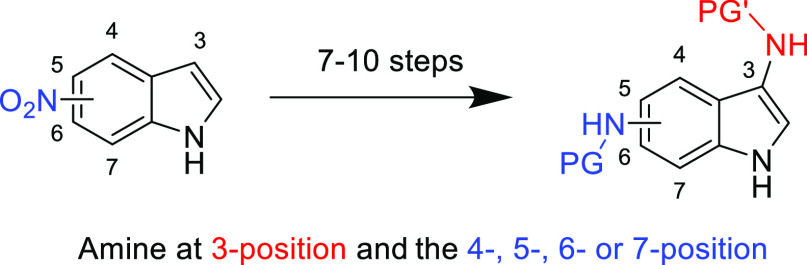

A selection of 3,4-diaminoindoles were required for a recent drug discovery project. To this end, a 10-step synthesis was developed from 4-nitroindole. This synthesis was subsequently adapted and used to synthesize 3,5-; 3,6-; and 3,7-diaminoindoles from the corresponding 5-, 6-, or 7-nitroindole. These novel intermediates feature orthogonal protecting groups that allow them to be further diversified. This is the first reported synthesis of these types of compounds.

## Introduction

Indoles are an important feature of many drugs.^1^ A recent search through DrugBank revealed 100 compounds in which an indole was a component. A search of ChEMBL indicated that there were 1688 structures with a 3-aminoindole embedded in the structure. Further, indoles are found in many natural products^2^ and dyes.^3^

**Figure 1:**
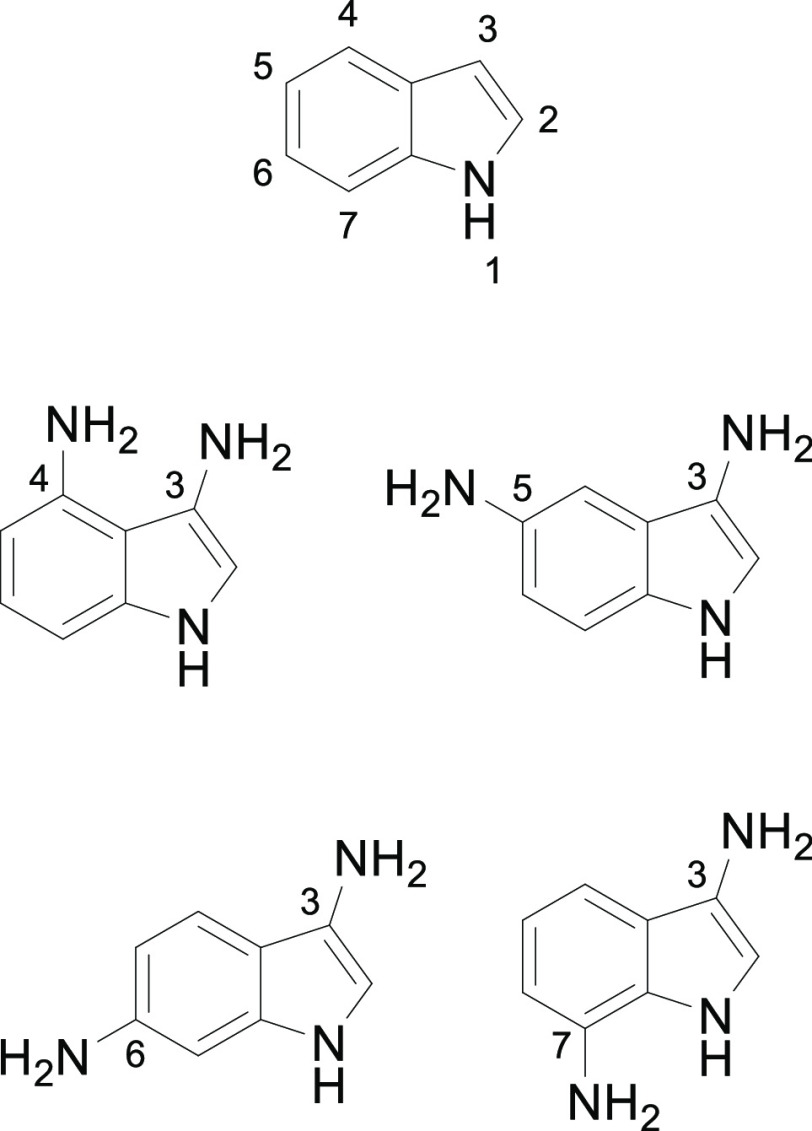
Top—Indole numbering scheme. Middle—3,4-Diaminoindole and 3,5-diaminoindole. Bottom—3,6-Diaminoindole and 3,7-diaminoindole.

During a recent drug discovery campaign, we required a selection of 3,4-diaminoindoles. It was important that each amino group could be differentially derivatized in order to carry out a hit expansion. However, literature searches revealed that the only published examples of diaminoindoles are 2,3-diaminoindole^4^ and 6,7-diaminoindole.^5^ Some dinitroindoles are known;^6^ however, conversion of these to diaminoindoles has not been reported. A 2-substituted 3-amino-4-nitroindole is also known.^7^ It is unusual that such simple cores are unknown in the literature. Our initial focus was on the synthesis of 3,4-diaminoindoles; however, this synthesis turned out to be problematic. In this article, we describe our synthesis of the differentially protected 3,4-diaminoindole, which was suitable for further modification. Once this was successfully completed, the 3,5-, 3,6-, and 3,7-analogues were also prepared, with all shown in Figure 1.

## Results and Discussion

**Scheme 1:**
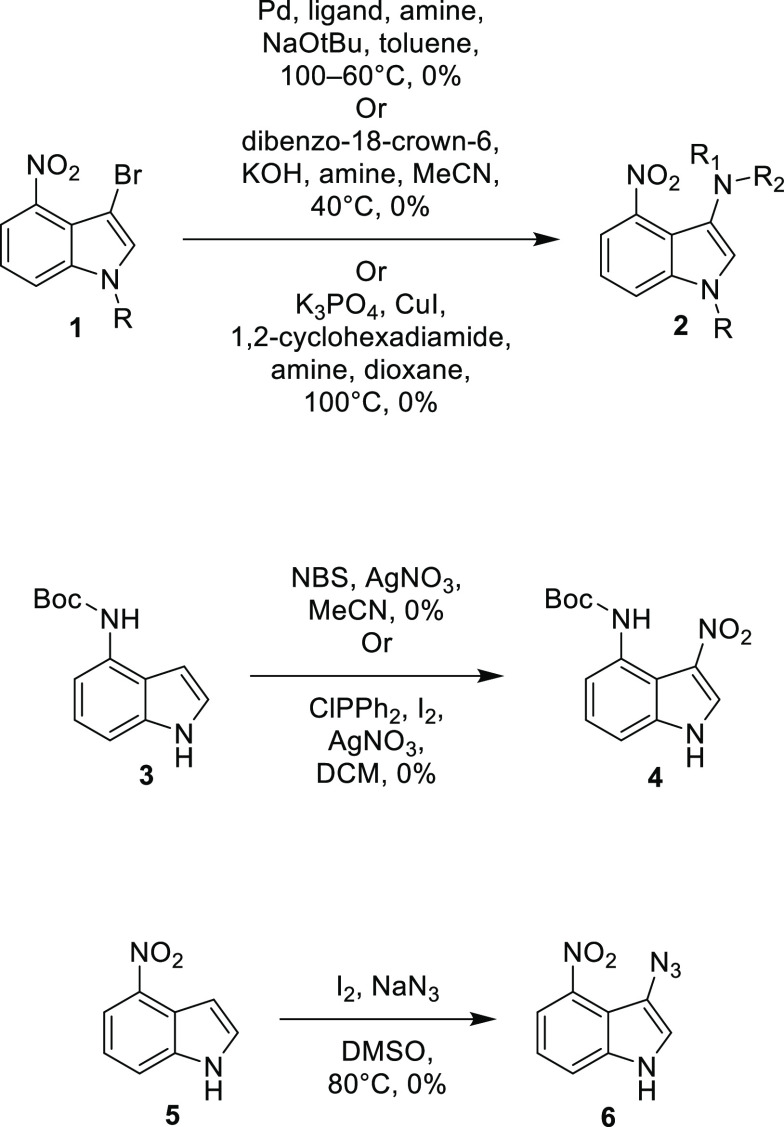
Attempted Synthesis of 3-Amino-4-nitroindoles **2**,4-(Boc)amino-3-nitroindole **4**, and 3-Azido-4-nitroindole **6**

To illustrate the challenges of preparation of the diaminoindoles, we report some of our initial approaches to access this system. It is known that the most reactive position on an indole ring is the 3-position, so we focused on introducing the required amine here. The synthesis was started with the nitrogen on the phenyl ring masked as a nitro group or as the Boc-protected or Cbz-protected amine. However, our attempts to functionalize the 3-position via typical means such as Buchwald–Hartwig couplings, nitrations, and azidations proved unsuccessful (Scheme 1).

We had previously found that a Curtius rearrangement could be used to convert indole-3-carboxylic acids into the corresponding indole-3-isocyanates, which could subsequently be converted to 3-aminoindoles and related derivatives. However, the required 4-aminoindole-3-carboxylic acid intermediate is not commercially available or synthetically known. Typically, indole-3-carboxylic acids can be synthesized by reacting indoles with trifluoroacetic anhydride (TFAA) to give the corresponding trifluoroketone, which subsequently undergoes base hydrolysis in a pseudo-haloform reaction to give the carboxylic acid,^8^ or alternatively by oxidation of the corresponding aldehyde.^9^

However, we found that in the case of 4-nitroindole or protected 4-aminoindoles, these routes were unsuccessful.

**Scheme 2:**
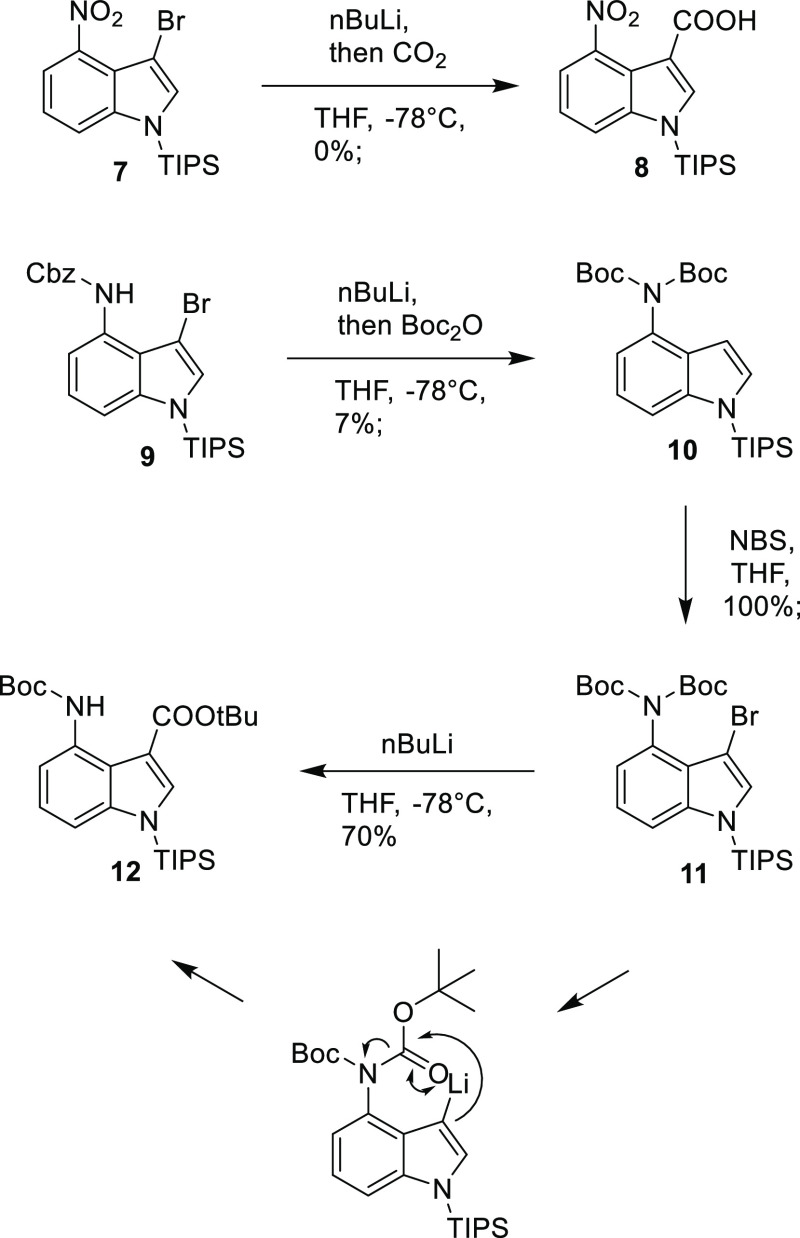
Top—Attempted Synthesis of 4-Nitro-1-TIPS-indole-3-carboxylic Acid **8**; Bottom—Synthesis of *tert*-Butyl 4-((*tert*-Butoxycarbonyl)amino)-1-(triisopropylsilyl)-1*H*-indole-3-carboxylate **12**

Instead, it was proposed that brominating the 3-position of 4-nitroindole to give **7** would allow a lithium halogen exchange to occur by treating with *n*-butyl lithium, and the resulting anion could be quenched with CO_2_ to give the required carboxylic acid **8** as shown in Scheme 2. However, no lithium halogen exchange was observed in this case, likely due to the electron withdrawing effect of the nitro group.

An analogous reaction was attempted where the nitro group was substituted with the Cbz-protected amine **9** and the CO_2_ substituted with Boc anhydride (Scheme 2). This reaction was unsuccessful, and instead, the di-Boc-protected amine **10** was found to be the main product. It was observed that by brominating this product and then treating it with *n*-butyl lithium, a novel Boc migration occurred, yielding ester **12.** This presumably occurred via lithiation, followed by transfer of the Boc group to the 3-position of the indole as shown in Scheme 2. This allowed access to the carboxylic acid and subsequently the synthesis of the desired 3,4-diaminoindoles.

**Scheme 3:**
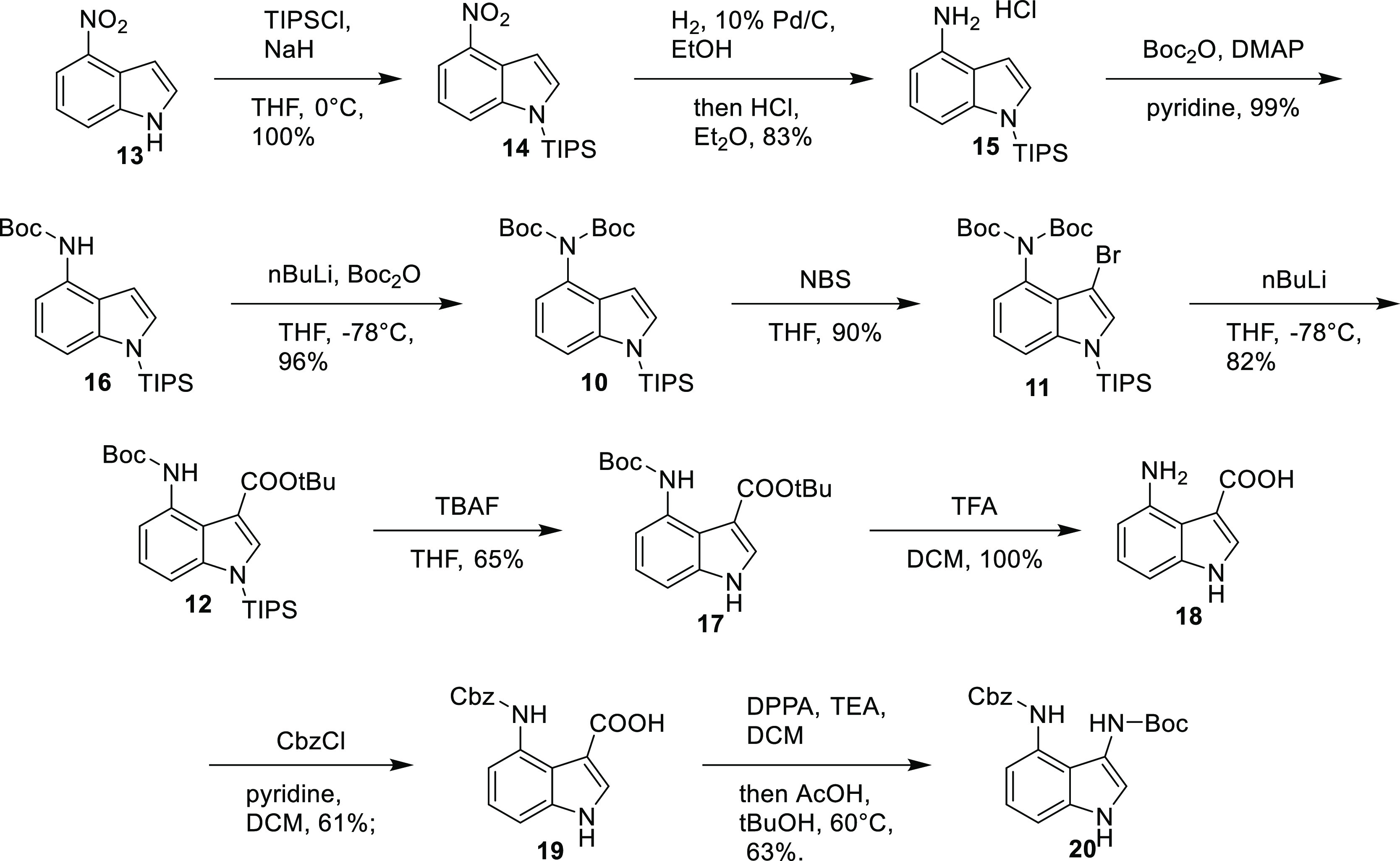
Synthesis of Benzyl *tert*-Butyl (1*H*-Indole-3,4-diyl)dicarbamate **20**

This key step allowed us to prepare the diprotected 3,4-diaminoindole (Scheme 3). A number of different strategies, protecting groups, and orders of reactions were investigated. Eventually, the following route was found to be successful. 4-Nitroindole **13** was protected with a triisopropylsilyl (TIPS)-protecting group **14**, and the nitro group reduced to amine **15** using hydrogen and palladium on carbon. The di-Boc-protected compound **10** was formed in two steps, initially via treatment with Boc anhydride and 4-dimethylaminopyridine (DMAP) to give the mono-protected compound **16** and then with more forcing conditions using *n*-butyl lithium and Boc anhydride. Bromination with *N*-bromosuccinimide (NBS), followed by rearrangement using *n*-butyl lithium, gave the required ester **12**. Deprotection of the indole NH was achieved using tetra-n-butylammonium fluoride (TBAF) to give indole **17**. The remaining Boc group and *tert*-butyl ester were cleaved using TFA, and the free amine **18** reprotected with Cbz to give carboxylic acid **19**. Treating this acid with diphenylphosphoryl azide (DPPA) in DCM gave the acyl azide which was then heated in *tert*-butanol to induce the rearrangement to give the isocyanate and subsequently the required bisprotected 3,4-diaminoindole **20**. The overall yield of this synthesis was 15% over 10 steps.

It was noted that no other examples of 3,*x*-diaminoindoles were identified in the literature, so the synthesis of 3,4-diaminoindole was modified to allow access to these compounds. It was found that the first step, forming the protected indole, was extremely challenging with 7-nitroindole and ultimately the product was unstable.

**Scheme 4:**
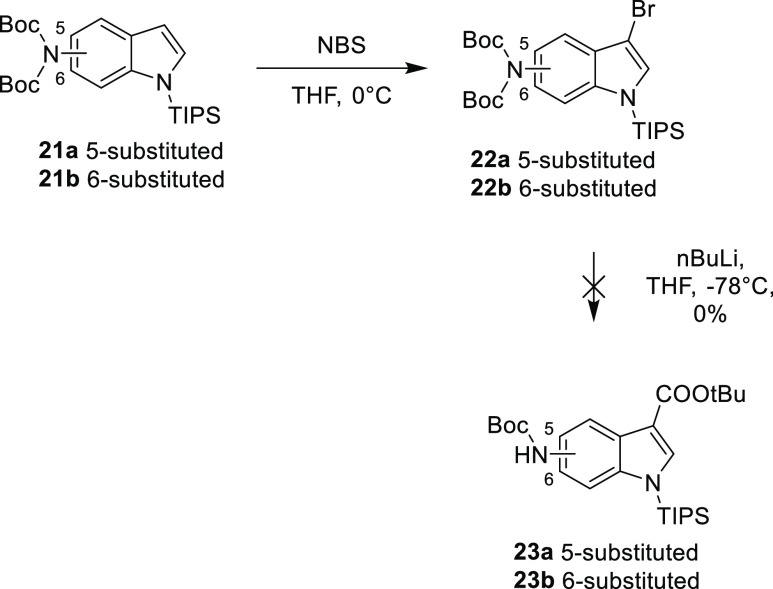
Attempted Synthesis of *tert*-Butyl 5-(Boc)aminoindole-3-carboxylate **23a** and *tert*-Butyl 6-(Boc)aminoindole-3-carboxylate **23b**

It was subsequently found that on attempting to lithiate the 3-bromo derivatives of 5- and 6-aminoindole **22a** and **22b** later in this synthesis, no transfer of a carboxylate occurred. This is probably due to the lack of proximity of the diprotected amino group to the lithiated center (Scheme 4).

**Scheme 5:**
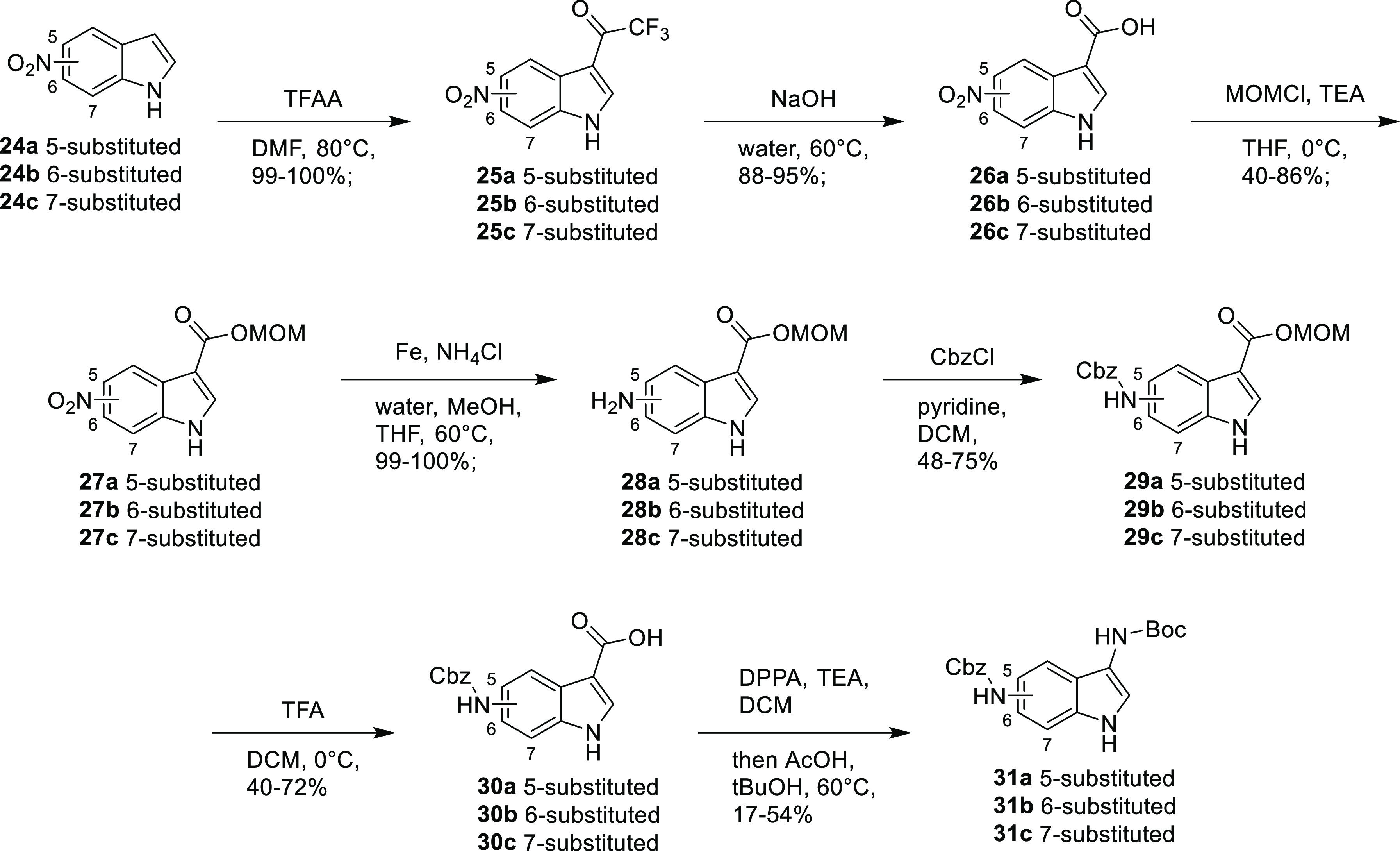
Synthesis of Benzyl *tert*-Butyl (1*H*-Indole-3,5-diyl)dicarbamate **31a**, Benzyl *tert*-Butyl (1*H*-Indole-3,6-diyl)dicarbamate **31b**, and Benzyl *tert*-Butyl (1*H*-Indole-3,7-diyl)dicarbamate **31c**

The pseudo-haloform reaction had previously been shown not to work when attempting the synthesis of 3,4-diaminoindole but was attempted again here as shown in Scheme 5 as the reactivity of these compounds appeared significantly different from that seen when synthesizing 3,4-diaminoindole. Reacting the appropriate nitroindoles **24a–c** with TFAA gave trifluoroketones **25a–c** which were readily hydrolyzed to carboxylic acids **26a–c** using sodium hydroxide. Attempting to reduce the nitro groups to amines using hydrogen and palladium resulted in a lot of degradation to give a complex mixture, so alternatives were investigated. A transfer hydrogenation using ammonium chloride and iron gave the expected amino acid cleanly.

However, residual ammonium chloride could not be completely separated from the product and inhibited the next step. To allow separation of the product and ammonium chloride, various carboxylic acid-protecting groups were investigated. In the case of a methyl group or a trimethylsilylethoxymethyl (SEM) group, the required chemistry was found to be successful; however, the protecting groups could not be cleaved. Ultimately, the methoxymethyl (MOM)-protecting group was found to facilitate the required chemistry and could be cleaved when required using TFA. Therefore, the carboxylic acids were protected as MOM esters **27a–c**. The nitro groups were then reduced with iron and ammonium chloride to give amines **28a–c**. The resulting amino compounds could be protected with a Cbz group to give the protected amino acids **29a–c**. Removal of the MOM-protecting groups with TFA gave carboxylic acids **30a–c**; the Curtius rearrangement could then be carried out. The carboxylic acid was converted to the acyl azide using DPPA; then, by heating in *tert*-butanol, the desired bisprotected diaminoindoles **31a–c** were isolated.

The final 3,4-, 3,5-, 3,6-, and 3,7-diaminoindoles synthesized here feature an orthogonal protecting group on each amine, allowing them to be selectively functionalized at each position to give compounds of interest to ongoing drug discovery projects.

## Conclusions

Here, we have developed the first reported synthesis of 3,4-diaminoindole, a novel core of interest in a drug discovery campaign. This synthesis was further developed to produce the novel 3,5-, 3,6-, and 3,7-diaminoindoles. These compounds are protected with orthogonal protecting groups to allow required chemistry to be undertaken on these attractive intermediates. This represents the first known route to this class of compounds.

## Experimental Section

### General Methods

Chemicals and solvents were purchased from commercial sources and were used without any further purification unless noted otherwise. Air- and water-sensitive reactions were carried out under an inert nitrogen atmosphere in oven-dried glassware. Reactions which required heating were heated in DrySyn heating blocks. Analytical thin-layer chromatography (TLC) was performed on precoated TLC plates (layer 0.20 mm silica gel 60 with fluorescent indicator UV254, from Merck). Developed plates were air-dried and analyzed under a UV lamp (UV254/365 nm) and by staining with permanganate or ninhydrin. Flash column chromatography was performed on prepacked silica gel cartridges (230–400 mesh, 35–70 μm, from RediSep) using a Teledyne ISCO Combiflash Rf or Combiflash Rf 200i. ^1^H (500 MHz), ^13^C (126 MHz), and 2D NMR spectra were recorded in dimethyl sulfoxide (DMSO)-*d*_6_ using a Bruker AVANCE spectrometer. Proton chemical shifts are reported in ppm relative to the residual DMSO peak (δ = 2.50 ppm). Multiplicities are given as s (singlet), d (doublet), t (triplet), q (quartet), hept (heptet), m (multiplet), brs (broad singlet), dd (doublet of doublets), or a combination of these. Coupling constants (*J*) are quoted to the nearest 0.1 Hz. ^13^C chemical shifts are reported in ppm relative to the residual DMSO peak (δ = 39.5 ppm). Assignment of proton and carbon signals was achieved using correlation spectroscopy, heteronuclear single-quantum coherence, and heteronuclear multiple bond correlation experiments, which are reported using the indole number scheme in Figure 1. High-resolution electrospray measurements were performed on a Bruker Daltonics MicrOTOF mass spectrometer.

#### *tert*-Butyl (*tert*-Butoxycarbonyl)(1-(triisopropylsilyl)-1*H*-indol-4-yl)carbamate **10**

*tert*-Butyl (1-(Triisopropylsilyl)-1*H*-indol-4-yl)carbamate **16** (4.38 g, 10.7 mmol, 1 equiv) was dissolved in THF (50 mL) under nitrogen and cooled to −78 °C. *n*-Butyl lithium (2.5 M in hexane) (6.42 mL, 16.1 mmol, 1.5 equiv) was added dropwise, and the reaction mixture was stirred at −78 °C for 30 min. Di*tert*-butyl dicarbonate (4.92 mL, 21.4 mmol, 2 equiv) was added, and the reaction mixture was allowed to slowly warm up to room temperature. After 3 h, the reaction mixture was quenched with 20 mL of saturated ammonium chloride solution, diluted with 20 mL of water, and 50 mL of DCM. The layers were separated, and the aqueous layer was extracted 2x with 50 mL of DCM. The combined organics were washed with brine, dried over MgSO_4_, passed through a phase separator, and evaporated to dryness. The residue was purified by flash chromatography (0–30% ethyl acetate in heptane) to give *tert*-butyl (*tert*-butoxycarbonyl)(1-(triisopropylsilyl)-1*H*-indol-4-yl)carbamate **10** (5.26 g, 96%) as a colorless oil which crystallized on standing. ^1^H (500 MHz, DMSO-*d*_6_): δ 7.49 (1H, d, *J* = 8.3 Hz, H7), 7.39 (1H, d, *J* = 3.1 Hz, H2), 7.11 (1H, t, *J* = 7.9 Hz, H6), 6.86 (1H, d, *J* = 7.5 Hz, H5), 6.38 (1H, d, *J* = 3.1 Hz, H3), 1.75 (3H, hept, *J* = 7.4 Hz, Si(CH)_3_), 1.35 (18H, s, Boc CH_3_), 1.09 (18H, d, *J* = 7.5 Hz, TIPS CH_3_); ^13^C{^1^H} (126 MHz, DMSO-*d*_6_): δ 151.6 (C=O), 141.3 (C7a), 132.0 (C2), 130.9 (C4), 128.8 (C3a), 121.2 (C6), 118.8 (C5), 112.9 (C7), 101.6 (C3), 81.7 (**C**(CH_3_)_3_), 27.4 (Boc CH_3_), 17.8 (TIPS CH_3_), 11.9 (Si**C**H(CH_3_)_2_); HRMS (ESI/TOF) *m*/*z*: [M + H]^+^ calcd for C_27_H_45_N_2_O_4_Si, 489.3143; found, 489.3161.

#### *tert*-Butyl (3-Bromo-1-(triisopropylsilyl)-1*H*-indol-4-yl)carbamate **11**

*tert*-Butyl (*tert*-butoxycarbonyl)(1-(triisopropylsilyl)-1*H*-indol-4-yl)carbamate **10** (8.59 g, 17.6 mmol, 1 equiv) was dissolved in THF (75 mL), and freshly recrystallized NBS (3.13 g, 17.6 mmol, 1 equiv) was added. The reaction mixture was stirred at room temperature for 1 h. The reaction mixture was diluted with 75 mL of water and extracted 3x with 75 mL of diethyl ether. The combined organics were washed with 75 mL of saturated NaHCO_3_ solution and 75 mL of brine, dried over MgSO_4_, passed through a phase separator, and evaporated to dryness to give *tert*-butyl (3-bromo-1-(triisopropylsilyl)-1*H*-indol-4-yl)carbamate **11** (9.00 g, 90%) as a brown powder. ^1^H (500 MHz, DMSO-*d*_6_): δ 7.56 (1H, d, *J* = 8.5 Hz, H7), 7.44(1H, s, H2), 7.18 (1H, t, *J* = 8.0 Hz, H6), 6.90 (1H, d, *J* = 7.5 Hz, H5), 1.76 (3H, hept, *J* = 7.4 Hz, Si(CH)_3_), 1.32 (18H, s, Boc CH_3_), 1.08 (18H, d, *J* = 7.5, TIPS CH_3_); ^13^C{^1^H} (126 MHz, DMSO-*d*_6_): δ 151.0 (C=O), 140.9 (C7a), 131.3 (C2), 131.0 (C4), 125.1 (C3a), 122.3 (C6), 120.8 (C5), 114.0 (C7), 89.6 (C3), 81.4 (**C**(CH_3_)_3_), 27.5 (Boc CH_3_), 17.7 (TIPS CH_3_), 11.8 (Si**C**H(CH_3_)_2_); HRMS (ESI/TOF) *m*/*z*: [M + H]^+^ calcd for C_27_H_44_N_2_O_4_SiBr, 567.2248; found, 567.2250.

#### *tert*-Butyl 4-((*tert*-Butoxycarbonyl)amino)-1-(triisopropylsilyl)-1*H*-indole-3-carboxylate **12**

*tert*-Butyl (3-Bromo-1-(triisopropylsilyl)-1*H*-indol-4-yl)carbamate **11** was dissolved in THF (75 mL) under nitrogen and cooled to −78 °C. *n*-Butyl lithium (2.5 M in hexane) (8.44 mL, 21.1 mmol, 1.2 equiv) was added portionwise over 30 min, and the reaction mixture was stirred at −78 °C for a further 30 min. The reaction mixture was quenched with 50 mL of saturated ammonium chloride solution, diluted with 50 mL of water, and extracted 3x with 50 mL of diethyl ether. The combined organic layers were washed with brine, dried over MgSO_4_, passed through a phase separator, and evaporated to dryness. The residue was purified by flash chromatography (0–20% ethyl acetate in heptane) to give *tert*-butyl 4-((*tert*-butoxycarbonyl)amino)-1-(triisopropylsilyl)-1*H*-indole-3-carboxylate **12** (7.08 g, 82%) yield as a white powder. ^1^H (500 MHz, DMSO-*d*_6_): δ 10.91 (1H, br s, NH), 7.91 (1H, d, *J* = 7.9 Hz, H5), 7.89 (1H, s, H2), 7.23 (1H, d, *J* = 8.3 Hz, H7), 7.17 (1H, t, *J* = 8.1 Hz, H6), 1.74 (3H, hept, *J* = 7.3 Hz, Si(CH)_3_), 1.57 (9H, s, CH3), 1.49 (9H, s, CH_3_), 1.09 (18H, d, *J* = 7.5, TIPS CH_3_); ^13^C{^1^H} (126 MHz, DMSO-*d*_6_): δ 165.9 (C=O ester), 152.6 (C=O carbamate), 142.1 (C7a), 139.3 (C2), 132.7 (C4) 123.8 (C6), 117.4 (C3a), 110.5 (C3), 109.9 (C5), 108.5 (C7), 81.0 (**C**(CH_3_)_3_), 78.9 (**C**(CH_3_)_3_), 28.0 (CH_3_), 27.9 (CH_3_), 17.7 (TIPS CH_3_), 11.8 (Si**C**H(CH_3_)_2_); HRMS (ESI/TOF) *m*/*z*: [M + H]^+^ calcd for C_27_H_45_N_2_O_4_Si, 489.3143; found, 489.3154.

#### 4-Nitro-1-(triisopropylsilyl)-1*H*-indole **14**

4-Nitroindole **13** (2.00 g, 12.3 mmol, 1 equiv) was dissolved in dry THF (20 mL) and cooled to 0 °C under nitrogen. Sodium hydride (60% in mineral oil) (592 mg, 14.8 mmol, 1.2 equiv) was added, and the reaction mixture was stirred at 0 °C for 30 min. Triisopropylsilyl chloride (3.4 mL, 16.0 mmol, 1.3 equiv) was added dropwise, and the reaction mixtures were stirred at 0 °C for 30 min. The reaction mixture was quenched with 20 mL of saturated ammonium chloride and extracted 3x with 20 mL of DCM. The combined organics were washed with brine, dried over MgSO_4_, passed through a phase separator, and evaporated to dryness. The residue was purified by flash chromatography (0–30% ethyl acetate in heptane) to give 4-nitro-1-(triisopropylsilyl)-1*H*-indole **14** (4.34 g, 100%) as a yellow powder. ^1^H (500 MHz, DMSO-*d*_6_): δ 8.10 (1H, d, *J* = 8.0 Hz, H5), 8.04 (1H, d, *J* = 8.2 Hz, H7), 7.78 (1H, d, *J* = 3.1 Hz, H2), 7.36 (1H, t, *J* = 8.1 Hz, H6), 7.26 (1H, d, *J* = 3.0 Hz, H3), 1.81 (3H, hept, *J* = 7.5 Hz, Si(CH)_3_), 1.09 (18H, d, *J* = 7.5, CH3); ^13^C{^1^H} (126 MHz, DMSO-*d*_6_): δ 142.5 (C7a), 139.5 (C4), 137.0 (C2), 124.8 (C3a), 121.0 (C7), 120.8 (C6), 117.4 (C5), 104.4 (C3), 17.7 (CH3), 11.8 (Si**C**H(CH_3_)_2_); HRMS (ESI/TOF) *m*/*z*: [M + H]^+^ calcd for C_17_H_27_N_2_O_2_Si, 319.1836; found, 319.1836.

#### 1-(Triisopropylsilyl)-1*H*-indol-4-amine Hydrochloride **15**

4-Nitro-1-TIPS-indole **14** (4.34 g, 12.3 mmol, 1 equiv) was suspended in ethanol (50 mL) with 10% Pd/C (69 mg, 0.06 mmol, 0.5 mol %) under a nitrogen atmosphere. The reaction mixture was purged 3x with hydrogen and then stirred under a hydrogen atmosphere for 4 days. The reaction mixture was purged 3x with nitrogen, filtered through Celite, and evaporated to dryness. The residue was dissolved in 20 mL of diethyl ether, and 6 N HCl was added dropwise until the precipitate stopped forming. The precipitate was isolated by vacuum filtration and washed 3x with diethyl ether and then dried under vacuum to give 1-(triisopropylsilyl)-1*H*-indol-4-amine hydrochloride **15** (3.52 g, 83%) as a white powder. ^1^H (500 MHz, DMSO-*d*_6_): δ 10.09 (3H, br s, NH_3_), 7.53 (1H, d, *J* = 7.8 Hz, H7), 7.48 (1H, d, *J* = 2.3 Hz, H2), 7.17 (1H, t, *J* = 7.8 Hz, H6), 7.06 (1H, br s, H5), 6.84 (1H, br s, H3), 1.76 (3H, hept, *J* = 7.5 Hz, Si(CH)_3_), 1.08 (18H, d, *J* = 7.5 Hz, TIPS CH_3_); ^13^C{^1^H} (126 MHz, DMSO-*d*_6_): δ 141.3 (C7a), 132.4 (C2), 121.6 (C6), 113.4 (C5), 113.3 (C7), 101.9 (C3), 17.7 (CH_3_), 11.8 (Si**C**H(CH_3_)_2_); HRMS (ESI/TOF) *m*/*z*: [M + H]^+^ calcd for C_17_H_29_N_2_Si, 289.2095; found, 289.2105.

#### *tert*-Butyl (1-(Triisopropylsilyl)-1*H*-indol-4-yl)carbamate **16**

1-(Triisopropylsilyl)-1*H*-indol-4-amine hydrochloride **15** (3.52 g, 10.8 mmol, 1 equiv) was dissolved in pyridine (20 mL) with DMAP (132 mg, 1.1 mmol, 10 mol %) and stirred under nitrogen. Di*tert*-butyl dicarbonate (2.59 g, 11.9 mmol, 1.1 equiv) was added, and a rapid evolution of gas was observed after about 30 s. The reaction mixture was stirred at room temperature overnight. The reaction mixture was evaporated to dryness, and the residue was dissolved in 20 mL of DCM and washed with 20 mL of 0.5 N HCl. The aqueous layer was extracted 2x with 10 mL of DCM, washed with saturated NaHCO_3_ solution and brine, dried over MgSO_4_, passed through a phase separator, and evaporated to dryness. The residue was purified by flash chromatography (0–20% ethyl acetate in heptane) to give *tert*-butyl (1-(triisopropylsilyl)-1*H*-indol-4-yl)carbamate **16** (4.38 g, 99%) as a colorless oil which crystallized on standing. ^1^H (500 MHz, DMSO-*d*_6_): δ 8.96 (1H, br s, NH), 7.35 (1H, d, *J* = 7.7 Hz, H5), 7.27 (1H, d, *J* = 3.0 Hz, H2), 7.21 (1H, d, *J* = 8.3 Hz, H7), 7.01 (1H, t, *J* = 8.0 Hz, H6), 6.90 (1H, d, *J* = 3.0 Hz, H3), 1.72 (3H, hept, *J* = 7.5 Hz, Si(CH)_3_), 1.50 (9H, s, Boc CH_3_), 1.08 (18H, d, *J* = 7.5 Hz, TIPS CH_3_); ^13^C{^1^H} (126 MHz, DMSO-*d*_6_): δ 153.25 (C=O), 141.0 (C7a), 131.0 (C4), 130.0 (C2), 121.4 (C6), 110.7 (C5), 108.8 (C7), 102.9 (C3), 78.7 (Boc **C**(CH_3_)_3_), 28.1 (Boc CH_3_), 17.8 (TIPS CH_**3**_), 11.9 (Si**C**H(CH_3_)_2_); HRMS (ESI/TOF) *m*/*z*: [M + H]^+^ calcd for C_22_H_37_N_2_O_2_Si, 389.2614; found, 389.2619.

#### *tert*-Butyl 4-((*tert*-Butoxycarbonyl)amino)-1*H*-indole-3-carboxylate **17**

*tert*-Butyl 4-((*tert*-Butoxycarbonyl)amino)-1-(triisopropylsilyl)-1*H*-indole-3-carboxylate **12** (7.08 g, 10.9 mmol, 1 equiv) was dissolved in THF (50 mL) under nitrogen, and TBAF (16.3 mL, 16.3 mmol, 1.5 equiv) was added dropwise. After 45 min, 50 mL of water was added, and the mixture extracted 3x with 50 mL of DCM. The combined organics were dried over MgSO_4_, passed through a phase separator, and evaporated to dryness. The residue was purified by flash chromatography (0–30% ethyl acetate in heptane) to give *tert*-butyl 4-((*tert*-butoxycarbonyl)amino)-1*H*-indole-3-carboxylate **17** (2.49 g, 65%) yield as a white powder. ^1^H (500 MHz, DMSO-*d*_6_): δ 11.99 (1H, br s, NH), 11.01 (1H, br s, NH), 7.99 (1H, s, H2), 7.88 (1H, d, *J* = 7.2 Hz, H5), 7.13 (2H, m, H6, H7), 1.57 (9H, s, CH_3_), 1.49 (9H, s, CH_3_); ^13^C{^1^H} (126 MHz, DMSO-*d*_6_): δ 166.5 (C=O), 152.5 (C=O), 137.9 (C7a), 133.7 (C2), 132.6 (C4), 123.4 (C6), 115.1 (C3a), 108.8 (C5), 107.3 (C3), 106.5 (C7), 80.5 (**C**(CH)_3_), 78.8 (**C**(CH)_3_), 28.0 (Boc CH_3_, *t*-Butyl CH_3_); HRMS (ESI/TOF) *m*/*z*: [M + H]^+^ calcd for C_18_H_25_N_2_O_4_, 333.1809; found, 333.1820.

#### 4-(((Benzyloxy)carbonyl)amino)-1*H*-indole-3-carboxylic Acid **19**

*tert*-Butyl 4-((*tert*-Butoxycarbonyl)amino)-1*H*-indole-3-carboxylate **17** (2.19 g, 6.57 mmol, 1 equiv) was dissolved in DCM (44 mL) under nitrogen. TFA (44 mL, 65.7 mmol, 10 equiv) was added, and the reaction mixture was stirred for 1 h. The reaction mixture was evaporated to dryness to give the intermediate as an off-white powder. The residue was dissolved in DCM (45 mL) under nitrogen, and pyridine (2.66 mL, 32.8 mmol, 5 equiv) was added. Benzyl chloroformate (1.84 mL, 13.1 mmol, 2 equiv) was added dropwise. The reaction mixture was stirred at room temperature overnight. The reaction mixture was evaporated to dryness to remove the residual pyridine, and the residue was dissolved in 50 mL of water and acidified to pH 1 with concentrated HCl. The resulting precipitate was isolated by vacuum filtration and then purified by reverse phase flash chromatography (C_18_ column) (5–95% acetonitrile in water (0.1% formic acid)) to give 4-(((benzyloxy)carbonyl)amino)-1*H*-indole-3-carboxylic acid **19** (1.31 g, 61%) as a white powder. ^1^H (500 MHz, DMSO-*d*_6_): δ 12.60 (1H, br s, COOH), 12.00 (1H, br s, H1), 11.85 (1H, br s, NH), 8.08 (1H, d, *J* = 2.8 Hz, H2), 7.91 (1H, d, *J* = 7.4 Hz, H5), 7.46–7.30 (5H, m, Cbz-CH), 7.2–7.1 (2H, m, H6, H7), 5.19 (2H, s, CH_2_); ^13^C{^1^H} (126 MHz, DMSO-*d*_6_): δ 168.7 (COOH), 153.0 (carbamate C=O), 138.0 (C7a), 136.8 (Cbz-C), 133.9 (C2), 132.2 (C4) 128.4 (Cbz-CH), 127.8 (Cbz-CH), 127.5 (Cbz-CH), 123.5 (C6), 115.4 (C3a), 108.6 (C5), 106.8 (C7), 106.4 (C3), 65.4 (CH_2_); HRMS (ESI/TOF) *m*/*z*: [M + H]^+^ calcd for C_17_H_15_N_2_O_4_, 311.1026; found, 311.1027.

#### Benzyl *tert*-Butyl (1*H*-Indole-3,4-diyl)dicarbamate **20**

**Caution! Azides are potentially explosive substances and can decompose violently**. 4-(((Benzyloxy)carbonyl)amino)-1*H*-indole-3-carboxylic acid **19** (500 mg, 1.53 mmol, 1 equiv) was suspended in DCM (25 mL) under nitrogen. Triethylamine (0.43 mL, 3.06 mmol, 2 equiv) was added, and the mixture was stirred until everything had dissolved. DPPA (0.38 mL, 1.68 mmol, 1.1 equiv) was added dropwise, and the reaction mixture was stirred at room temperature overnight. 25 mL of DCM was added, and the mixture was extracted with 25 mL of 1 N HCl. The aqueous layer was extracted a further 2x with 25 mL of DCM, and the combined organic layers were washed with 25 mL of saturated NaHCO_3_ and brine, dried over MgSO_4_, and passed through a phase separator into a well-dried flask, with the volume reduced by half in vacuo. The solution was diluted with *tert*-butanol (25 mL), and then, the volume reduced to ∼20 mL to remove the residual DCM. Acetic acid (0.18 mL, 3.06 mmol, 2 equiv) was added, and the reaction mixture was heated to 60 °C under nitrogen for 3 days. The reaction mixture was cooled to room temperature and evaporated to dryness. The residue was dissolved in 25 mL of DCM and washed with 25 mL of water. The aqueous layer was extracted 2x with 25 mL of DCM, and the combined organics were washed with 25 mL of saturated NaHCO_3_ and 25 mL of brine, dried over MgSO_4_, passed through a phase separator, and evaporated to dryness. The residue was purified by flash chromatography (0–50% ethyl acetate in heptane) to give benzyl *tert*-butyl (1*H*-indole-3,4-diyl)dicarbamate **20** (389 mg, 63%) as a white powder. ^1^H (500 MHz, DMSO-*d*_6_): δ 11.01 (1H, br s, H1) 8.83 (1H, br s, Cbz NH), 8.42 (1H, br s, Boc NH), 7.40 (5H, m, Cbz-CH), 7.25 (1H, d, *J* = 6.1 Hz, H5), 7.21 (1H, d, *J* = 1.4 Hz, H2), 7.13 (1H, d, *J* = 8.2 Hz, H7), 7.05 (1H, t, *J* = 7.9 Hz, H6), 5.17 (2H, s, CH_2_), 1.41 (9H, s, CH_3_); ^13^C{^1^H} (126 MHz, DMSO-*d*_6_): δ 155.6 (Boc C=O), 153.9 (Cbz C=O), 136.6 (Cbz C), 135.7 (C7a), 129.5 (C4), 128.4 (Cbz-CH), 128.0 (Cbz-CH), 128.0 (Cbz-CH), 121.4 (C5), 120.2 (C2), 116.3 (C3a), 112.6 (C3), 112.0 (C5), 108.1 (H7), 78.8 (**C**(CH_3_)_3_), 65.9 (CH_2_), 20.1 (CH_3_); ^15^N (50 MHz, DMSO-*d*_6_): δ 128.4 (N1), 109.7 (Boc NH), 967 (Cbz NH); HRMS (ESI/TOF) *m*/*z*: [M + H]^+^ calcd for C_21_H_24_N_3_O_4_, 382.1761; found, 382.1763.

### General Method 1—Synthesis of Trifluoroketone **25**

The appropriate nitroindole (1 equiv) was dissolved in DMF (5 mL/mmol) under nitrogen, and TFAA (2 equiv) was added. The reaction mixture was heated to 80 °C overnight. The reaction mixture was diluted with water and extracted 3x with ethyl acetate, and the combined organic layers were washed 2x with 10% LiCl solution and brine, dried over MgSO_4_, passed through a phase separator, and evaporated to dryness azeotroping 3x from toluene to give the products.

#### 2,2,2-Trifluoro-1-(5-nitro-1*H*-indol-3-yl)ethan-1-one **25a**

2,2,2-Trifluoro-1-(5-nitro-1*H*-indol-3-yl)ethan-1-one **25a** (723 mg, 100%) as a yellow powder. ^1^H{^19^F} (500 MHz, DMSO-*d*_6_): δ 13.20 (1H, br s, NH), 8.97 (1H, d, *J* = 2.2 Hz, H4), 8.74 (1H, s, H2), 8.21 (1H, dd, *J* = 8.9, 2.3 Hz, H6), 7.77 (1H, d, *J* = 9.0 Hz, H7); ^13^C{^1^H} (126 MHz, DMSO-*d*_6_): δ 174.3 (d, *J* = 35.2 Hz, C=O), 143.7 (C5), 140.9 (q, *J* = 4.2 Hz, C2), 139.9 (C7a), 125.2 (C3a), 119.6 (C6), 117.1 (C4), 113.9 (C7), 110.0 (C3); ^19^F{^1^H} (470 MHz, DMSO-*d*_6_): δ −71.94 (s); HRMS (ESI/TOF) *m*/*z*: [M – H]^−^ calcd for C_10_H_4_N_2_O_3_F_3_, 257.0180; found, 257.0191.

#### 2,2,2-Trifluoro-1-(6-nitro-1*H*-indol-3-yl)ethan-1-one **25b**

2,2,2-Trifluoro-1-(6-nitro-1*H*-indol-3-yl)ethan-1-one **25b** (745 mg, 99%) as a brown powder. ^1^H{^19^F} (500 MHz, DMSO-*d*_6_): δ 13.19 (1H, br s, NH), 8.80 (1H, s, H2), 8.44 (1H, d, *J* = 2.1 Hz, H7), 8.33 (1H, d, *J* = 8.8 Hz, H4), 8.19 (1H, dd, *J* = 8.8, 2.1 Hz, H5); ^13^C{^1^H} (126 MHz, DMSO-*d*_6_): δ 174.2 (d, *J* = 34.9 Hz, C=O), 144.1 (C6), 141.8 (q, *J* = 4.3 Hz, C2), 135.5 (C7a), 130.6 (C3a), 121.4 (C4), 118.2 (C5), 109.4 (C7), 108.9 (C3); ^19^F{^1^H} (470 MHz, DMSO-*d*_6_): δ −71.94 (s), HRMS (ESI/TOF) *m*/*z*: [M – H]– calcd for C_10_H_4_N_2_O_3_F_3_, 257.0180; found, 257.0187.

#### 2,2,2-Trifluoro-1-(7-nitro-1*H*-indol-3-yl)ethan-1-one **25c**

2,2,2-Trifluoro-1-(7-nitro-1*H*-indol-3-yl)ethan-1-one **25c** (462 mg, 100%) as an orange powder. ^1^H{^19^F} (500 MHz, DMSO-*d*_6_): δ 13.14 (1H, br s, NH), 8.62 (1H, dd, *J* = 7.9, 1.1 Hz, H4), 8.37 (1H, s, H2), 8.28 (1H, dd, *J* = 8.1, 1.1 Hz, H6), 7.56 (1H, t, *J* = 8.0 Hz, H5); ^13^C{^1^H} (126 MHz, DMSO-*d*_6_): δ 174.5 (d, *J* = 35.1 Hz, C=O), 138.9 (q, *J* = 4.3 Hz, C2), 133.8 (C7), 129.0 (C3a), 128.9 (C4), 128.8 (C7a), 123.4 (C5), 121.0 (C6), 116.4 (q, *J* = 291.2 Hz, CF_3_), 109.5 (C3); ^19^F{^1^H} (470 MHz, DMSO-*d*_6_): δ −72.00 (s), HRMS (ESI/TOF) *m*/*z*: [M – H]^−^ calcd for C_10_H_4_N_2_O_3_F_3_, 257.0180; found, 257.0177.

### General Method 2—Synthesis of Carboxylic Acid **26**

The appropriate trifluoroketone **25** (1 equiv) was suspended in 4 M sodium hydroxide solution (20 equiv) and stirred at 60 °C overnight. The reaction mixture was cooled to room temperature, diluted with water, and extracted 3x with diethyl ether. The aqueous layer was acidified by addition of concentrated HCl to pH 1, and the precipitate was isolated by vacuum filtration. The precipitate was washed 2x with water, 5x with diethyl ether, and then dried under vacuum to give the desired products.

#### 5-Nitro-1*H*-indole-3-carboxylic Acid **26a**

5-Nitro-1*H*-indole-3-carboxylic acid **26a** (419 mg, 90%) as a yellow powder. ^1^H (500 MHz, DMSO-*d*_6_): δ 12.48 (2H, m, NH, COOH), 8.89 (1H, d, *J* = 1.9 Hz, H4), 8.26 (1H, s, H2), 8.09 (1H, dd, *J* = 9.0, 2.0 Hz, H6), 7.67 (1H, d, *J* = 9.0 Hz, H7); ^13^C{^1^H} (126 MHz, DMSO-*d*_6_): δ 165.1 (C=O), 142.2 (C5), 139.6 (C7a), 135.6 (C2), 125.3 (C3a), 117.5 (C6), 117.0 (C4), 113.0 (C7), 109.5 (C3); HRMS (ESI/TOF) *m*/*z*: [M – H]^−^ calcd for C_9_H_5_N_2_O_4_, 205.0255; found, 205.0266.

#### 6-Nitro-1*H*-indole-3-carboxylic Acid **26b**

6-Nitro-1*H*-indole-3-carboxylic acid **26b** (388 mg, 88%) as a yellow powder. ^1^H (500 MHz, DMSO-*d*_6_): δ 12.53 (1H, br s, NH), 12.35 (1H, br s, COOH), 8.40 (1H, d, *J* = 2.0 Hz, H7), 8.37 (1H, d, *J* = 3.0 Hz, H2), 8.16 (1H, d, *J* = 8.9 Hz, H4), 8.05 (1H, dd, *J* = 8.9, 2.1 Hz, H5); ^13^C{^1^H} (126 MHz, DMSO-*d*_6_): δ 165.1 (C=O), 142.7 (C6), 137.7 (C2), 135.0 (C7a), 130.8 (C3a), 120.8 (C4), 116.0 (C5), 108.9 (C7), 108.3 (C3); HRMS (ESI/TOF) *m*/*z*: [M – H]^−^ calcd for C_9_H_5_N_2_O_4_; found, 205.0260.

#### 7-Nitro-1*H*-indole-3-carboxylic Acid **26c**

7-Nitro-1*H*-indole-3-carboxylic acid **26c** (233 mg, 95%) as a yellow powder. ^1^H (500 MHz, DMSO-*d*_6_): δ 12.44 (2H, br s, NH, COOH), 8.50 (1H, d, *J* = 7.8 Hz, H6), 8.18 (1H, d, *J* = 8.0 Hz, H4), 8.07 (1H, d, *J* = 2.8 Hz, H2), 7.41 (1H, t, *J* = 7.9 Hz, H5); ^13^C{^1^H} (126 MHz, DMSO-*d*_6_): δ 164.9 (C=O), 134.6 (C2), 133.2 (C7), 129.6 (C3a), 128.8 (C6), 128.5 (C7a), 120.9 (C5), 119.45 (C4), 109.0 (C3); HRMS (ESI/TOF) *m*/*z*: [M – H]^−^ calcd for C_9_H_5_N_2_O_4_, 205.0255; found, 205.0262.

### General Method 3—Synthesis of MOM Ester **27**

The appropriate carboxylic acid **26** (1 equiv) was suspended in THF (5 mL/mmol) under nitrogen and cooled to 0 °C. Triethylamine (3 equiv) was added, and the reaction mixture was stirred at 0 °C for 5 min. MOMCl (1.5 equiv) was added dropwise, and the reaction mixture was allowed to warm up to room temperature overnight. The reaction mixture was diluted with water and extracted 3x with ethyl acetate. The combined organic extracts were washed with saturated NaHCO_3_ solution and brine, dried over MgSO_4_, passed through a phase separator, and evaporated to dryness. The residue was purified by flash chromatography (0–50% ethyl acetate in heptane) to give the desired products.

#### Methoxymethyl 5-Nitro-1*H*-indole-3-carboxylate **27a**

Methoxymethyl 5-nitro-1*H*-indole-3-carboxylate **27a** (491 mg, 40%) as a yellow powder. ^1^H (500 MHz, DMSO-*d*_6_): δ 12.62 (1H, br s, NH), 8.88 (1H, d, *J* = 2.3 Hz, H4), 8.43 (1H, s, H2), 8.11 (1H, dd, *J* = 9.0, 2.3 Hz, H6), 7.69 (1H, d, *J* = 9.0 Hz, H7), 5.47 (2H, s, CH_2_), 3.49 (3H, s, CH_3_); ^13^C{^1^H} (126 MHz, DMSO-*d*_6_): δ 162.9 (C=O), 142.5 (C5), 139.6 (C7a), 136.7 (C2), 125.0 (C3a), 117.8 (C6), 116.8 (C4), 113.3 (C7), 108.0 (C3) 89.4 (CH_2_), 56.9 (CH_3_); HRMS (ESI/TOF) *m*/*z*: [M + H]^+^ calcd for C_11_H_11_N_2_O_5_, 251.0662; found, 251.0662.

#### Methoxymethyl 6-Nitro-1*H*-indole-3-carboxylate **27b**

Methoxymethyl 6-nitro-1*H*-indole-3-carboxylate **27b** (642 mg, 56%) as a yellow powder. ^1^H (500 MHz, DMSO-*d*_6_): δ 12.63 (1H, br s, NH), 8.52 (1H, s, H2), 8.42 (1H, d, *J* = 2.0 Hz, H7), 8.17 (1H, d, *J* = 8.8 Hz, H4), 8.09 (1H, dd, *J* = 8.9, 2.0 Hz, H5), 5.45 (2H, s, CH_2_), 3.48 (3H, s, CH_3_); ^13^C{^1^H} (126 MHz, DMSO-*d*_6_): δ 162.9 (C=O), 142.9 (C6), 138.5 (C2), 135.2 (C7a), 130.5 (C3a), 120.6 (C4), 116.5 (C5), 109.1 (C7), 106.9 (C3), 89.3 (CH_2_), 56.9 (CH_3_); HRMS (ESI/TOF) *m*/*z*: [M + H]^+^ calcd for C_11_H_11_N_2_O_5_, 251.0662; found, 251.0659.

#### Methoxymethyl 7-Nitro-1*H*-indole-3-carboxylate **27c**

Methoxymethyl 7-nitro-1*H*-indole-3-carboxylate **27c** (989 mg, 86%) as a yellow powder. ^1^H (500 MHz, DMSO-*d*_6_): δ 12.61 (1H, br s, NH), 8.50 (1H, d, *J* = 7.9 Hz, H6), 8.21 (1H, d, *J* = 8.1 Hz, H4), 8.20 (1H, s, H2), 7.46 (1H, t, *J* = 8.0 Hz, H5), 5.45 (2H, s, CH_2_), 3.48 (3H, s, CH_3_); ^13^C{^1^H} (126 MHz, DMSO-*d*_6_): δ 162.9 (C=O), 135.4 (C2), 133.4 (C7), 129.3 (C3a), 128.6 (C6), 121.4 (C5), 119.7 (C4), 107.7 (C3), 89.4 (CH_2_), 56.9 (CH_3_); HRMS (ESI/TOF) *m*/*z*: [M + H]^+^ calcd for C_11_H_11_N_2_O_5_, 251.0662; found, 251.0656.

### General Method 4—Reduction of Nitro Group **28**

The appropriate nitro compound **27** (1 equiv) was dissolved in methanol, THF, and water (1:1:1) (25 mL/mmol). Ammonium chloride (6.7 equiv) was added, followed by iron (4.2 equiv), and the reaction mixture was stirred at 60 °C overnight. The reaction mixture was filtered through Celite, rinsing with methanol, and the volatiles were removed *in vacuo*. The aqueous residue was diluted with water, saturated NaHCO_3_ solution, and extracted 3x with ethyl acetate. The combined organics were washed with brine, dried over MgSO_4_, passed through a phase separator, and evaporated to dryness to give the desired products.

#### Methoxymethyl 5-Amino-1*H*-indole-3-carboxylate **28a**

Methoxymethyl 5-amino-1*H*-indole-3-carboxylate **28a** (495 mg, 100%) as a brown gum. ^1^H (500 MHz, DMSO-*d*_6_): δ 11.55 (1H, br s, NH), 7.89 (1H, s, H2), 7.19 (1H, d, *J* = 1.9 Hz, H4), 7.16 (1H, d, *J* = 8.6 Hz, H7), 6.57 (1H, dd, *J* = 8.6, 2.1 Hz, H6), 5.36 (2H, s, CH_2_), 4.75 (2H, br s, NH_2_), 3.44 (3H, s, CH_3_); ^13^C{^1^H} (126 MHz, DMSO-*d*_6_): δ 163.8 (C=O), 143.7 (C5), 132.0 (C2), 129.6 (C7a), 127.1 (C3a), 112.6 (C6), 112.4 (C7), 104.6 (C3), 103.4 (C4) 84.4 (CH_2_), 56.6 (CH_3_); HRMS (ESI/TOF) *m*/*z*: [M + H]^+^ calcd for C_11_H_13_N_2_O_3_, 221.0921; found, 221.0920.

#### Methoxymethyl 6-Amino-1*H*-indole-3-carboxylate **28b**

Methoxymethyl 6-amino-1*H*-indole-3-carboxylate **28b** (573 mg, 100%) as an orange powder. ^1^H (500 MHz, DMSO-*d*_6_): δ 11.38 (1H, br s, NH), 7.80 (1H, s, H2), 7.63 (1H, d, *J* = 8.5 Hz, H4), 6.62 (1H, d, *J* = 1.4 Hz, H7), 6.56 (1H, dd, *J* = 8.5, 1.8 Hz, H5), 5.36 (2H, s, CH_2_), 4.89 (2H, br s, NH_2_), 3.43 (3H, s, CH_3_); ^13^C{^1^H} (126 MHz, DMSO-*d*_6_): δ 163.7 (C=O), 145.0 (C6), 138.1 (C7a), 130.1 (C2), 120.5 (C4), 117.0 (C3a), 112.0 (C5), 106.0 (C3), 95.5 (C7), 88.5 (CH_2_), 56.6 (CH_3_); HRMS (ESI/TOF) *m*/*z*: [M + H]^+^ calcd for C_11_H_13_N_2_O_3_, 221.0921; found, 221.0919.

#### Methoxymethyl 7-Amino-1*H*-indole-3-carboxylate **28c**

Methoxymethyl 7-amino-1*H*-indole-3-carboxylate **28c** (930.4 mg, 99%) as a gray powder. ^1^H (500 MHz, DMSO-*d*_6_): δ 11.56 (1H, br s, NH), 8.07 (1H, s, H2), 7.26 (1H, d, *J* = 7.9 Hz, H4), 6.91 (1H, t, *J* = 7.7 Hz, H5), 6.43 (1H, d, *J* = 7.5 Hz, H6), 5.39 (2H, s, CH_2_), 5.19 (2H, br s, NH_2_), 3.45 (3H, s, CH_3_); ^13^C{^1^H} (126 MHz, DMSO-*d*_6_): δ 164.3 (C=O), 134.9 (C7), 132.1 (C2), 127.1 (C3a), 126.2 (C7a), 123.1 (C5), 109.1 (C4), 106.8 (C3), 106.6 (C6), 89.2 (CH_2_), 57.2 (CH_3_); HRMS (ESI/TOF) *m*/*z*: [M + H]^+^ calcd for C_11_H_13_N_2_O_3_, 221.0921; found, 221.0923.

### General Method 5—Protection of Amine **29**

The appropriate amine **28** (1 equiv) was dissolved in DCM (10 mL/mmol) with pyridine (5 equiv) under nitrogen. CbzCl (2.4 equiv) was added, and the reaction mixture was stirred at room temperature overnight. The reaction mixture was diluted with water and extracted 3x with DCM. The combined organics were washed with brine, dried over MgSO_4_, passed through a phase separator, and evaporated to dryness. The residue was purified by flash chromatography (0–100% ethyl acetate in heptane) to give the desired products.

#### Methoxymethyl 5-(((Benzyloxy)carbonyl)amino)-1*H*-indole-3-carboxylate **29a**

Methoxymethyl 5-(((benzyloxy)carbonyl)amino)-1*H*-indole-3-carboxylate **29a** (492 mg, 75%) as an off-white powder. ^1^H (500 MHz, DMSO-*d*_6_): δ 11.89 (1H, br s, NH), 9.63 (1H, br s, NH), 8.21 (1H, br s, H4), 8.09 (1H, s, H2), 7.44 (2H, d, *J* = 7.3 Hz, Cbz-CH), 7.40 (3H, m, H7, Cbz-CH), 7.33 (2H, m, H6, Cbz-CH), 5.40 (2H, s, MOM-CH_2_), 5.16 (2H, s, Cbz-CH_2_), 3.48 (3H, s, CH_3_); ^13^C{^1^H} (126 MHz, DMSO-*d*_6_): δ 164.1 (ester C=O), 154.2 (carbamate C=O), 137.4 (Cbz-C), 133.9 (C2), 133.3 (C7a), 128.9 (Cbz-CH), 128.5 (Cbz-CH), 128.4 (Cbz-CH), 126.4 (C3a), 116.0 (C6), 112.8 (C7), 110.6 (C4), 106.4 (C3), 89.3 (MOM-CH_2_), 66.0 (Cbz-CH_2_), 57.3 (CH_3_); HRMS (ESI/TOF) *m*/*z*: [M + NH_4_]^+^ calcd for C_19_H_22_N_3_O_5_, 372.1554; found, 372.1566.

#### Methoxymethyl 6-(((Benzyloxy)carbonyl)amino)-1*H*-indole-3-carboxylate **29b**

Methoxymethyl 6-(((benzyloxy)carbonyl)amino)-1*H*-indole-3-carboxylate **29b** (439 mg, 48%) as an off-white powder. ^1^H (500 MHz, DMSO-*d*_6_): δ 11.86 (1H, br s, NH), 9.74 (1H, br s, NH), 8.05 (1H, s, H2), 7.85 (2H, m, H2, H4), 7.39 (5H, m, Cbz-CH), 7.21 (1H, dd, *J* = 8.6, 1.6 Hz, H5), 5.39 (2H, s, MOM-CH_2_), 5.17 (2H, s, Cbz-CH_2_), 3.45 (3H, s, CH_3_); ^13^C{^1^H} (126 MHz, DMSO-*d*_6_): δ 163.6 (ester C=O), 153.5 (carbamate C=O), 136.8 (C7a), 136.7 (Cbz-CH), 134.5 (C6), 132.5 (C2) 128.4 (Cbz-CH), 128.0 (Cbz-CH), 127.9 (Cbz-CH), 121.2 (C3a), 120.3 (C4), 113.9 (C5), 106.0 (C3), 101.6 (C7), 88.8 (MOM-CH_2_), 65.6 (Cbz-CH_2_), 56.7 (CH_3_); HRMS (ESI/TOF) *m*/*z*: [M + H]^+^ calcd for C_19_H_19_N_2_O_5_, 355.1288; found, 355.1294.

#### Methoxymethyl 7-(((Benzyloxy)carbonyl)amino)-1*H*-indole-3-carboxylate **29c**

Methoxymethyl 7-(((benzyloxy)carbonyl)amino)-1*H*-indole-3-carboxylate **29c** (1170 mg, 74%) as an off-white powder. ^1^H (500 MHz, DMSO-*d*_6_): δ 11.70 (1H, br s, NH), 9.53 (1H, br s, NH), 8.14 (1H, s, H2), 7.77 (1H, d, *J* = 8.0 Hz, H4), 7.43 (6H, m, Cbz-CH, H6), 7.17 (1H, d, *J* = 7.9 Hz, H5), 5.46 (2H, s, MOM-CH_2_), 5.21 (2H, s, Cbz-CH_2_), 3.46 (3H, s, CH_3_); ^13^C{^1^H} (126 MHz, DMSO-*d*_6_): δ 163.5 (ester C=O), 153.67 (carbamate C=O), 136.4 (Cbz-C), 132.7 (C2), 128.4 (Cbz-CH), 128.1 (Cbz-CH), 128.0 (Cbz-CH), 127.0 (C3a), 124.1 (C7), 121.7 (C5), 116.0 (C4), 113.7 (C6), 106.4 (C3), 88.9 (MOM-CH_2_), 66.1 (Cbz-CH_2_), 56.7 (CH_3_); HRMS (ESI/TOF) *m*/*z*: [M + H]^+^ calcd for C_19_H_19_N_2_O_5_, 355.1288; found, 355.1303.

### General Method 6—Deprotection of MOM Ester **30**

The appropriate protected ester **29** (1 equiv) was dissolved in DCM (8 mL/mmol) under nitrogen and cooled to 0 °C. TFA (10 equiv) was added, and the reaction mixture was stirred at 0 °C for 1 h. The reaction mixture was evaporated to dryness, and the residue was purified by reverse-phase flash chromatography (C_18_ column) [5–95% acetonitrile in water (0.1% formic acid)] to give the desired products.

#### 5-(((Benzyloxy)carbonyl)amino)-1*H*-indole-3-carboxylic Acid **30a**

5-(((Benzyloxy)carbonyl)amino)-1*H*-indole-3-carboxylic acid **30a** (189 mg, 47%) as a white powder. ^1^H (500 MHz, DMSO-*d*_6_): δ 11.80 (1H, br s, COOH), 11.67 (1H, br s, NH), 9.55 (1H, br s, NH), 8.19 (1H, s, H4), 7.93 (1H, d, *J* = 3.0 Hz, H2), 7.44 (2H, d, *J* = 7.1 Hz, Cbz-CH), 7.40 (2H, t, *J* = 7.5 Hz, Cbz-CH), 7.34 (2H, m, H7, Cbz-CH), 7.26 (1H, d, *J* = 8.6 Hz, H6), 5.15 (2H, s, CH_2_); ^13^C{^1^H} (126 MHz, DMSO-*d*_6_): δ 165.8 (COOH), 153.6 (carbamate C=O), 136.9 (Cbz-C), 132.9 (C7a), 132.7 (C5), 132.6 (C2), 128.3 (Cbz-CH), 127.9 (Cbz-CH), 127.8 (Cbz-CH), 126.1 (C3a), 115.2 (C6), 112.0 (C7), 110.4 (C4), 107.2 (C3), 65.4 (Cbz-CH_2_); HRMS (ESI/TOF) *m*/*z*: [M – H]^−^ calcd for C_17_H_13_N_2_O_4_, 309.0881; found, 309.0883.

#### 6-(((Benzyloxy)carbonyl)amino)-1*H*-indole-3-carboxylic Acid **30b**

6-(((Benzyloxy)carbonyl)amino)-1*H*-indole-3-carboxylic acid **30b** (234 mg, 72%) as a white powder. ^1^H (500 MHz, DMSO-*d*_6_): δ 11.81 (1H, br s, COOH), 11.64 (1H, d, *J* = 1.4 Hz, NH) 9.70 (1H, br s, NH), 7.89 (1H, d, *J* = 2.8 Hz, H2), 7.84 (1H, d, *J* = 8.6 Hz, H4), 7.79 (1H, br s, H7), 7.44 (2H, d, *J* = 7.2 Hz, Cbz-CH), 7.40 (2H, t, *J* = 7.5 Hz, Cbz-CH), 7.34 (1H, t, *J* = 7.2 Hz, Cbz-CH), 7.16 (1H, dd, *J* = 8.6, 1.6 Hz, H5), 5.16 (2H, s, CH_2_); ^13^C{^1^H} (126 MHz, DMSO-*d*_6_): δ 165.8 (COOH), 153.5 (carbamate C=O), 136.7 (C7a), 136.7 (Cbz-C), 134.2 (C6), 131.6 (C2) 128.4 (Cbz-CH), 128.0 (Cbz-CH), 127.9 (Cbz-CH), 121.6 (C3a), 120.4 (C4), 113.5 (C5), 107.2 (C3), 101.4 (C7), 65.5 (CH_2_); HRMS (ESI/TOF) *m*/*z*: [M – H]^−^ calcd for C_17_H_13_N_2_O_4_, 309.0881; found, 309.0885.

#### 7-(((Benzyloxy)carbonyl)amino)-1*H*-indole-3-carboxylic Acid **30c**

7-(((Benzyloxy)carbonyl)amino)-1*H*-indole-3-carboxylic acid **30c** (332 mg, 40%) as a white powder. ^1^H (500 MHz, DMSO-*d*_6_): δ 11.91 (1H, br s, NH), 11.49 (1H, br s, COOH), 9.49 (1H, br s, NH), 7.98 (1H, d, *J* = 3.0 Hz, H2), 7.76 (1H, d, *J* = 8.0 Hz, H4), 7.41 (6H, m, Cbz-CH, H6), 7.10 (1H, t, *J* = 7.9 Hz, H5), 5.20 (2H, s, CH_2_); ^13^C{^1^H} (126 MHz, DMSO-*d*_6_): δ 163.7 (COOH), 153.7 (carbamate C=O), 136.5 (Cbz-C), 131.9 (C2), 128.4 (Cbz-CH), 128.1 (Cbz-CH), 128.0 (Cbz-CH), 127.3 (C3a), 123.8 (C7), 121.2 (C5), 116.2 (C4), 107.7 (C3), 66.0 (CH_2_); HRMS (ESI/TOF) *m*/*z*: [M – H]^−^ calcd for C_17_H_13_N_2_O_4_, 309.0881; found, 309.0885.

### General Method 7—Synthesis of Boc-Protected Amine **31**

**Caution! Azides are potentially explosive substances and can decompose violently**. The appropriate carboxylic acid **30** (1 equiv) was suspended in DCM (30 mL/mmol) under nitrogen. Triethylamine (2 equiv) was added and stirred until everything dissolved. DPPA (1.1 equiv) was added, and the reaction mixture was stirred at room temperature overnight. The reaction mixture was washed with 1 N HCl, and the aqueous layer was extracted 2x with DCM. The combined organics were washed with saturated NaHCO_3_ solution and brine, dried over MgSO_4_, and passed through a phase separator, with the volume reduced to ∼5 mL in vacuo. *t*-Butanol (30 mL/mmol) was added, and the residual DCM was removed *in vacuo*. Acetic acid (2 equiv) was added, and the reaction mixture was stirred at 60 °C for 24 h under nitrogen. The solvent was removed *in vacuo*, and the residue was partitioned between DCM and water. The layers were separated, and the aqueous layer was extracted 2x with DCM; the combined organics were washed with saturated NaHCO_3_ solution and brine, dried over MgSO_4_, passed through a phase separator, and evaporated to dryness. The residue was purified by flash chromatography (0–50% ethyl acetate in heptane) to give the desired products.

#### Benzyl *tert*-Butyl (1*H*-Iindole-3,5-diyl)dicarbamate **31a**

Benzyl *tert*-butyl (1*H*-indole-3,5-diyl)dicarbamate **31a** (10.7 mg, 17%) as a white powder. ^1^H (500 MHz, DMSO-*d*_6_): δ 10.60 (1H, br s, NH), 9.39 (1H, br s, NH), 8.88 (1H, br s, NH), 7.80 (1H, s, H4), 7.42 (4H, m, Cbz-CH), 7.30 (2H, m, H2, Cbz-CH), 7.20 (21, m, H7), 7.07 (1H, d, *J* = 7.5 Hz, H6), 5.15 (2H, s, CH_2_), 1.48 (9H, s, CH_3_); ^13^C{^1^H} (126 MHz, DMSO-*d*_6_): δ 153.8 (carbamate C=O), 137.0 (Cbz-C), 130.8 (C7a), 130.0 (C5), 128.4 (Cbz–CH), 127.9 (Cbz-CH), 127.8 (Cbz-CH), 121.6 (C3a), 116.5 (C2), 115.2 (C6), 114.9 (C3), 111.1 (C7), 108.8 (C4), 78.1 (**C**(CH3)_3_), 65.3 (Cbz-CH_2_), 28.22 (CH_3_); HRMS (ESI/TOF) *m*/*z*: [M + H]^+^ calcd for C_21_H_24_N_3_O_4_, 382.1761; found, 382.1764.

#### Benzyl *tert*-Butyl (1*H*-Indole-3,6-diyl)dicarbamate **31b**

Benzyl *tert*-butyl (1*H*-indole-3,6-diyl)dicarbamate **31b** (33 mg, 54%) as a white powder. ^1^H (500 MHz, DMSO-*d*_6_): δ 10.53 (1H, br s, NH), 9.55 (1H, br s, NH) 8.99 (1H, br s, NH), 7.61 (2H, m, H4, H7), 7.42 (4H, m, Cbz-CH), 7.34 (1H, t, *J* = 7.2 Hz, Cbz-CH), 7.29 (1H, br s, H2), 6.95 (1H, d, *J* = 8.6 Hz, H5), 5.15 (2H, s, CH_2_), 1.48 (9H, s, CH_3_); ^13^C{^1^H} (126 MHz, DMSO-*d*_6_): δ 153.4 (carbamate C=O), 153.3 (carbamate C=O), 136.8 (Cbz-C), 134.1 (C7a), 133.4 (C6), 128.4 (Cbz-CH), 128.0 (Cbz-CH), 127.9 (Cbz-CH), 118.1 (C4), 116.9 (C3a), 115.2 (C3), 113.5 (C2), 110.9 (C5), 100.7 (C7), 78.2 (**C**(CH_3_)_3_), 65.4 (CH_2_), 28.2 (CH_3_); HRMS (ESI/TOF) *m*/*z*: [M + H]^+^ calcd for C_21_H_24_N_3_O_4_, 382.1761; found, 382.1767.

#### Benzyl *tert*-Butyl (1*H*-Indole-3,7-diyl)dicarbamate **31c**

Benzyl *tert*-butyl (1*H*-indole-3,7-diyl)dicarbamate **31c** (16 mg, 26%) as a white powder. ^1^H (500 MHz, DMSO-*d*_6_): δ 10.44 (1H, br s, NH), 9.38 (1H, br s, NH), 9.05 (1H, br s, NH), 7.44 (8H, m, H2, H4, H5, Cbz-CH), 6.93 (1H, t, *J* = 7.8 Hz, H5), 5.21 (2H, s, CH_2_), 1.50 (9H, s, CH_3_); ^13^C{^1^H} (126 MHz, DMSO-*d*_6_): δ 153.8 (carbamate C=O), 153.5 (carbamate C=O), 136.6 (Cbz-C), 128.5 (Cbz-CH), 128.1 (Cbz-CH), 128.1 (Cbz-CH), 125.9 (C7a), 123.1 (C7), 122.3 (C3a), 118.2 (C5), 115.8 (C3), 114.4 (C4), 114.0 (C6), 112.3 (C2), 78.4 (**C**CH_3_), 66.00 (CH_2_), 28.3 (CH_3_); HRMS (ESI/TOF) *m*/*z*: [M + H]^+^ calcd for C_21_H_24_N_3_O_4_, 382.1761; found, 382.1754.

## Supporting Information Available

Copies of NMR spectra (PDF)
