# Supplementary material for: Synthesis of a Series of Diaminoindoles
Source: J Org Chem. 2021 Aug 5;86(17):11333–40. doi: 10.1021/acs.joc.1c00652 (PMC8419841; doi:10.1021/acs.joc.1c00652)
Supplement: Supplementary file 1 — jo1c00652_si_001.pdf [file jo1c00652_si_001.pdf]

Synthesis of a Series of Diaminoindoles.

James S. Martin, Claire J. Mackenzie, Ian H. Gilbert*

Division of Biological Chemistry and Drug Discovery, University of Dundee, Dundee, DD1 5EH, UK.

i.h.gilbert@dundee.ac.uk

NMR Spectra:

tert-Butyl (*tert*-butoxycarbonyl)(1-(triisopropylsilyl)-1*H*-indol-4-yl)carbamate **10**	S1
tert-Butyl (3-bromo-1-(triisopropylsilyl)-1*H*-indol-4-yl)carbamate **11**	S6
tert-Butyl 4-((*tert*-butoxycarbonyl)amino)-1-(triisopropylsilyl)-1*H*-indole-3-carboxylate **12**	S11
4-Nitro-1-(triisopropylsilyl)-1*H*-indole **14**	S16
1-(Triisopropylsilyl)-1*H*-indol-4-amine hydrochloride **15**	S21
tert-Butyl (1-(triisopropylsilyl)-1*H*-indol-4-yl)carbamate **16**	S26
tert-Butyl 4-((*tert*-butoxycarbonyl)amino)-1*H*-indole-3-carboxylate **17**	S31
4-(((Benzyloxy)carbonyl)amino)-1*H*-indole-3-carboxylic acid **19**	S36
Benzyl *tert*-butyl (1*H*-indole-3,4-diyl)dicarbamate **20**	S41
2,2,2-Trifluoro-1-(5-nitro-1*H*-indol-3-yl)ethan-1-one **25a**	S46
2,2,2-Trifluoro-1-(6-nitro-1*H*-indol-3-yl)ethan-1-one **25b**	S52
2,2,2-Trifluoro-1-(7-nitro-1*H*-indol-3-yl)ethan-1-one **25c**	S58
5-Nitro-1*H*-indole-3-carboxylic acid **26a**	S64
6-Nitro-1*H*-indole-3-carboxylic acid **26b**	S69
7-Nitro-1*H*-indole-3-carboxylic acid **26c**	S74
Methoxymethyl 5-nitro-1*H*-indole-3-carboxylate **27a**	S79
Methoxymethyl 6-nitro-1*H*-indole-3-carboxylate **27b**	S84
Methoxymethyl 7-nitro-1*H*-indole-3-carboxylate **27c**	S89
Methoxymethyl 5-amino-1*H*-indole-3-carboxylate **28a**	S94
Methoxymethyl 6-amino-1*H*-indole-3-carboxylate **28b**	S99
Methoxymethyl 7-amino-1*H*-indole-3-carboxylate **28c**	S104
Methoxymethyl 5-(((benzyloxy)carbonyl)amino)-1*H*-indole-3-carboxylate **29a**	S109
Methoxymethyl 6-(((benzyloxy)carbonyl)amino)-1*H*-indole-3-carboxylate **29b**	S114
Methoxymethyl 7-(((benzyloxy)carbonyl)amino)-1*H*-indole-3-carboxylate **29c**	S119
5-(((Benzyloxy)carbonyl)amino)-1*H*-indole-3-carboxylic acid **30a**	S124
6-(((Benzyloxy)carbonyl)amino)-1*H*-indole-3-carboxylic acid **30b**	S129
7-(((Benzyloxy)carbonyl)amino)-1*H*-indole-3-carboxylic acid **30c**	S134
Benzyl *tert*-butyl (1*H*-indole-3,5-diyl)dicarbamate **31a**	S139
Benzyl *tert*-butyl (1*H*-indole-3,6-diyl)dicarbamate **31b**	S144
Benzyl *tert*-butyl (1*H*-indole-3,7-diyl)dicarbamate **31c**	S149

^{1}H NMR (500 MHz, DMSO-d_6) δ 7.49 (d, J = 8.3 Hz, 1H), 7.39 (d, J = 3.2 Hz, 1H), 7.11 (t, J = 7.9 Hz, 1H), 6.86 (d, J = 7.5 Hz, 1H), 6.38 (d, J = 3.2 Hz, 1H), 1.75 (hept, J = 7.6 Hz, 3H), 1.35 (s, 18H), 1.09 (d, J = 7.5 Hz, 18H).

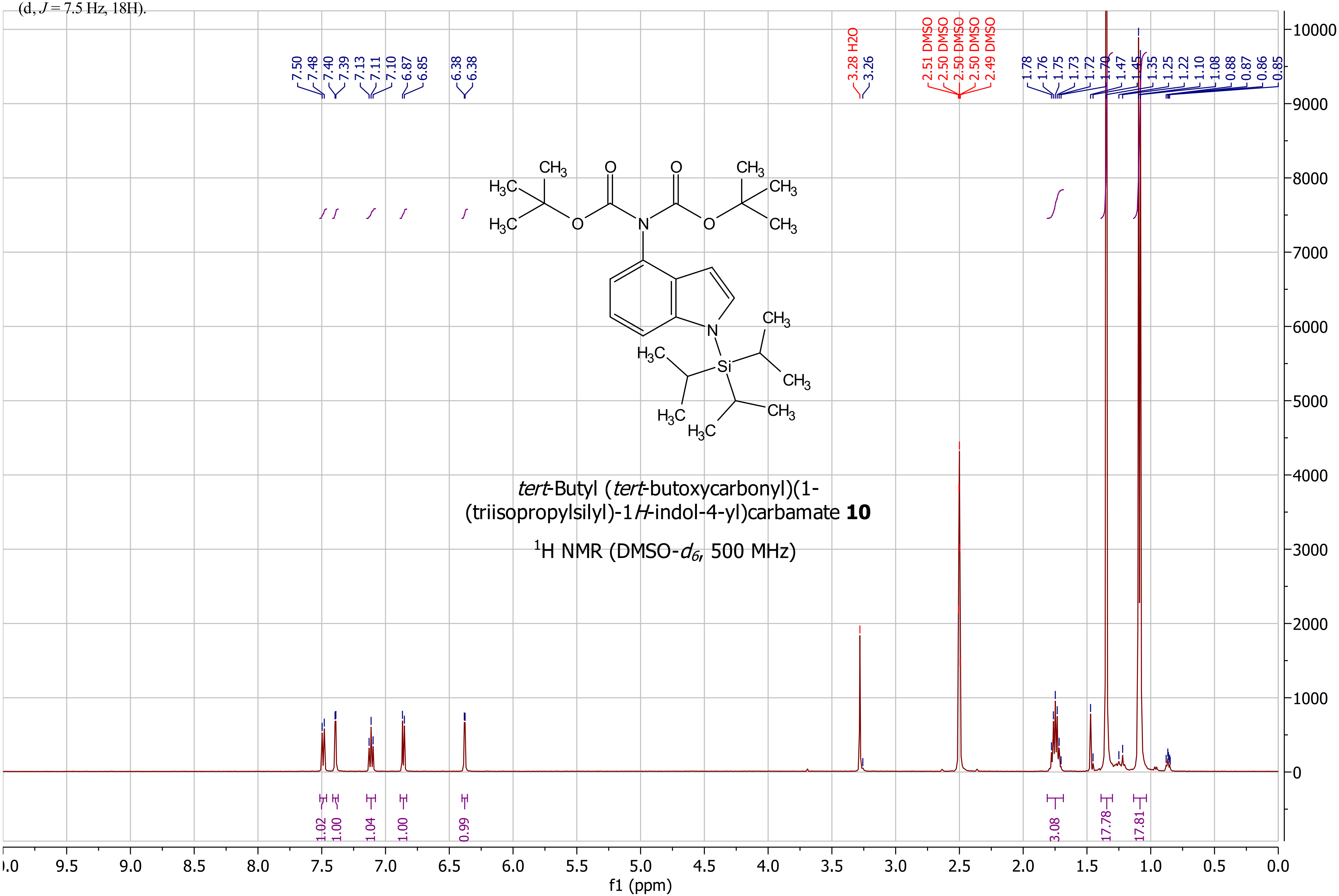

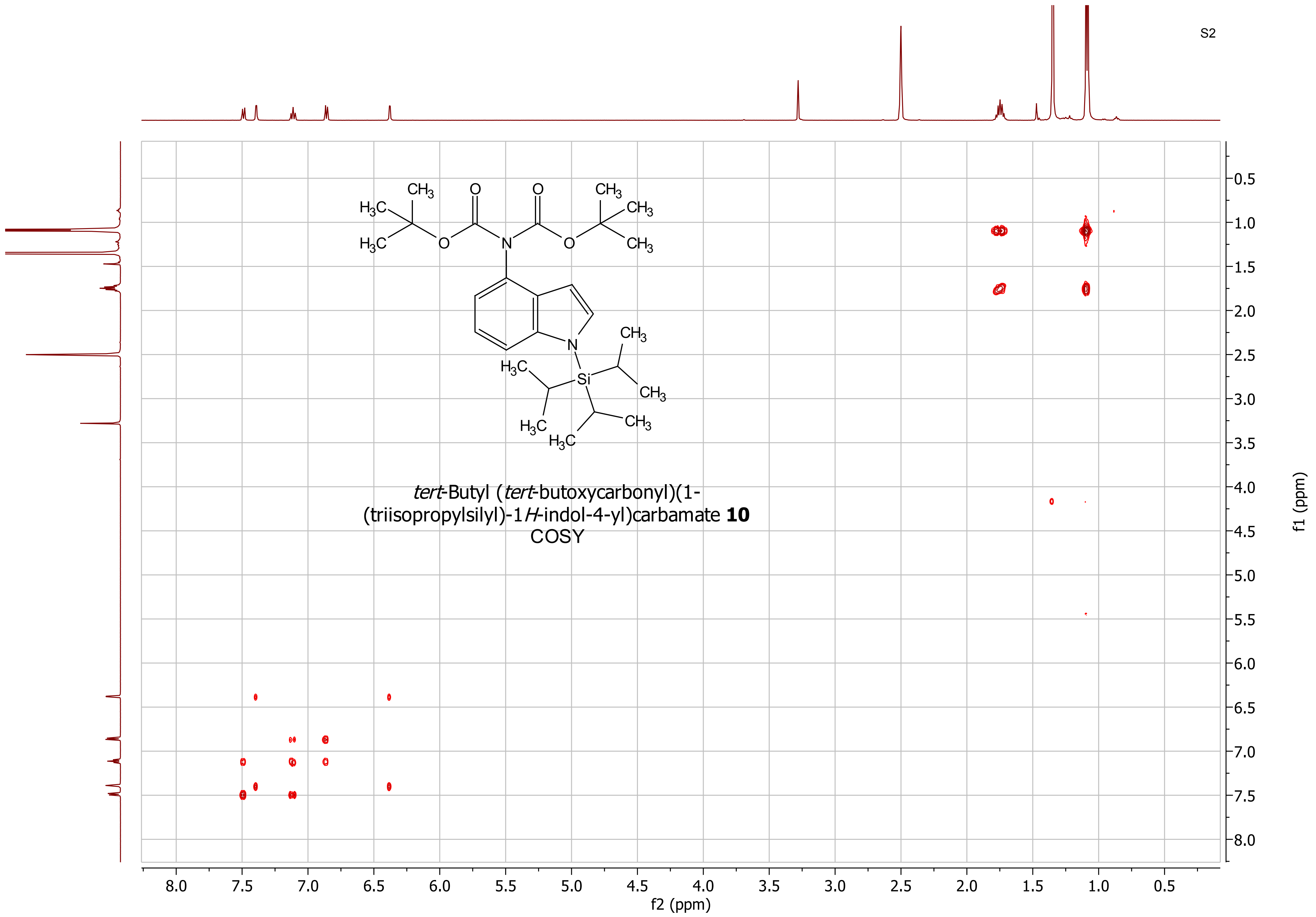
tert-Butyl (*tert*-butoxycarbonyl)(1-(triisopropylsilyl)-1*H*-indol-4-yl)carbamate **10**
COSY
f2 (ppm)
f1 (ppm)

^{13}C NMR (126 MHz, DMSO-d_6) δ 151.6, 141.3, 132.0, 130.9, 128.7, 121.2, 118.8, 112.9, 101.6, 81.7, 27.4, 17.8, 11.9.

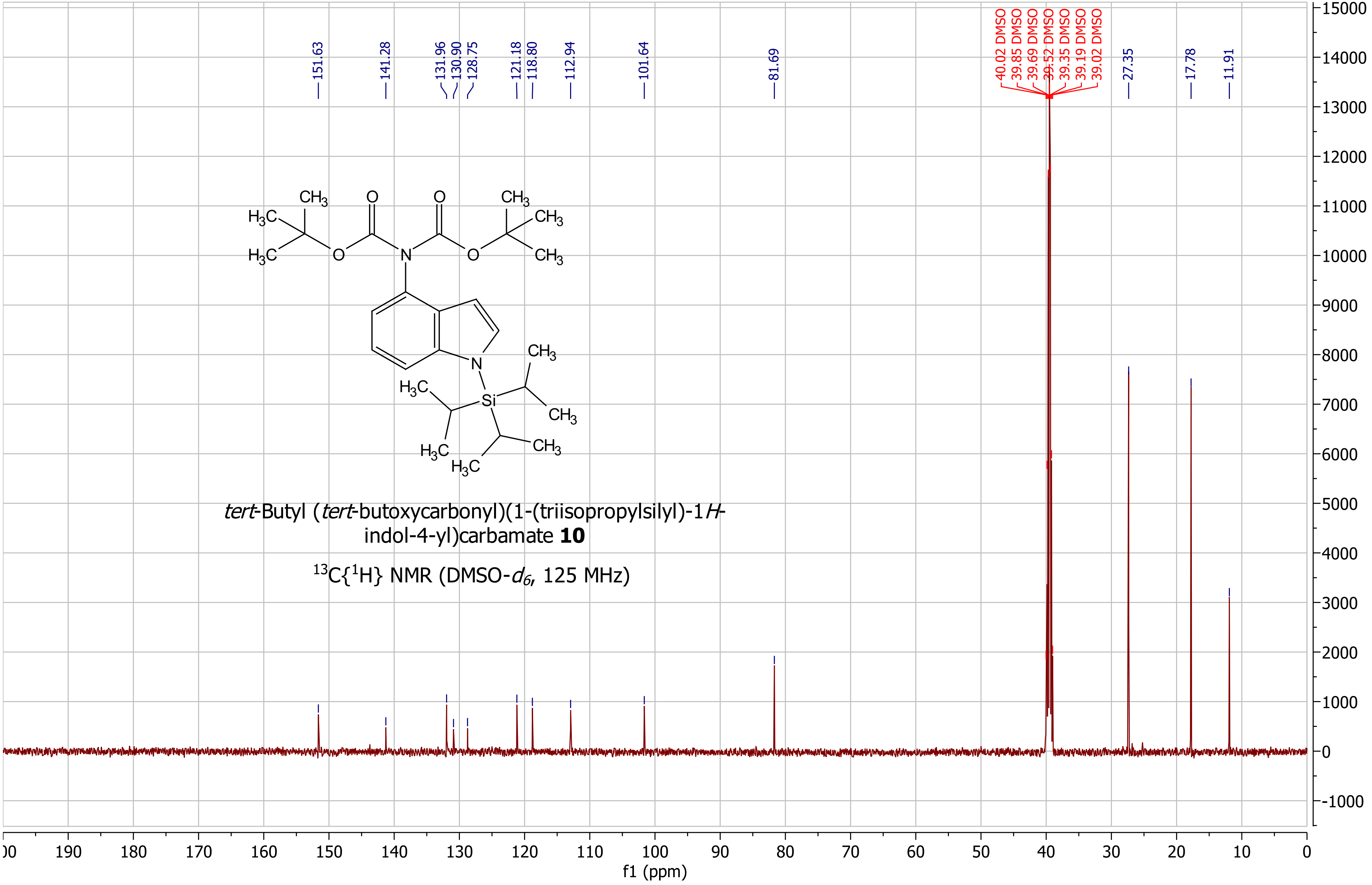

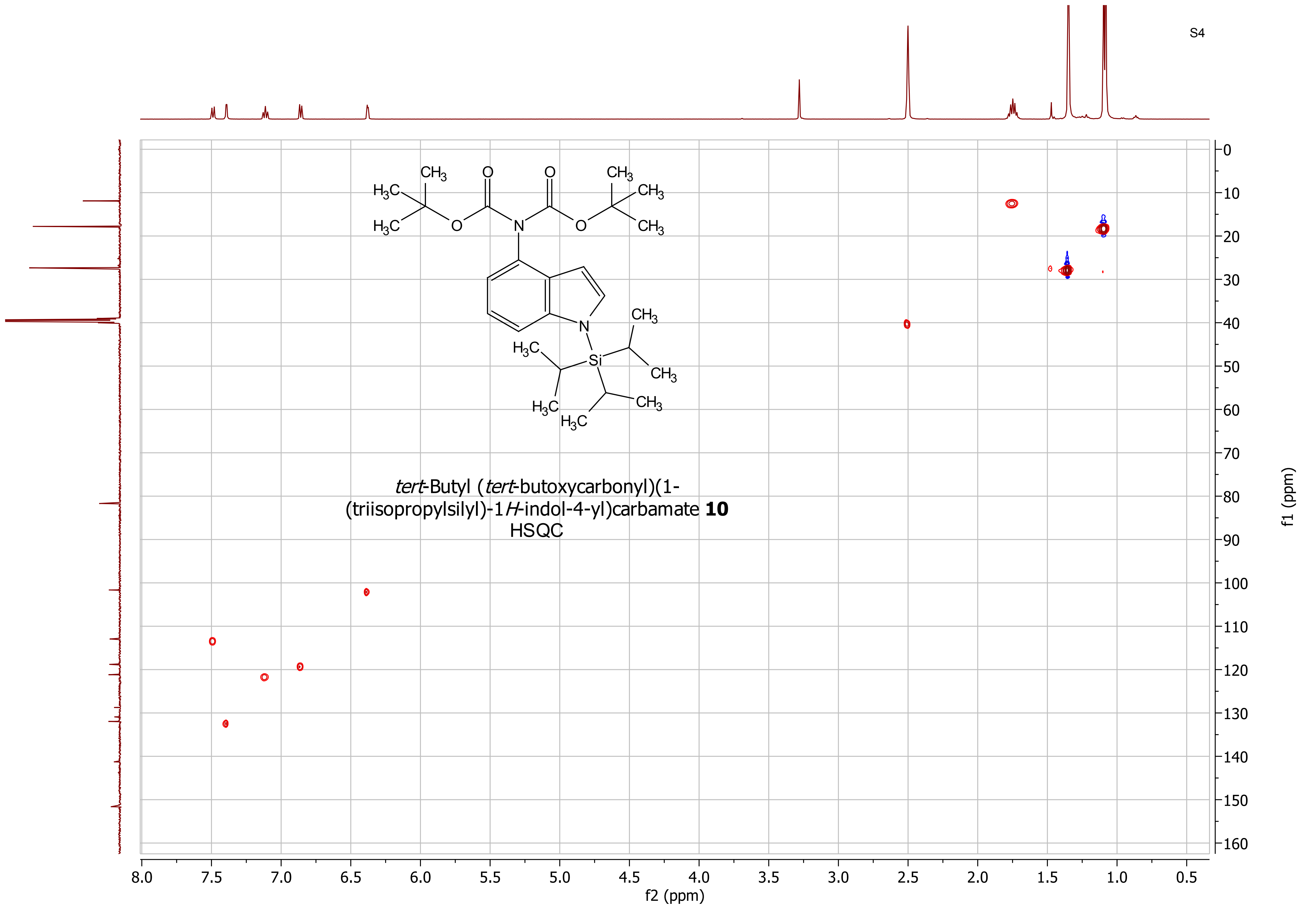

tert-Butyl (tert-butoxycarbonyl)(1-(triisopropylsilyl)-1H-indol-4-yl)carbamate 10
HSQC
f1 (ppm)
f2 (ppm)

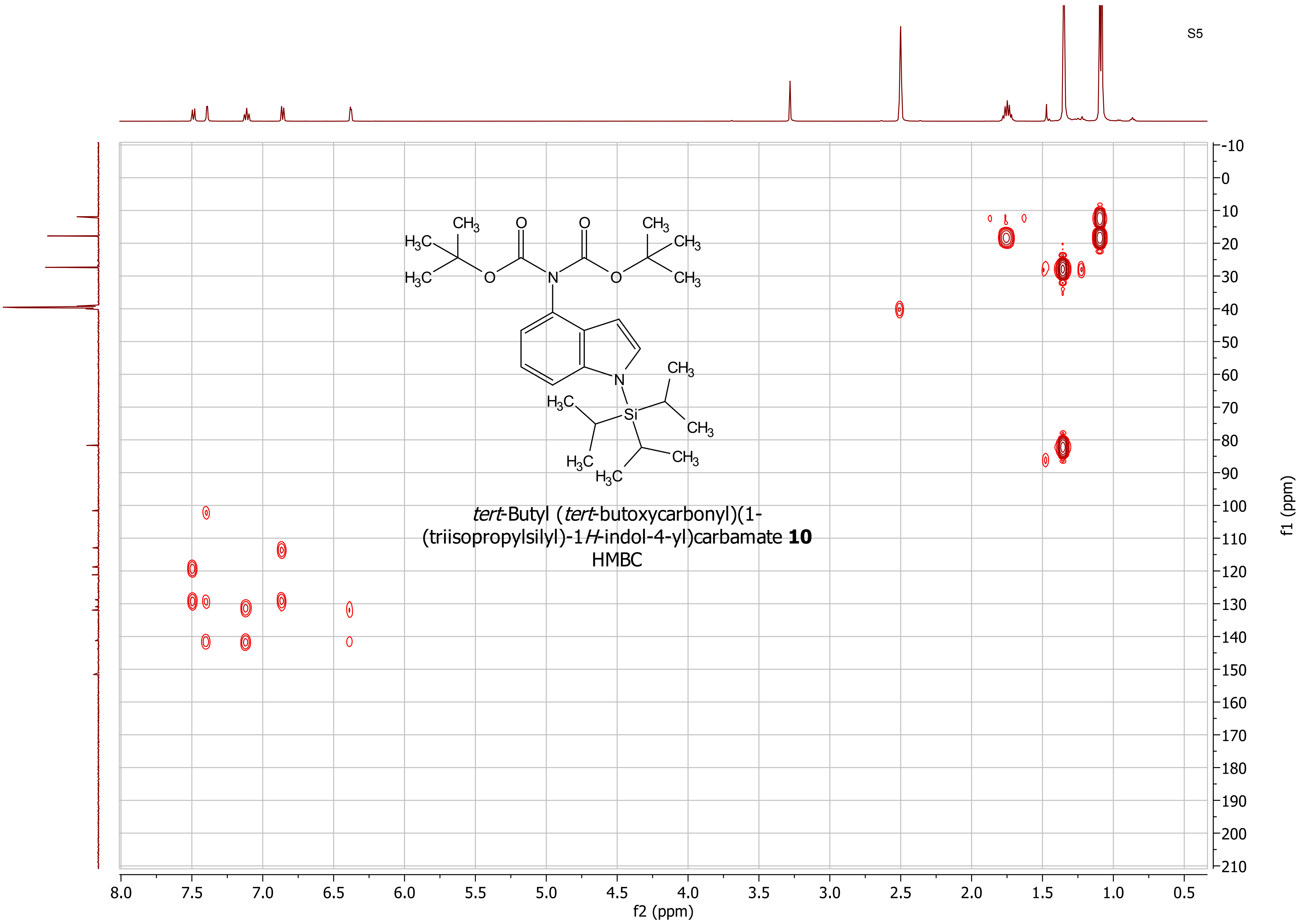

CH3
O
O
CH3
H3C
CH3
H3C
O
N
O
CH3
N
CH3
H3C
Si
CH3
CH3
H3C
H3C
tert-Butyl (tert-butoxycarbonyl)(1-(triisopropylsilyl)-1H-indol-4-yl)carbamate 10
HMBC
f1 (ppm)
f2 (ppm)

^{1}H NMR (500 MHz, DMSO-d_6) δ 7.56 (d, J = 8.5 Hz, 1H), 7.44 (s, 1H), 7.18 (t, J = 7.9 Hz, 1H), 6.90 (d, J = 7.4 Hz, 1H), 1.76 (hept, J = 7.5 Hz, 3H), 1.32 (s, 18H), 1.08 (d, J = 7.5 Hz, 18H).

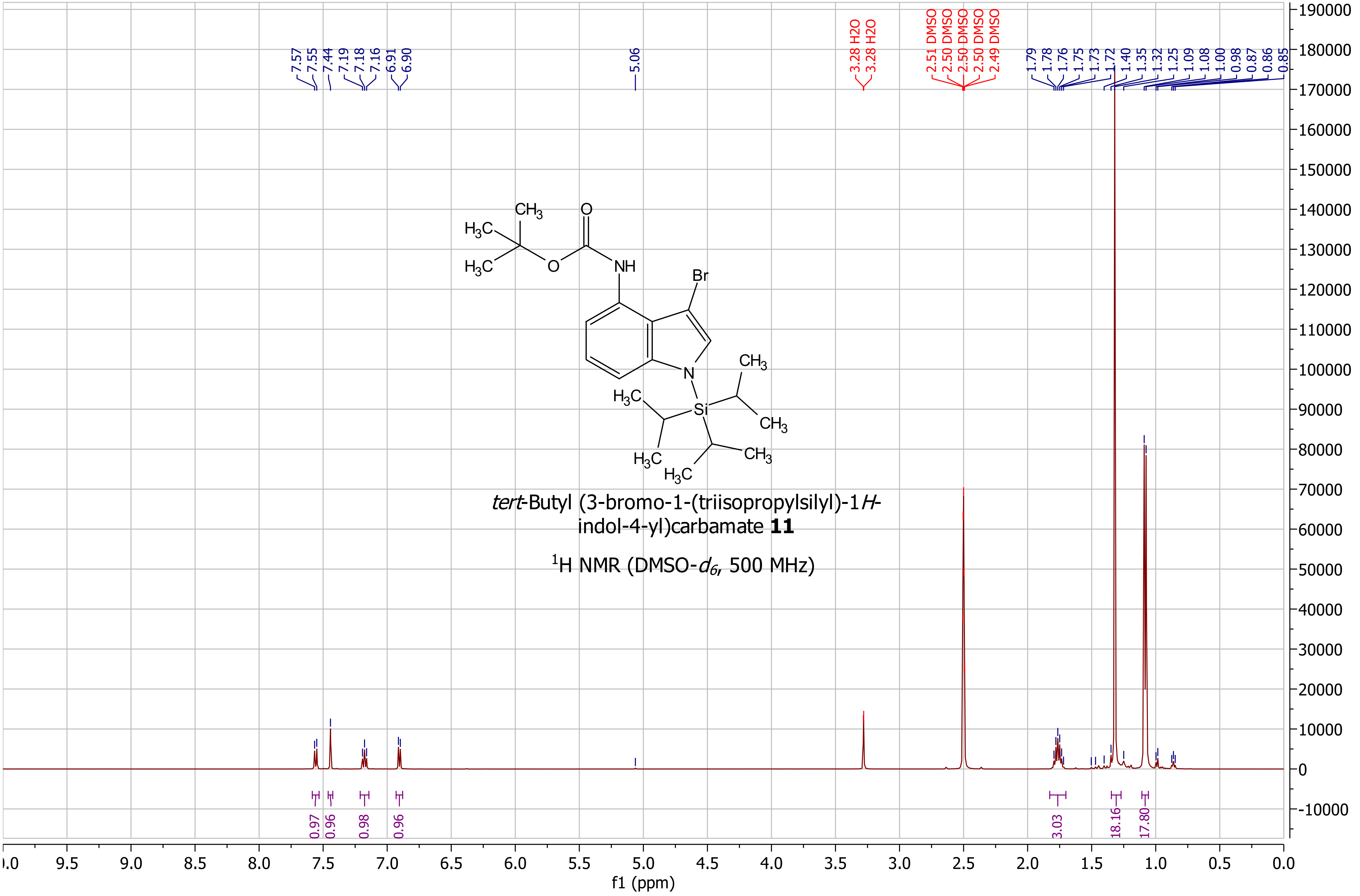

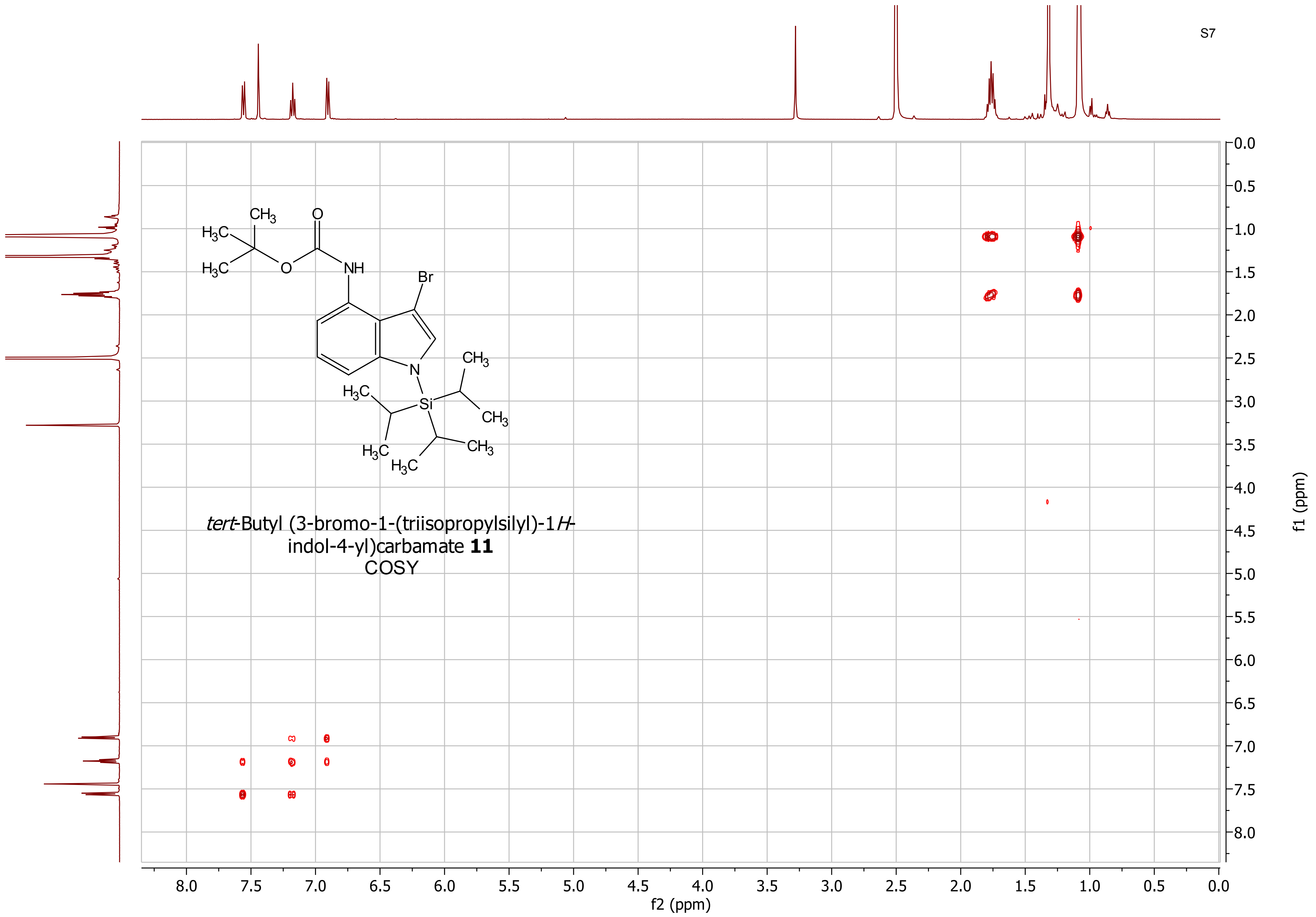
tert-Butyl (3-bromo-1-(triisopropylsilyl)-1H-indol-4-yl)carbamate 11
COSY
f2 (ppm)
f1 (ppm)

^{13}C NMR (126 MHz, DMSO-d_6) δ 151.0, 140.9, 131.3, 131.0, 125.1, 122.3, 120.8, 114.0, 89.6, 81.4, 27.5, 17.7, 11.8.

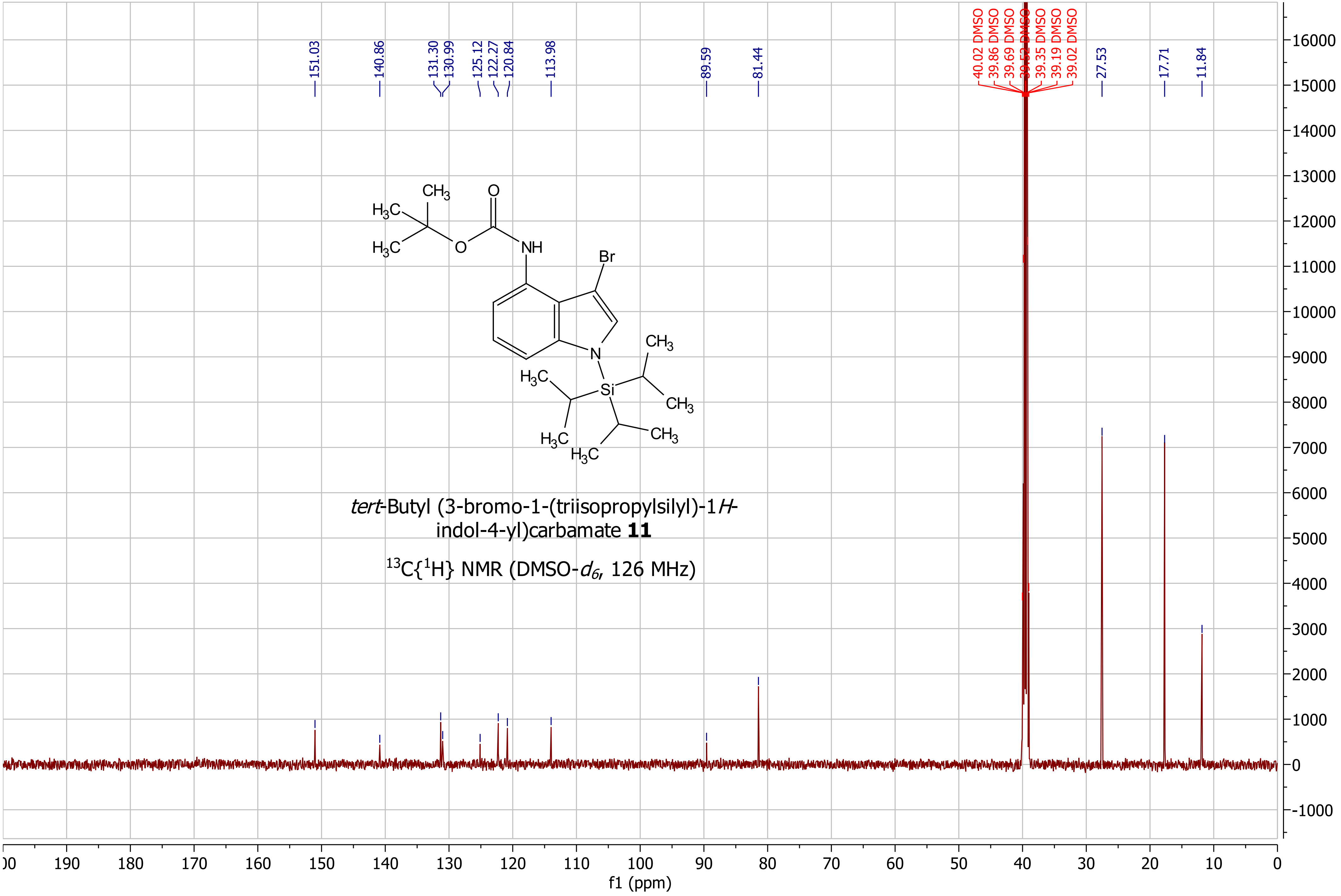

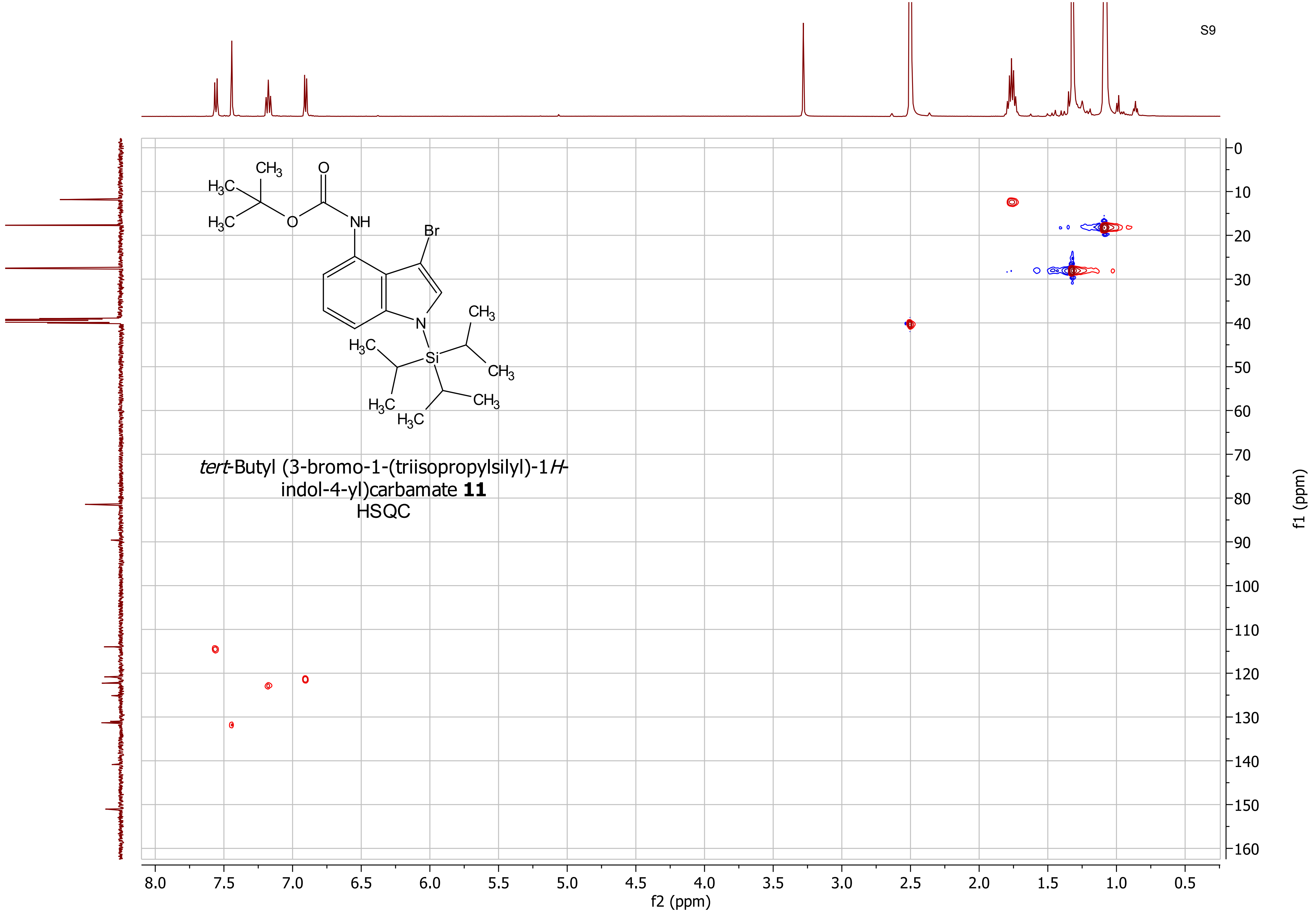

tert-Butyl (3-bromo-1-(triisopropylsilyl)-1*H*-indol-4-yl)carbamate **11**
HSQC

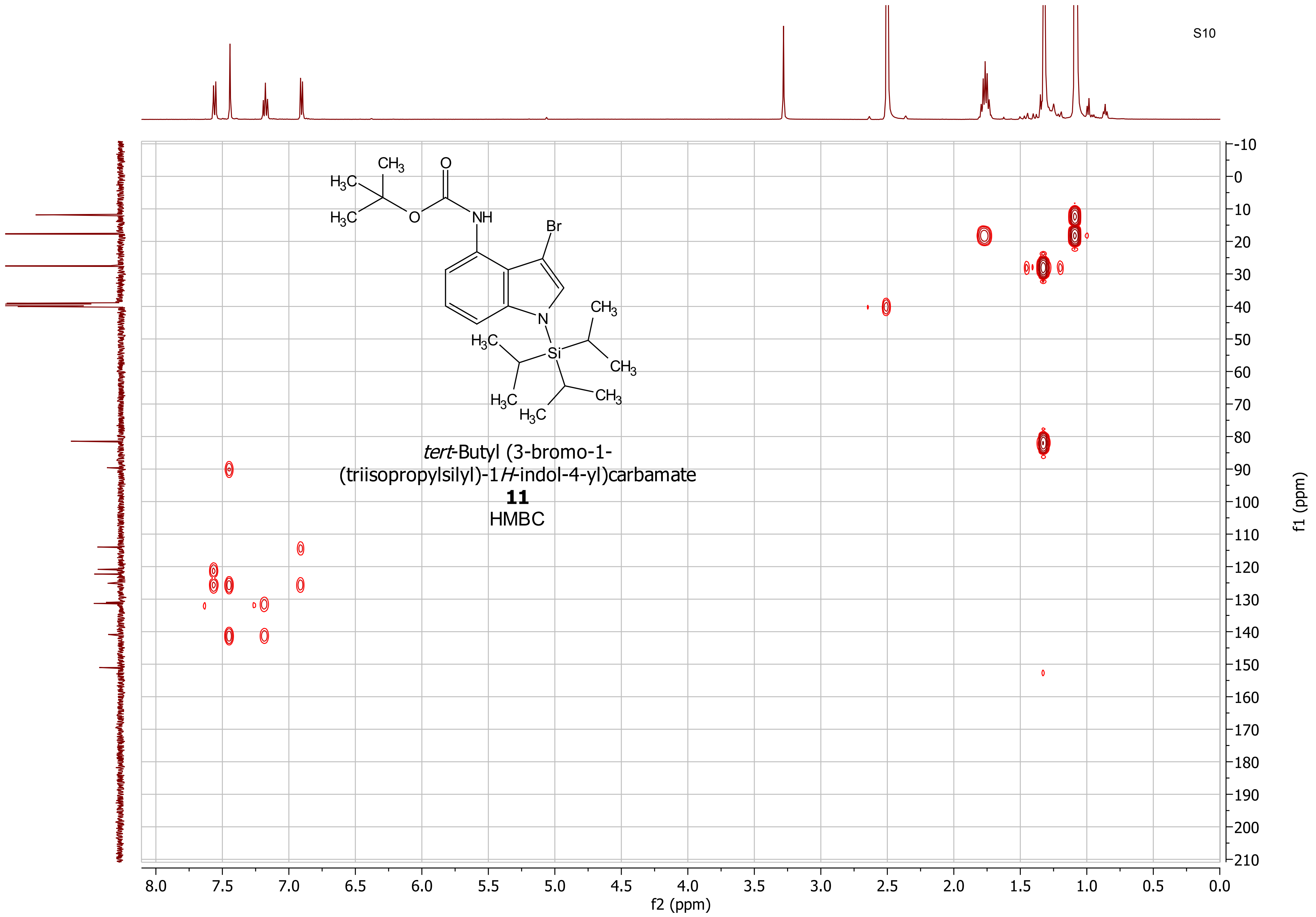

tert-Butyl (3-bromo-1-(triisopropylsilyl)-1*H*-indol-4-yl)carbamate
11
HMBC
f2 (ppm)
f1 (ppm)

^{1}H NMR (500 MHz, DMSO-d_6) δ 10.91 (s, 1H), 7.91 (d, J = 7.8 Hz, 1H), 7.89 (s, 1H), 7.23 (d, J = 8.3 Hz, 1H), 7.17 (t, J = 8.1 Hz, 1H), 1.74 (hept, J = 7.4 Hz, 3H), 1.57 (s, 9H), 1.49 (s, 9H), 1.09 (d, J = 7.4 Hz, 18H).

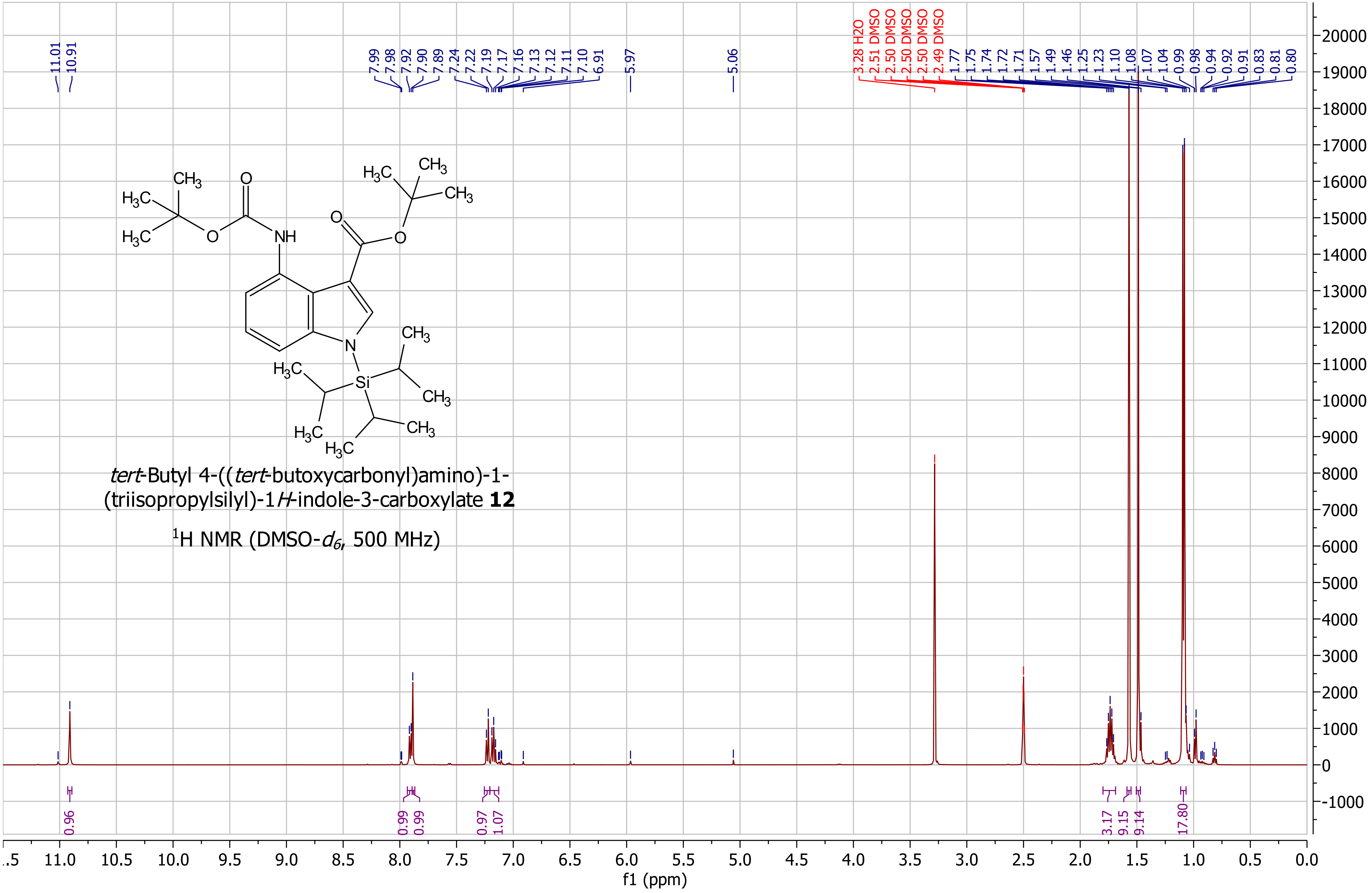

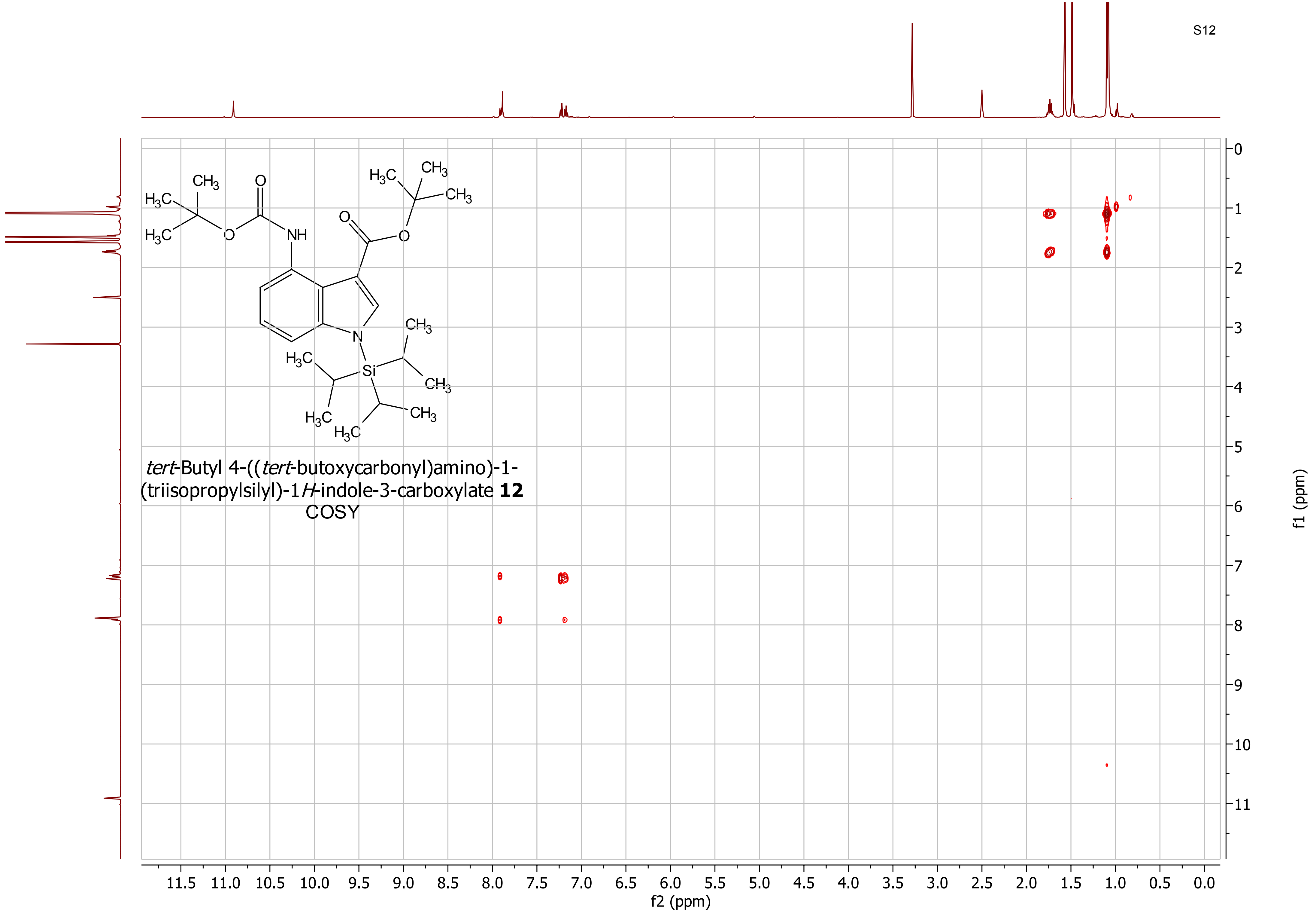
tert-Butyl 4-((tert-butoxycarbonyl)amino)-1-(triisopropylsilyl)-1H-indole-3-carboxylate 12
COSY
f2 (ppm)
f1 (ppm)
11.5 11.0 10.5 10.0 9.5 9.0 8.5 8.0 7.5 7.0 6.5 6.0 5.5 5.0 4.5 4.0 3.5 3.0 2.5 2.0 1.5 1.0 0.5 0.0
0 1 2 3 4 5 6 7 8 9 10 11

^{13}C NMR (126 MHz, DMSO-d_6) δ 165.9, 152.6, 142.1, 139.3, 132.7, 123.8, 117.4, 110.5, 109.9, 108.5, 81.0, 78.9, 28.0, 27.9, 17.8, 17.7, 12.0, 11.8.

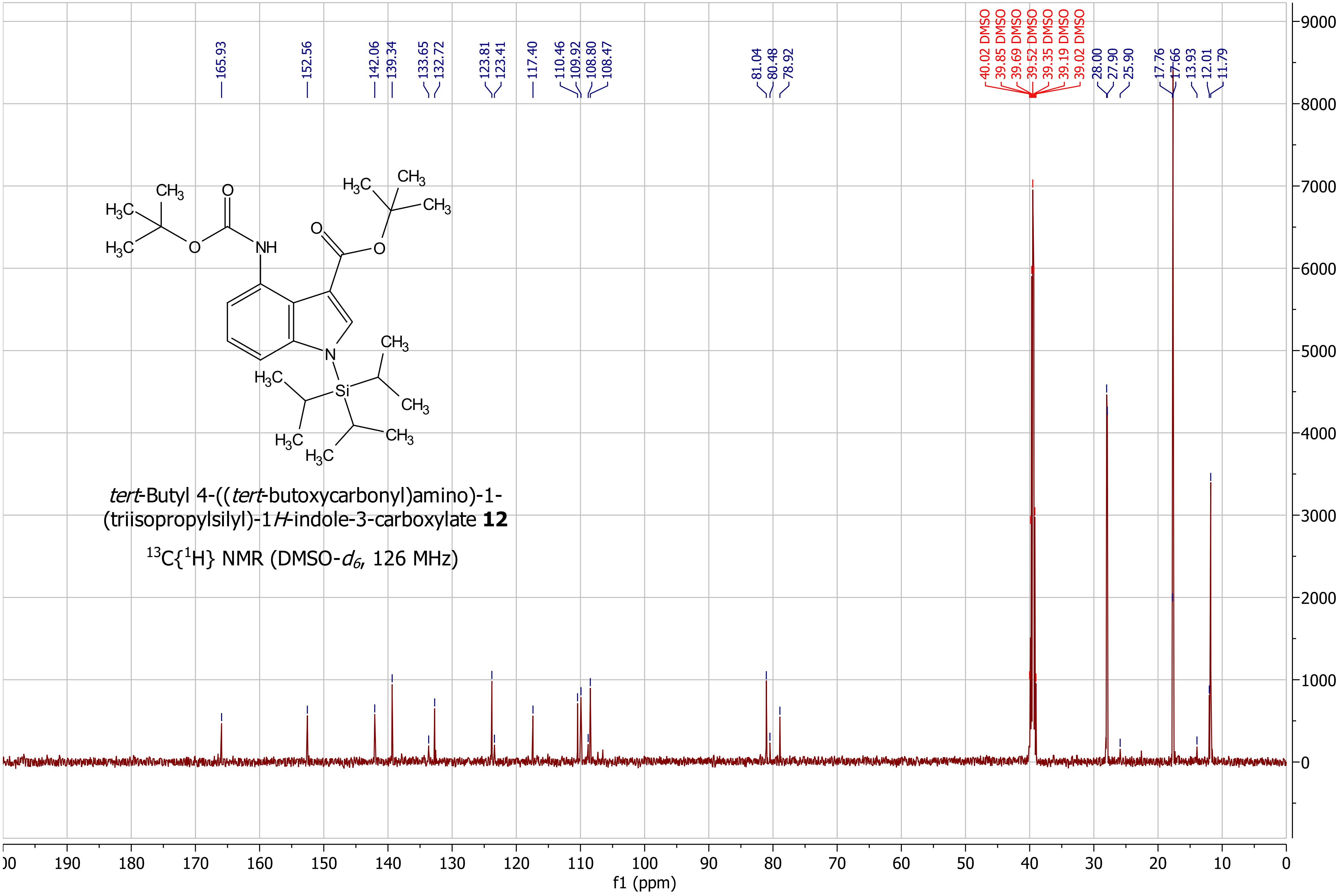

tert-Butyl 4-((*tert*-butoxycarbonyl)amino)-1-(triisopropylsilyl)-1*H*-indole-3-carboxylate **12**

^{13}C{^{1}H} NMR (DMSO-d_6, 126 MHz)

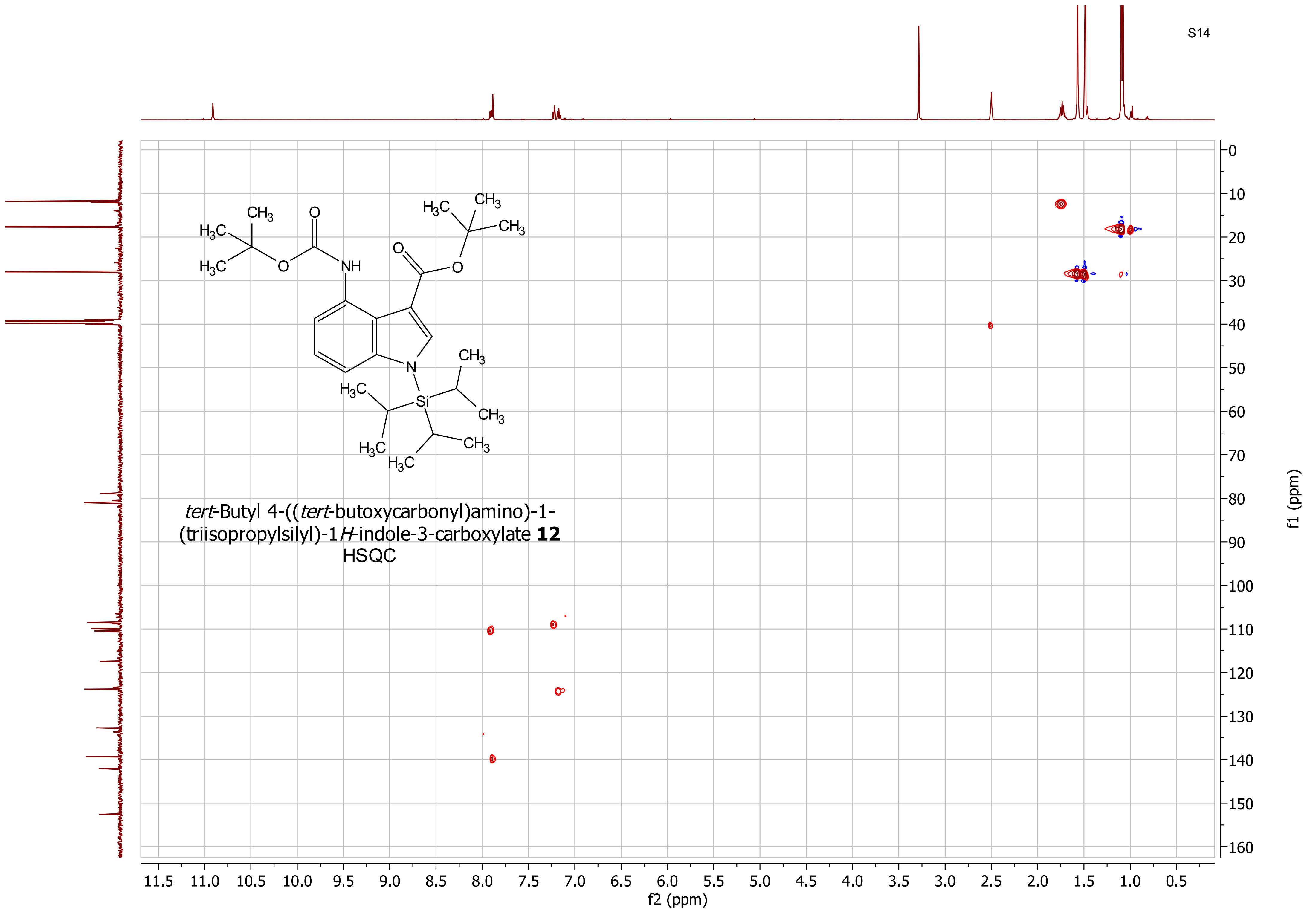

tert-Butyl 4-((*tert*-butoxycarbonyl)amino)-1-(triisopropylsilyl)-1*H*-indole-3-carboxylate **12**
HSQC

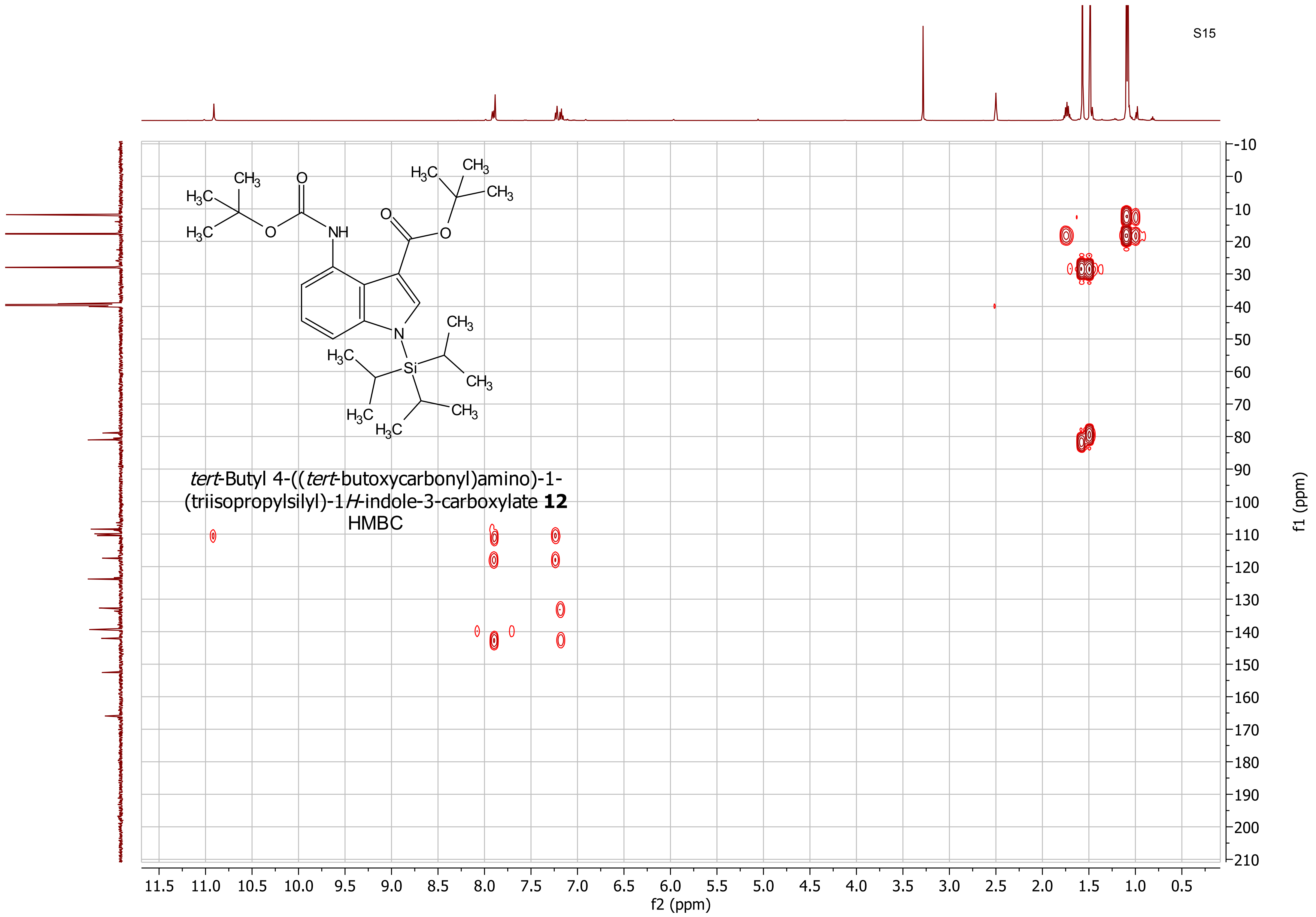
tert-Butyl 4-((tert-butoxycarbonyl)amino)-1-(triisopropylsilyl)-1H-indole-3-carboxylate 12
HMBC
f2 (ppm)
f1 (ppm)

^{1}H NMR (500 MHz, DMSO-d_6) δ 8.10 (d, J = 8.0 Hz, 1H), 8.04 (d, J = 8.2 Hz, 1H), 7.78 (d, J = 3.1 Hz, 1H), 7.36 (t, J = 8.1 Hz, 1H), 7.26 (d, J = 3.1 Hz, 1H), 1.81 (hept, J = 7.5 Hz, 3H), 1.09 (d, J = 7.5 Hz, 18H).

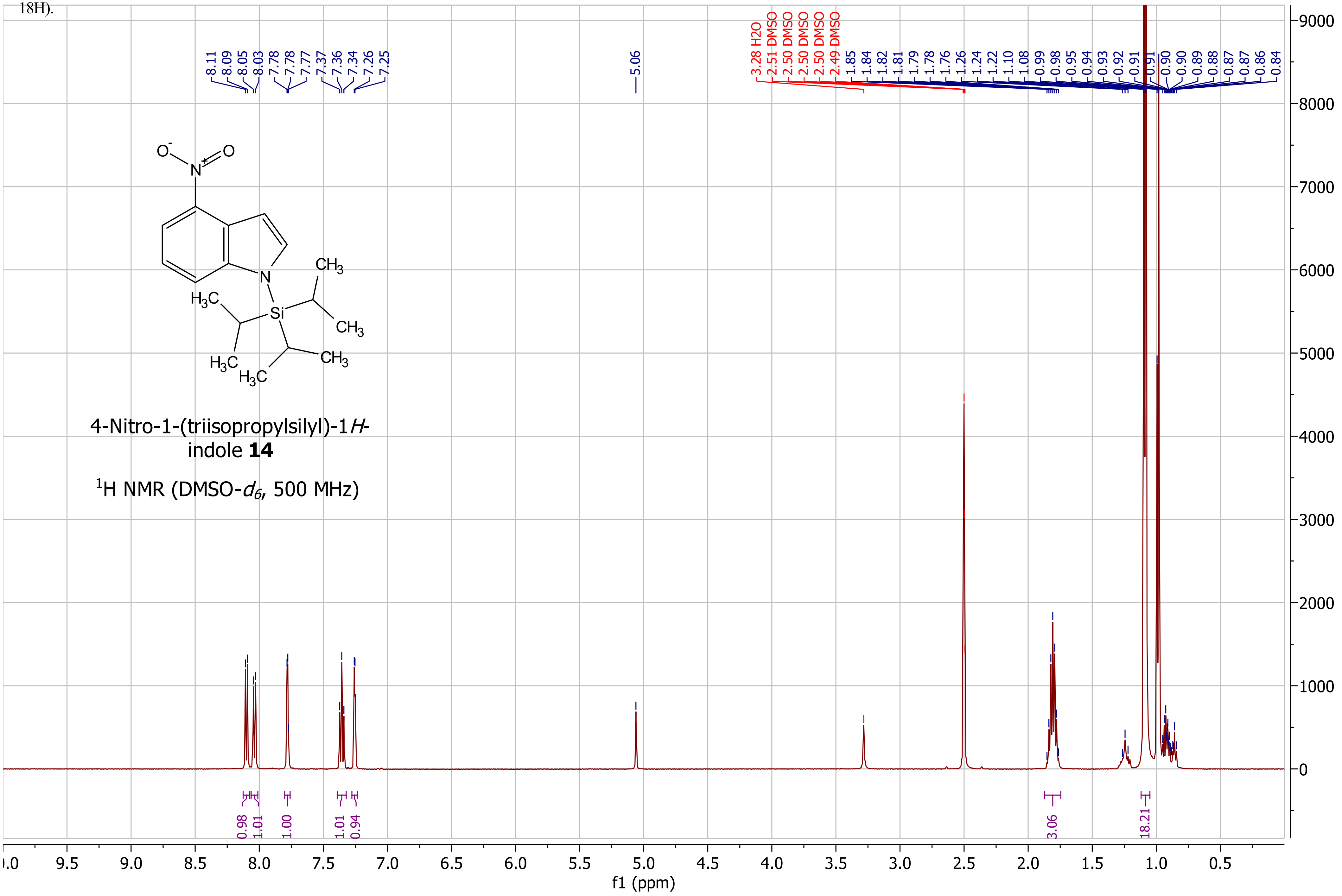

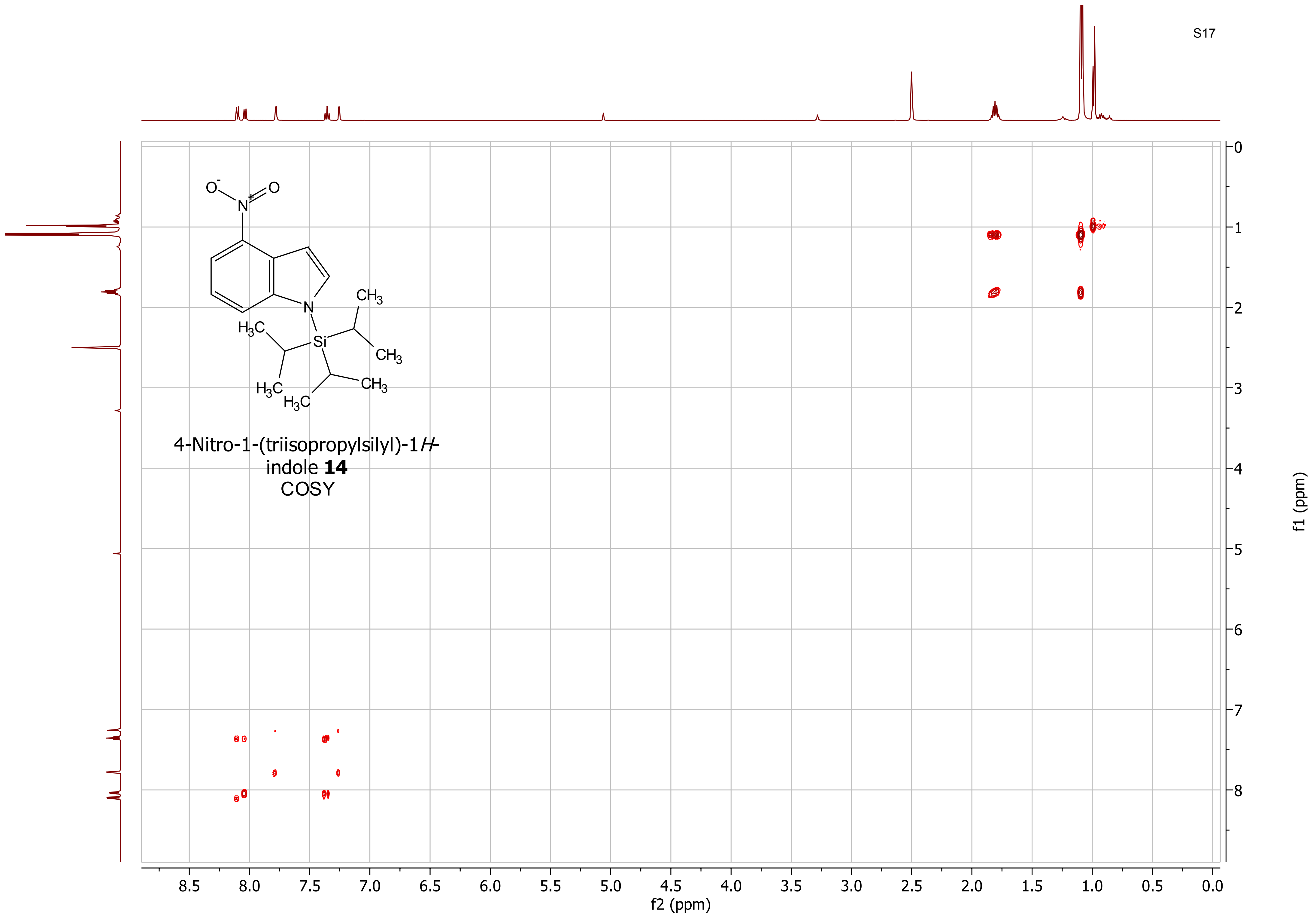

4-Nitro-1-(triisopropylsilyl)-1*H*-indole **14**
COSY

^{13}C NMR (126 MHz, DMSO-d_6) δ 142.5, 139.5, 137.0, 124.8, 121.0, 120.8, 117.4, 104.4, 17.7, 11.8.

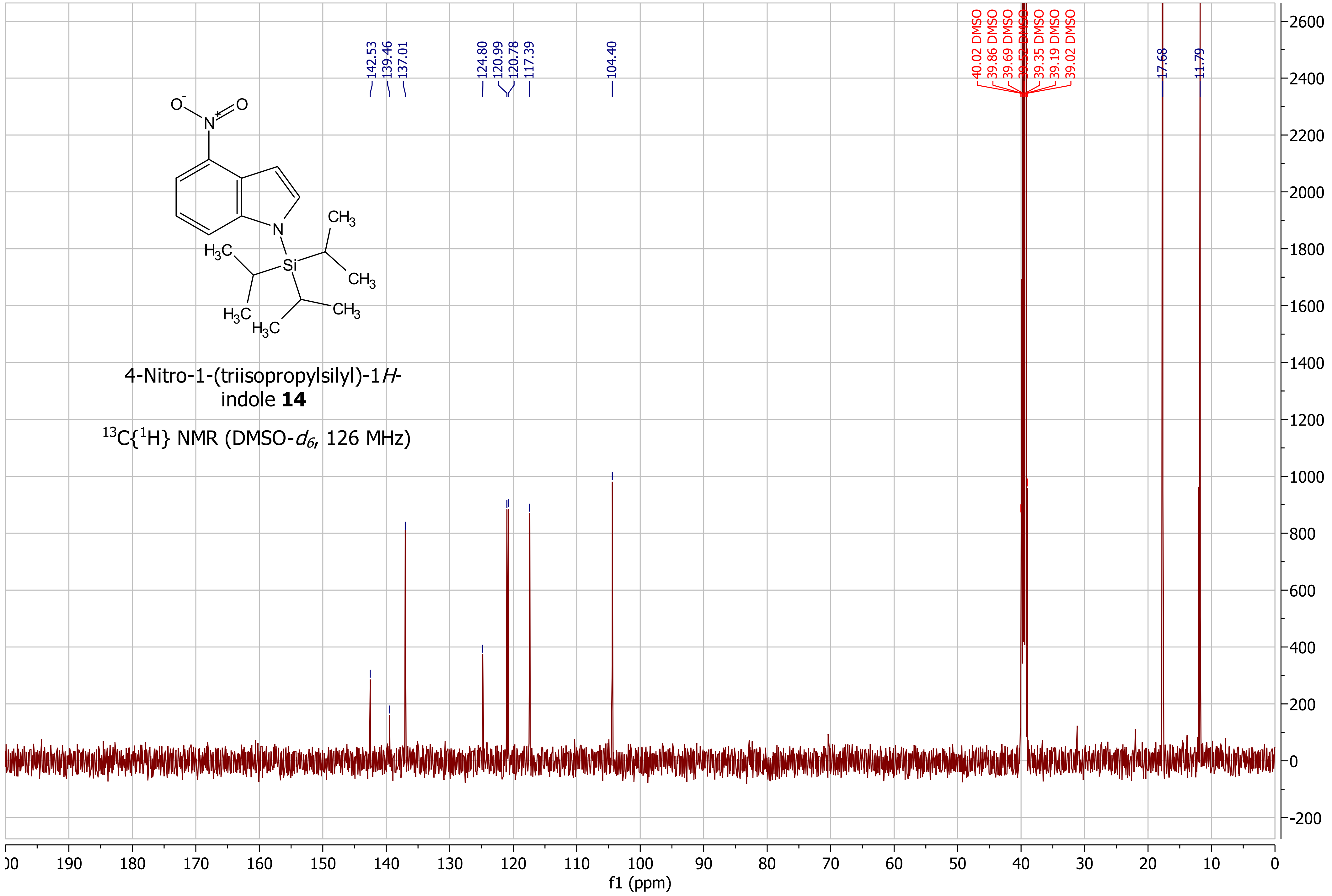

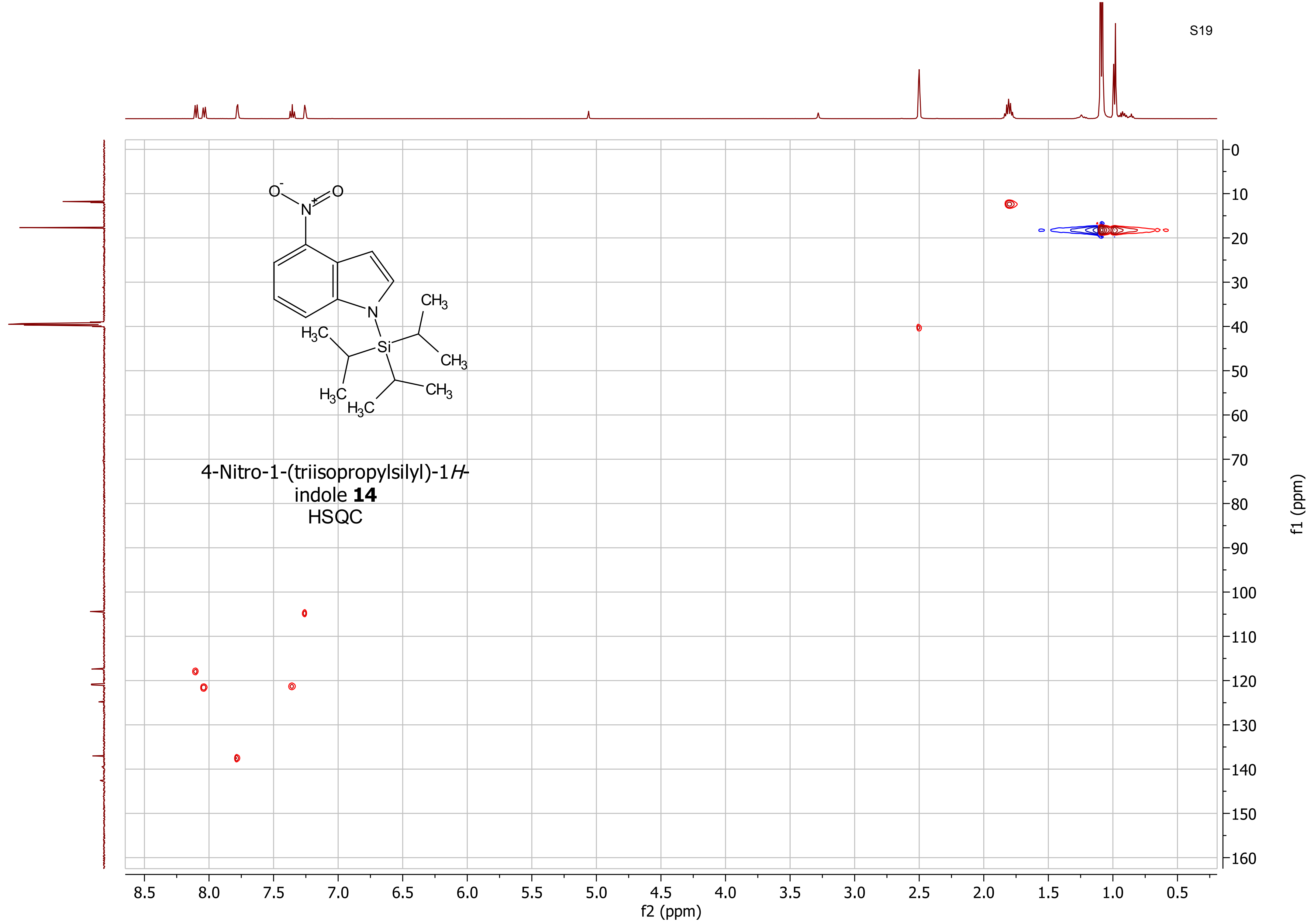
4-Nitro-1-(triisopropylsilyl)-1H-
indole 14
HSQC
f2 (ppm)
f1 (ppm)

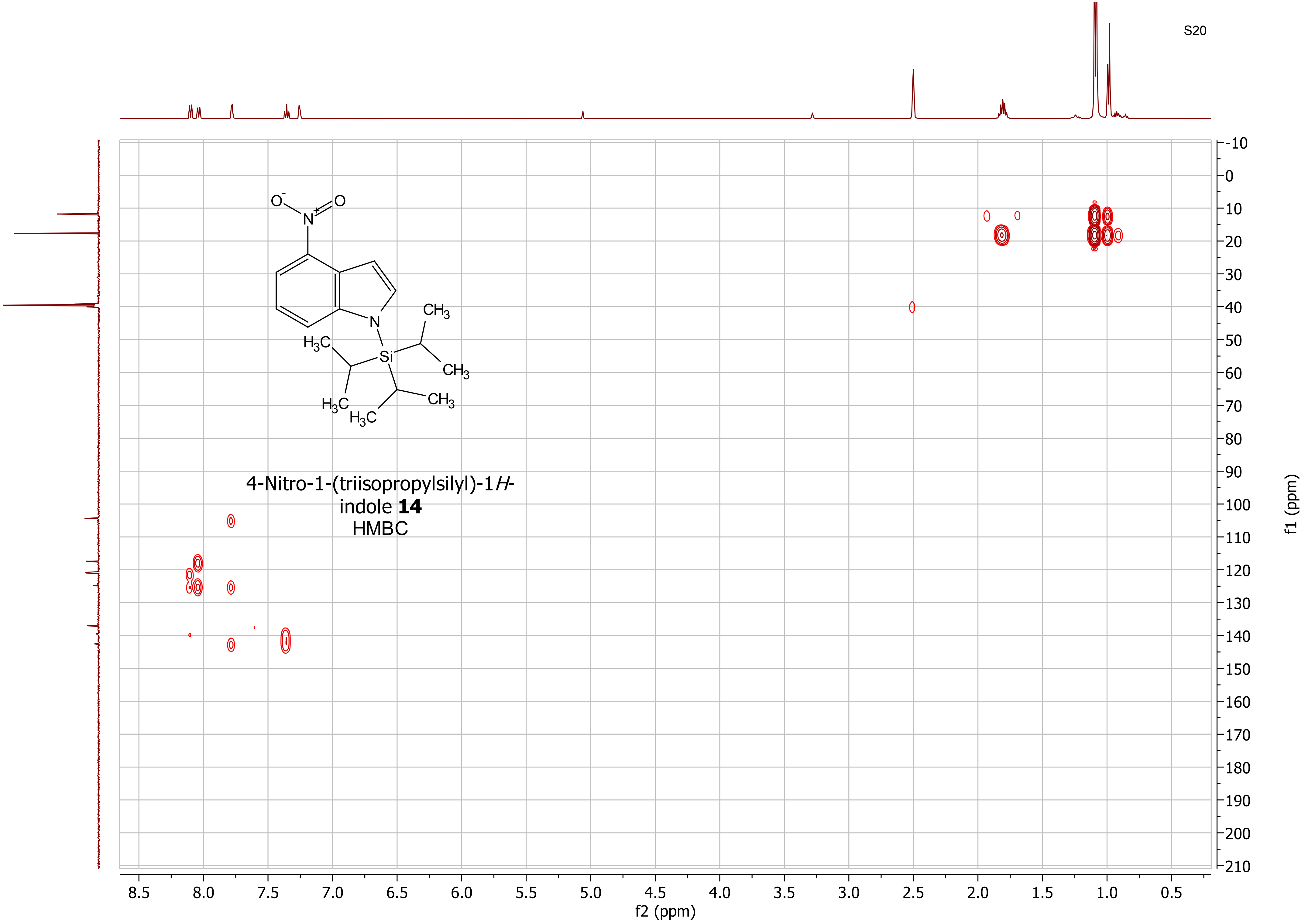

4-Nitro-1-(triisopropylsilyl)-1*H*-indole **14**
HMBC
f2 (ppm)
f1 (ppm)

^{1}H NMR (500 MHz, DMSO-d_6) δ 10.09 (s, 3H), 7.53 (d, J = 8.2 Hz, 1H), 7.50 – 7.46 (m, 1H), 7.21 – 7.14 (m, 1H), 7.07 – 7.04 (m, 1H), 6.84 (d, J = 3.3 Hz, 1H), 1.76 (hept, J = 7.5 Hz, 3H), 1.08 (d, J = 7.5 Hz, 18H).

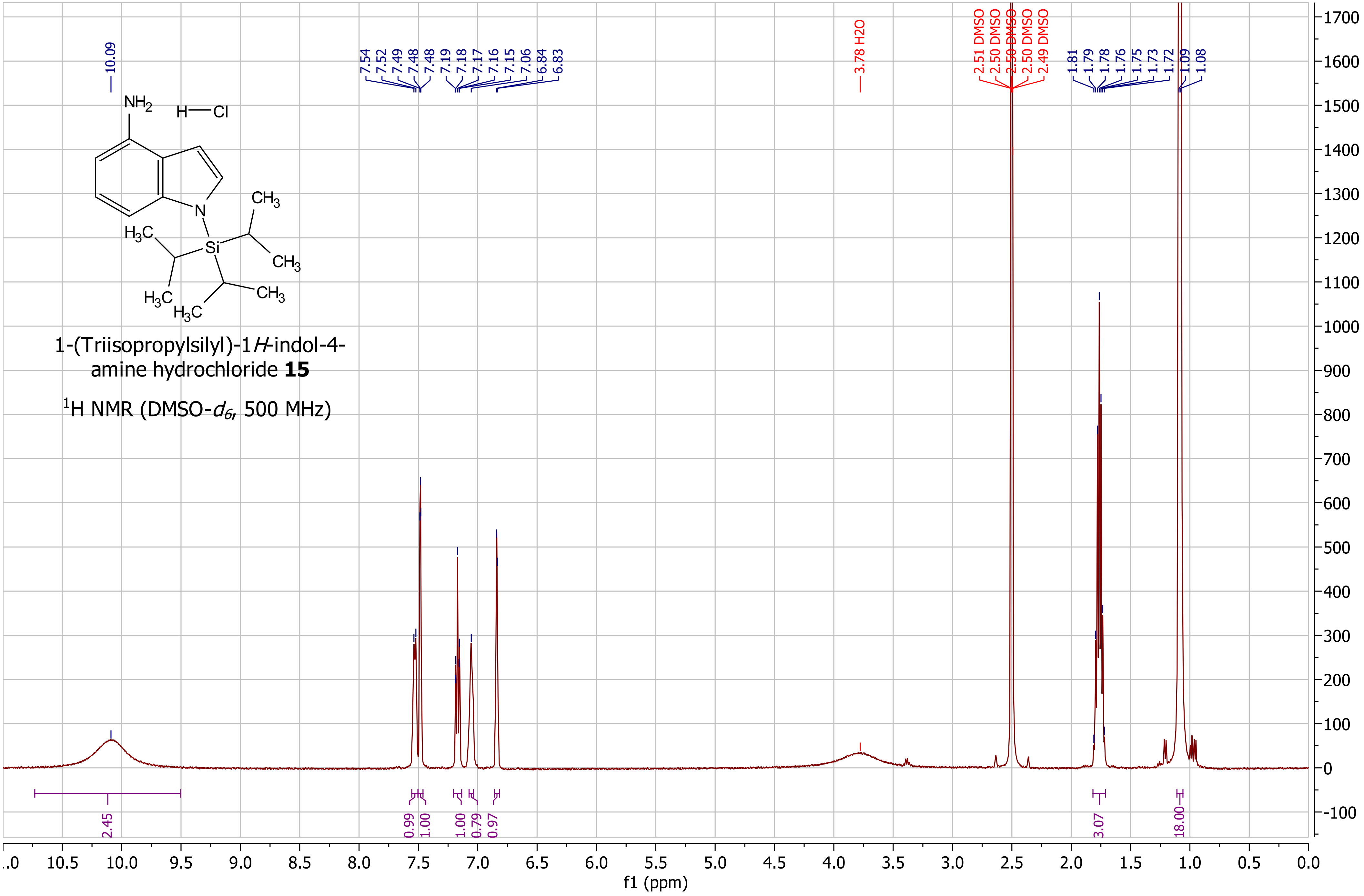

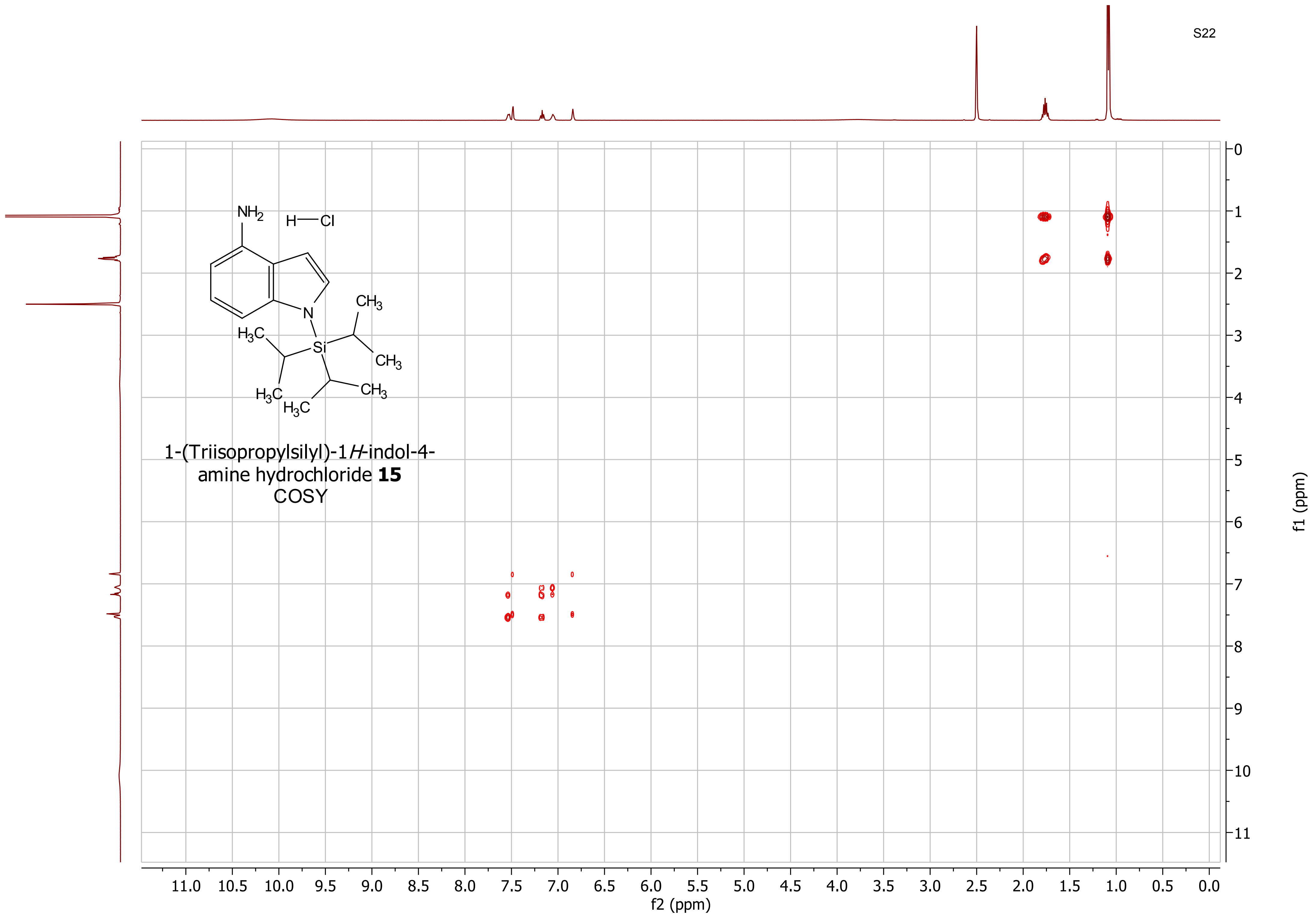
NH2
H—Cl
N
Si
CH3
H3C
1-(Triisopropylsilyl)-1H-indol-4-amine hydrochloride 15
COSY
f2 (ppm)
f1 (ppm)

^{13}C NMR (126 MHz, DMSO-d_6) δ 141.3, 132.3, 121.6, 113.0, 113.0, 101.9, 17.7, 11.8.

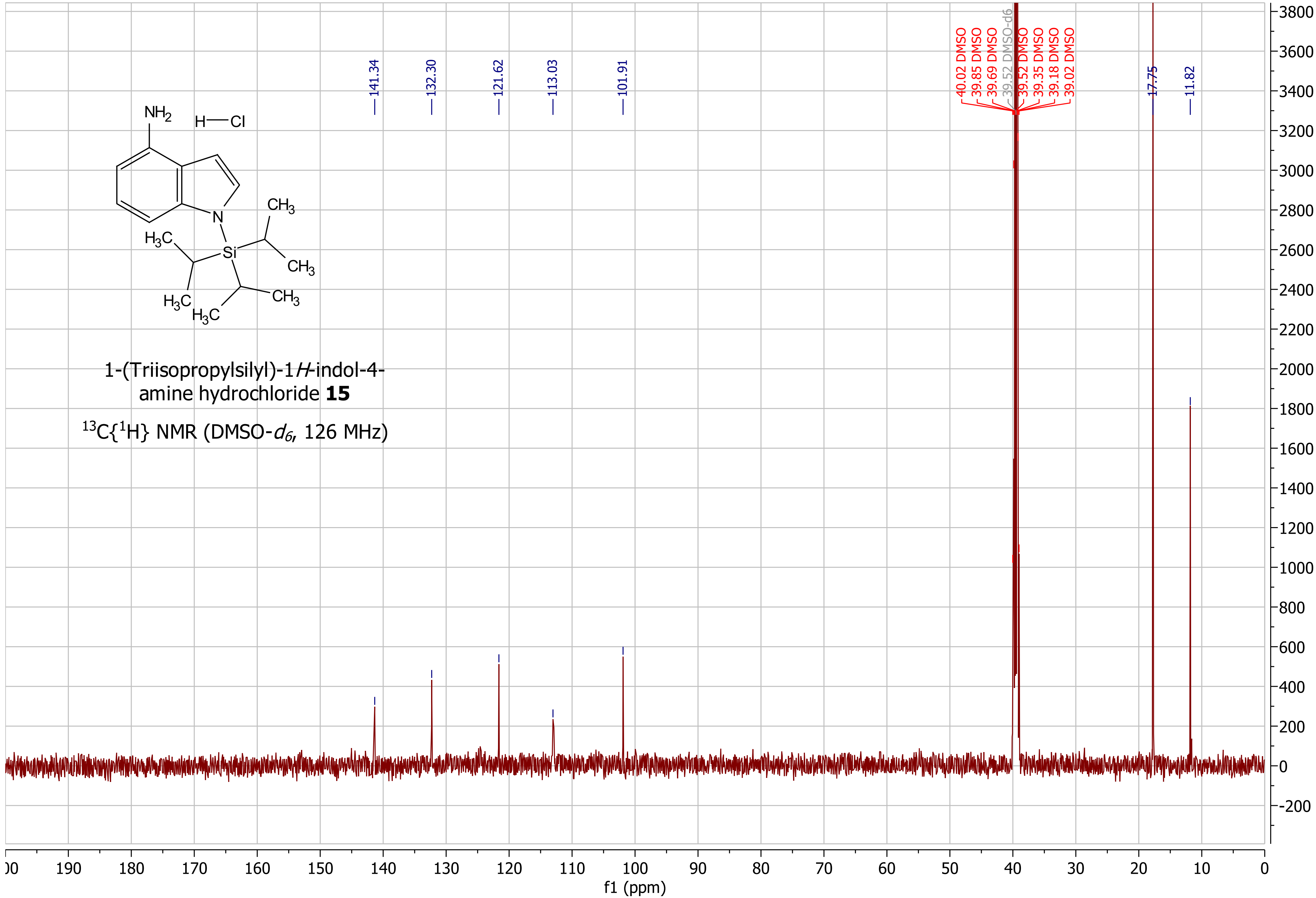

1-(Triisopropylsilyl)-1*H*-indol-4-amine hydrochloride **15**

^{13}C{^{1}H} NMR (DMSO-d_6, 126 MHz)

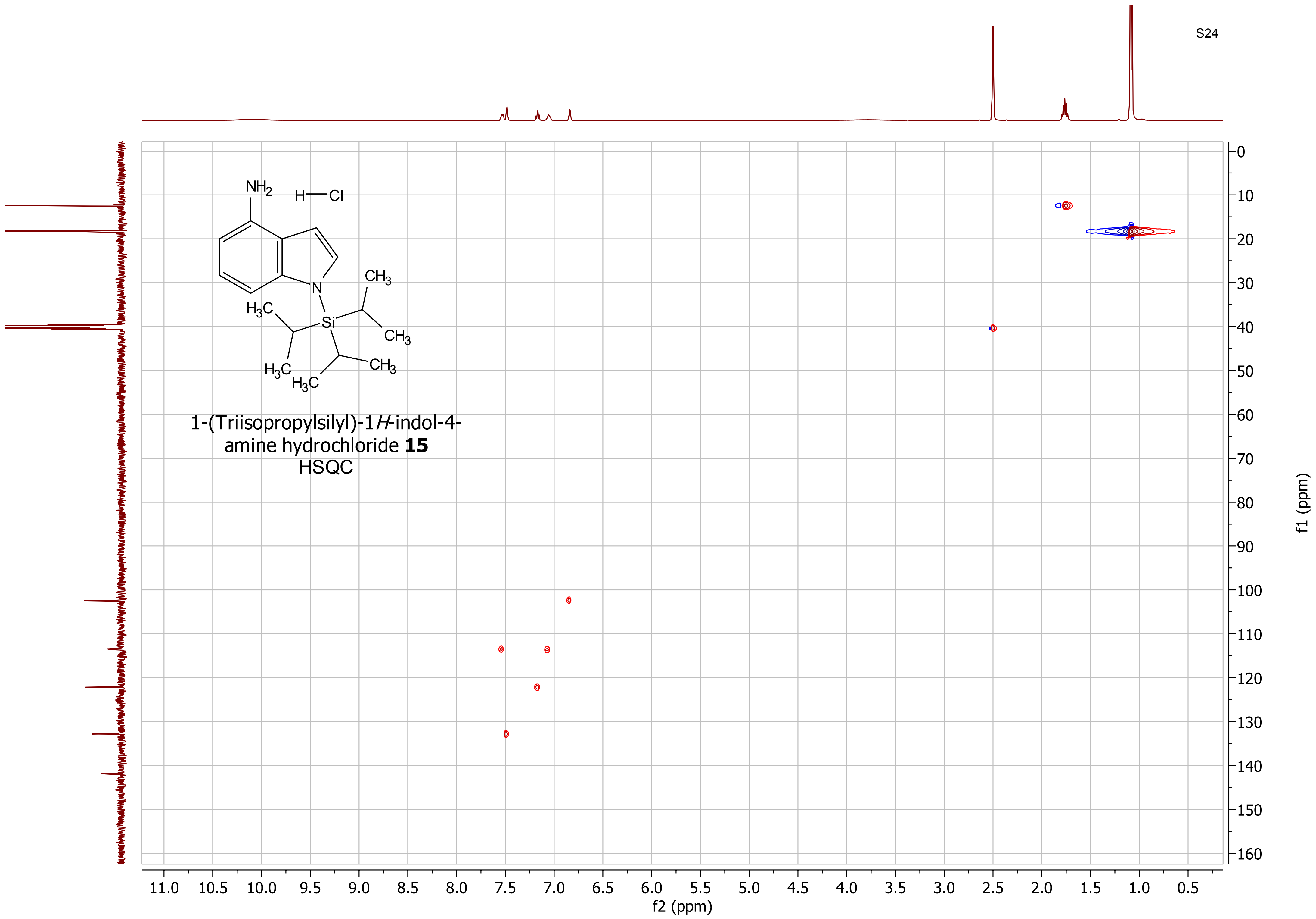

1-(Triisopropylsilyl)-1*H*-indol-4-amine hydrochloride **15**
HSQC

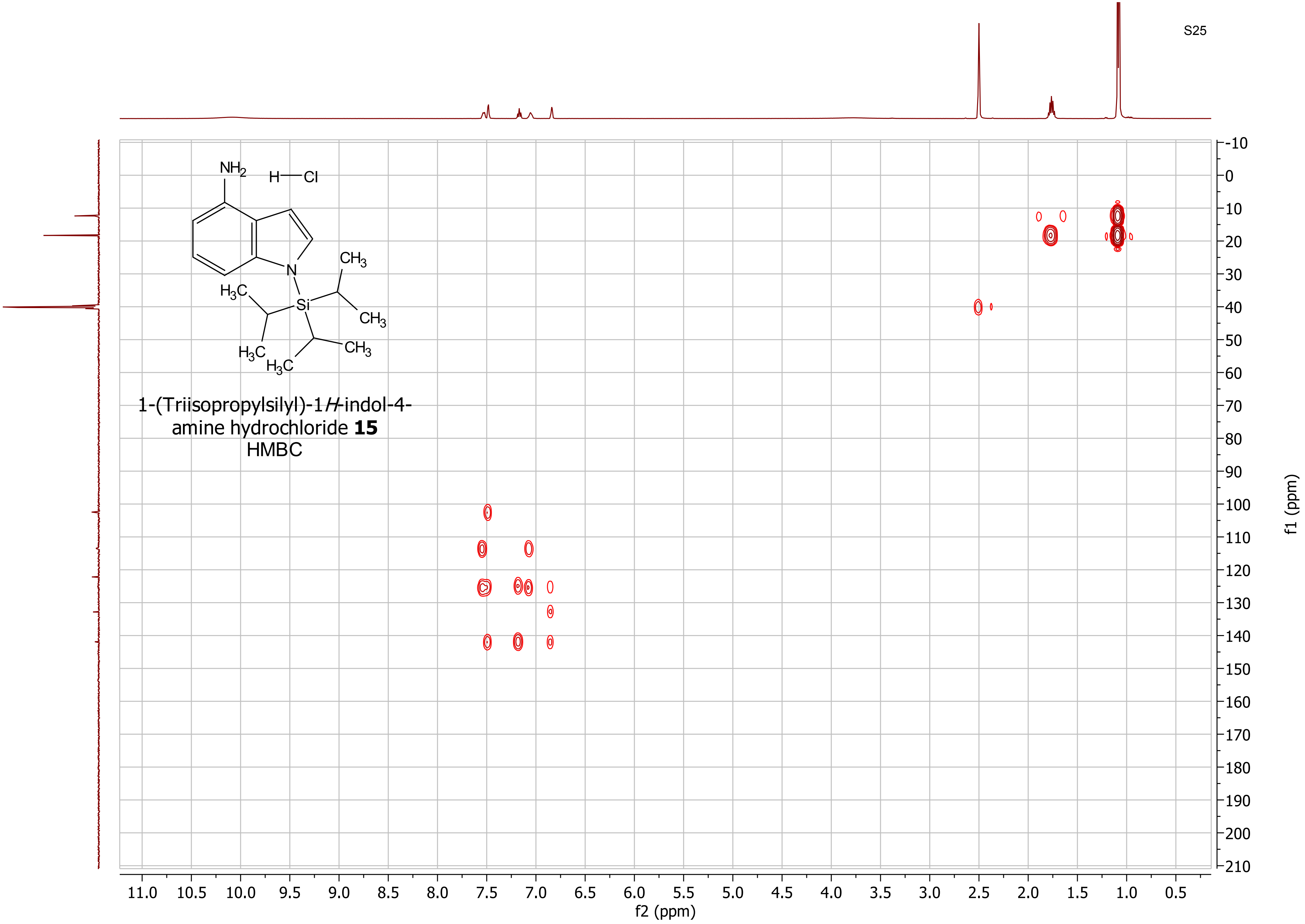

1-(Triisopropylsilyl)-1*H*-indol-4-amine hydrochloride **15**
HMBC

^{1}H NMR (500 MHz, DMSO-d_6) δ 8.96 (s, 1H), 7.35 (d, J = 7.7 Hz, 1H), 7.26 (d, J = 3.1 Hz, 1H), 7.21 (d, J = 8.3 Hz, 1H), 7.01 (t, J = 7.9 Hz, 1H), 6.90 (d, J = 3.2 Hz, 1H), 1.72 (hept, J = 7.5 Hz, 3H), 1.50 (s, 9H), 1.08 (d, J = 7.5 Hz, 18H).

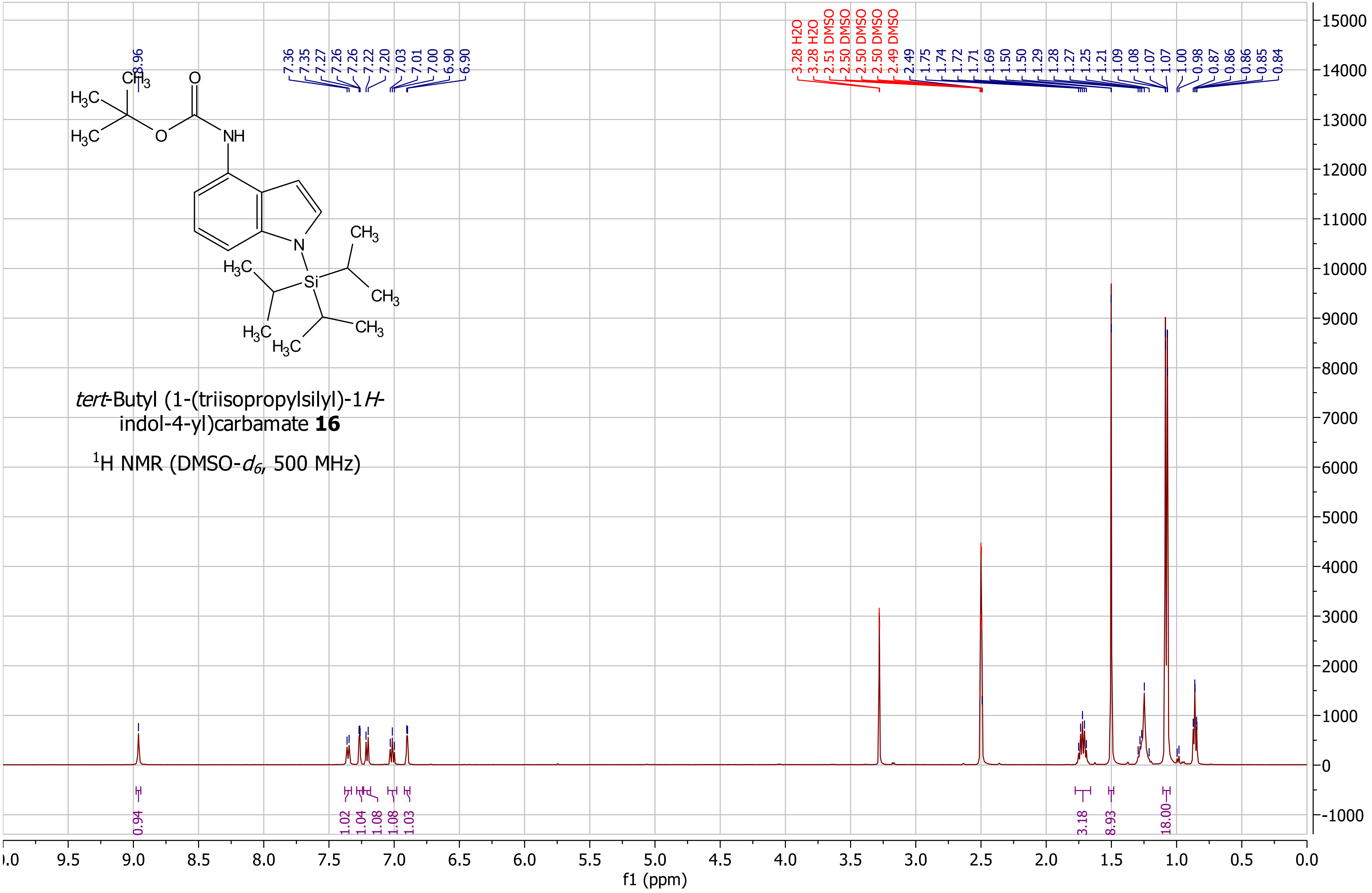

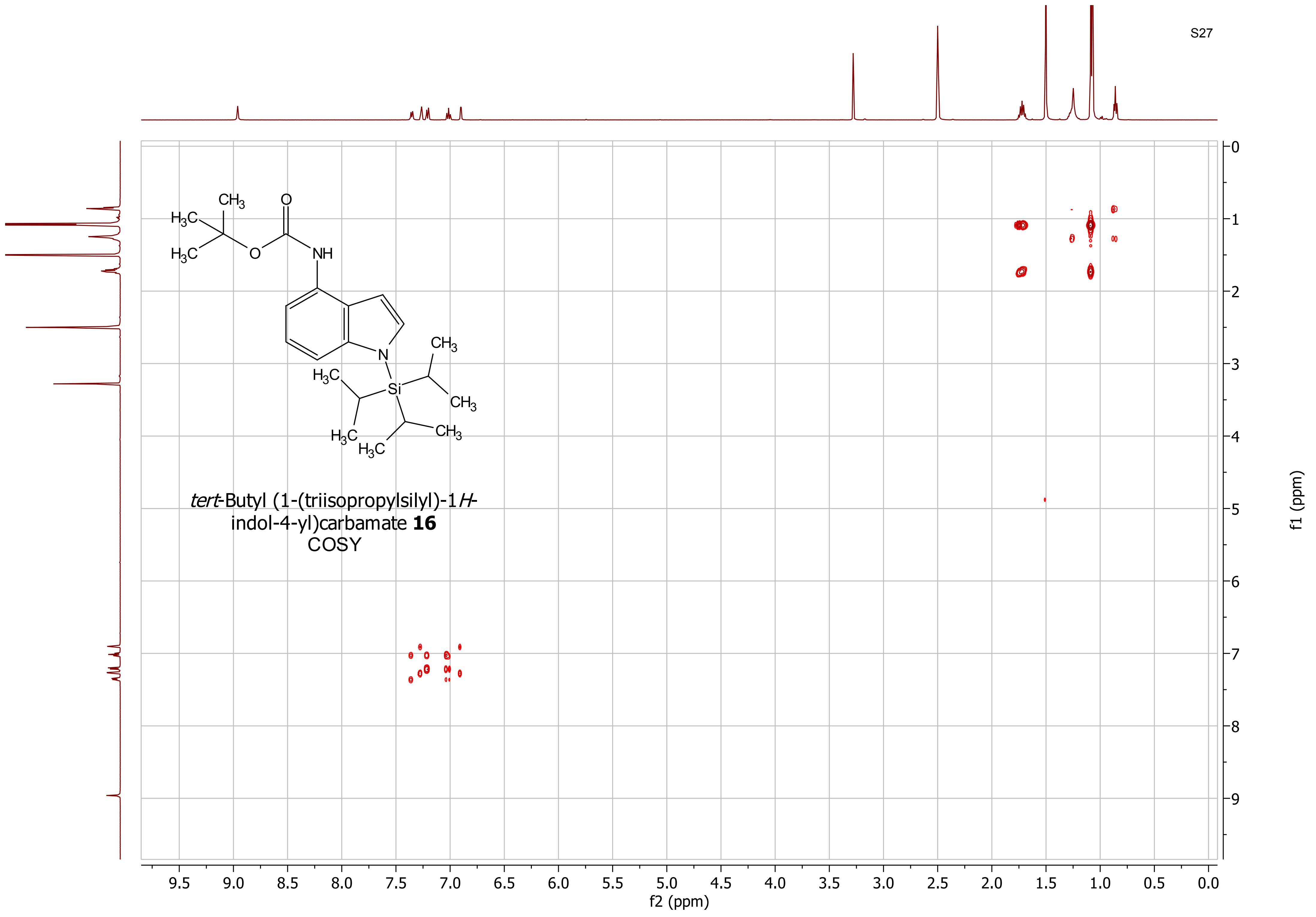
tert-Butyl (1-(triisopropylsilyl)-1*H*-indol-4-yl)carbamate **16**
COSY
f2 (ppm)
f1 (ppm)

^{13}C NMR (126 MHz, DMSO-d_6) δ 153.2, 141.0, 131.0, 130.0, 121.4, 110.7, 108.8, 102.9, 78.7, 28.1, 17.8, 11.9.

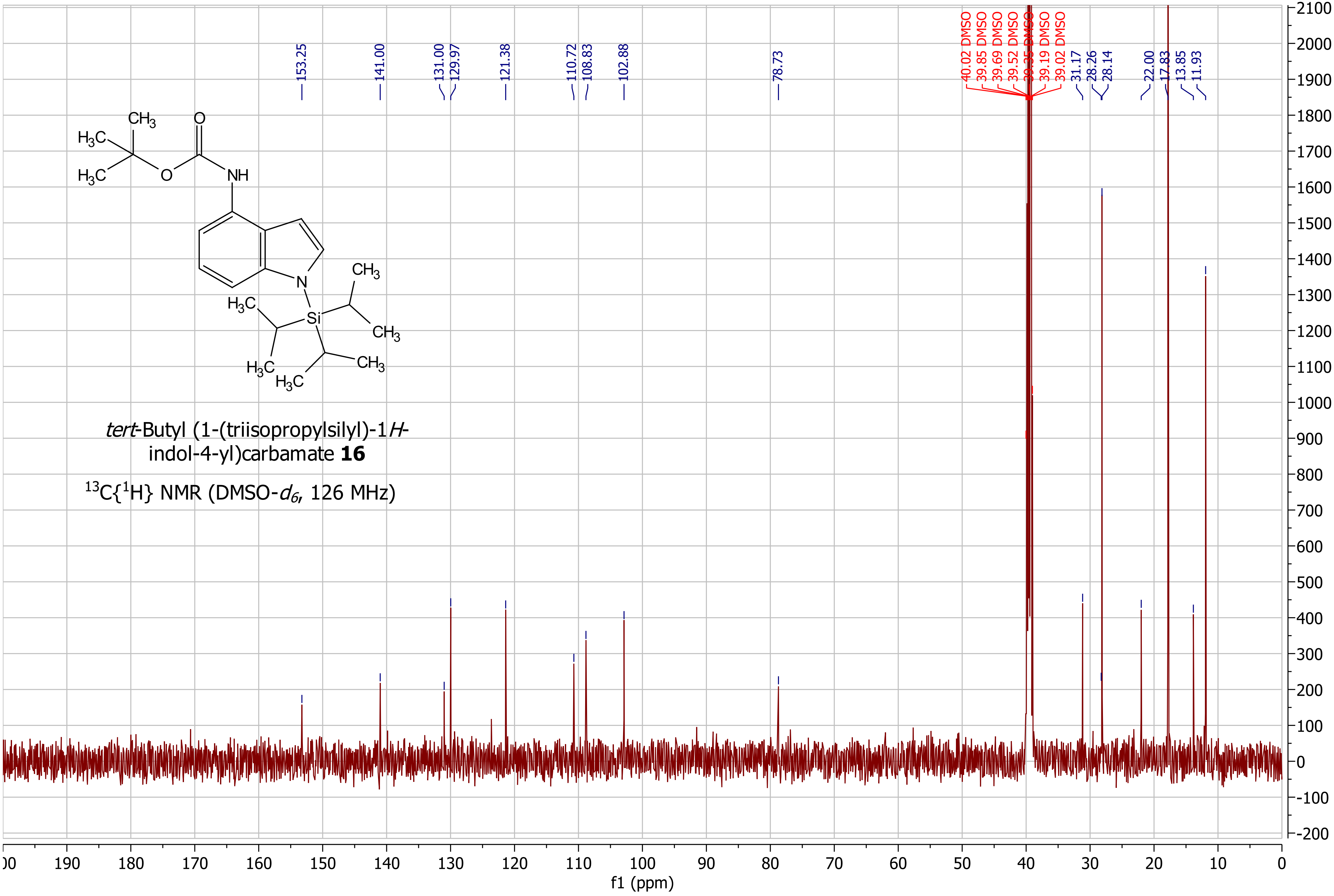

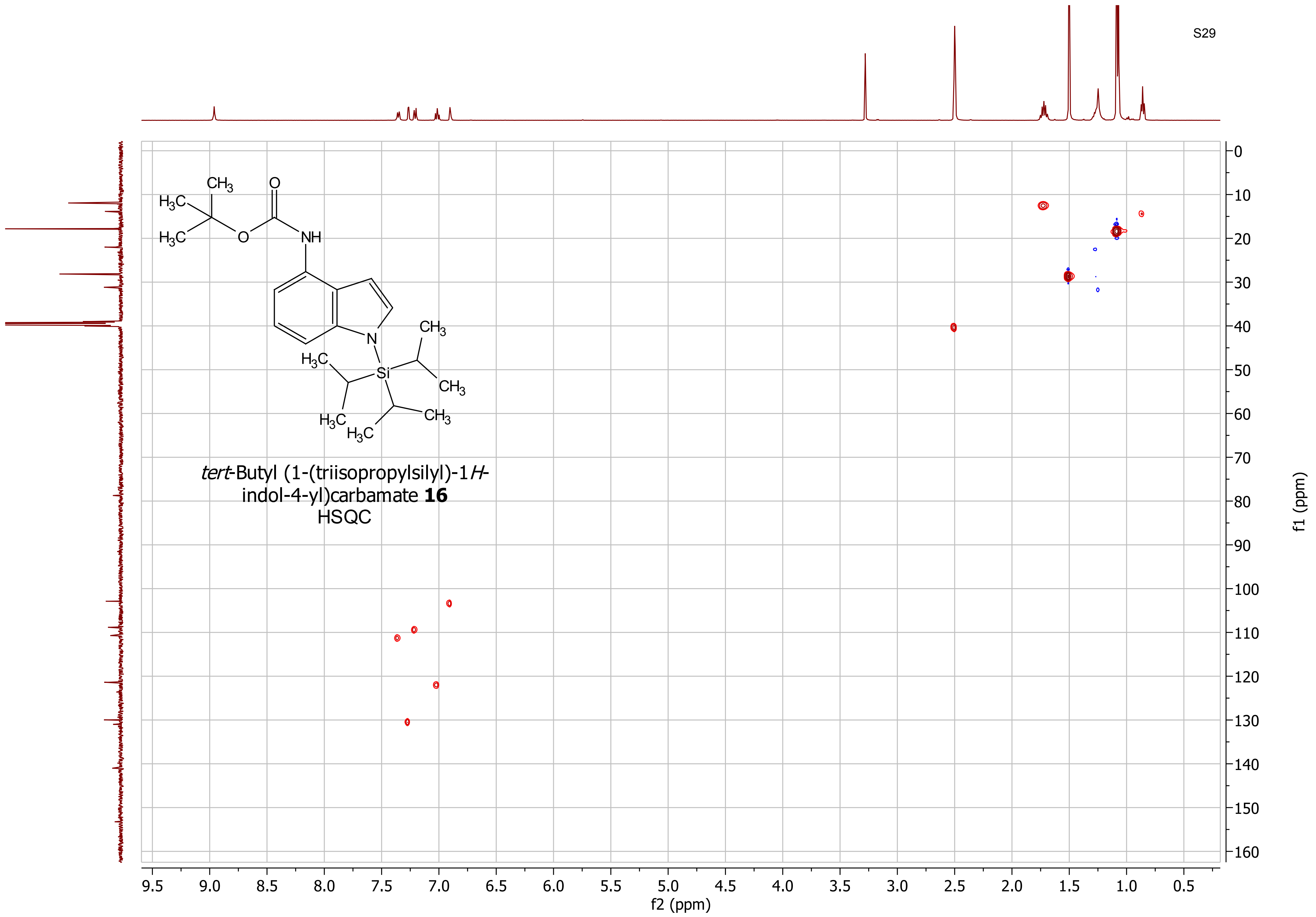

tert-Butyl (1-(triisopropylsilyl)-1*H*-indol-4-yl)carbamate **16**
HSQC

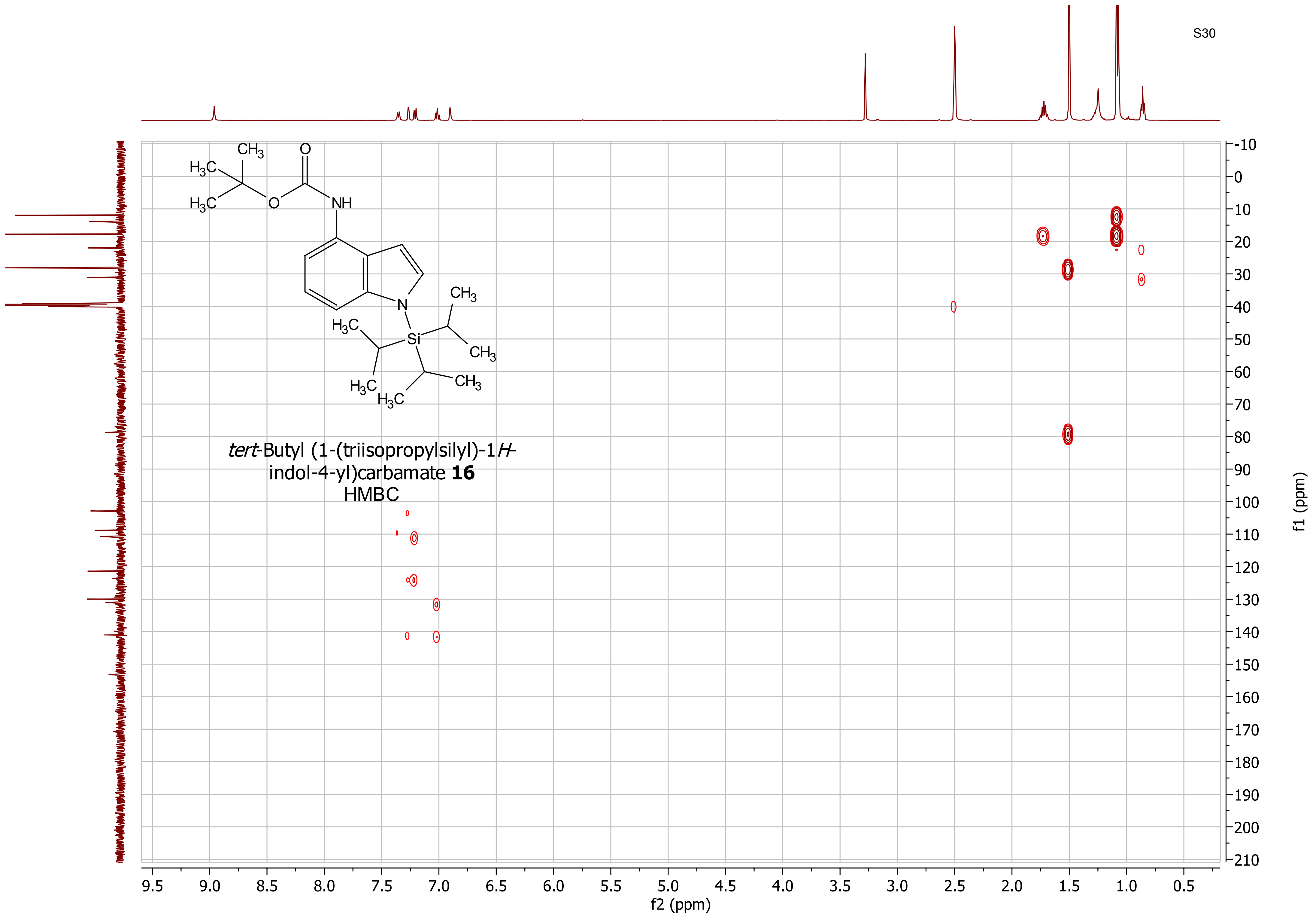

tert-Butyl (1-(triisopropylsilyl)-1*H*-indol-4-yl)carbamate **16**
HMBC
f2 (ppm)
f1 (ppm)

^{1}H NMR (500 MHz, DMSO-d_6) δ 11.99 (s, 1H), 11.01 (s, 1H), 7.99 (d, J = 1.6 Hz, 1H), 7.88 (d, J = 7.2 Hz, 1H), 7.15 – 7.08 (m, 2H), 1.57 (s, 9H), 1.49 (s, 9H).

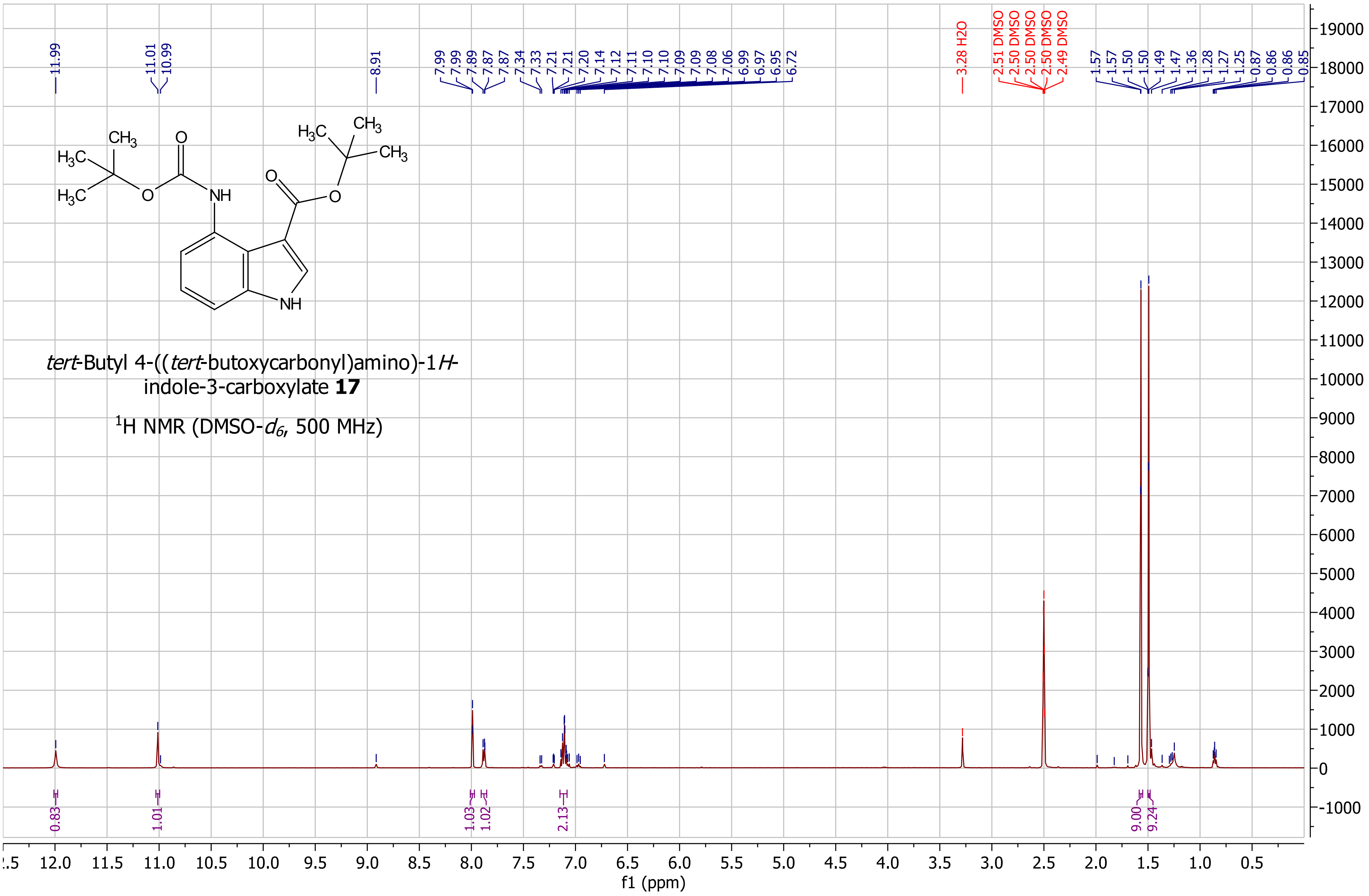

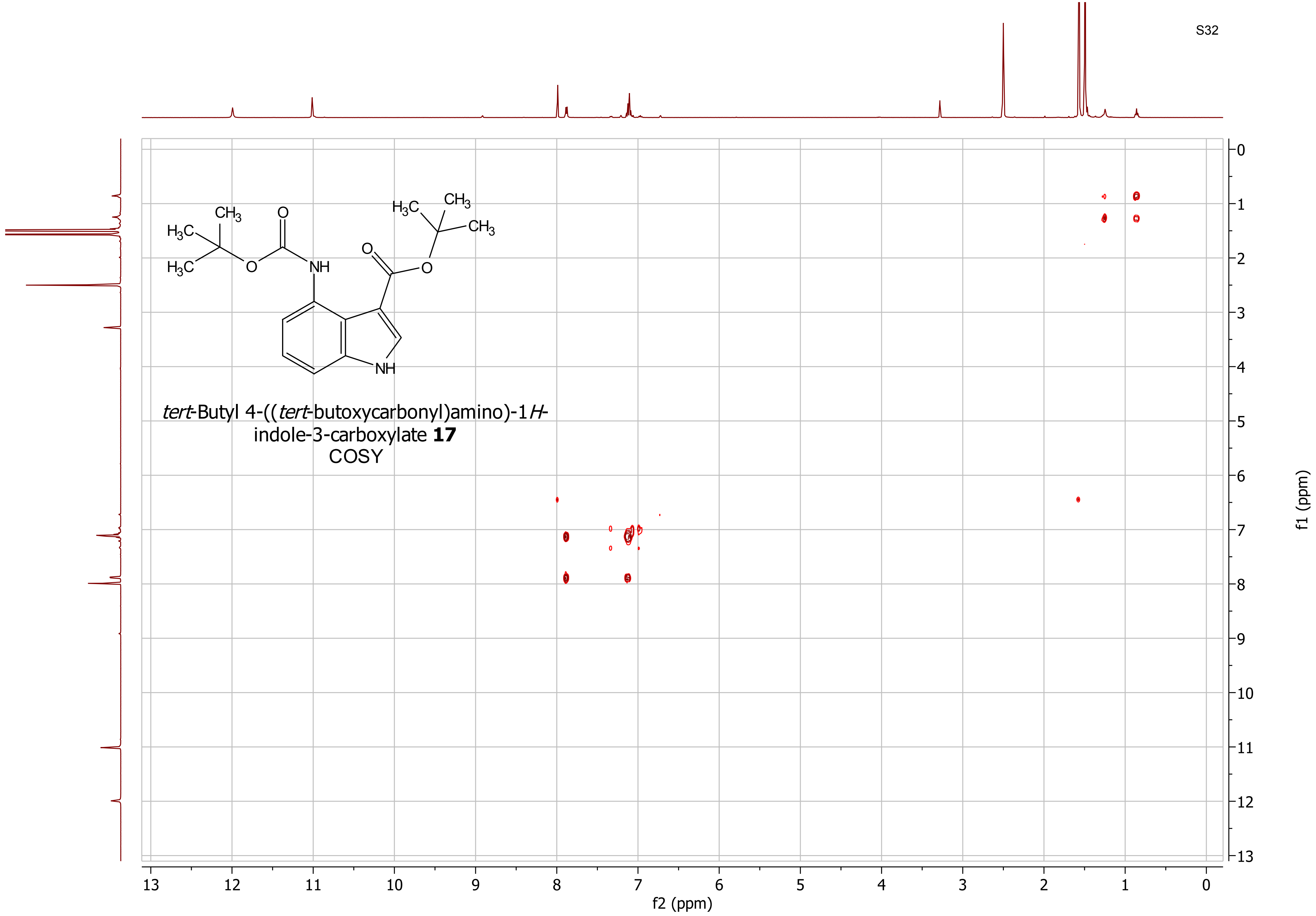
tert-Butyl 4-((*tert*-butoxycarbonyl)amino)-1*H*-indole-3-carboxylate **17**
COSY
f2 (ppm)
f1 (ppm)

^{13}C NMR (126 MHz, DMSO-d_6) δ 166.5, 152.5, 137.9, 133.7, 132.6, 123.4, 115.1, 108.8, 107.3, 106.5, 80.5, 78.8, 28.0.

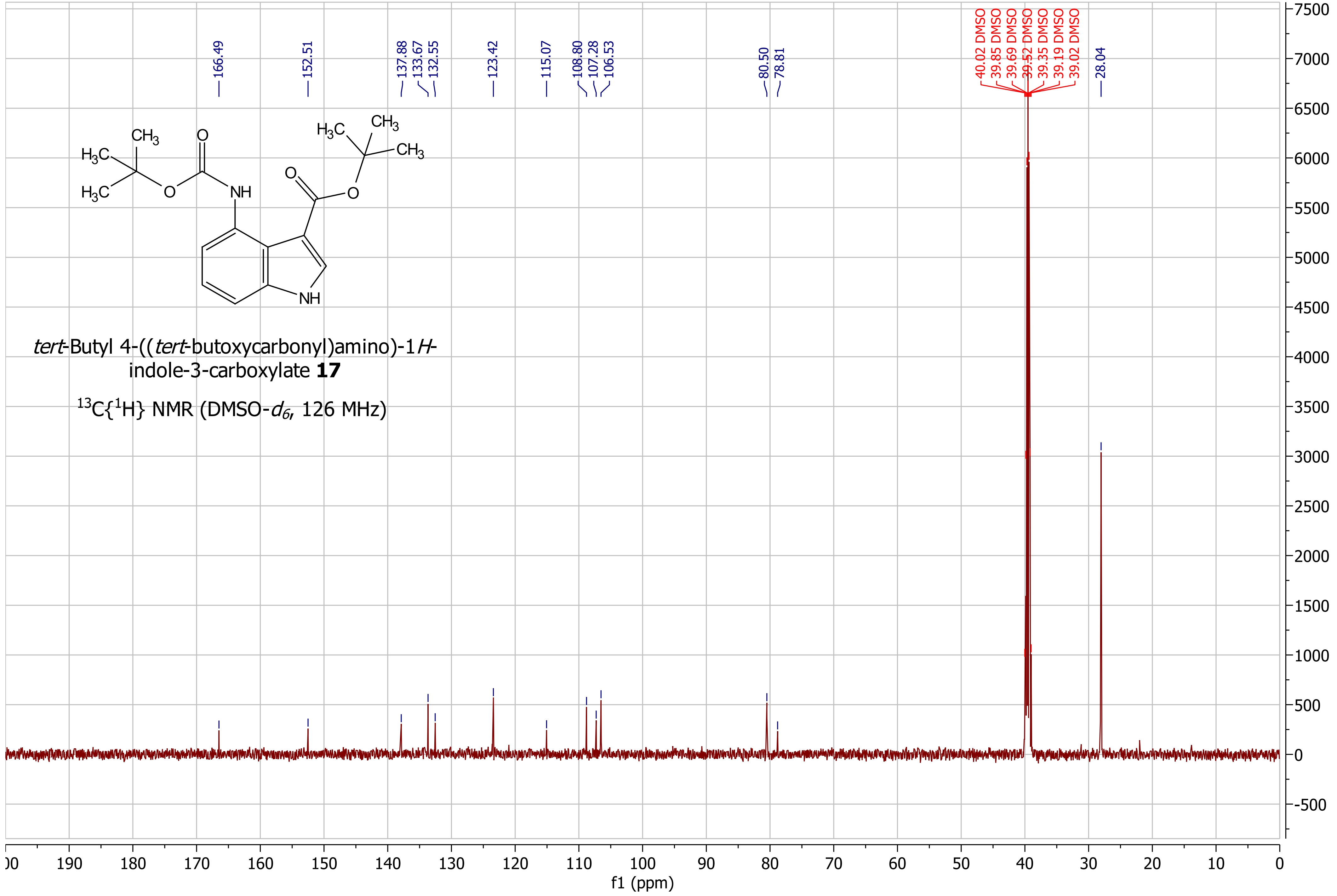

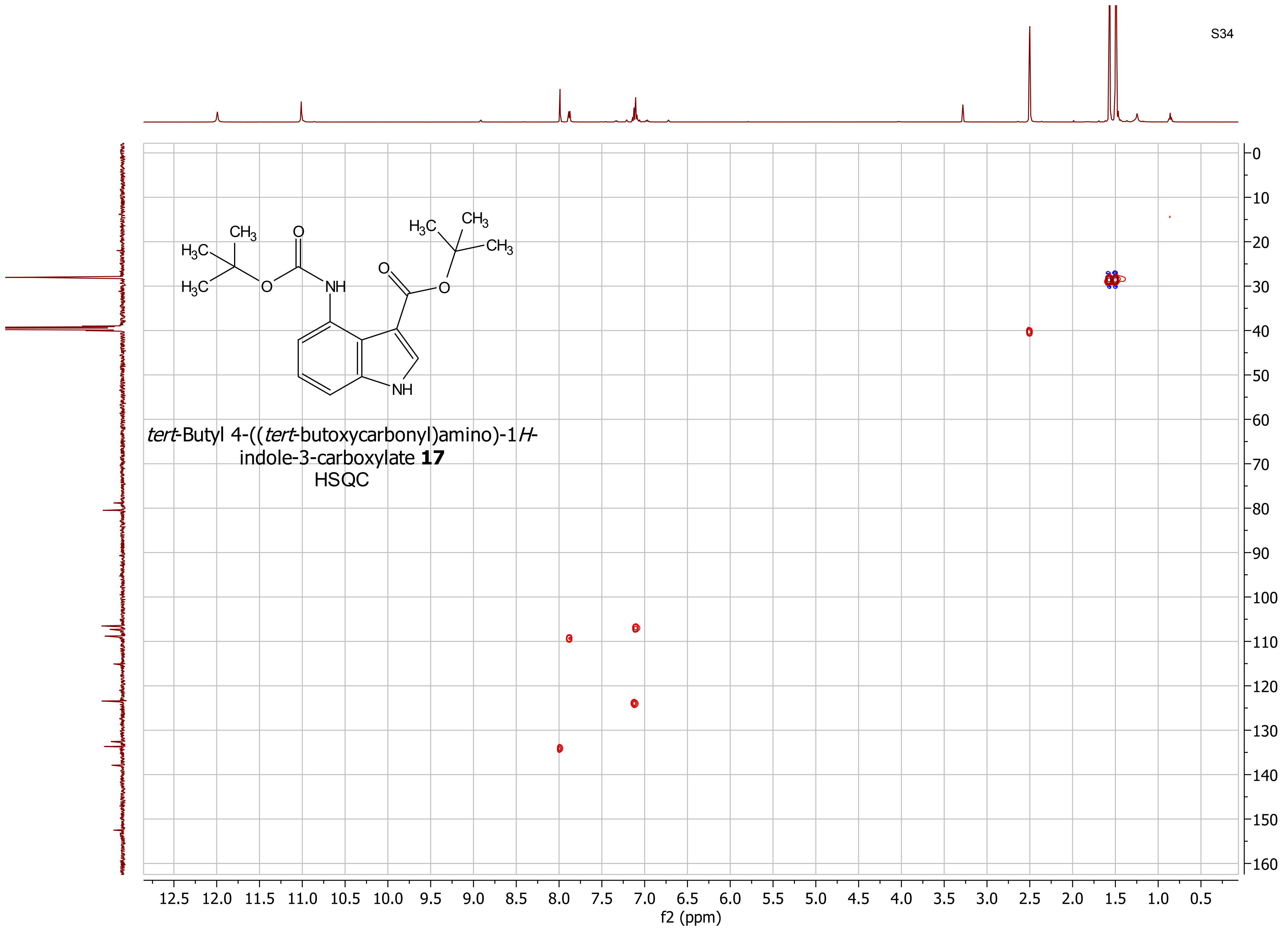

tert-Butyl 4-((*tert*-butoxycarbonyl)amino)-1*H*-indole-3-carboxylate **17**
HSQC

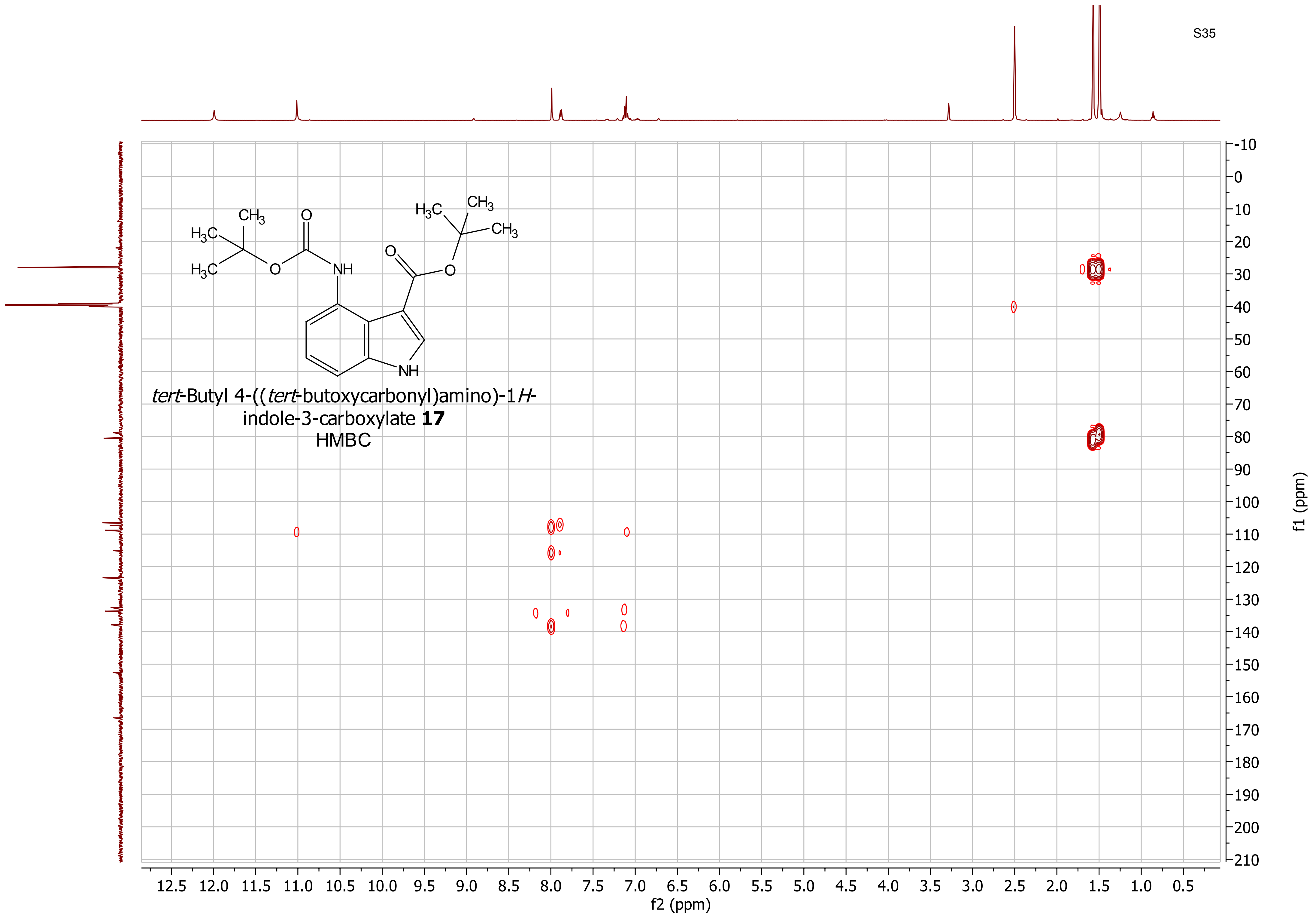

tert-Butyl 4-((*tert*-butoxycarbonyl)amino)-1*H*-indole-3-carboxylate **17**

HMBC

^{1}H NMR (500 MHz, DMSO-d_6) δ 12.60 (s, 1H), 12.02 (s, 1H), 11.86 (s, 1H), 8.08 (d, J = 3.1 Hz, 1H), 7.91 (d, J = 7.5 Hz, 1H), 7.45 – 7.36 (m, 4H), 7.33 (t, J = 7.2 Hz, 1H), 7.20 – 7.10 (m, 2H), 5.19 (s, 2H).

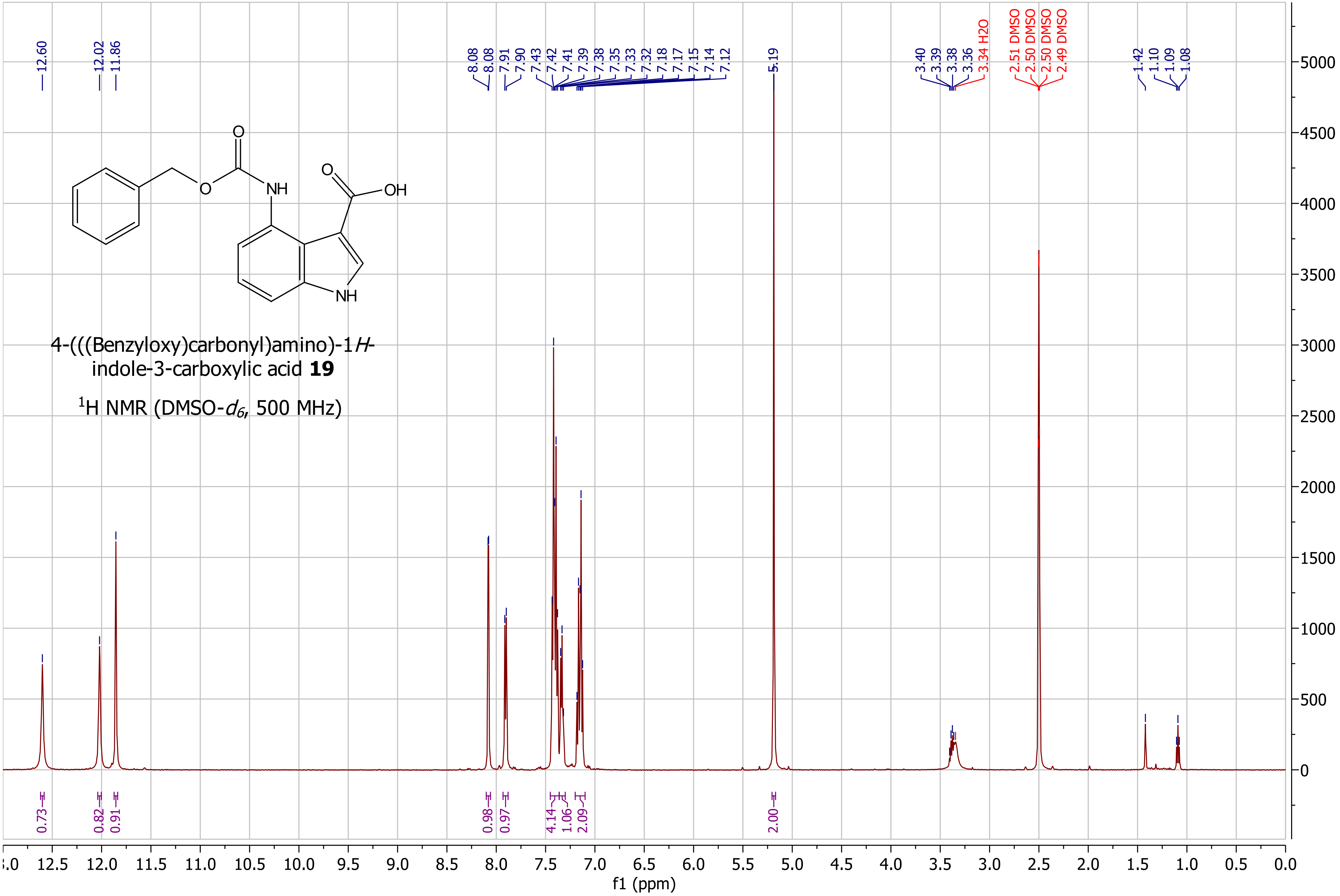

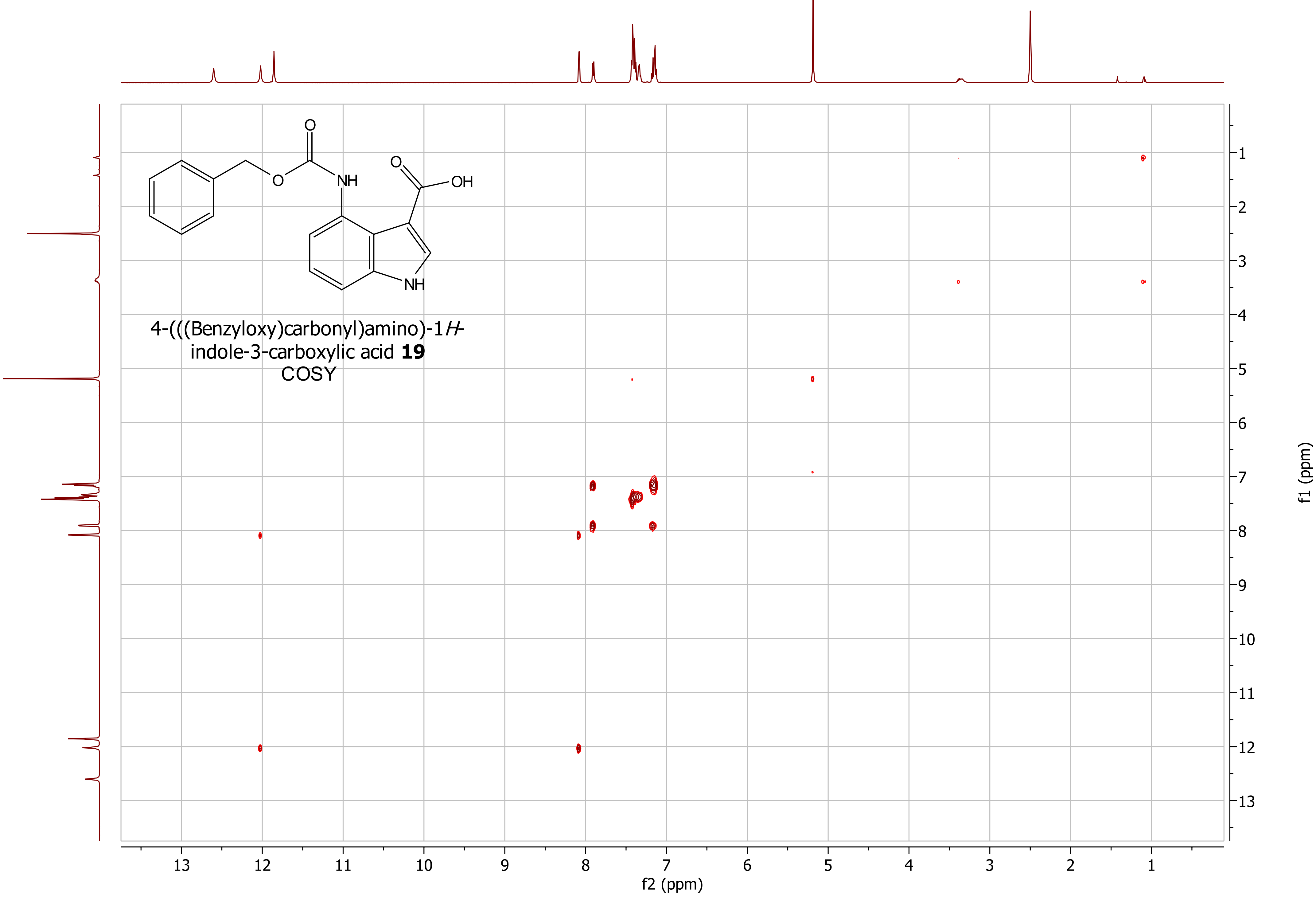
4-(((Benzyloxy)carbonyl)amino)-1*H*-indole-3-carboxylic acid **19**
COSY
O
O
NH
O
OH
NH
f2 (ppm)
f1 (ppm)
13
12
11
10
9
8
7
6
5
4
3
2
1

^{13}C NMR (126 MHz, DMSO-d_6) δ 168.7, 153.0, 138.0, 136.8, 133.9, 132.2, 128.4, 127.8, 127.5, 123.5, 115.4, 108.6, 106.8, 106.4, 65.4.

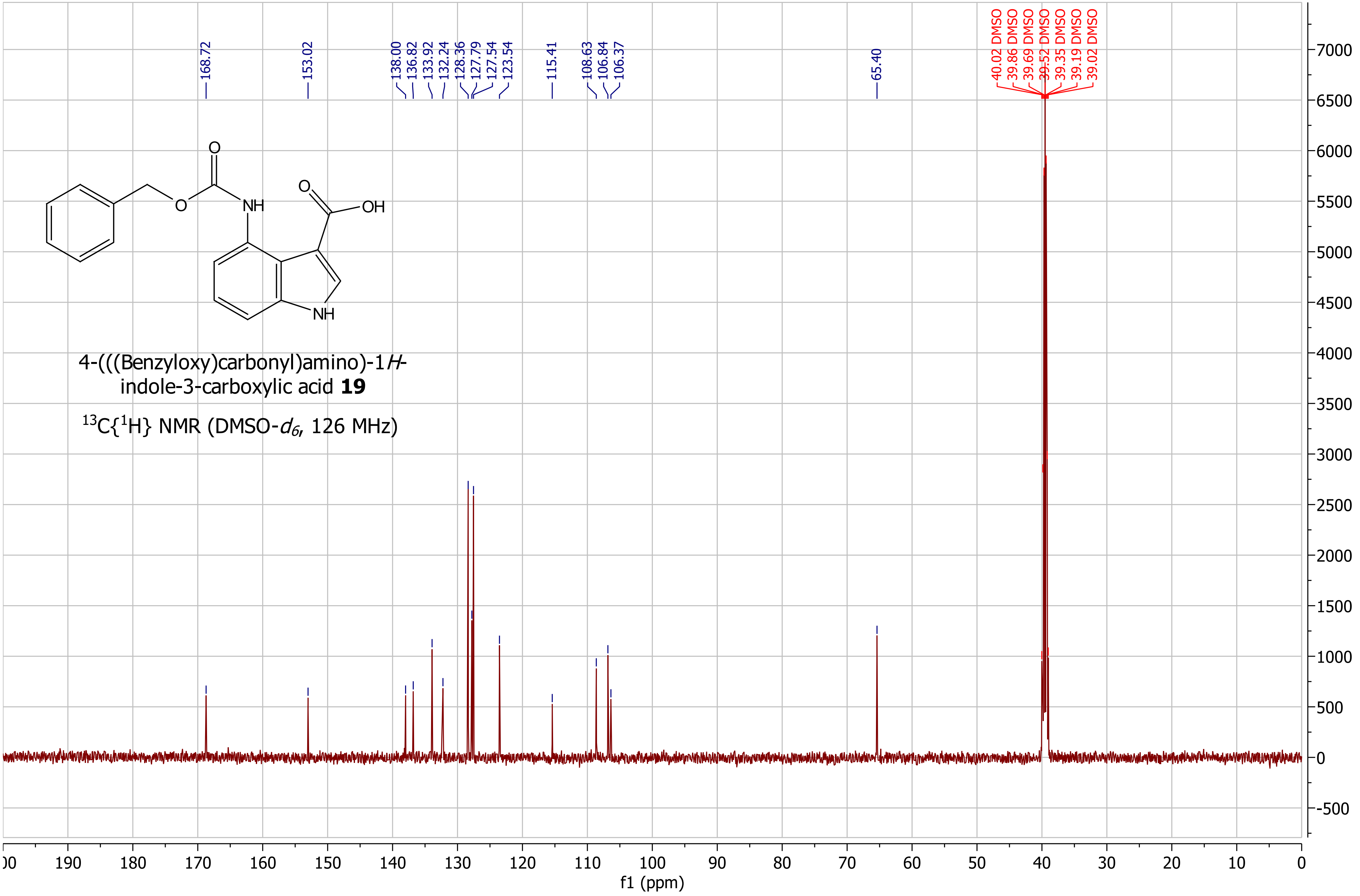

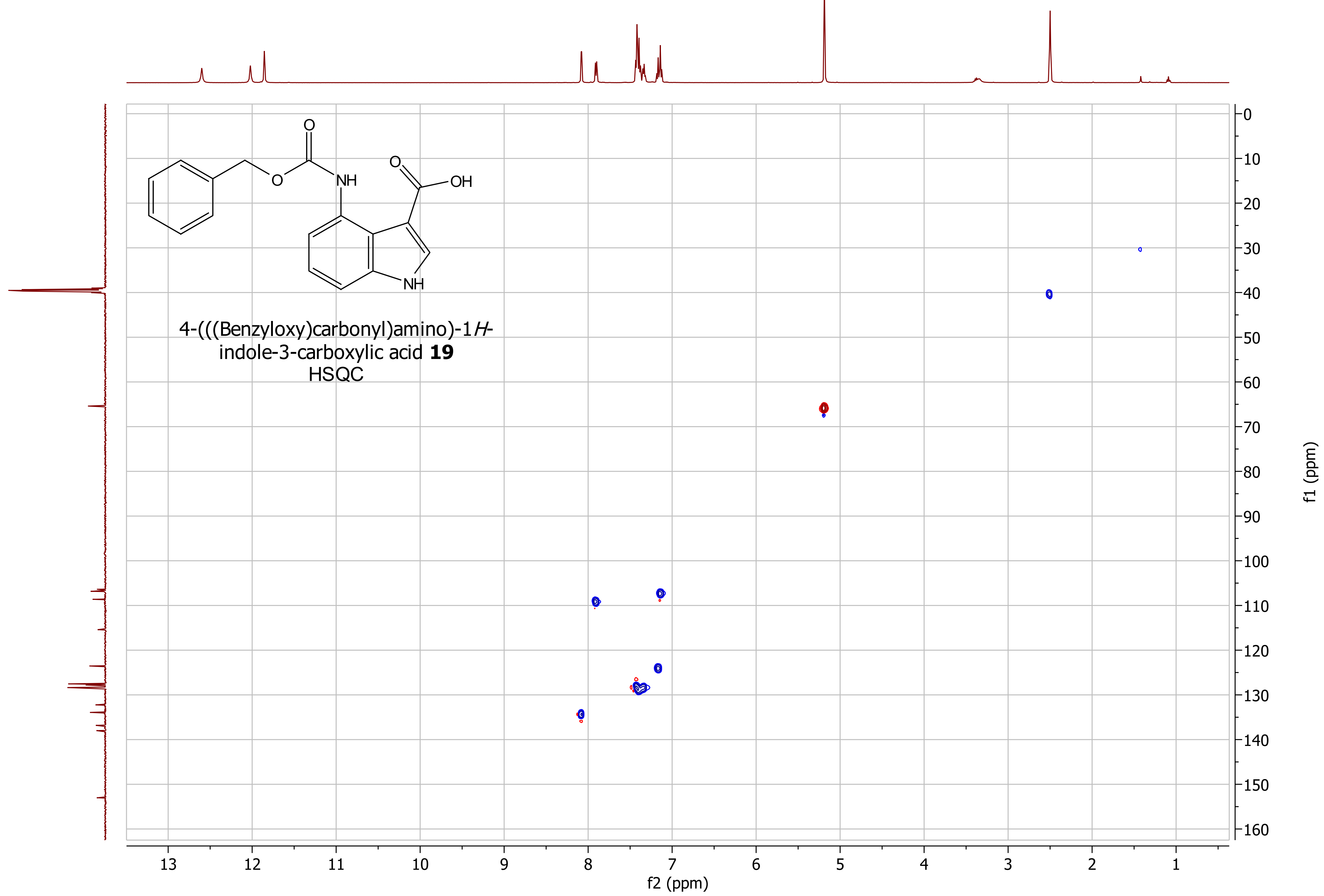
O
O
NH
O
OH
NH
4-(((Benzyloxy)carbonyl)amino)-1*H*-
indole-3-carboxylic acid **19**
HSQC
f1 (ppm)
f2 (ppm)
0
10
20
30
40
50
60
70
80
90
100
110
120
130
140
150
160
13
12
11
10
9
8
7
6
5
4
3
2
1

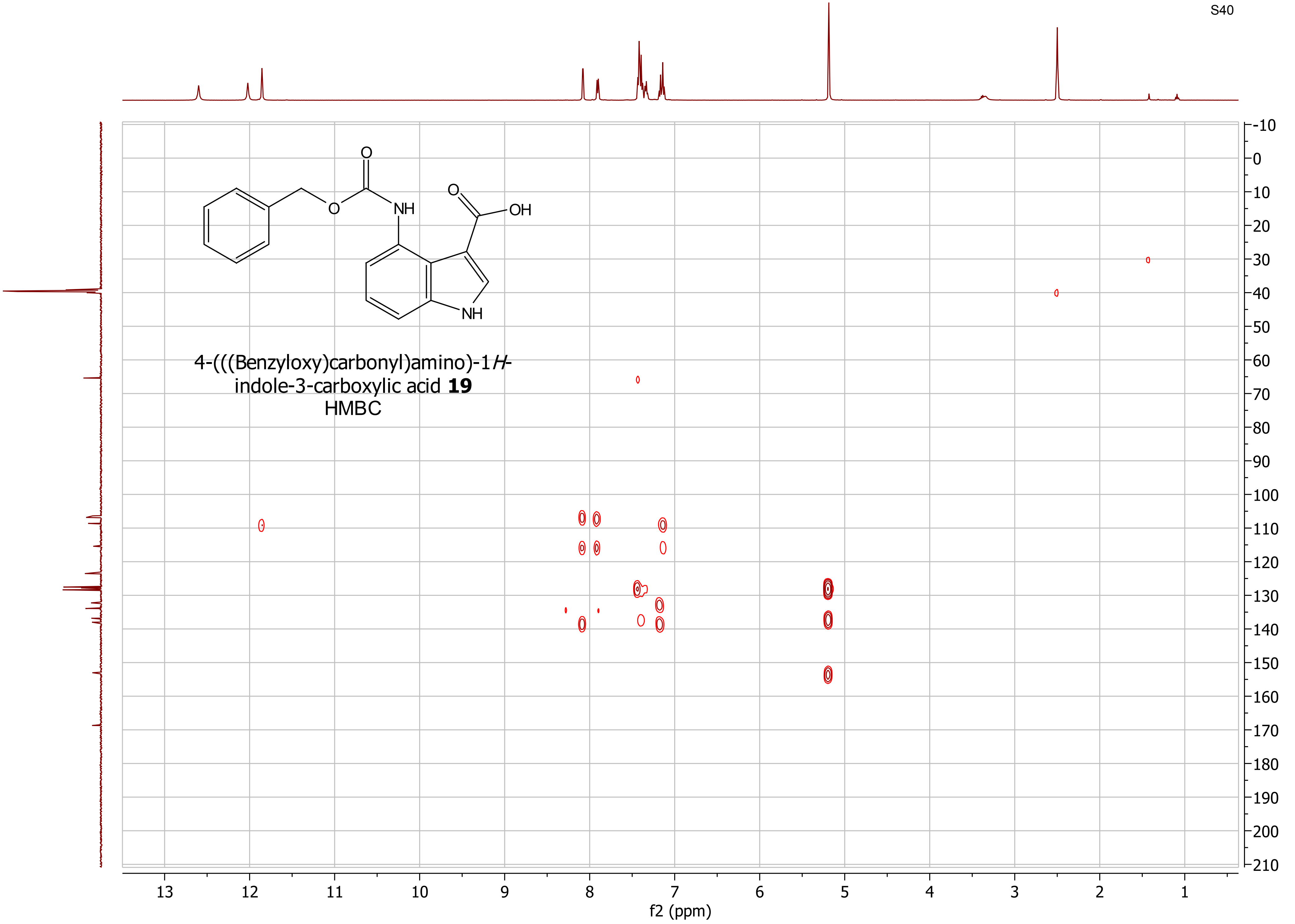

4-(((Benzyloxy)carbonyl)amino)-1*H*-indole-3-carboxylic acid **19**
HMBC

^{1}H NMR (500 MHz, DMSO-d_6) δ 11.01 (s, 1H), 8.83 (s, 1H), 8.42 (s, 1H), 7.46 – 7.38 (m, 4H), 7.35 (t, J = 6.6 Hz, 1H), 7.25 (d, J = 7.7 Hz, 1H), 7.21 (d, J = 2.6 Hz, 1H), 7.13 (d, J = 8.1 Hz, 1H), 7.05 (t, J = 7.9 Hz, 1H), 5.17 (s, 2H), 1.41 (s, 9H).

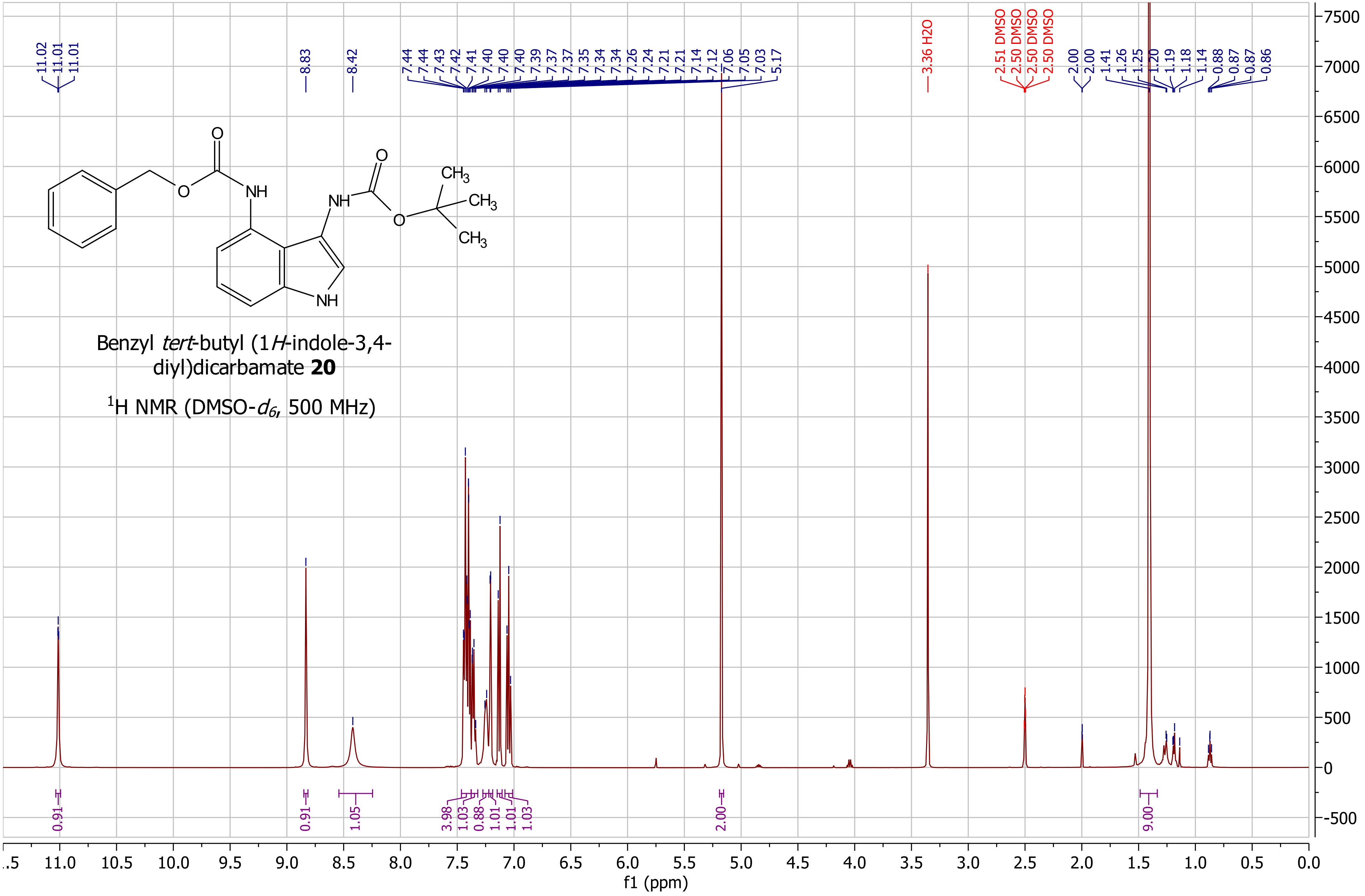

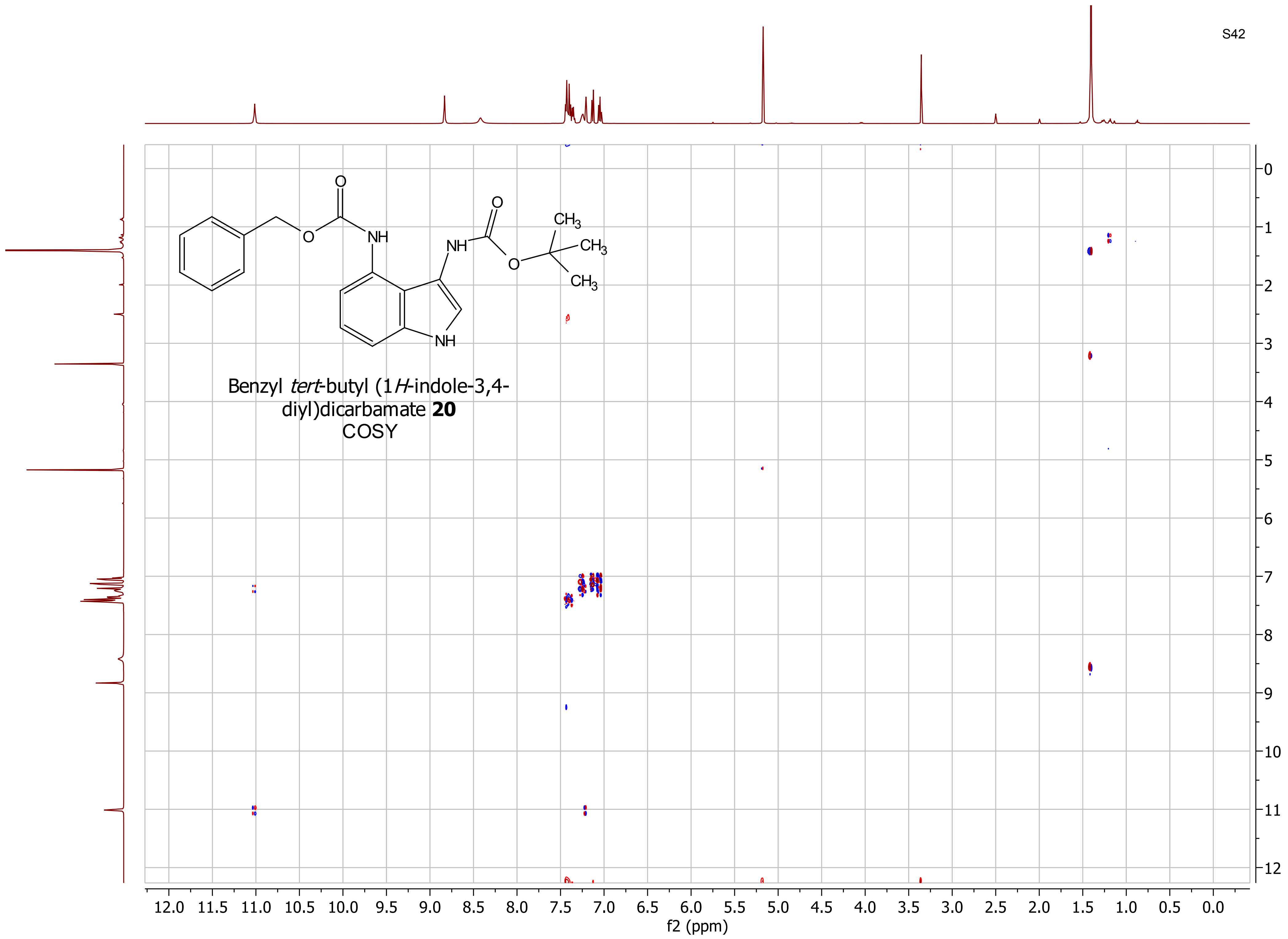

Benzyl *tert*-butyl (1*H*-indole-3,4-diyl)dicarbamate **20**
COSY

^{13}C NMR (126 MHz, DMSO-d_6) δ 155.6, 153.9, 136.6, 135.7, 129.5, 128.4, 128.0, 128.0, 121.4, 120.2, 116.3, 112.6, 112.0, 108.1, 78.8, 65.9, 28.1.

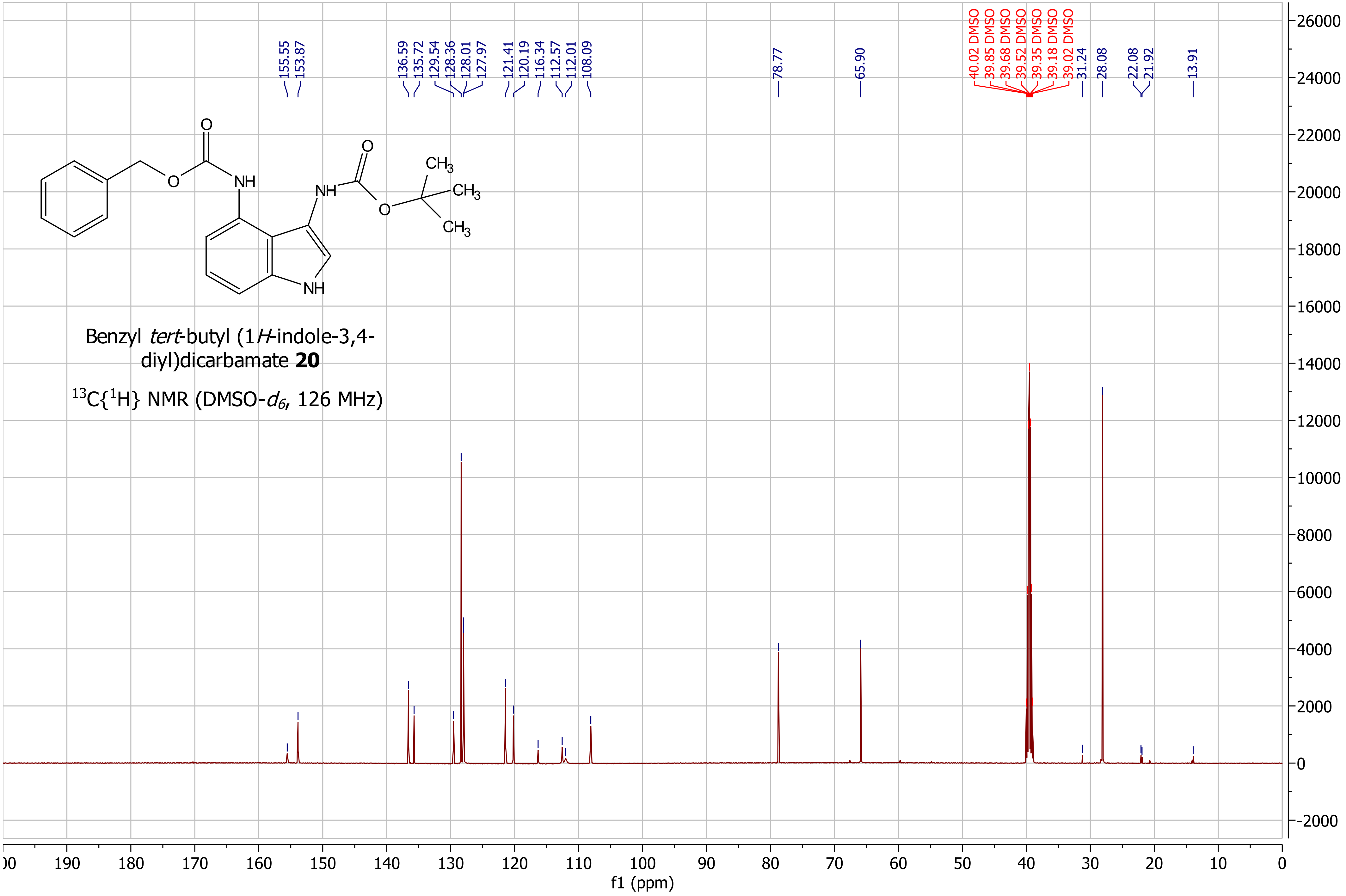

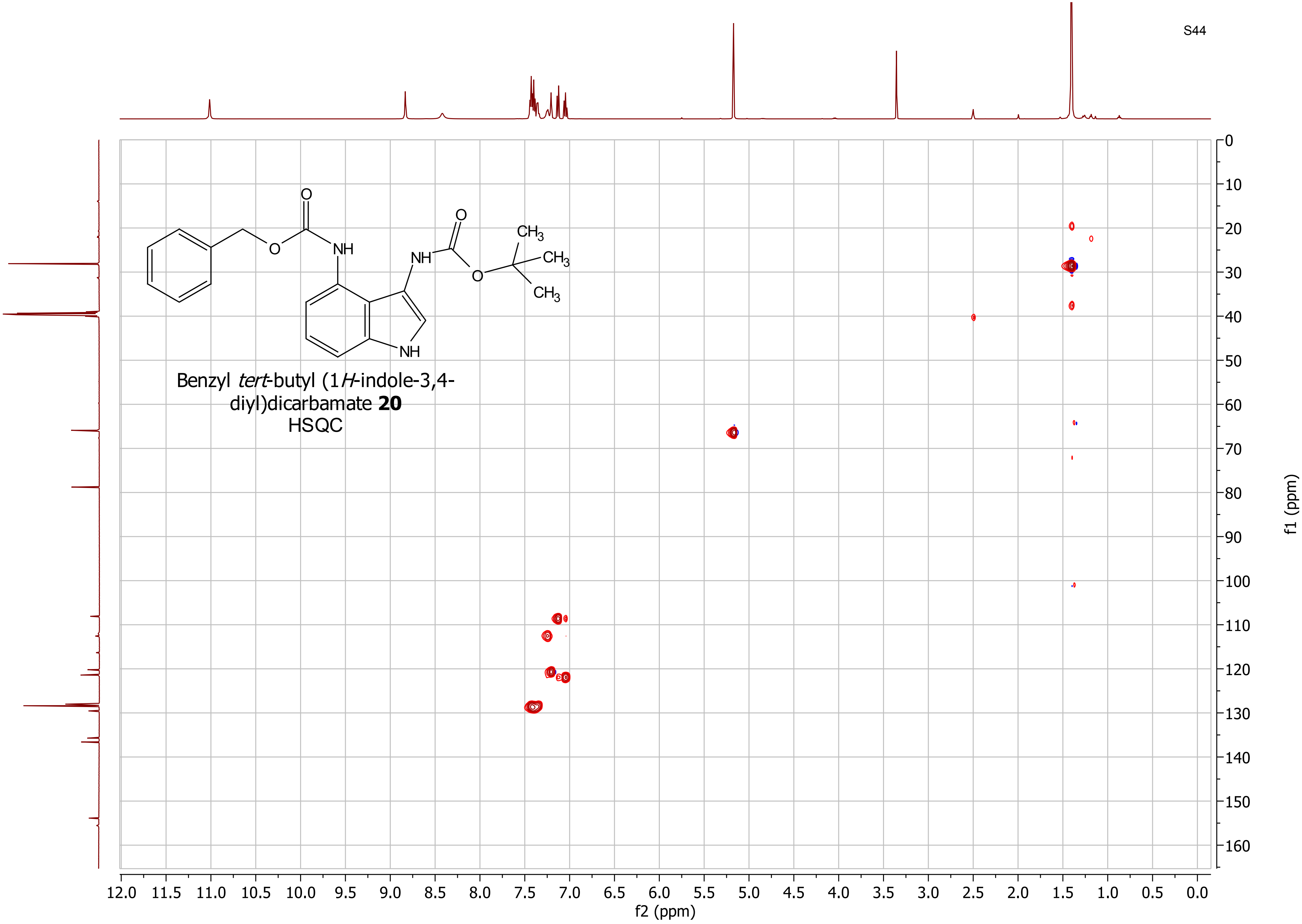

Benzyl *tert*-butyl (1*H*-indole-3,4-diyl)dicarbamate **20**
HSQC

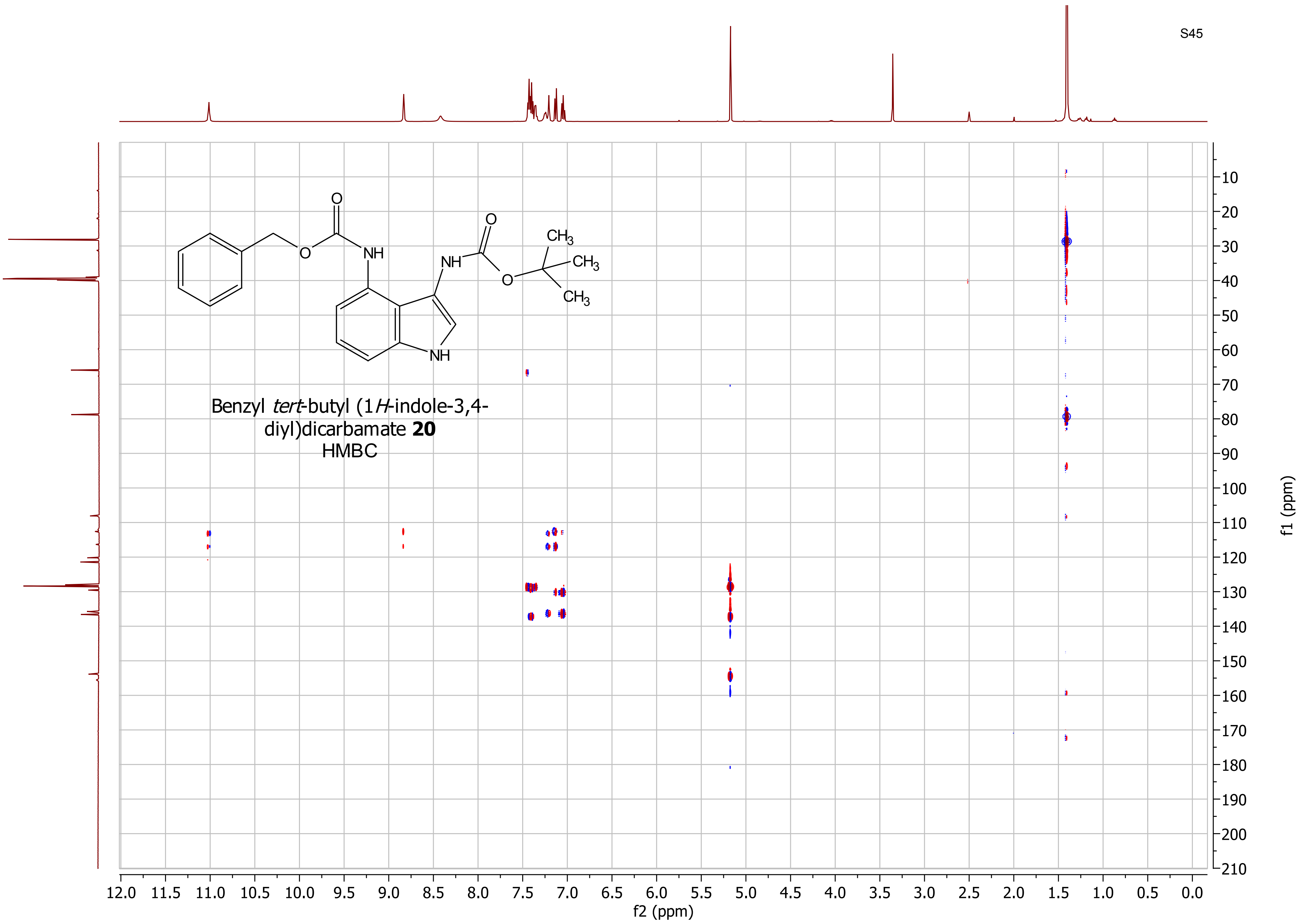
O
O
NH
NH
O
O
CH3
CH3
CH3
NH
Benzyl tert-butyl (1H-indole-3,4-diyl)dicarbamate 20
HMBC
f2 (ppm)
f1 (ppm)
12.0 11.5 11.0 10.5 10.0 9.5 9.0 8.5 8.0 7.5 7.0 6.5 6.0 5.5 5.0 4.5 4.0 3.5 3.0 2.5 2.0 1.5 1.0 0.5 0.0
10 20 30 40 50 60 70 80 90 100 110 120 130 140 150 160 170 180 190 200 210

^{1}H NMR (500 MHz, DMSO-d_6) δ 13.20 (s, 1H), 8.97 (d, J = 2.3 Hz, 1H), 8.74 (s, 1H), 8.21 (dd, J = 9.0, 2.3 Hz, 1H), 7.78 (d, J = 9.0 Hz, 1H).

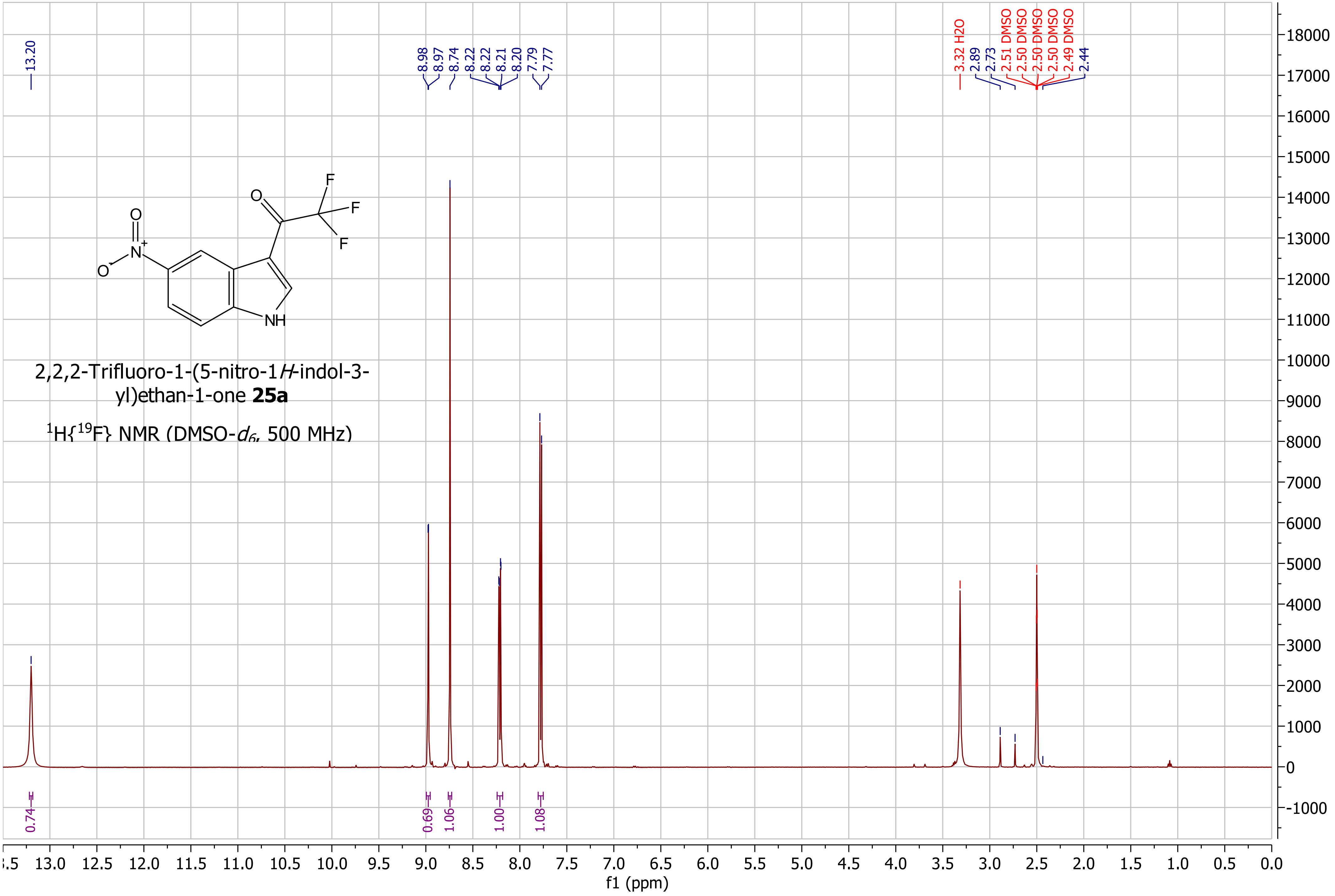

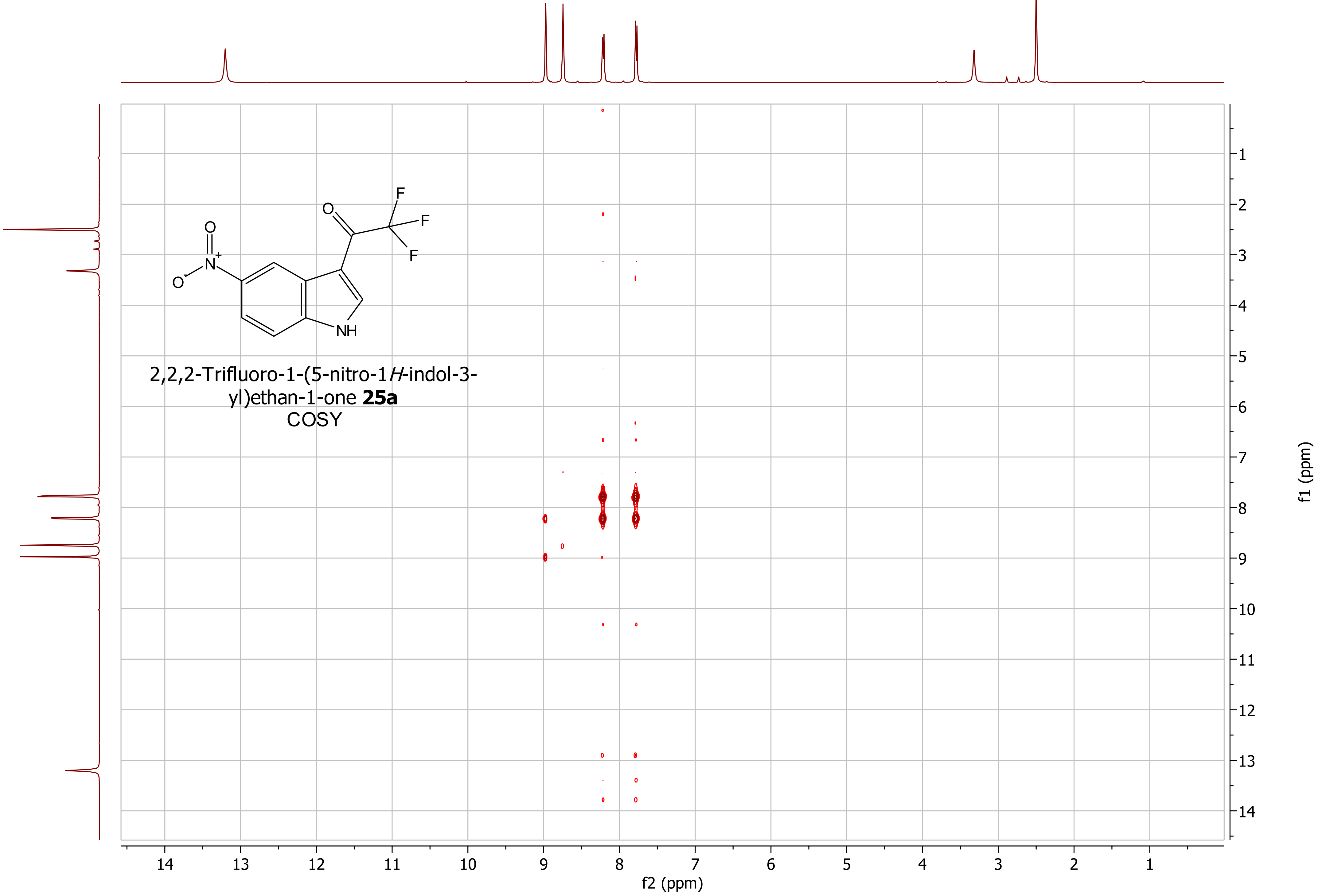

2,2,2-Trifluoro-1-(5-nitro-1*H*-indol-3-yl)ethan-1-one **25a**
COSY
f2 (ppm)
f1 (ppm)

^{13}C NMR (126 MHz, DMSO-d_6) δ 174.3 (d, J = 35.2 Hz), 143.7, 140.8 (d, J = 4.7 Hz), 139.9, 125.2, 119.6, 117.6, 117.1, 113.8, 110.0.

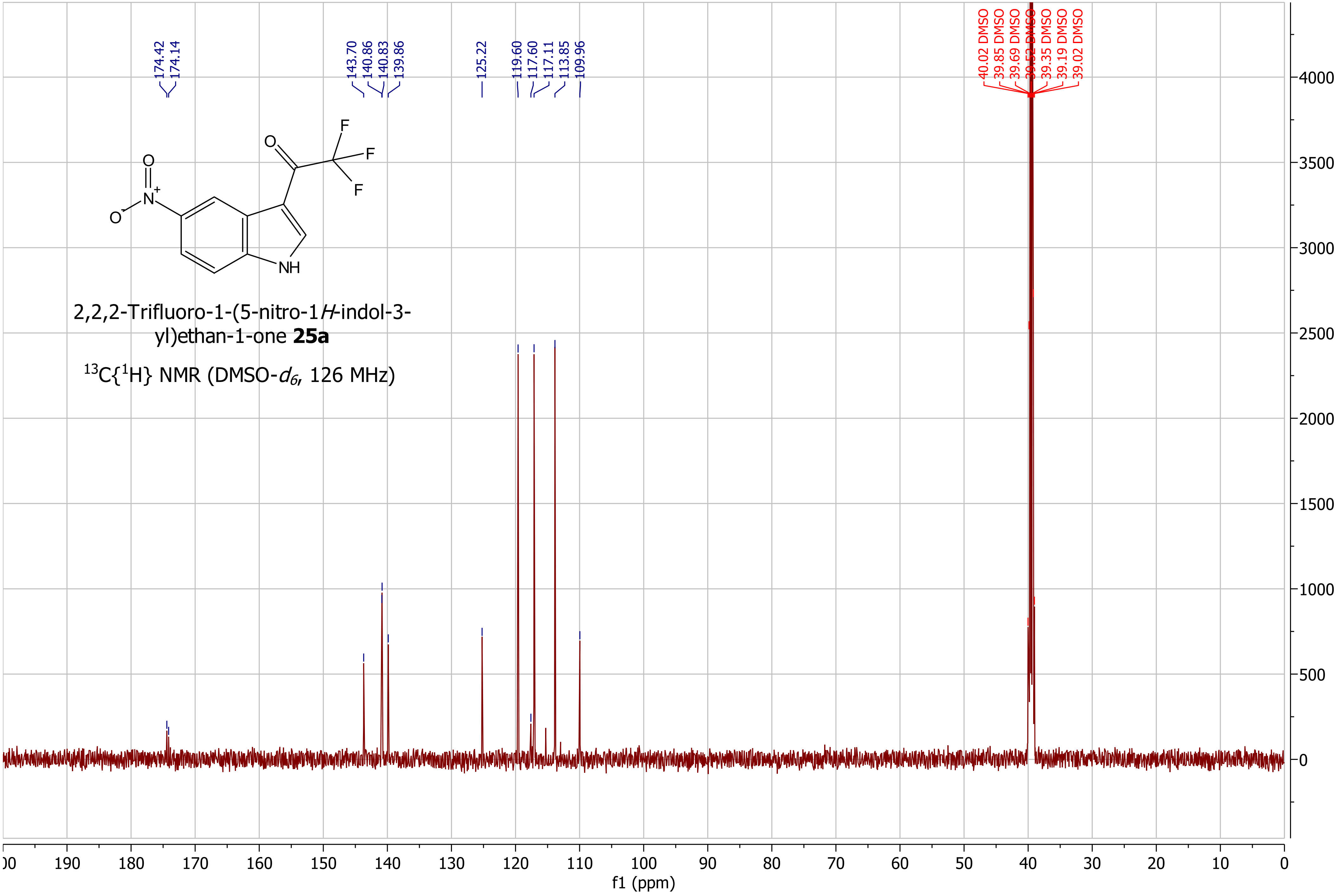

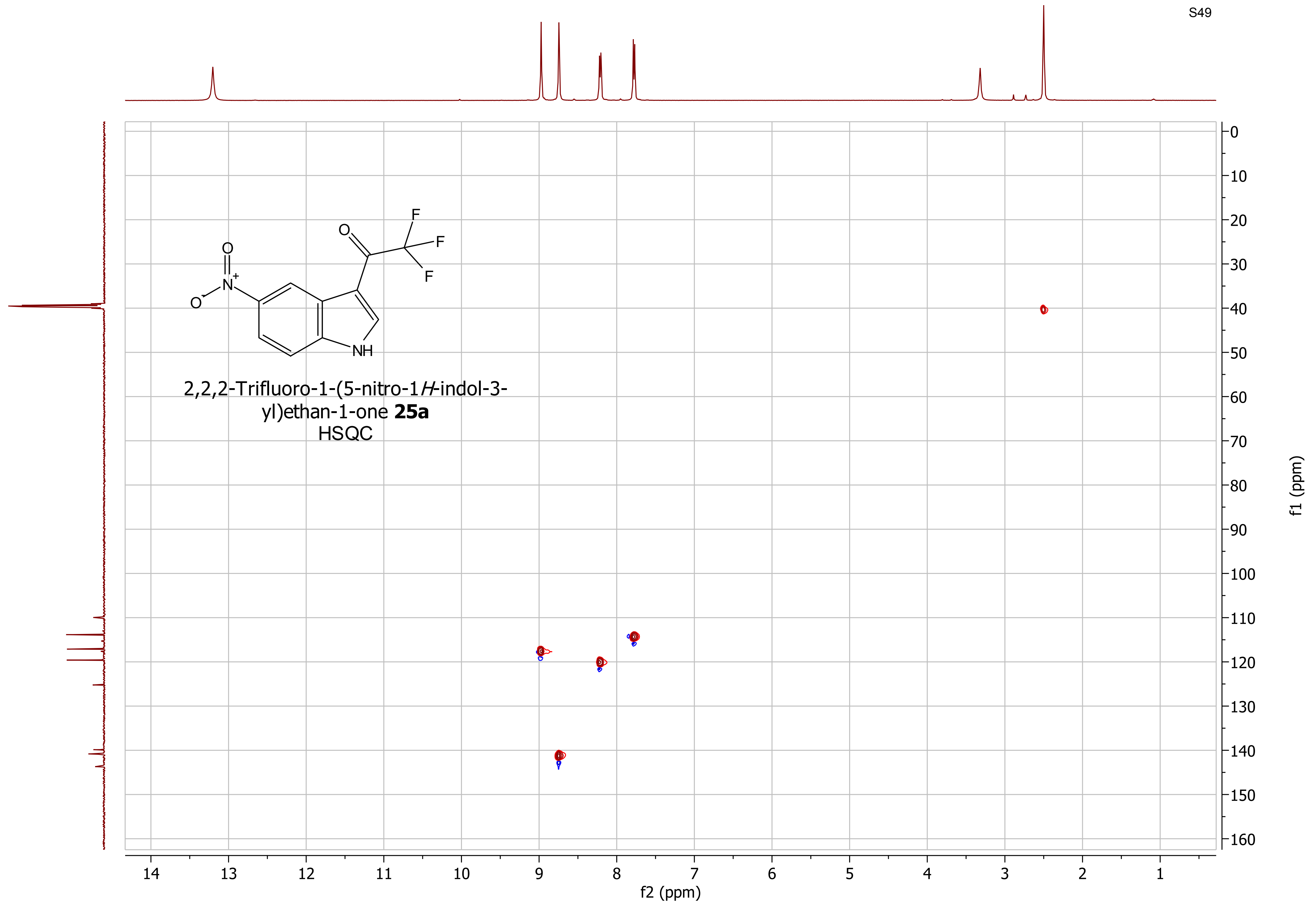

2,2,2-Trifluoro-1-(5-nitro-1*H*-indol-3-yl)ethan-1-one **25a**
HSQC
f2 (ppm)
f1 (ppm)

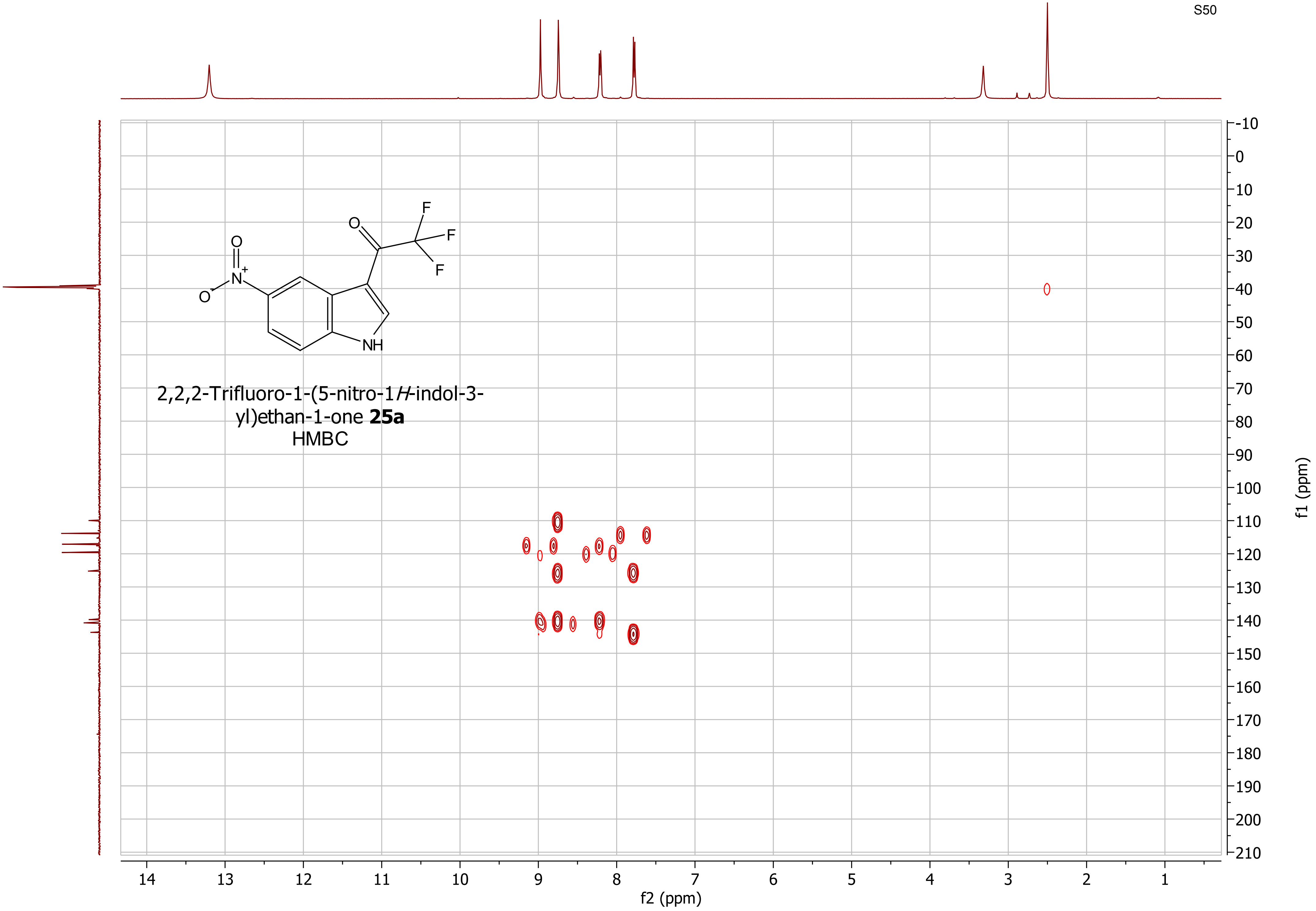
2,2,2-Trifluoro-1-(5-nitro-1H-indol-3-yl)ethan-1-one 25a
HMBC
f2 (ppm)
f1 (ppm)
14 13 12 11 10 9 8 7 6 5 4 3 2 1
-10 0 10 20 30 40 50 60 70 80 90 100 110 120 130 140 150 160 170 180 190 200 210
O
F
F
F
O
N+
O-
NH

^{19}F NMR (471 MHz, DMSO-d_6) δ -71.94.

2,2,2-Trifluoro-1-(5-nitro-1*H*-indol-3-yl)ethan-1-one **25a**

^{19}F{^{1}H} NMR (DMSO-d_6, 471 MHz)

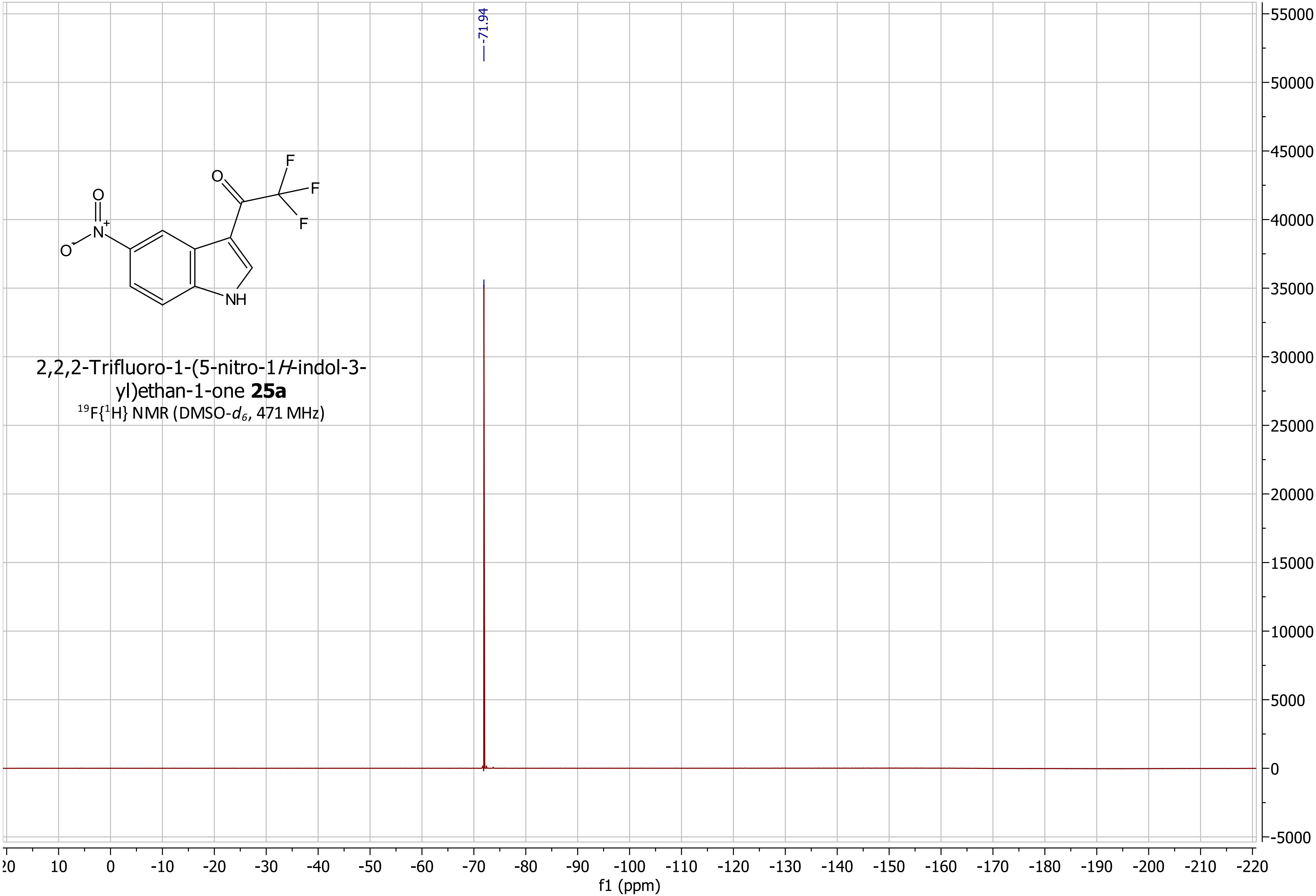

^{1}H NMR (500 MHz, DMSO-d_6) δ 13.19 (s, 1H), 8.80 (s, 1H), 8.44 (d, J = 2.1 Hz, 1H), 8.33 (d, J = 8.8 Hz, 1H), 8.19 (dd, J = 8.8, 2.2 Hz, 1H).

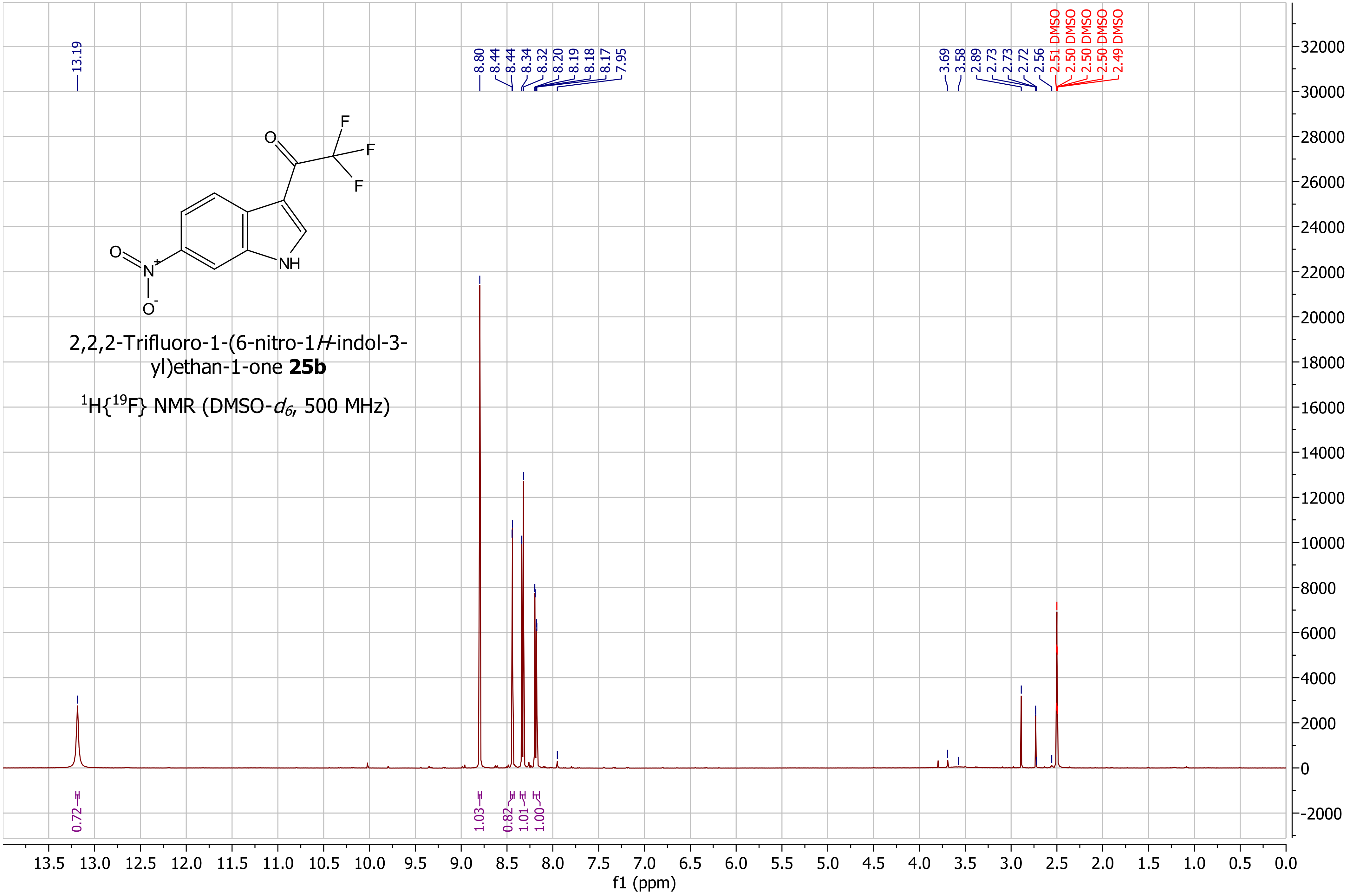

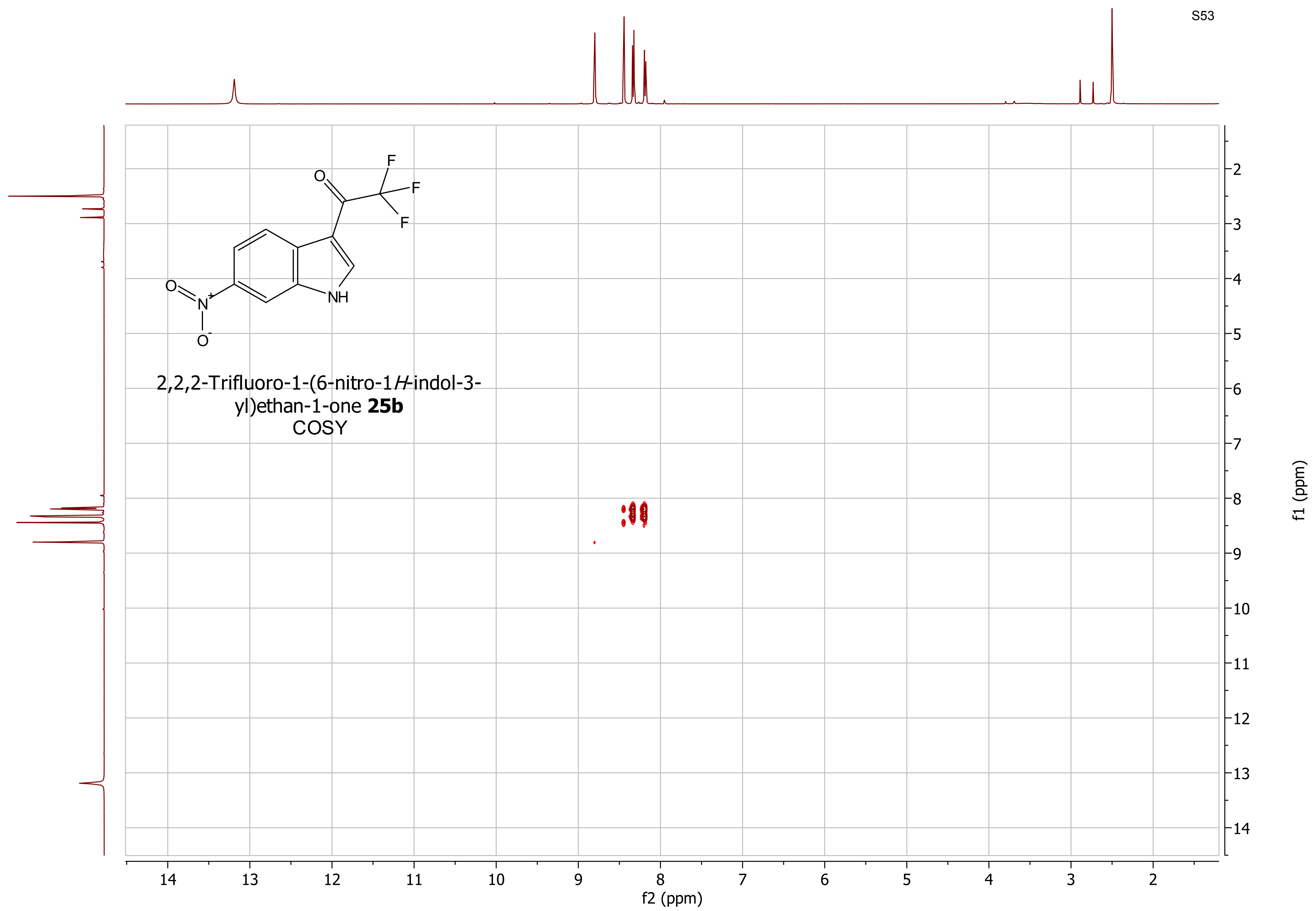

O
F
F
F
NH
O
N+
O-
2,2,2-Trifluoro-1-(6-nitro-1*H*-indol-3-yl)ethan-1-one **25b**
COSY
f2 (ppm)
f1 (ppm)

^{13}C NMR (126 MHz, DMSO-d_6) δ 174.2 (d, J = 34.9 Hz), 144.1, 141.8 (q, J = 4.6 Hz), 135.5, 130.6, 121.4, 118.2, 109.4, 108.9.

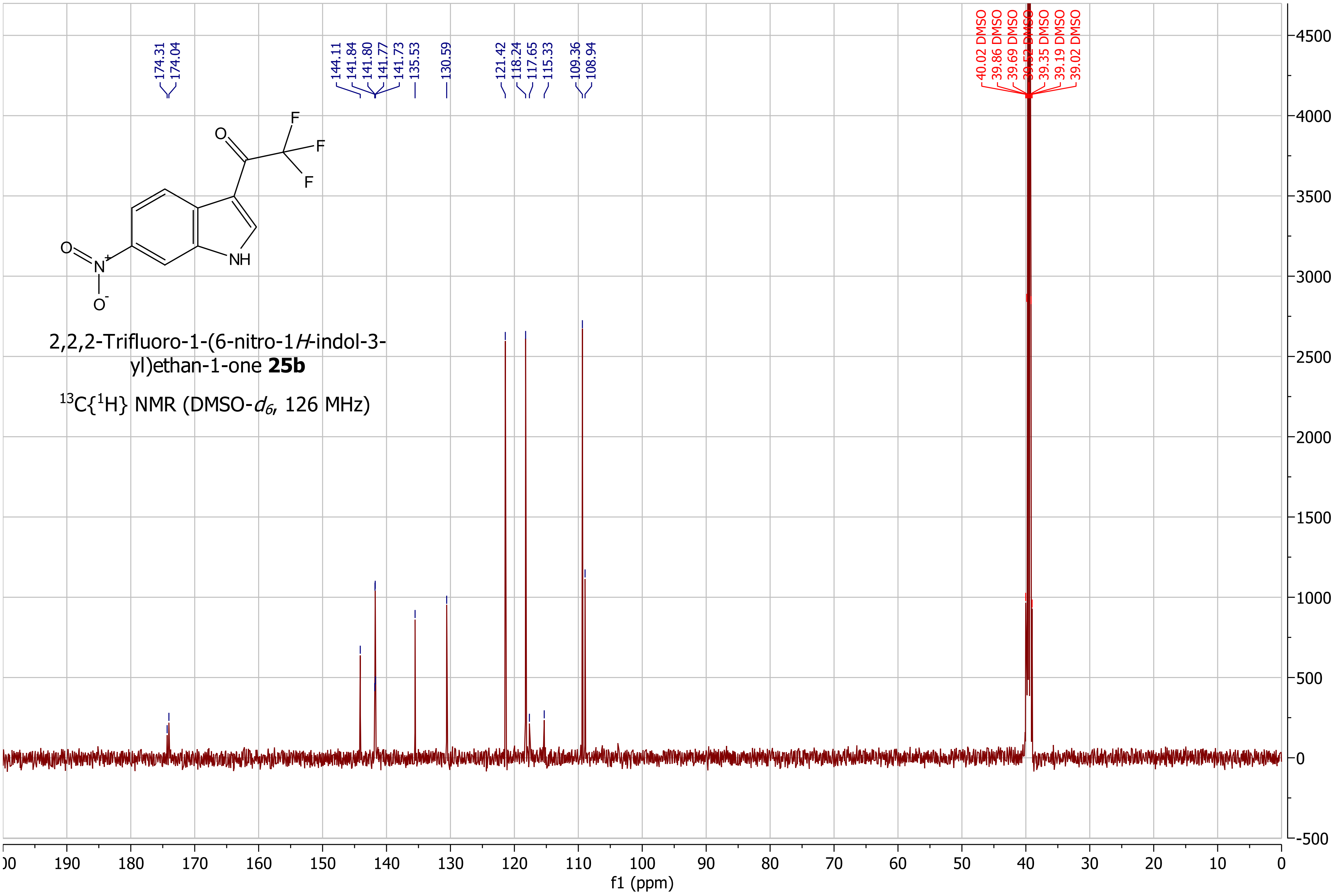

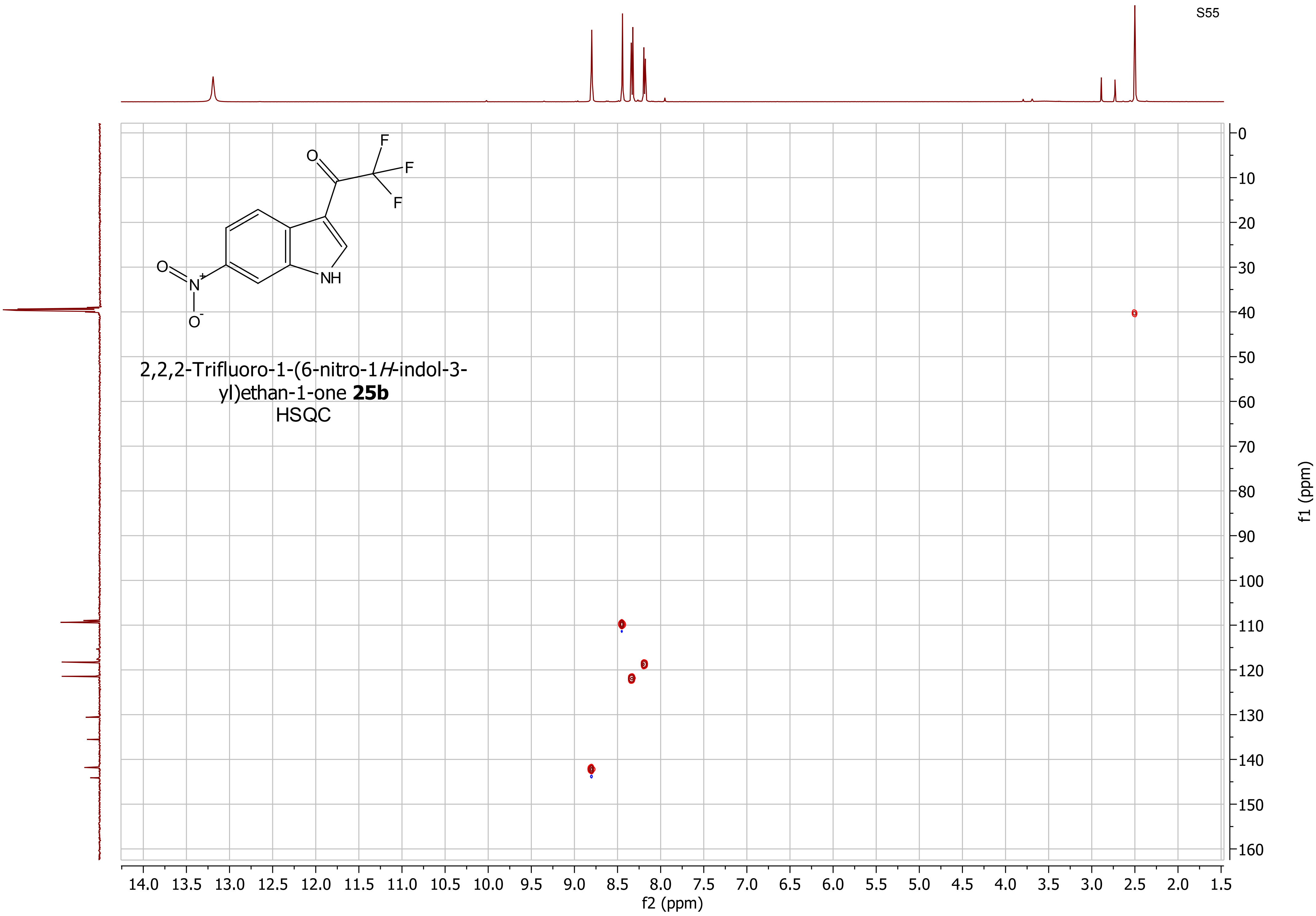
2,2,2-Trifluoro-1-(6-nitro-1*H*-indol-3-yl)ethan-1-one **25b**
HSQC
f2 (ppm)
f1 (ppm)

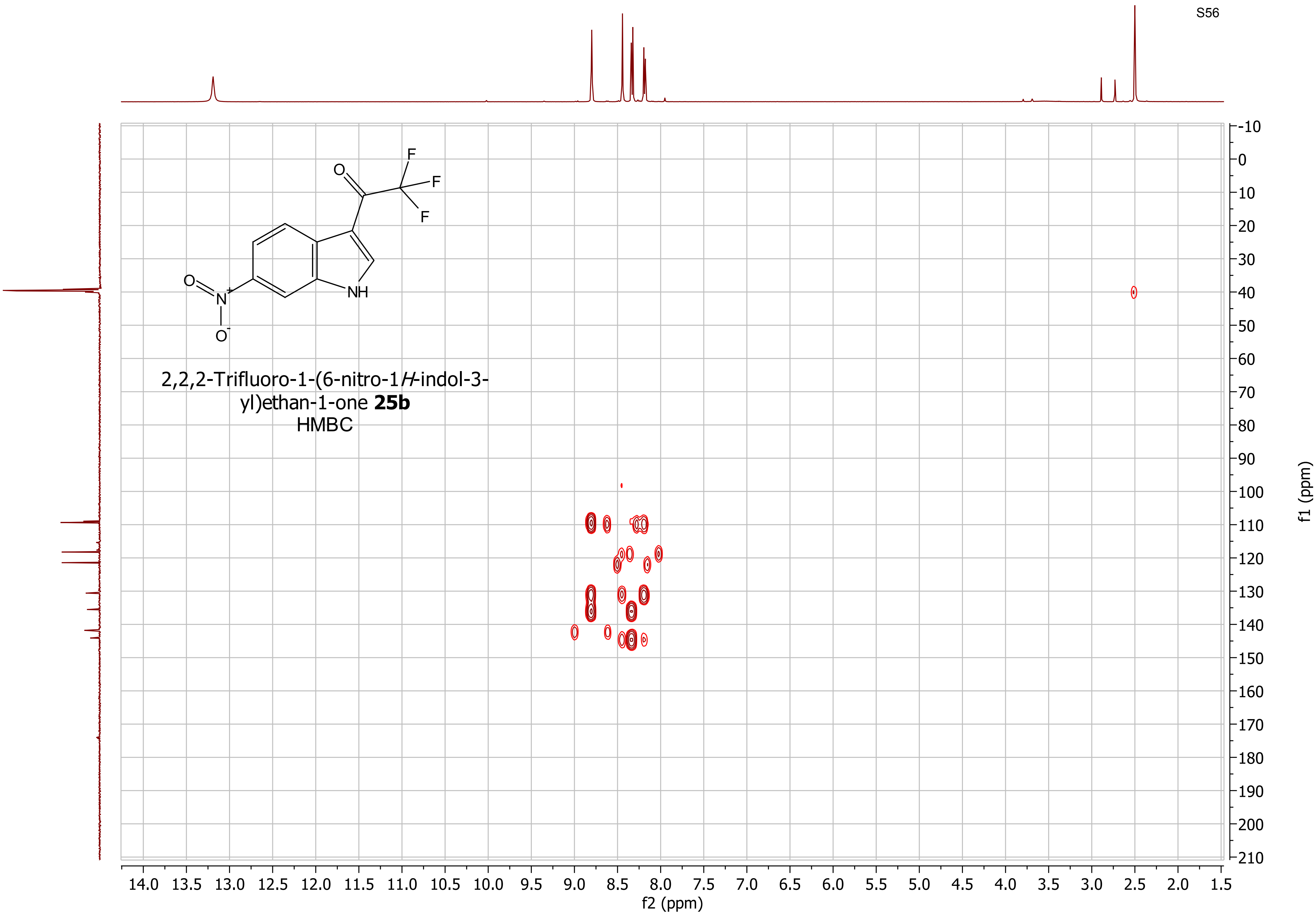

2,2,2-Trifluoro-1-(6-nitro-1*H*-indol-3-yl)ethan-1-one **25b**
HMBC

^{19}F NMR (471 MHz, DMSO-d_6) δ -71.94.

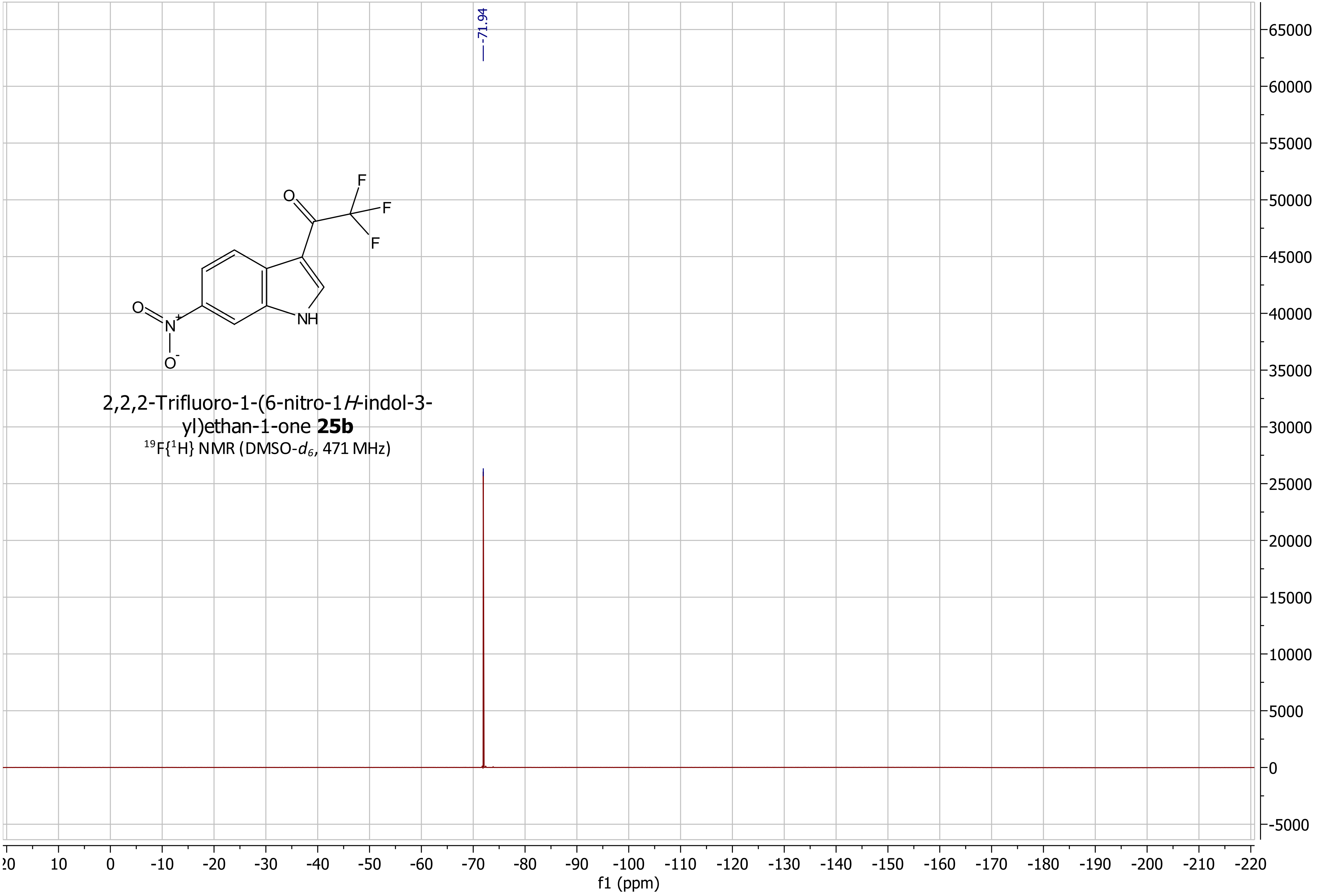

2,2,2-Trifluoro-1-(6-nitro-1*H*-indol-3-yl)ethan-1-one **25b**

$^{19}F\{^1H\}$ NMR (DMSO-d_6, 471 MHz)

^{1}H NMR (500 MHz, DMSO-d_6) δ 13.14 (s, 1H), 8.62 (dd, J = 7.9, 1.1 Hz, 1H), 8.37 (s, 1H), 8.28 (dd, J = 8.1, 1.1 Hz, 1H), 7.56 (t, J = 8.0 Hz, 1H).

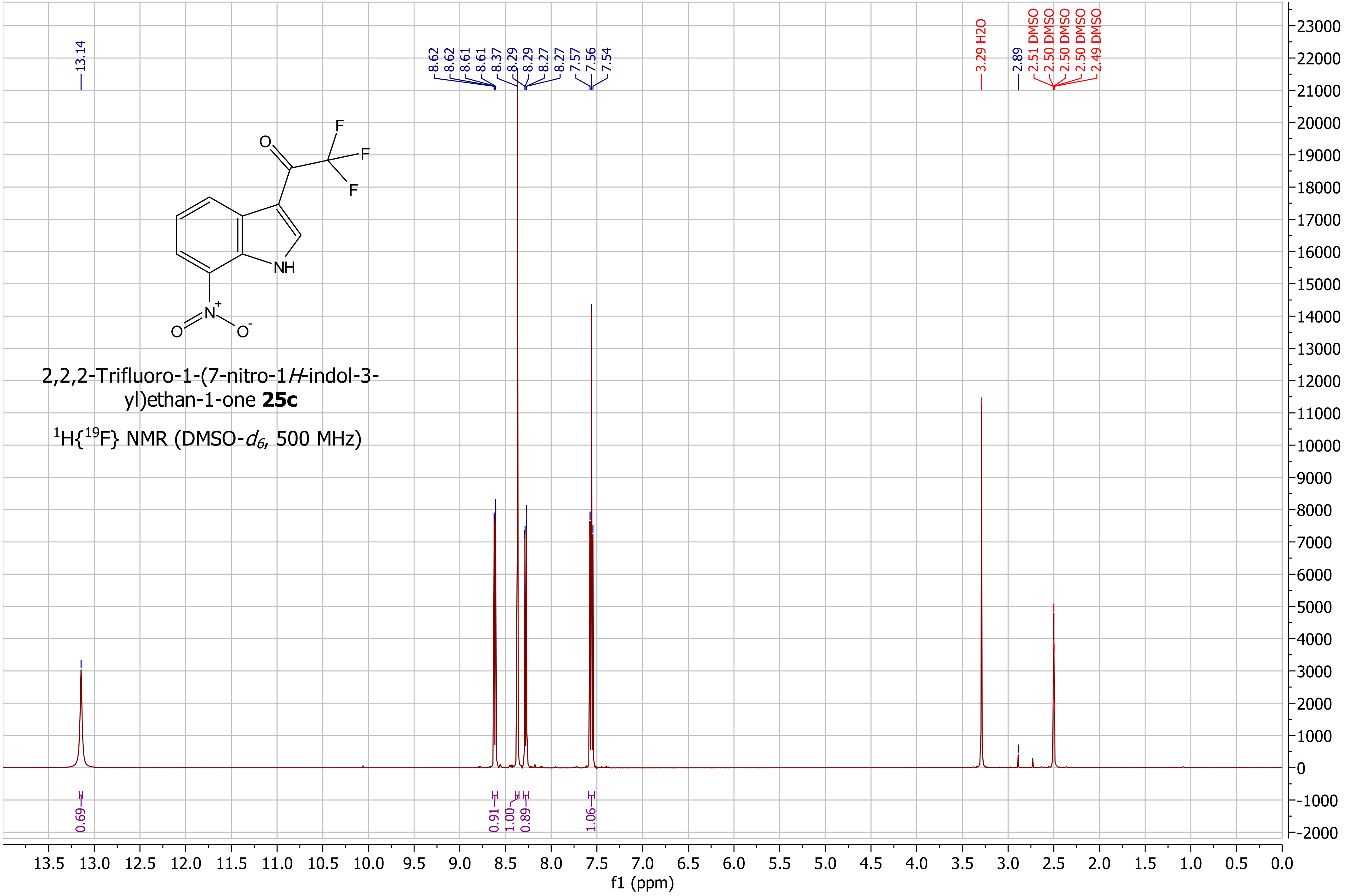

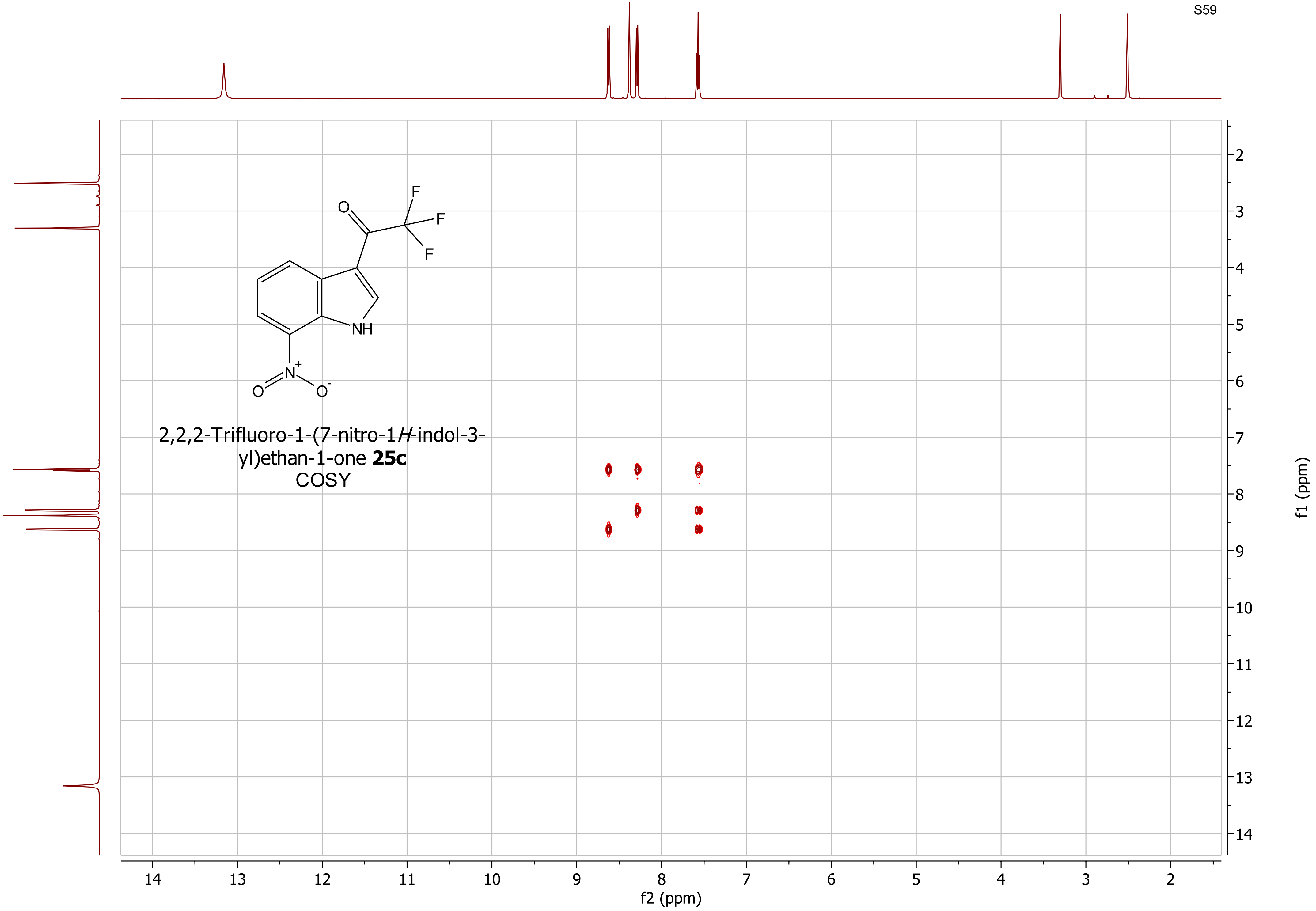
2,2,2-Trifluoro-1-(7-nitro-1*H*-indol-3-yl)ethan-1-one **25c**
COSY
f2 (ppm)
f1 (ppm)
14 13 12 11 10 9 8 7 6 5 4 3 2

^{13}C NMR (126 MHz, DMSO-d_6) δ 174.5 (d, J = 35.1 Hz), 138.9 (d, J = 4.9 Hz), 133.8, 129.0, 128.9, 128.8, 123.4, 121.0, 116.4 (q, J = 291.2 Hz), 109.5.

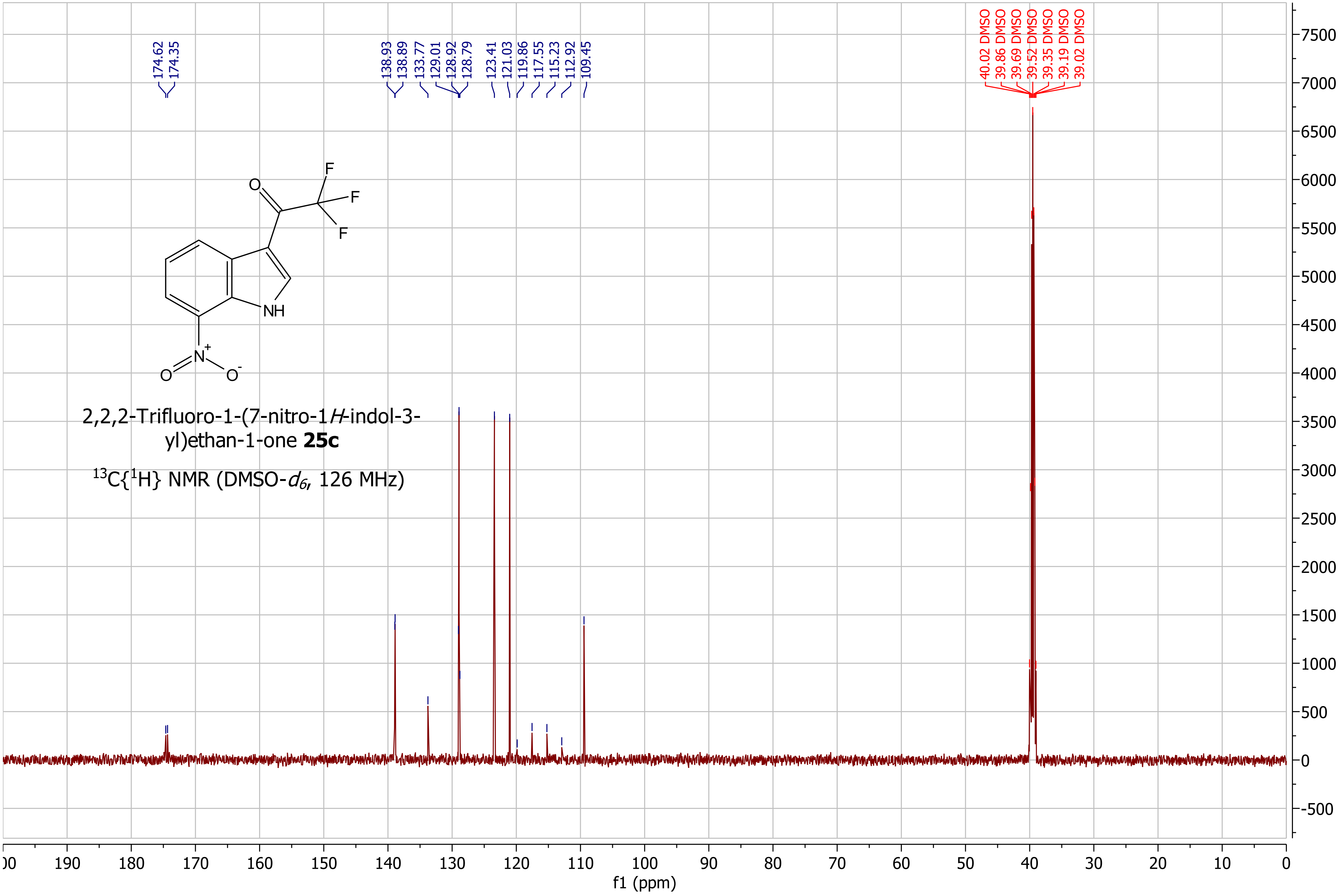

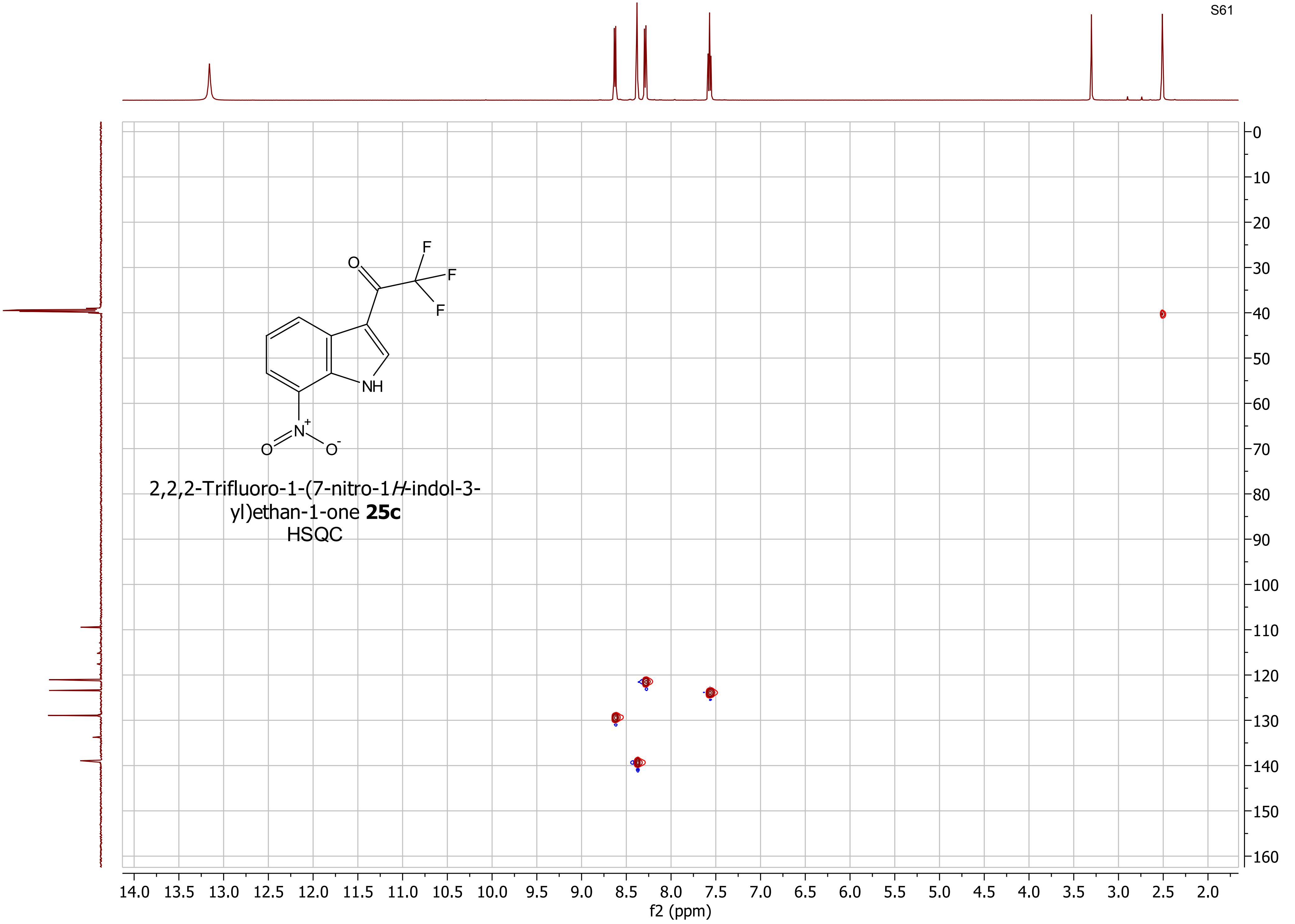
2,2,2-Trifluoro-1-(7-nitro-1*H*-indol-3-yl)ethan-1-one **25c**
HSQC
f2 (ppm)
f1 (ppm)

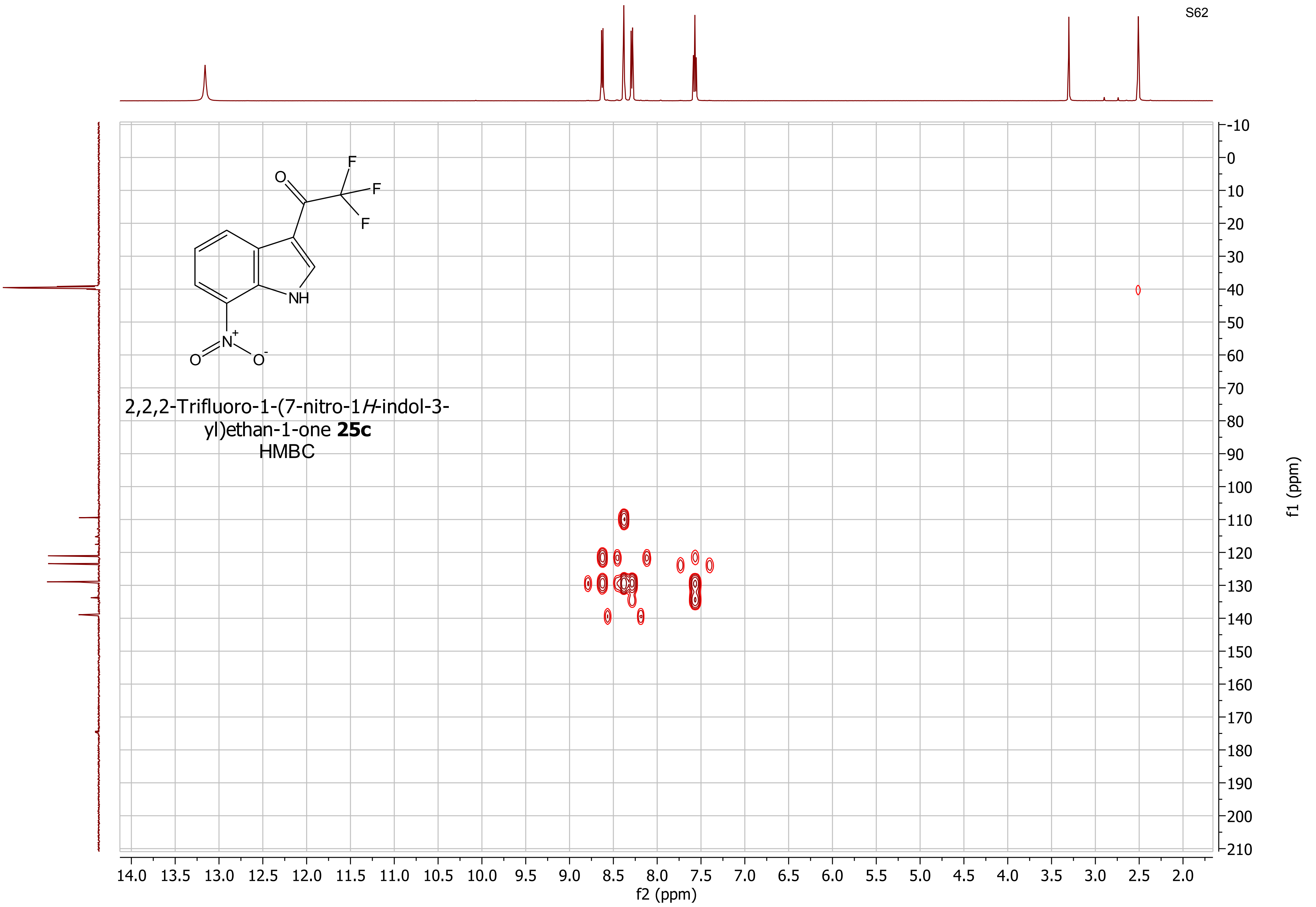

2,2,2-Trifluoro-1-(7-nitro-1*H*-indol-3-yl)ethan-1-one **25c**
HMBC

^{19}F NMR (471 MHz, DMSO-d_6) δ -71.99.

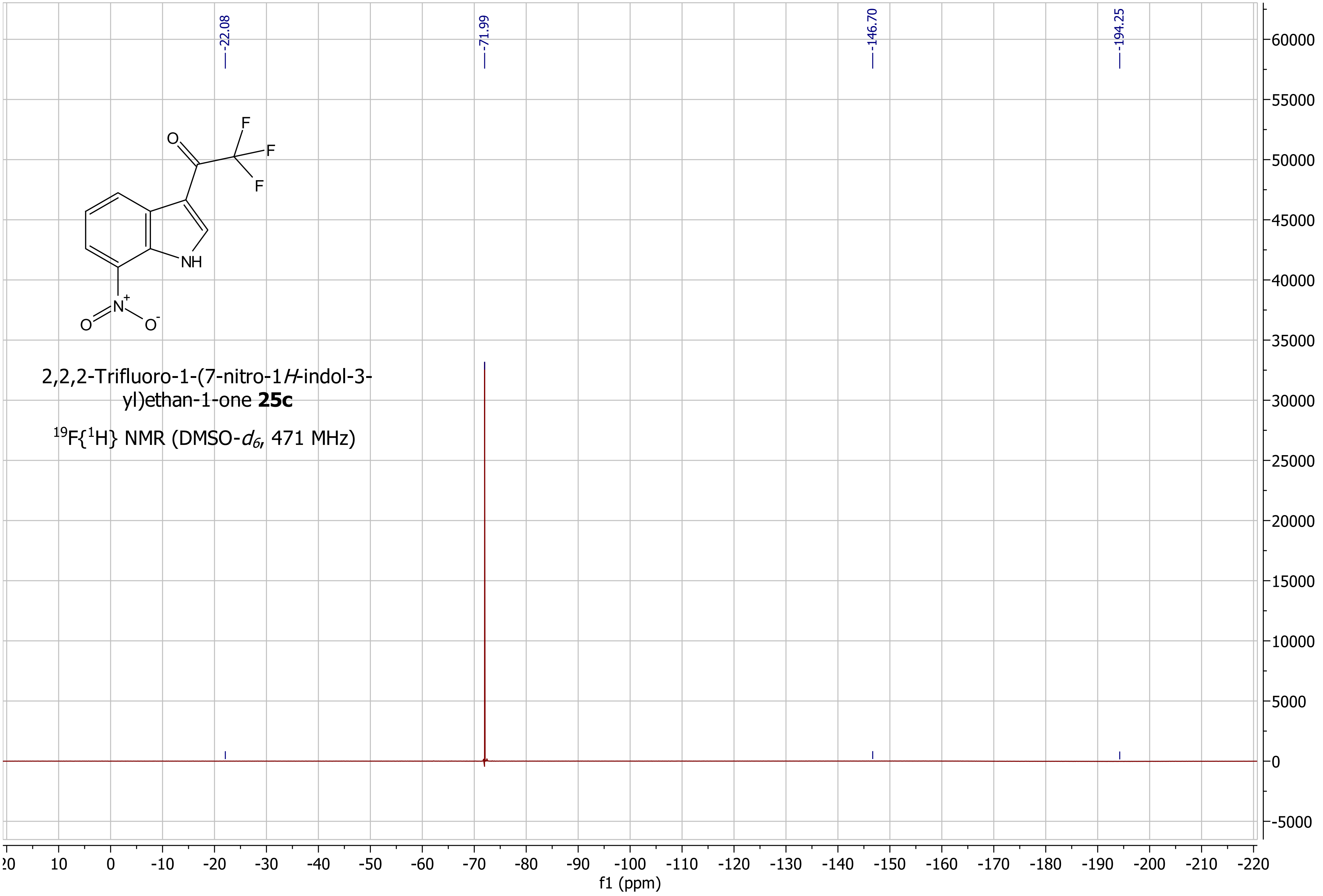

^{1}H NMR (500 MHz, DMSO-d_6) δ 12.54 (s, 1H), 12.41 (s, 1H), 8.89 (d, J = 2.3 Hz, 1H), 8.26 (s, 1H), 8.09 (dd, J = 8.9, 2.4 Hz, 1H), 7.67 (d, J = 8.9 Hz, 1H).

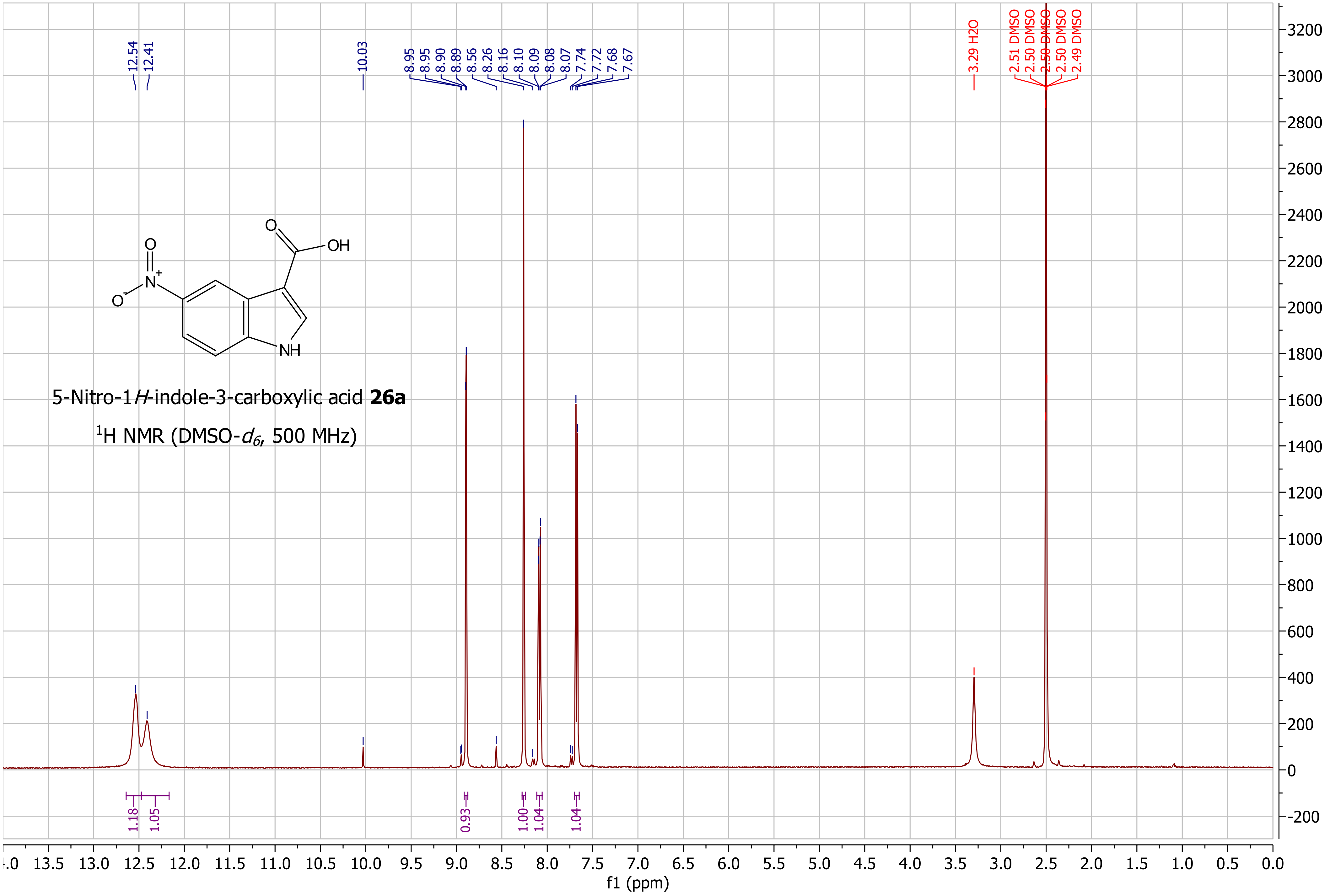

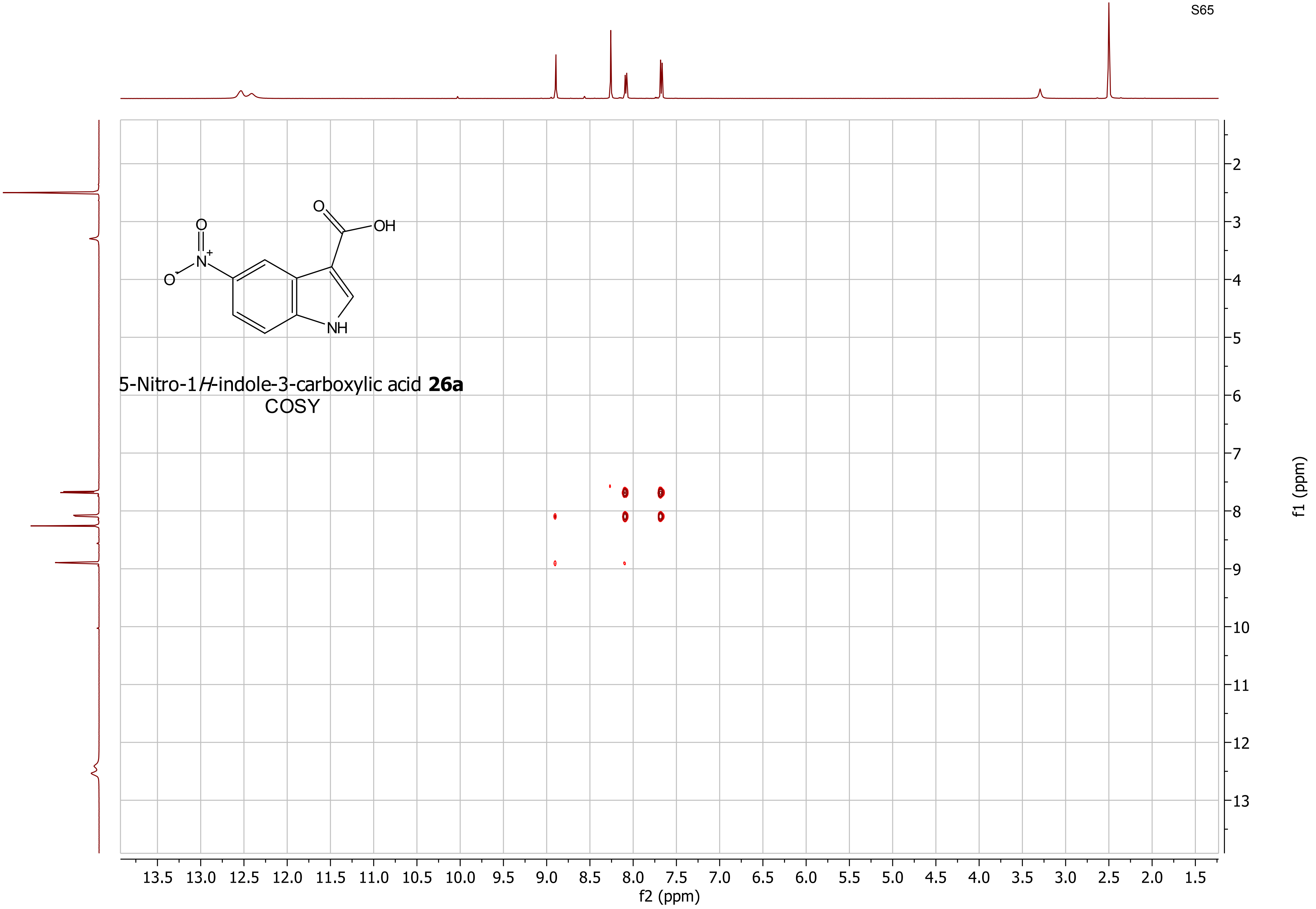

O
OH
O
N+
O-
NH
5-Nitro-1*H*-indole-3-carboxylic acid **26a**
COSY
13.5 13.0 12.5 12.0 11.5 11.0 10.5 10.0 9.5 9.0 8.5 8.0 7.5 7.0 6.5 6.0 5.5 5.0 4.5 4.0 3.5 3.0 2.5 2.0 1.5
f2 (ppm)
2 3 4 5 6 7 8 9 10 11 12 13
f1 (ppm)

^{13}C NMR (126 MHz, DMSO-d_6) δ 165.1, 142.2, 139.6, 135.7, 125.3, 117.5, 117.0, 113.0, 109.5.

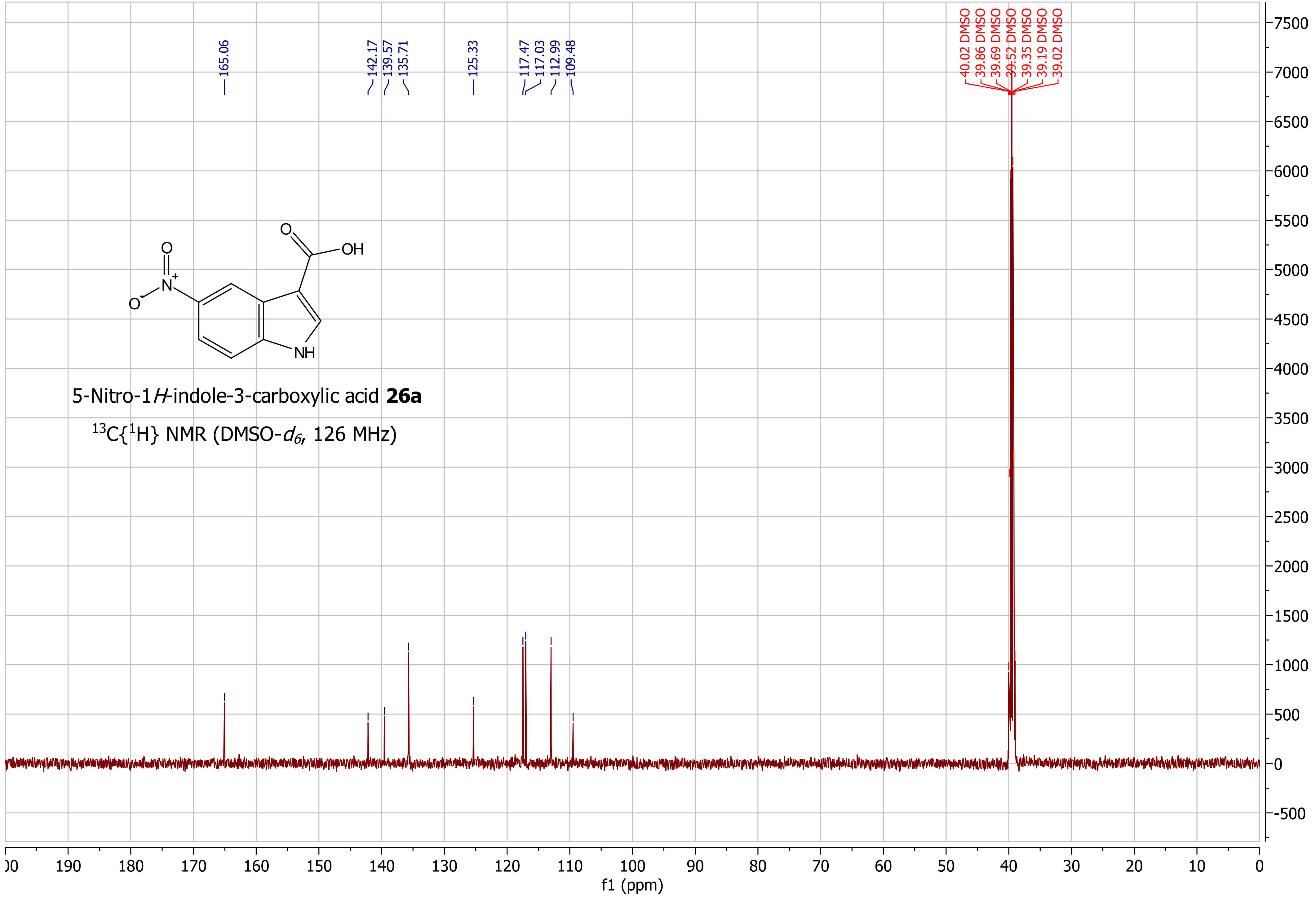

5-Nitro-1*H*-indole-3-carboxylic acid **26a**

^{13}C{^{1}H} NMR (DMSO-d_6, 126 MHz)

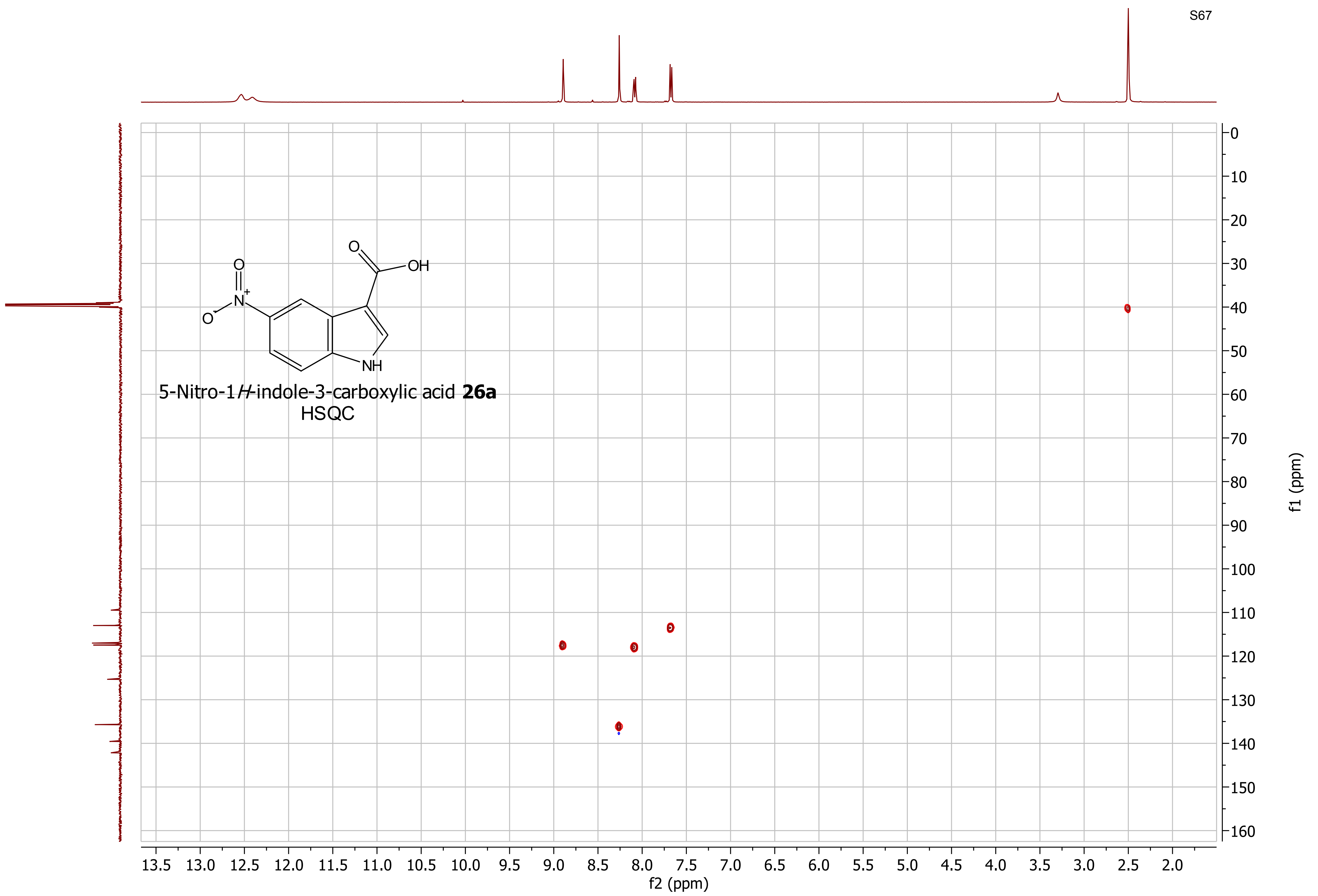
5-Nitro-1H-indole-3-carboxylic acid 26a
HSQC
f2 (ppm)
f1 (ppm)

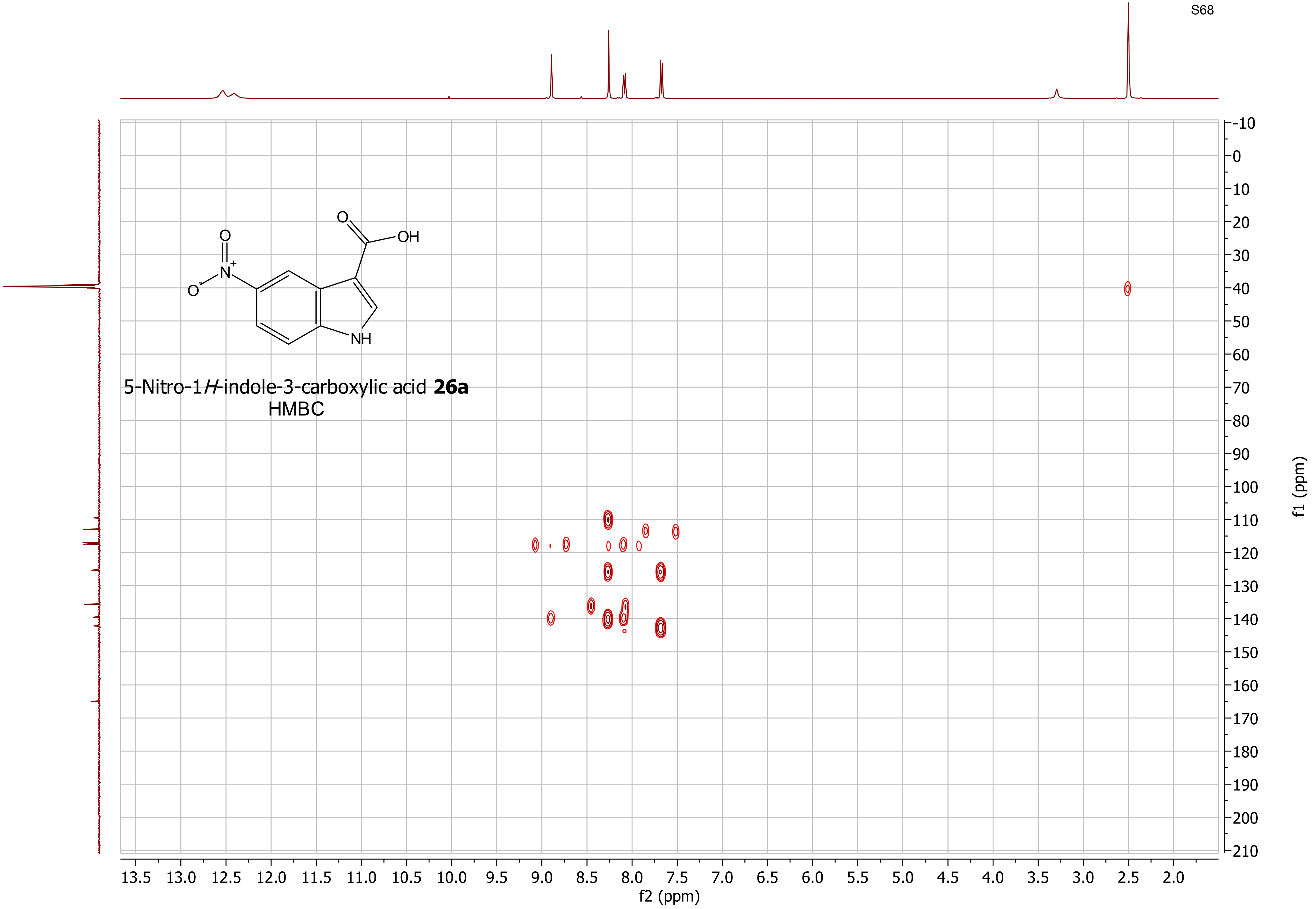
O
OH
O
N+
O-
NH
5-Nitro-1H-indole-3-carboxylic acid 26a
HMBC
13.5 13.0 12.5 12.0 11.5 11.0 10.5 10.0 9.5 9.0 8.5 8.0 7.5 7.0 6.5 6.0 5.5 5.0 4.5 4.0 3.5 3.0 2.5 2.0
f2 (ppm)
-10 0 10 20 30 40 50 60 70 80 90 100 110 120 130 140 150 160 170 180 190 200 210
f1 (ppm)

^{1}H NMR (500 MHz, DMSO-d_6) δ 12.53 (s, 1H), 12.35 (s, 1H), 8.40 (d, J = 2.1 Hz, 1H), 8.37 (d, J = 3.0 Hz, 1H), 8.16 (d, J = 8.9 Hz, 1H), 8.05 (dd, J = 8.9, 2.1 Hz, 1H).

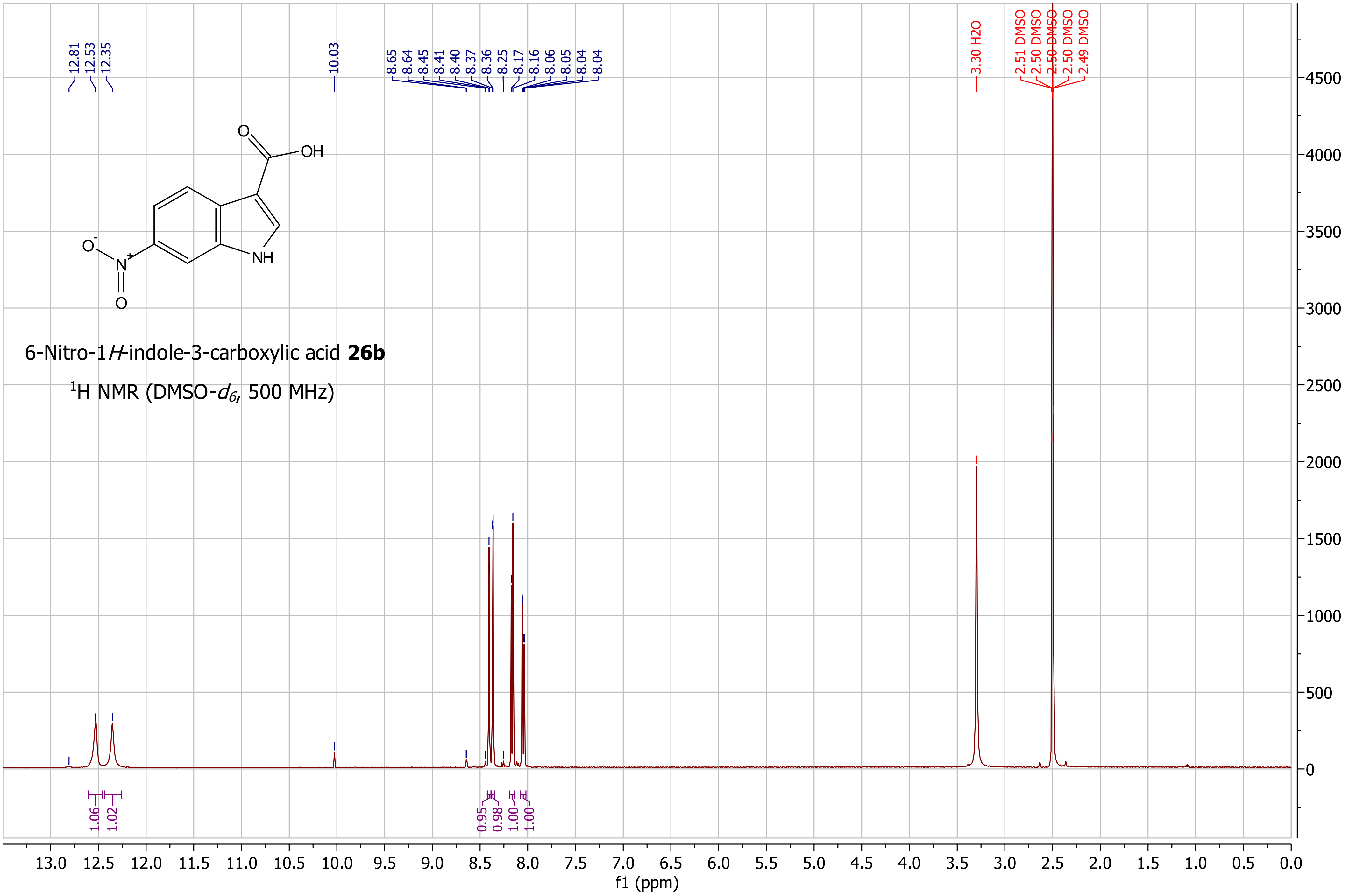

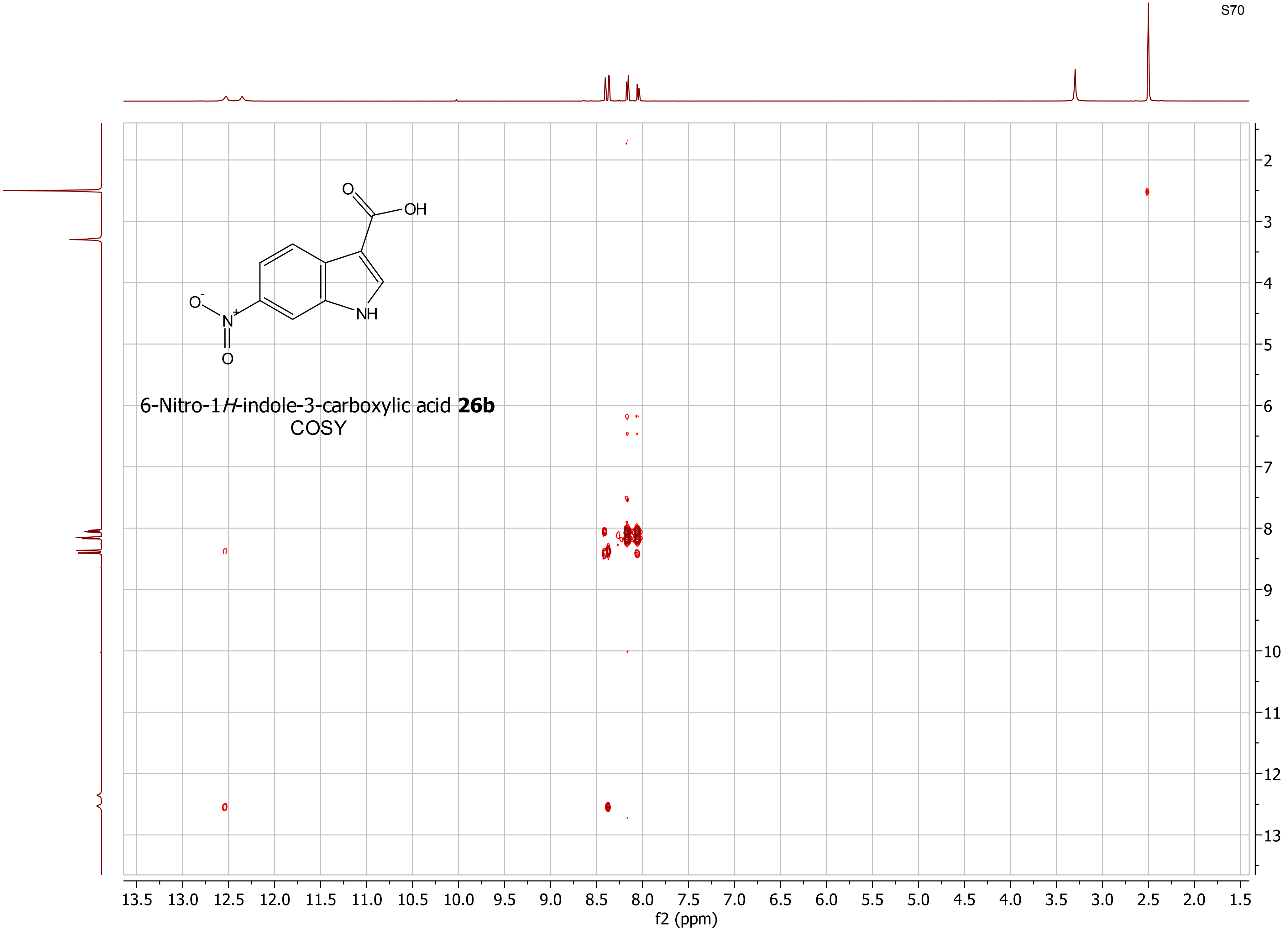
6-Nitro-1H-indole-3-carboxylic acid 26b
COSY
f2 (ppm)
f1 (ppm)

^{13}C NMR (126 MHz, DMSO-d_6) δ 165.1, 142.6, 137.7, 135.0, 130.8, 120.8, 116.0, 108.9, 108.3.

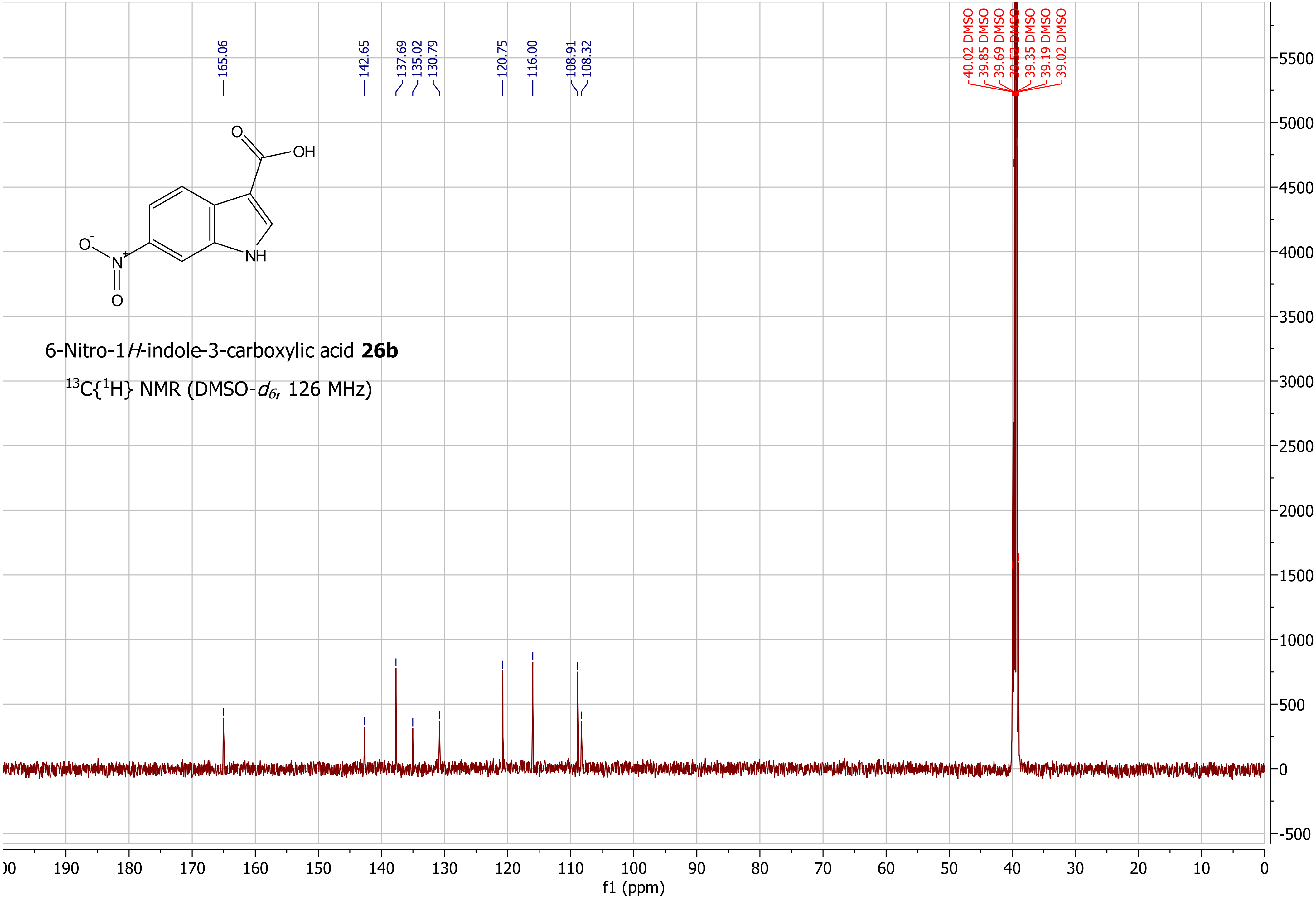

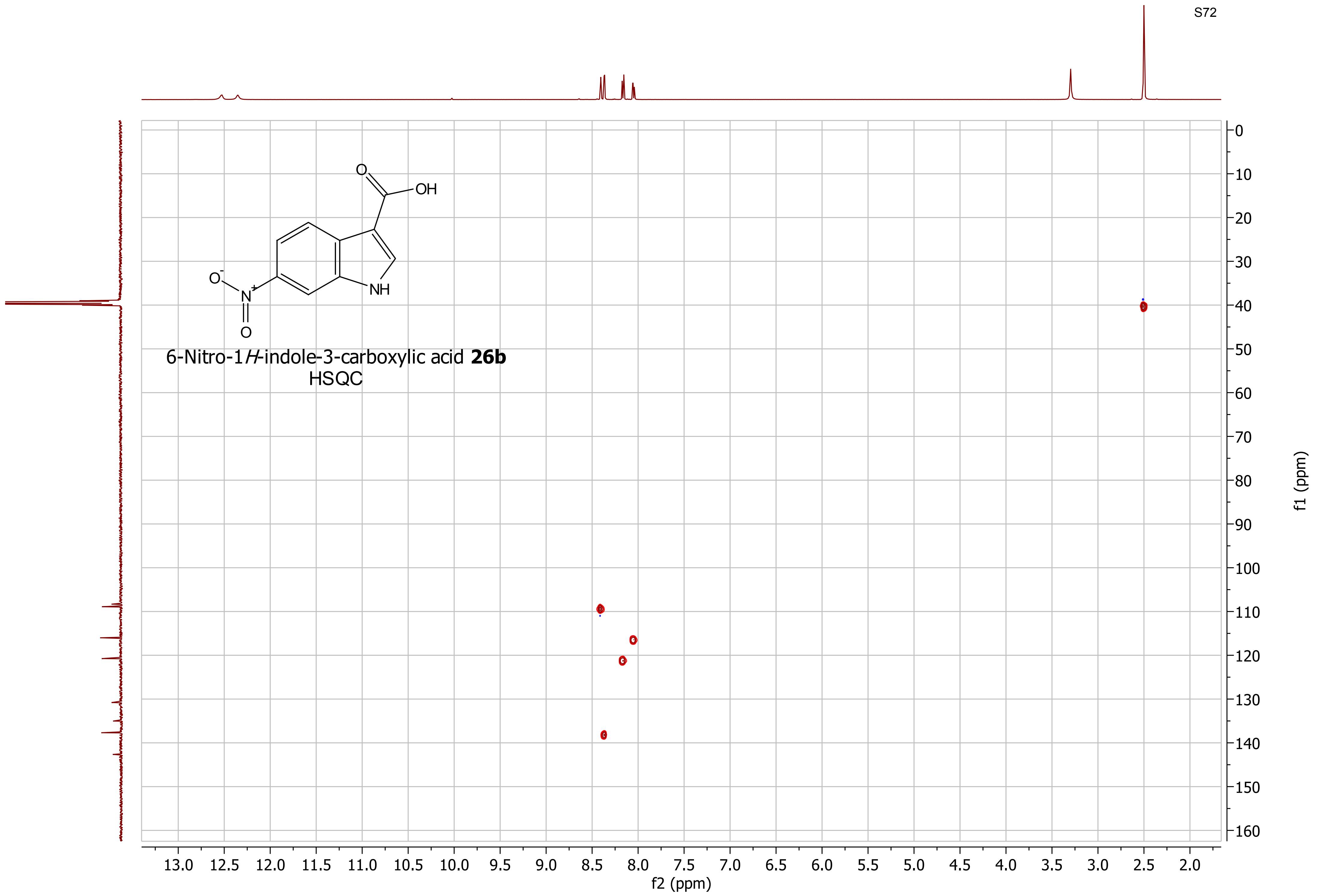
6-Nitro-1*H*-indole-3-carboxylic acid **26b**
HSQC
O
OH
NH
O-
N+
O
f1 (ppm)
f2 (ppm)
13.0 12.5 12.0 11.5 11.0 10.5 10.0 9.5 9.0 8.5 8.0 7.5 7.0 6.5 6.0 5.5 5.0 4.5 4.0 3.5 3.0 2.5 2.0
0 10 20 30 40 50 60 70 80 90 100 110 120 130 140 150 160

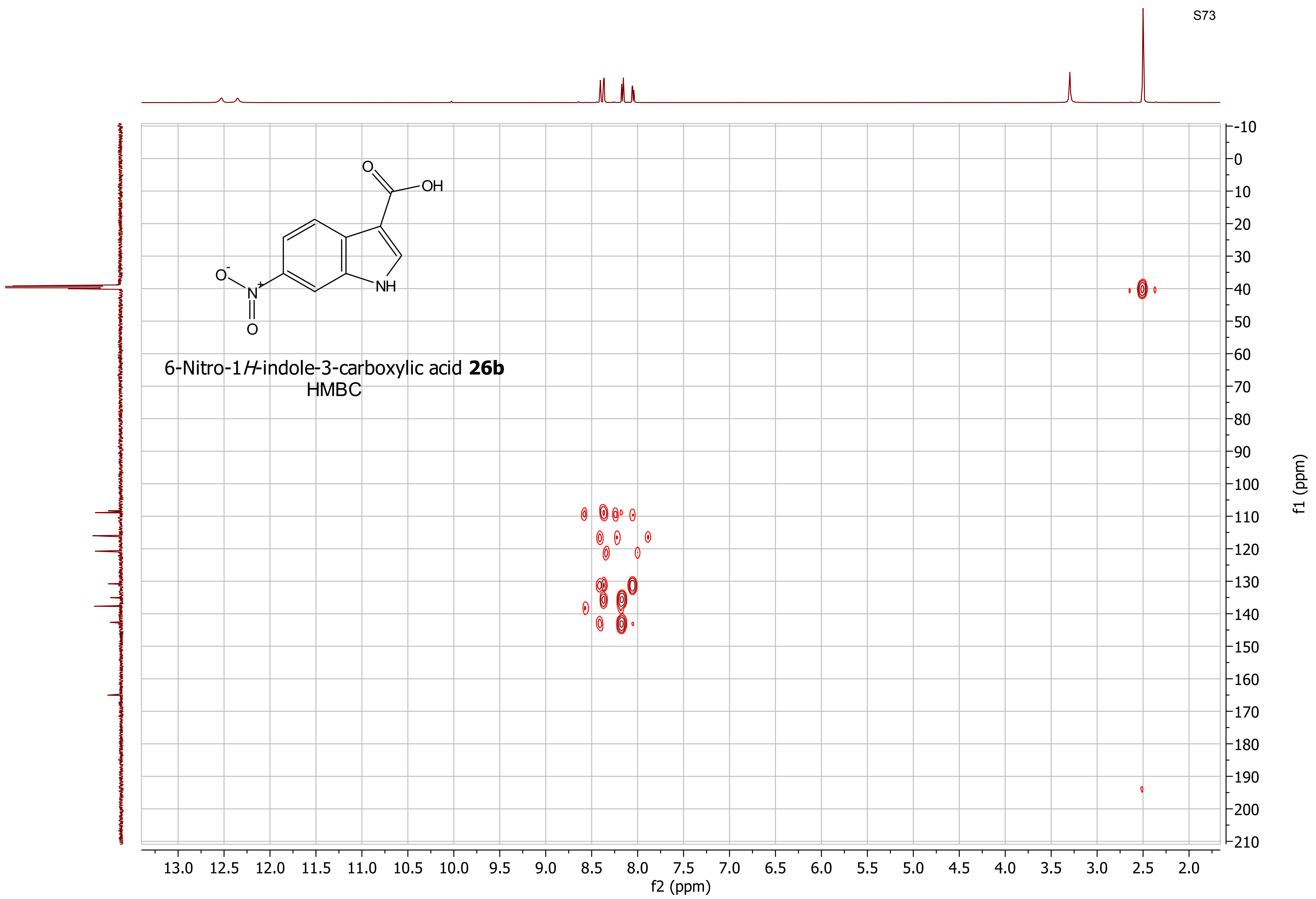
O
OH
O-
N+
O
NH
6-Nitro-1H-indole-3-carboxylic acid 26b
HMBC
f2 (ppm)
f1 (ppm)

^{1}H NMR (500 MHz, DMSO-d_6) δ 12.44 (s, 2H), 8.50 (d, J = 7.7 Hz, 1H), 8.18 (d, J = 7.9 Hz, 1H), 8.07 (d, J = 2.9 Hz, 1H), 7.41 (t, J = 7.9 Hz, 1H).

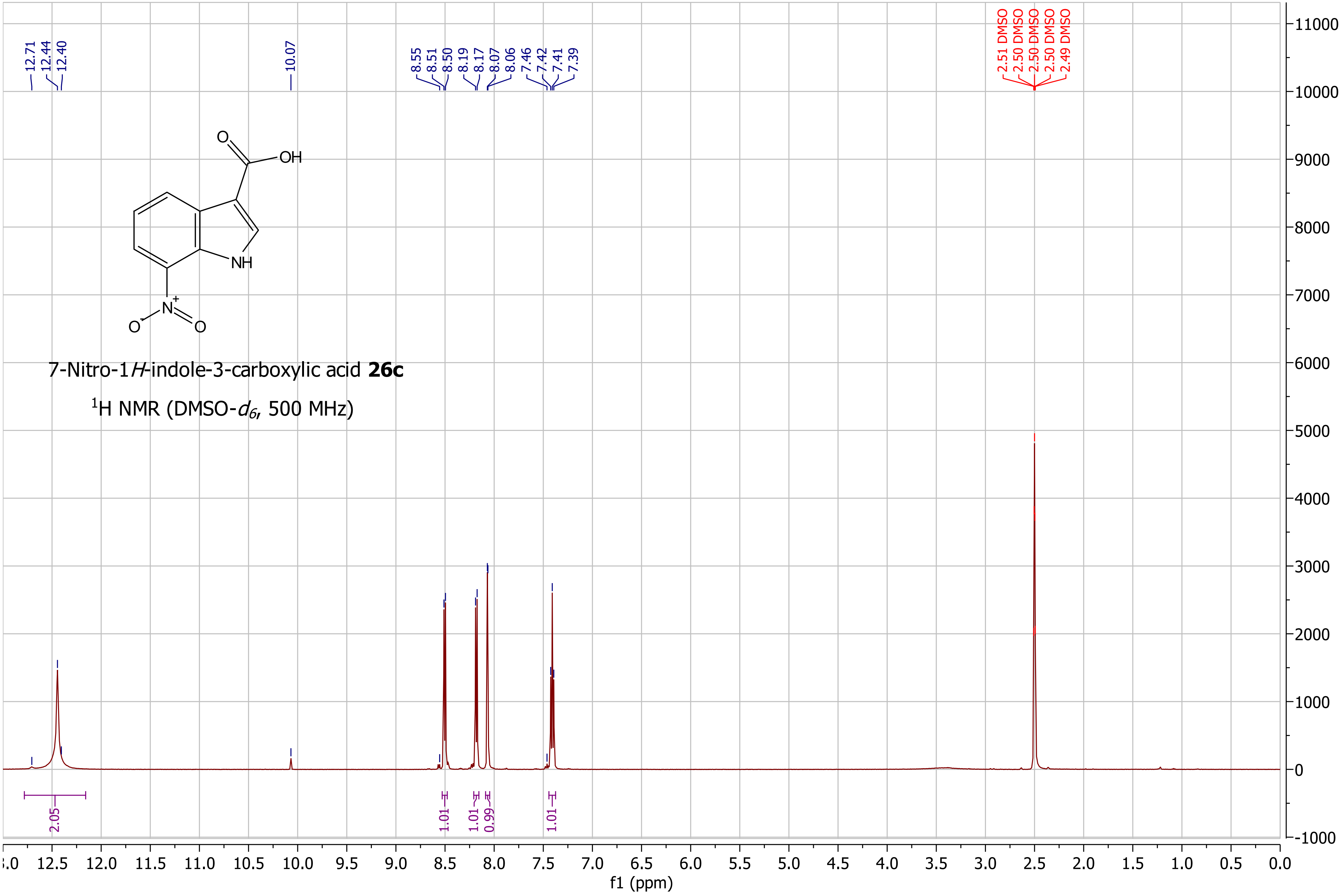

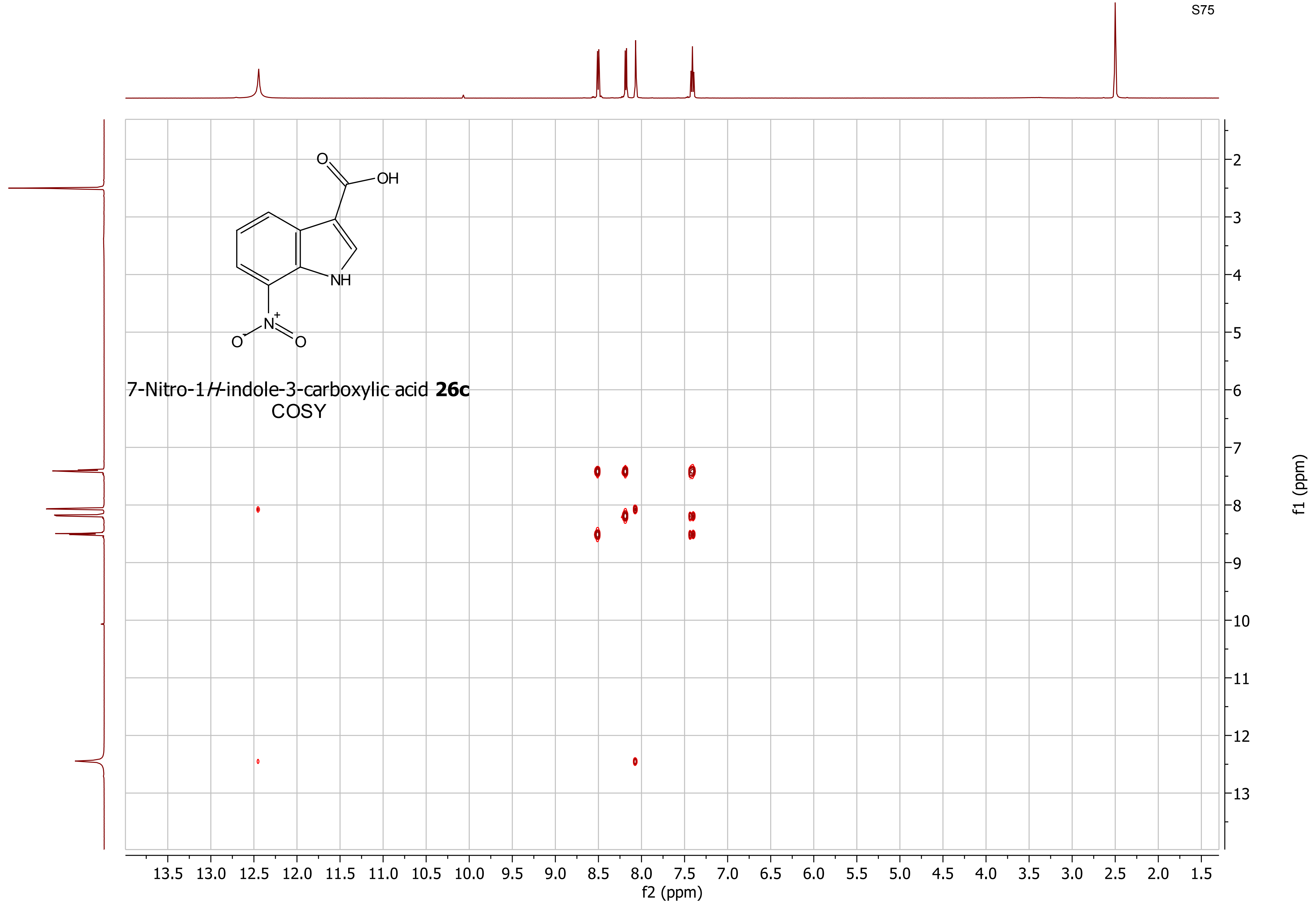

O
OH
NH
N+
O-
O
7-Nitro-1*H*-indole-3-carboxylic acid **26c**
COSY
13.5 13.0 12.5 12.0 11.5 11.0 10.5 10.0 9.5 9.0 8.5 8.0 7.5 7.0 6.5 6.0 5.5 5.0 4.5 4.0 3.5 3.0 2.5 2.0 1.5
f2 (ppm)
2 3 4 5 6 7 8 9 10 11 12 13
f1 (ppm)

^{13}C NMR (126 MHz, DMSO-d_6) δ 164.9, 134.6, 133.2, 129.6, 128.8, 128.4, 120.9, 119.3, 109.0.

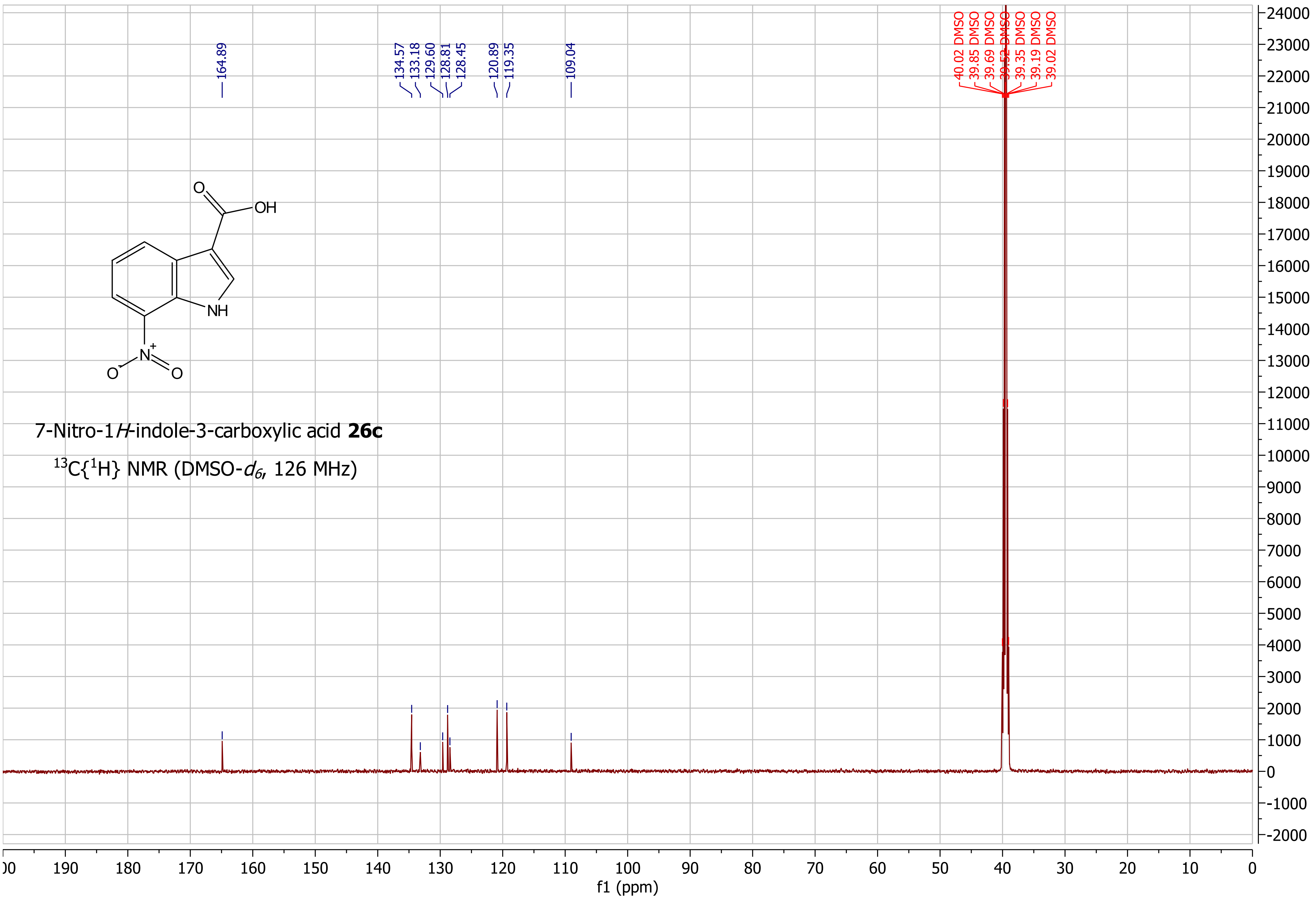

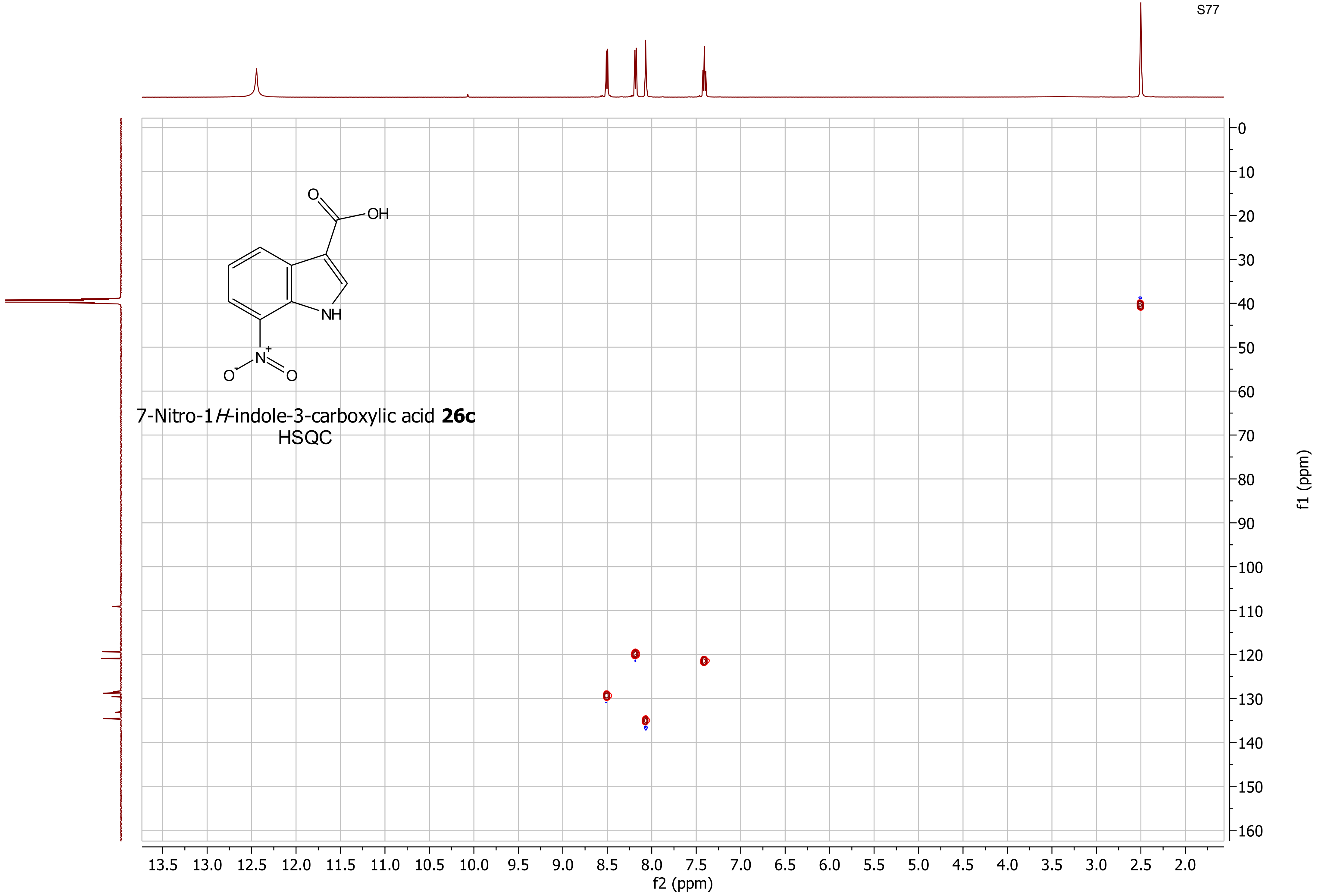

7-Nitro-1*H*-indole-3-carboxylic acid **26c**
HSQC

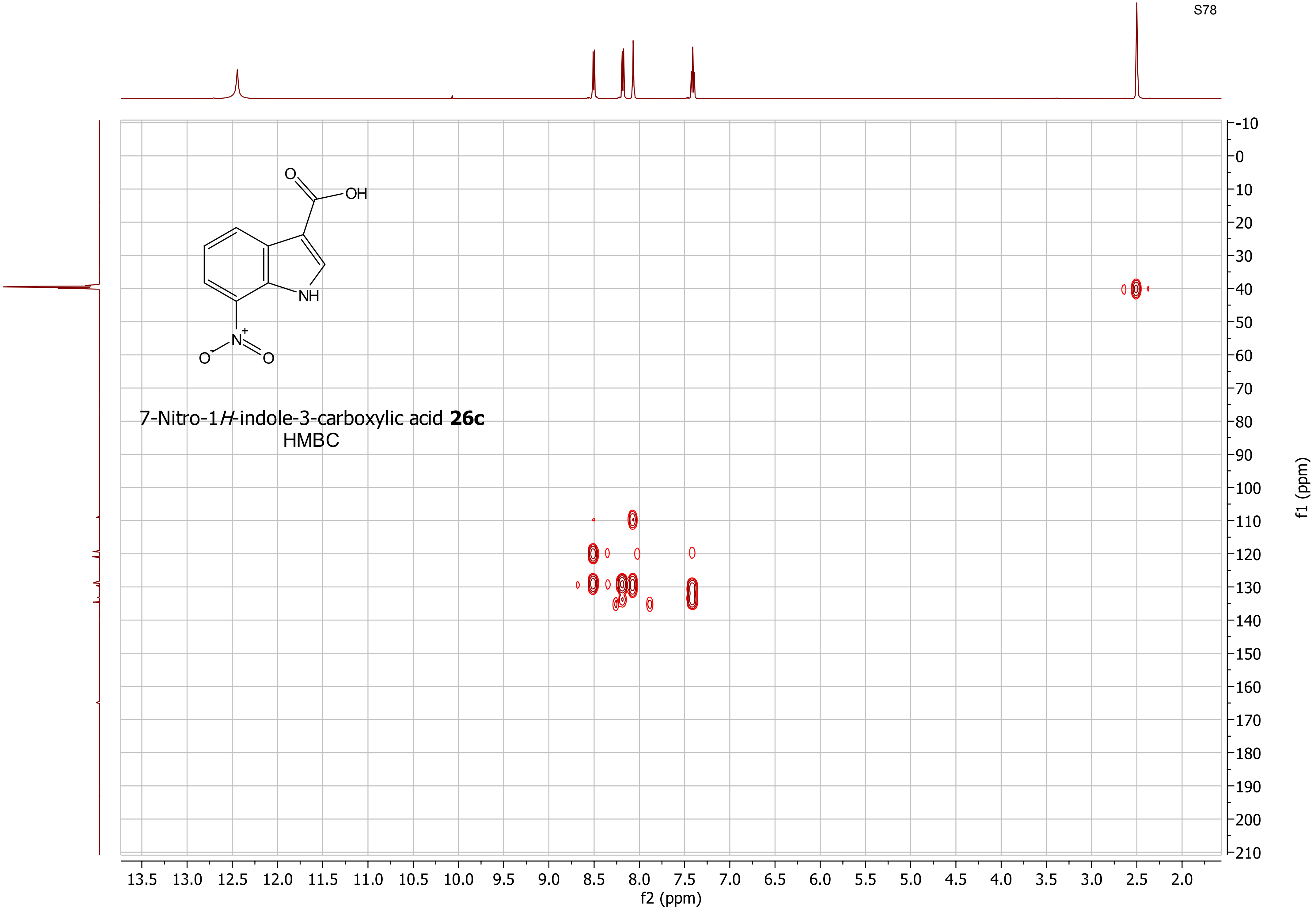
7-Nitro-1*H*-indole-3-carboxylic acid **26c**
HMBC
O
OH
NH
N+
O-
O
f2 (ppm)
f1 (ppm)
13.5 13.0 12.5 12.0 11.5 11.0 10.5 10.0 9.5 9.0 8.5 8.0 7.5 7.0 6.5 6.0 5.5 5.0 4.5 4.0 3.5 3.0 2.5 2.0
-10 0 10 20 30 40 50 60 70 80 90 100 110 120 130 140 150 160 170 180 190 200 210

^{1}H NMR (500 MHz, DMSO-d_6) δ 12.62 (s, 1H), 8.88 (d, J = 2.3 Hz, 1H), 8.43 (s, 1H), 8.11 (dd, J = 9.0, 2.4 Hz, 1H), 7.69 (d, J = 8.9 Hz, 1H), 5.47 (s, 2H), 3.49 (s, 3H).

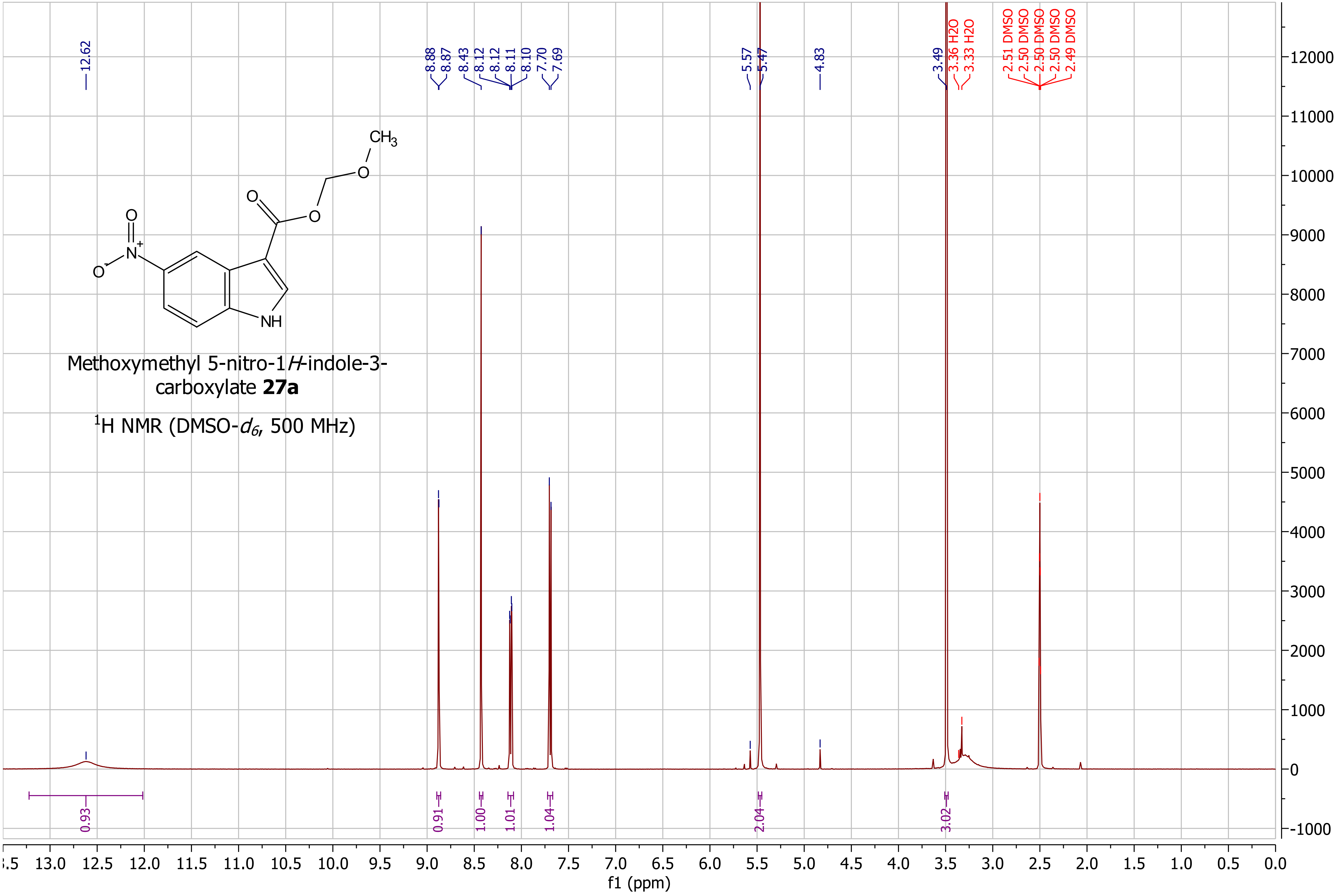

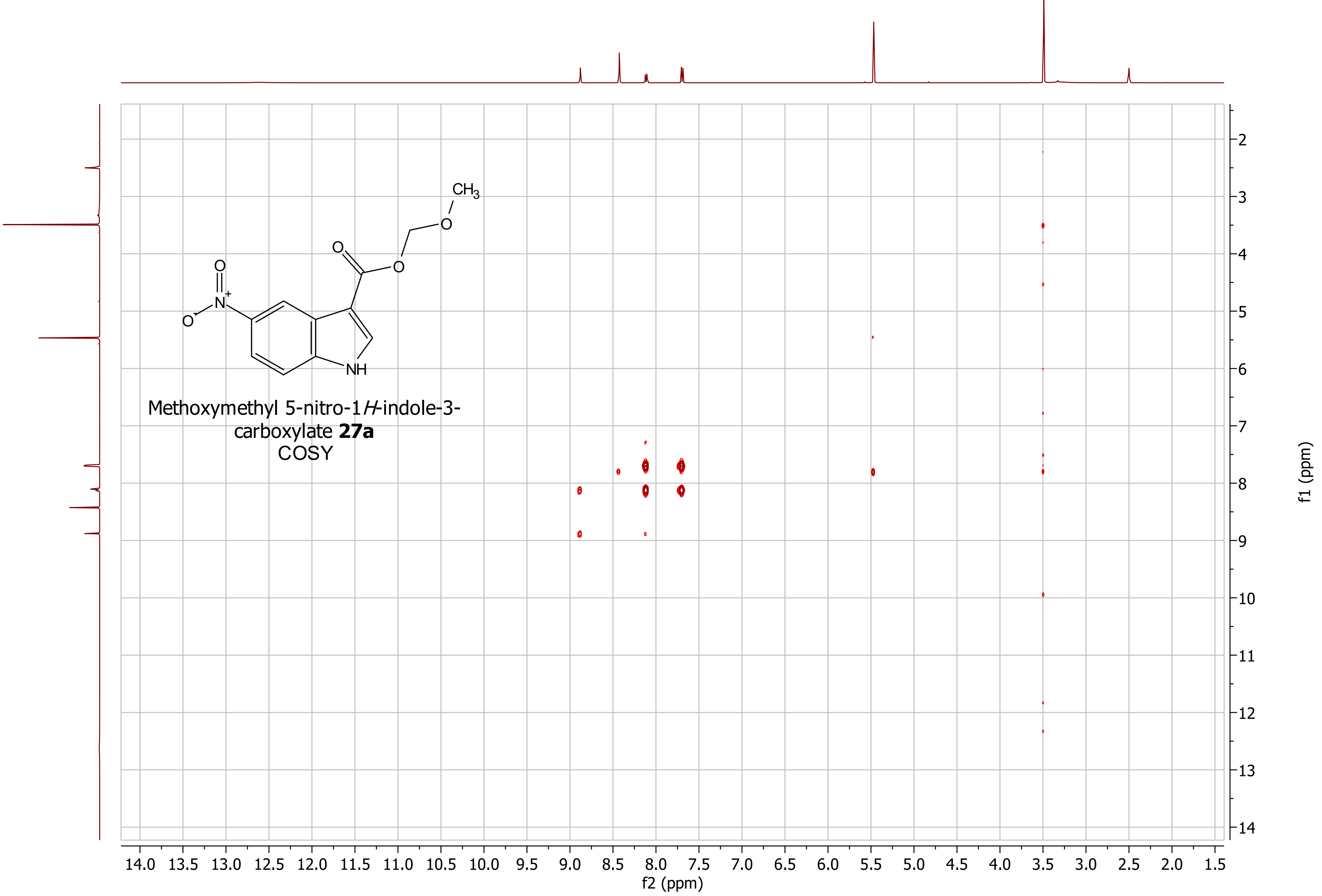

CH3
O
O
O
O
N+
O-
NH
Methoxymethyl 5-nitro-1H-indole-3-carboxylate 27a
COSY
f2 (ppm)
f1 (ppm)
14.0 13.5 13.0 12.5 12.0 11.5 11.0 10.5 10.0 9.5 9.0 8.5 8.0 7.5 7.0 6.5 6.0 5.5 5.0 4.5 4.0 3.5 3.0 2.5 2.0 1.5
2 3 4 5 6 7 8 9 10 11 12 13 14

^{13}C NMR (126 MHz, DMSO-d_6) δ 162.9, 142.5, 139.6, 136.7, 125.0, 117.8, 116.8, 113.2, 108.0, 89.4, 56.9.

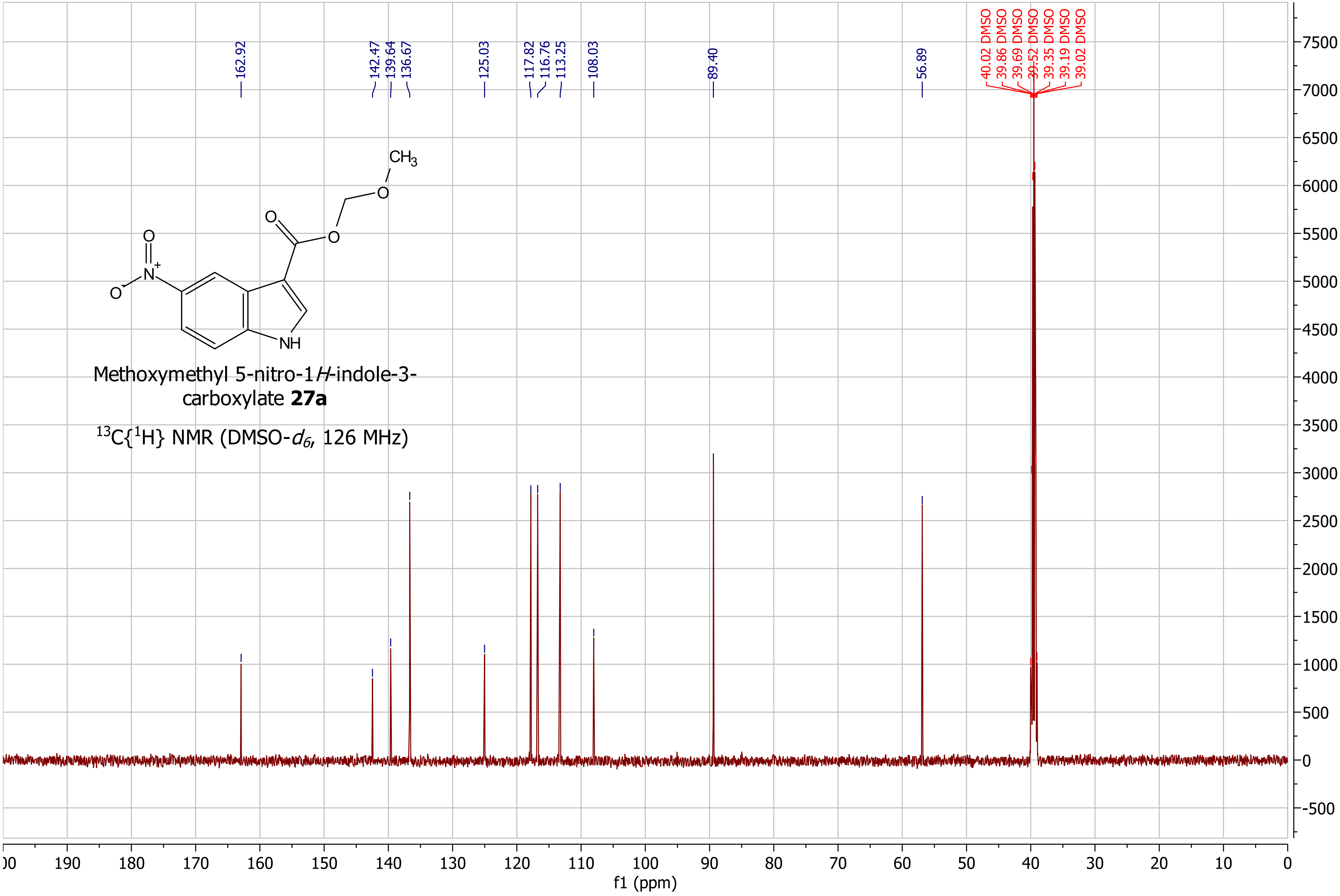

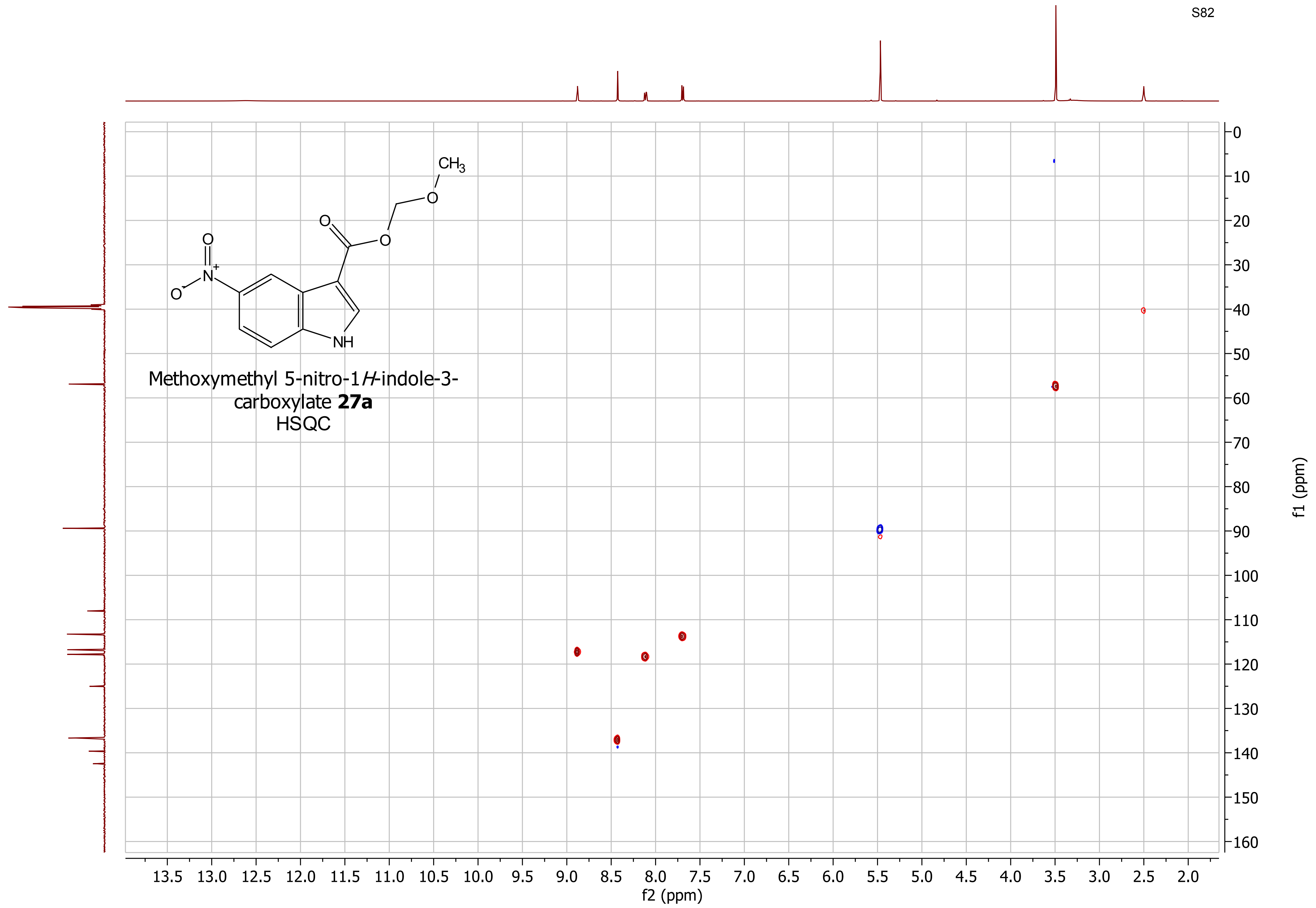

CH3
O
O
O
O
N+
O-
NH
Methoxymethyl 5-nitro-1H-indole-3-carboxylate 27a
HSQC
13.5 13.0 12.5 12.0 11.5 11.0 10.5 10.0 9.5 9.0 8.5 8.0 7.5 7.0 6.5 6.0 5.5 5.0 4.5 4.0 3.5 3.0 2.5 2.0
f2 (ppm)
0 10 20 30 40 50 60 70 80 90 100 110 120 130 140 150 160
f1 (ppm)

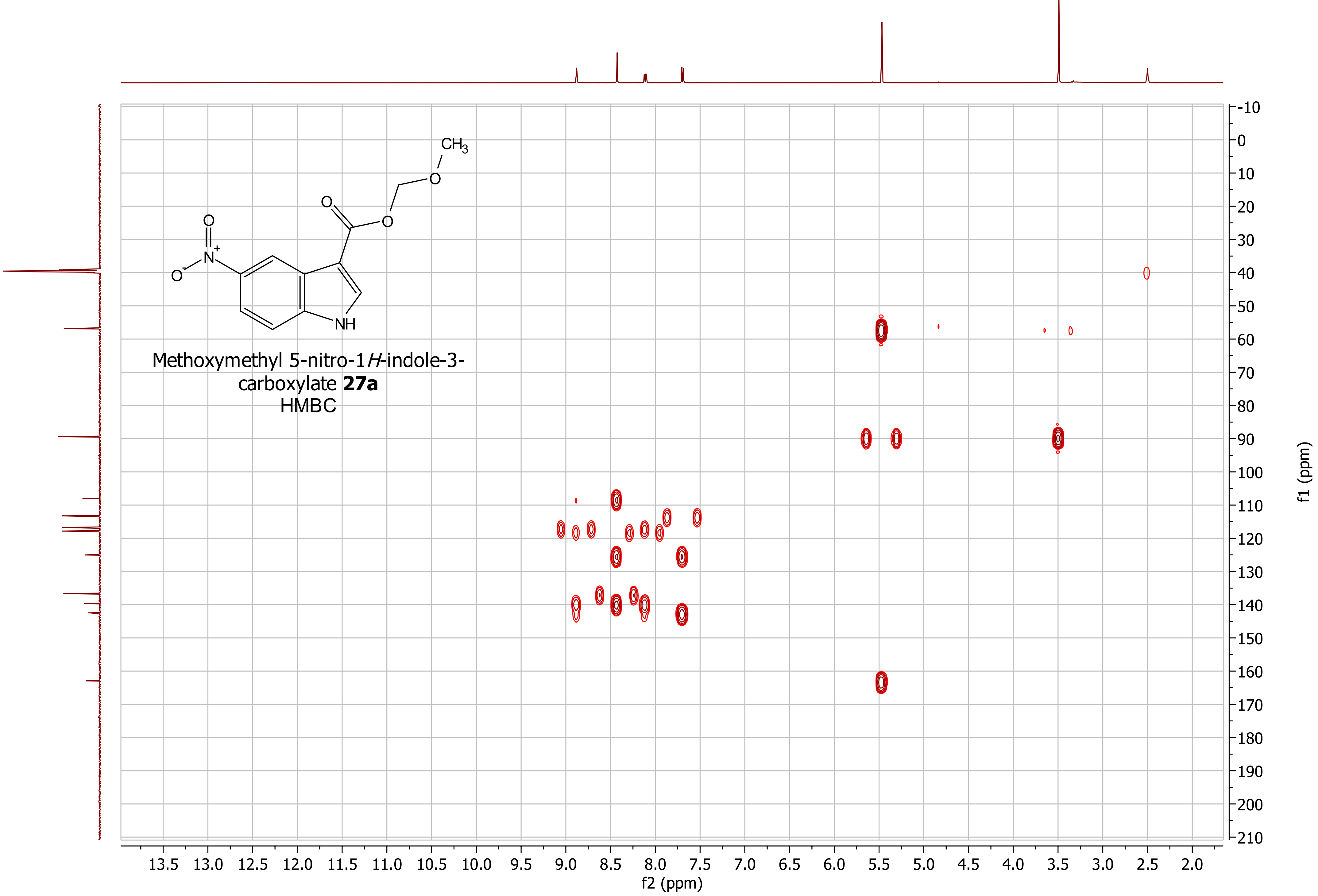

Methoxymethyl 5-nitro-1*H*-indole-3-carboxylate **27a**
HMBC
f2 (ppm)
f1 (ppm)

^{1}H NMR (500 MHz, DMSO-d_6) δ 12.63 (s, 1H), 8.52 (s, 1H), 8.42 (d, J = 2.1 Hz, 1H), 8.17 (d, J = 8.8 Hz, 1H), 8.09 (dd, J = 8.8, 2.1 Hz, 1H), 5.45 (s, 2H), 3.48 (s, 3H).

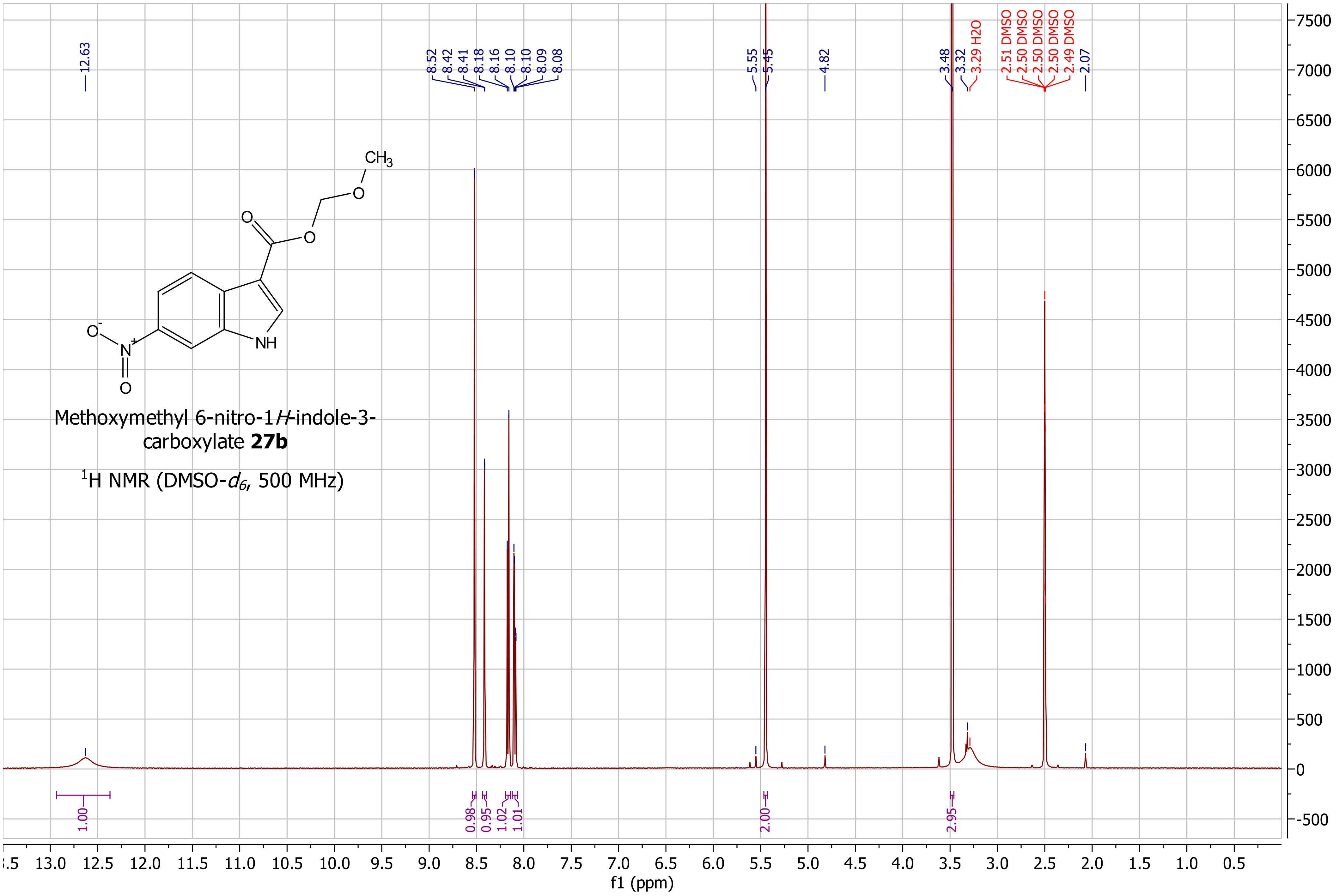

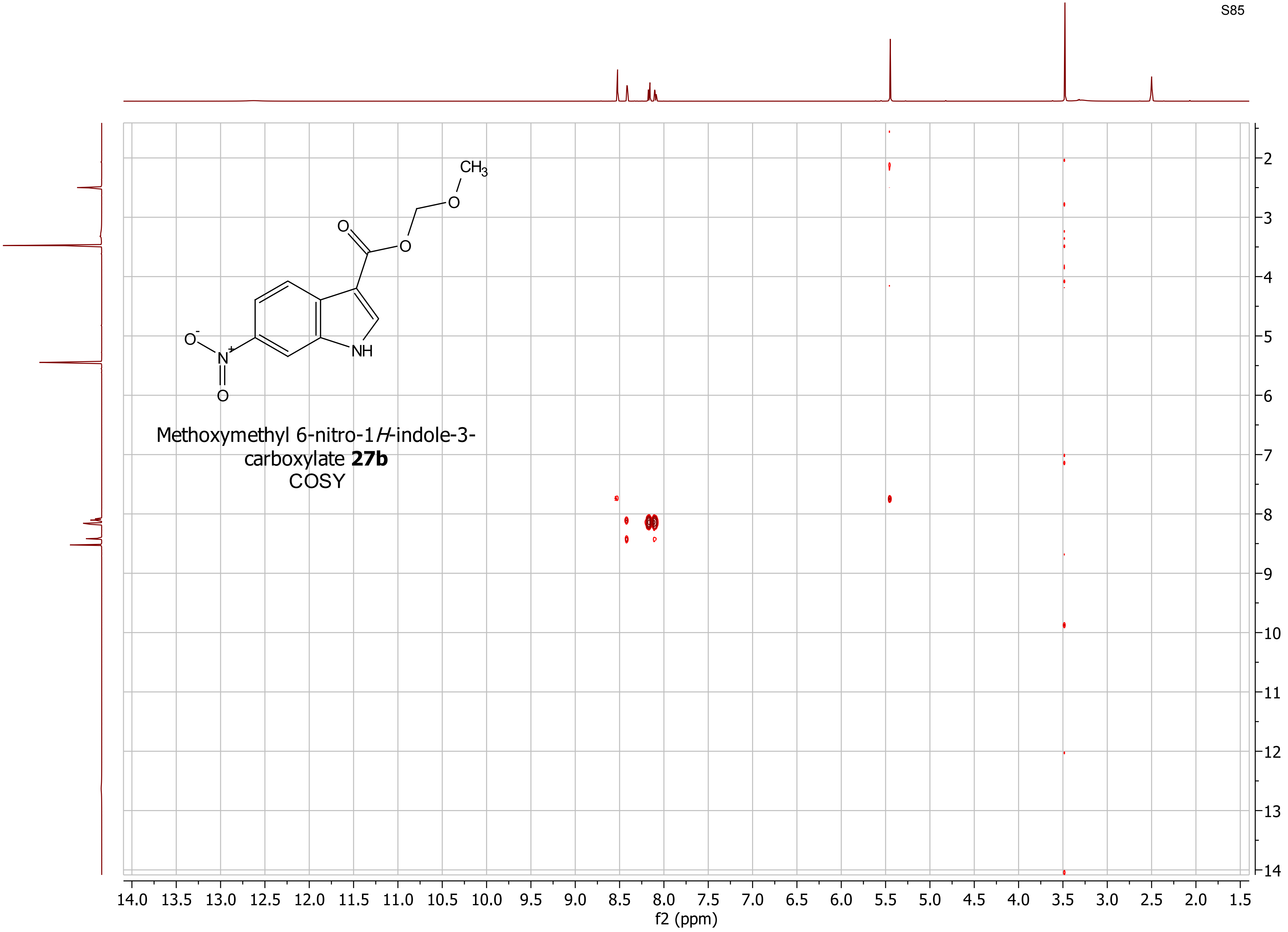

Methoxymethyl 6-nitro-1*H*-indole-3-carboxylate **27b**
COSY

^{13}C NMR (126 MHz, DMSO-d_6) δ 162.9, 142.9, 138.5, 135.2, 130.5, 120.6, 116.5, 109.1, 106.9, 89.3, 56.9.

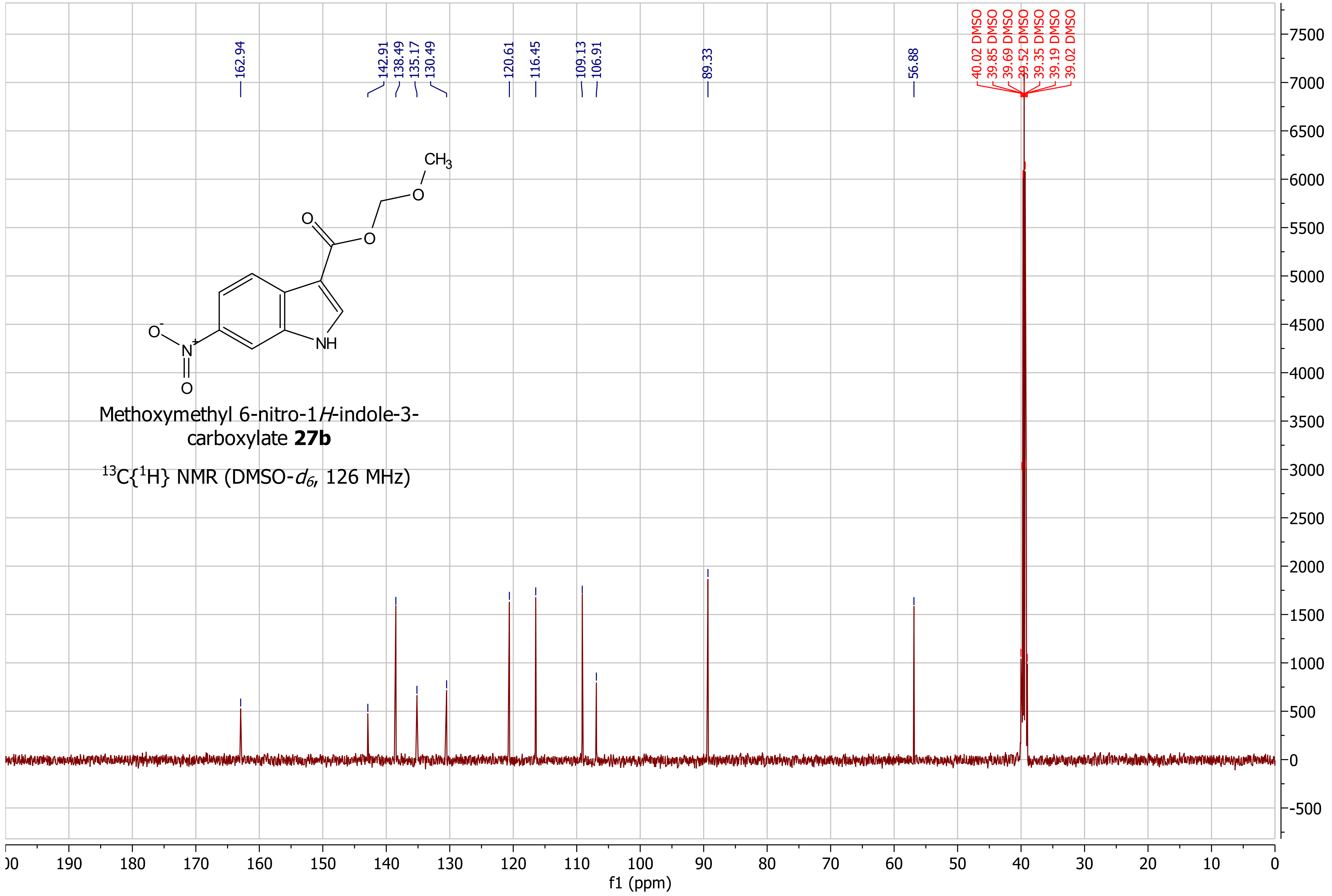

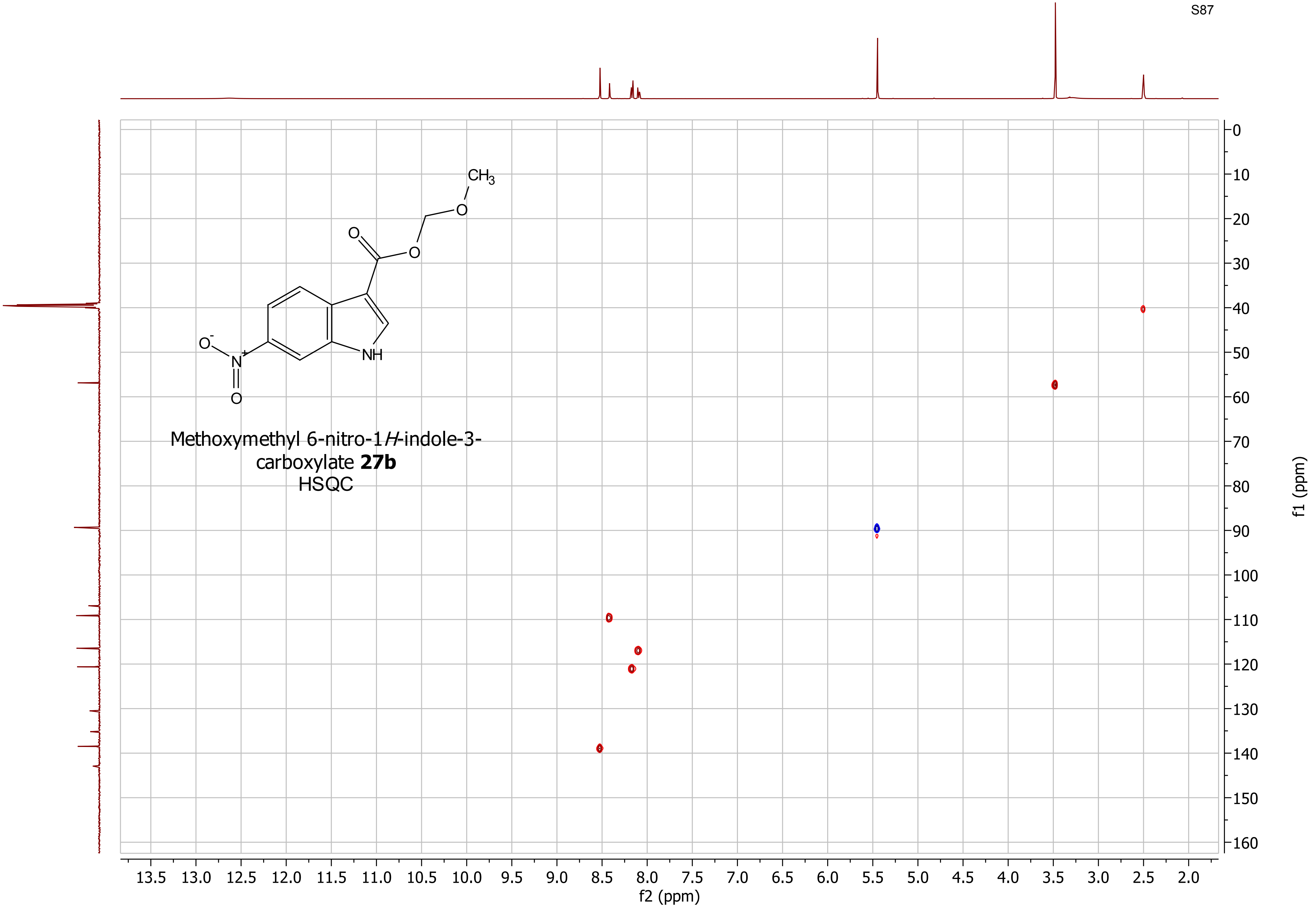
Methoxymethyl 6-nitro-1*H*-indole-3-carboxylate **27b**
HSQC
f2 (ppm)
f1 (ppm)

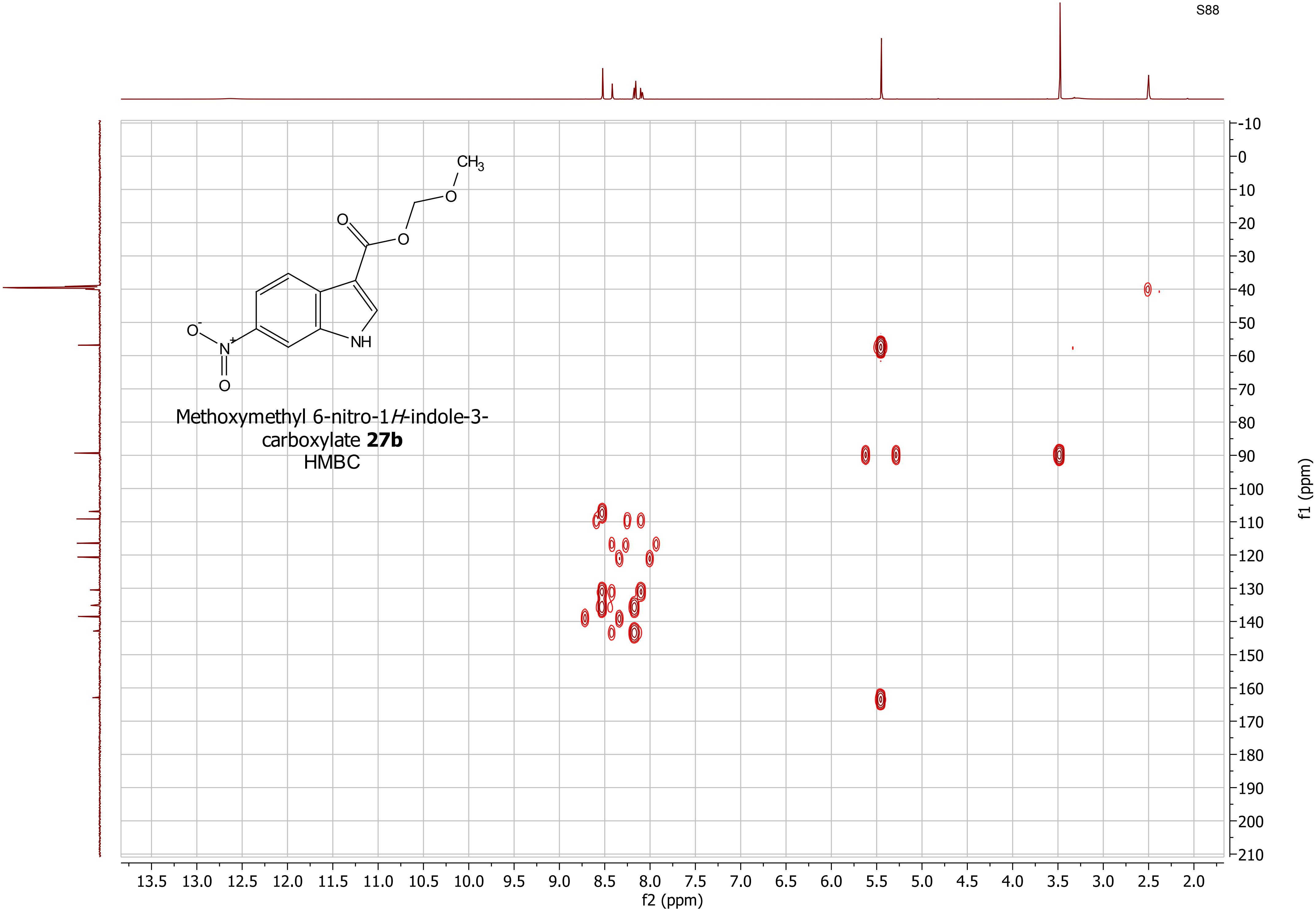
Methoxymethyl 6-nitro-1*H*-indole-3-carboxylate **27b**
HMBC
f2 (ppm)
f1 (ppm)

^{1}H NMR (500 MHz, DMSO-d_6) δ 12.61 (s, 1H), 8.49 (dd, J = 7.9, 1.0 Hz, 1H), 8.21 (d, J = 8.0 Hz, 1H), 8.20 (s, 1H), 7.46 (t, J = 8.0 Hz, 1H), 5.45 (s, 2H), 3.48 (s, 3H).

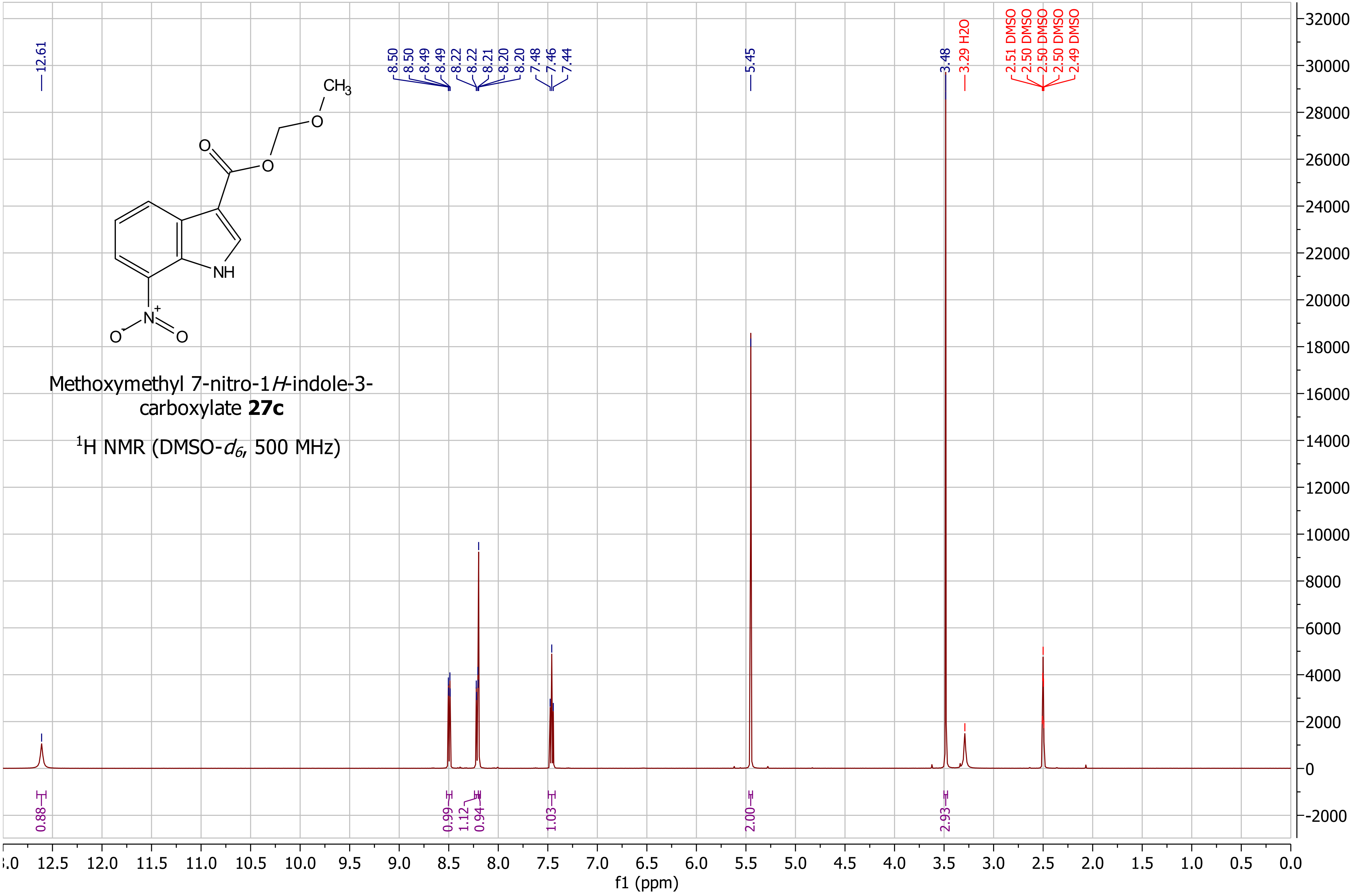

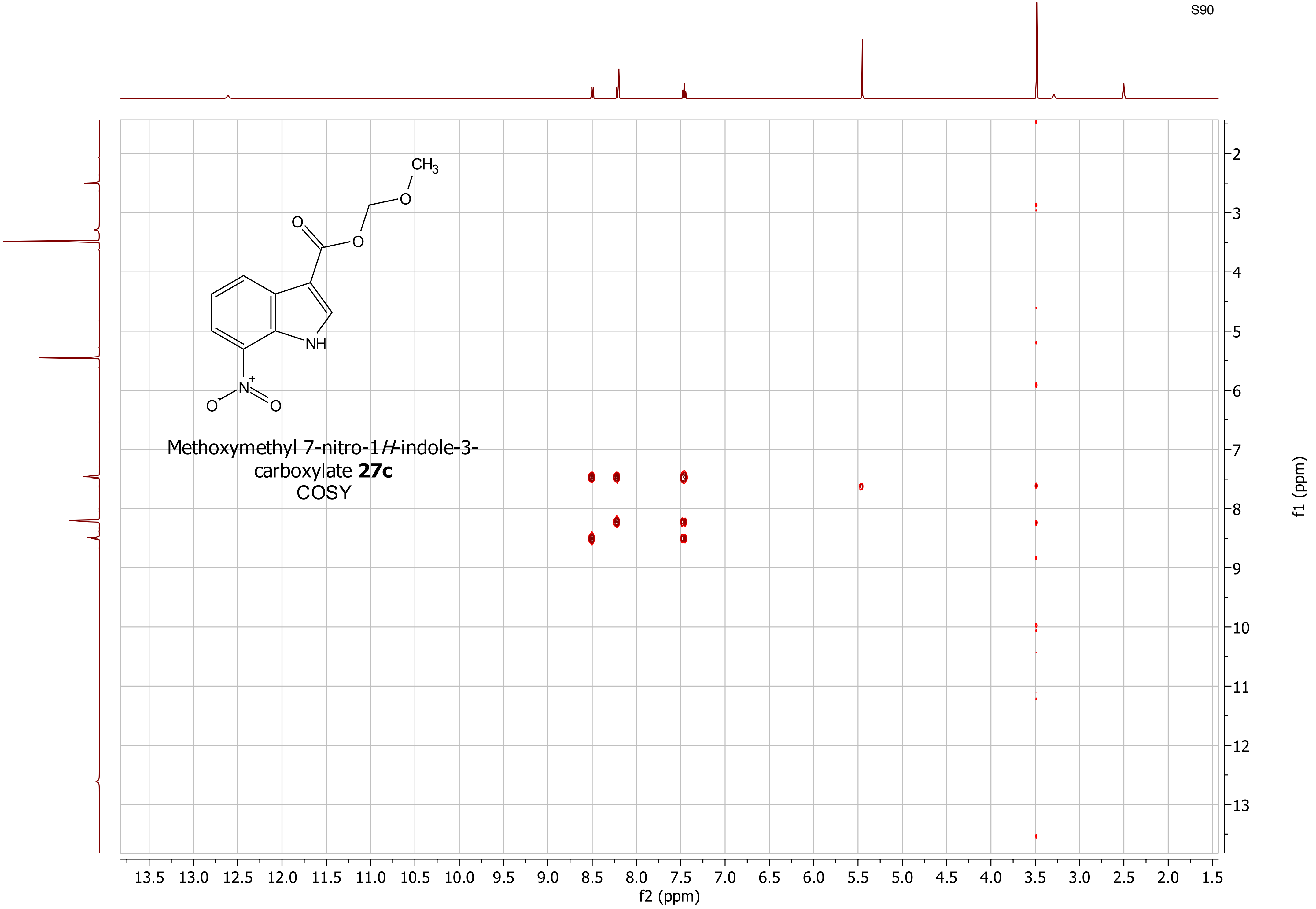
CH3
O
O
O
NH
N+
O-
O
Methoxymethyl 7-nitro-1H-indole-3-carboxylate 27c
COSY
f2 (ppm)
f1 (ppm)

^{13}C NMR (126 MHz, DMSO-d_6) δ 162.9, 135.4, 133.4, 129.3, 128.6, 121.4, 119.7, 107.7, 89.4, 56.9.

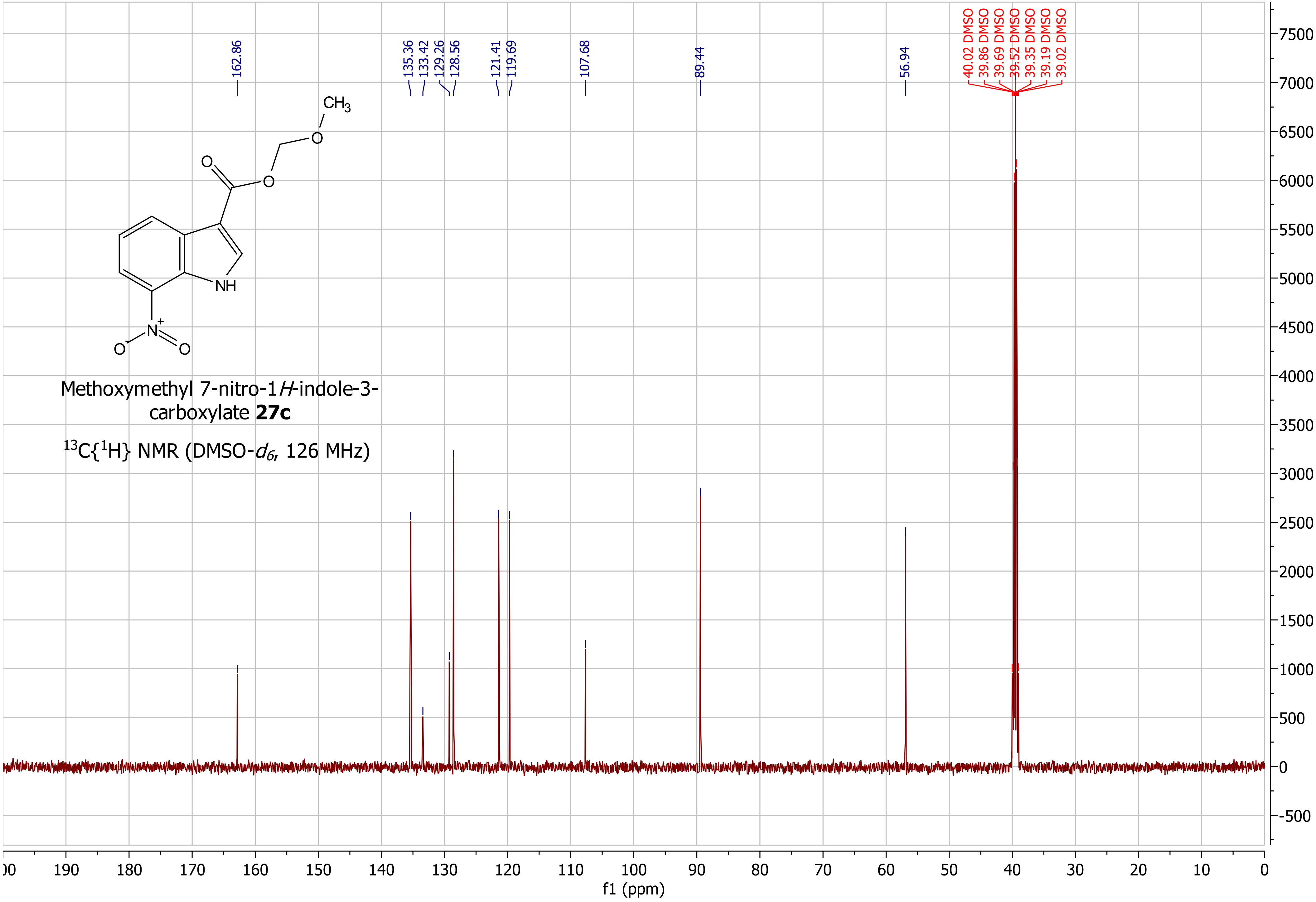

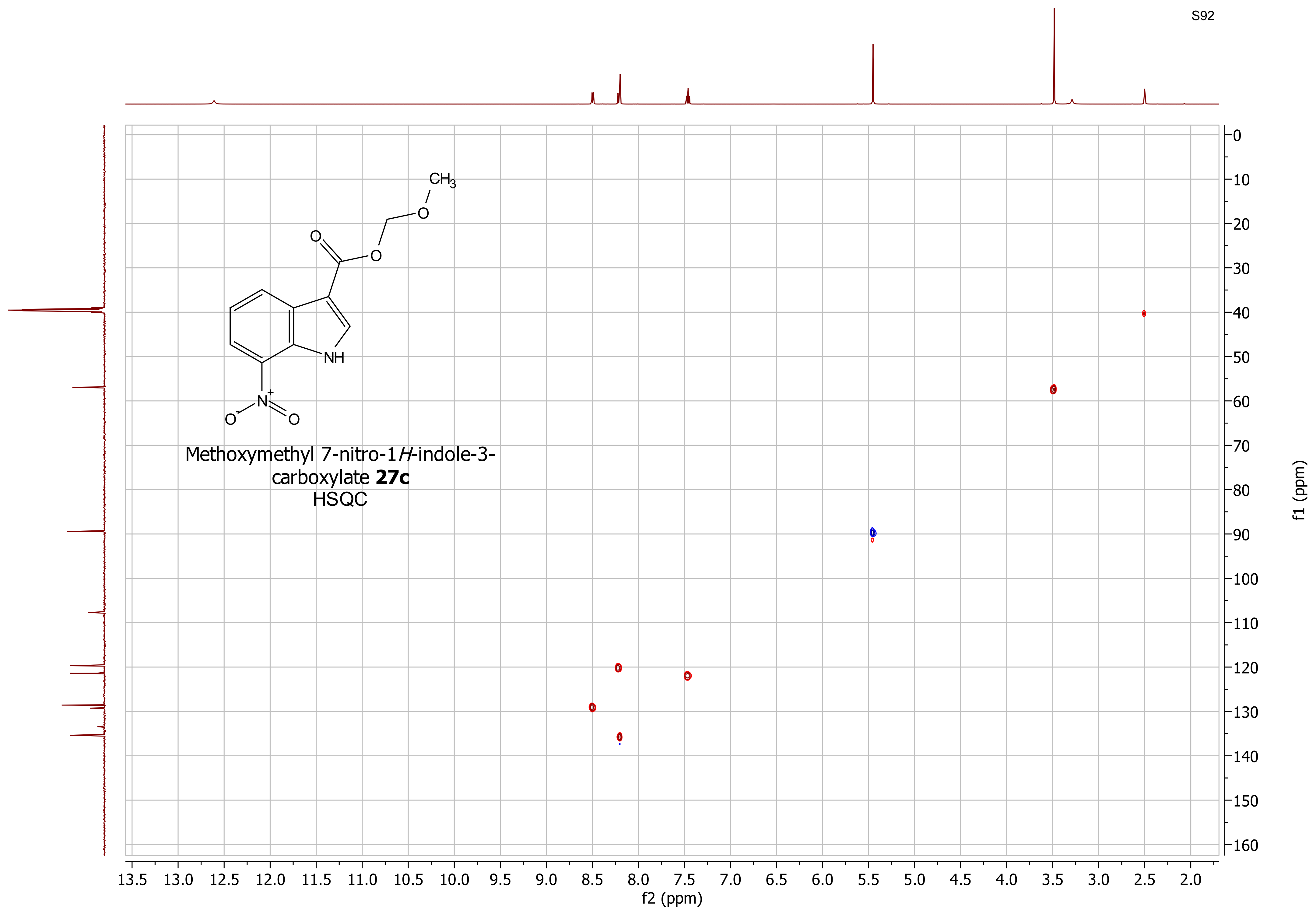

Methoxymethyl 7-nitro-1*H*-indole-3-carboxylate **27c**
HSQC
f2 (ppm)
f1 (ppm)

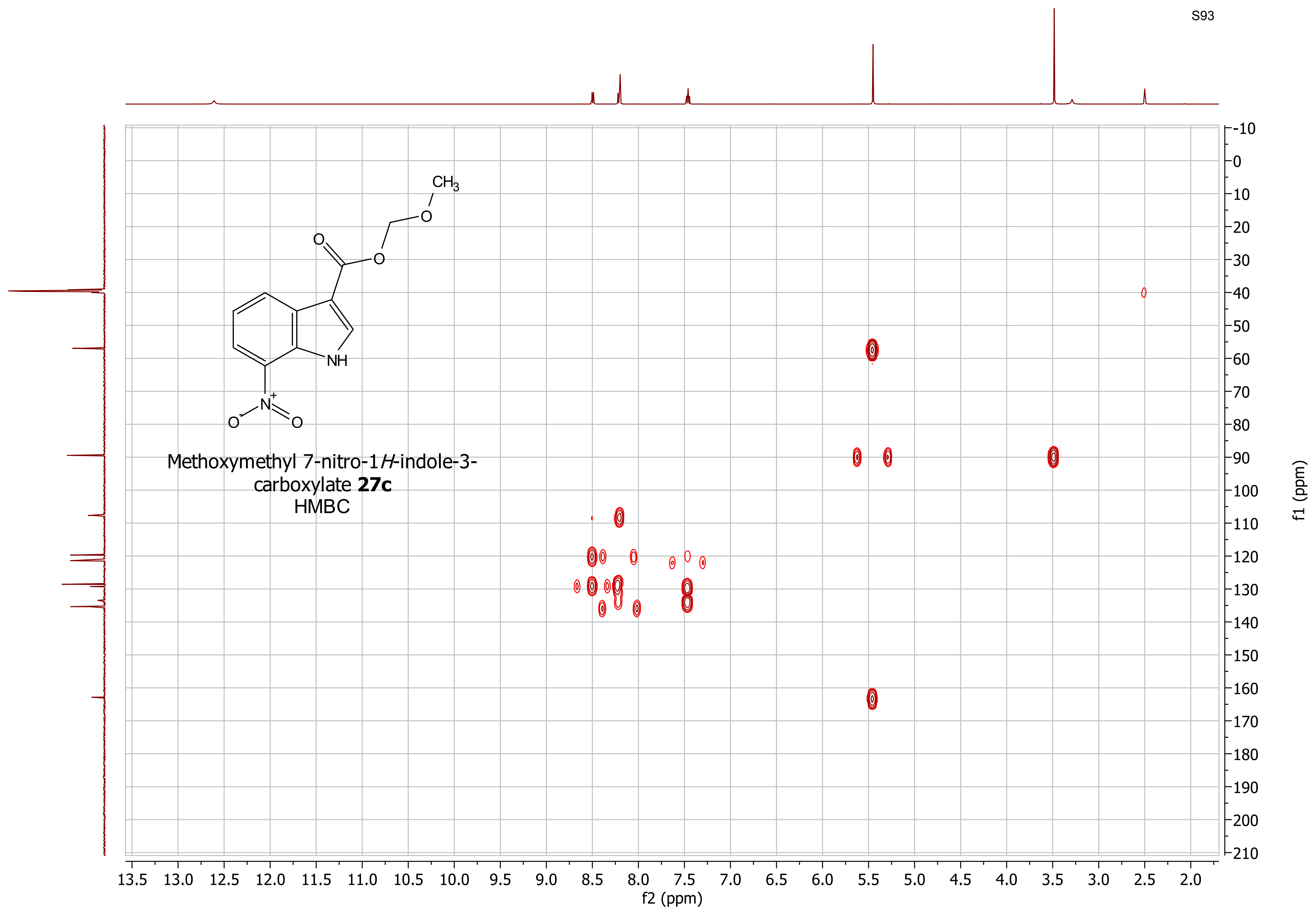
Methoxymethyl 7-nitro-1H-indole-3-carboxylate 27c
HMBC
f2 (ppm)
f1 (ppm)

^{1}H NMR (500 MHz, DMSO-d_6) δ 11.55 (s, 1H), 7.89 (s, 1H), 7.19 (d, J = 2.2 Hz, 1H), 7.16 (d, J = 8.5 Hz, 1H), 6.57 (dd, J = 8.6, 2.2 Hz, 1H), 5.36 (s, 2H), 4.75 (s, 2H), 3.44 (s, 3H).

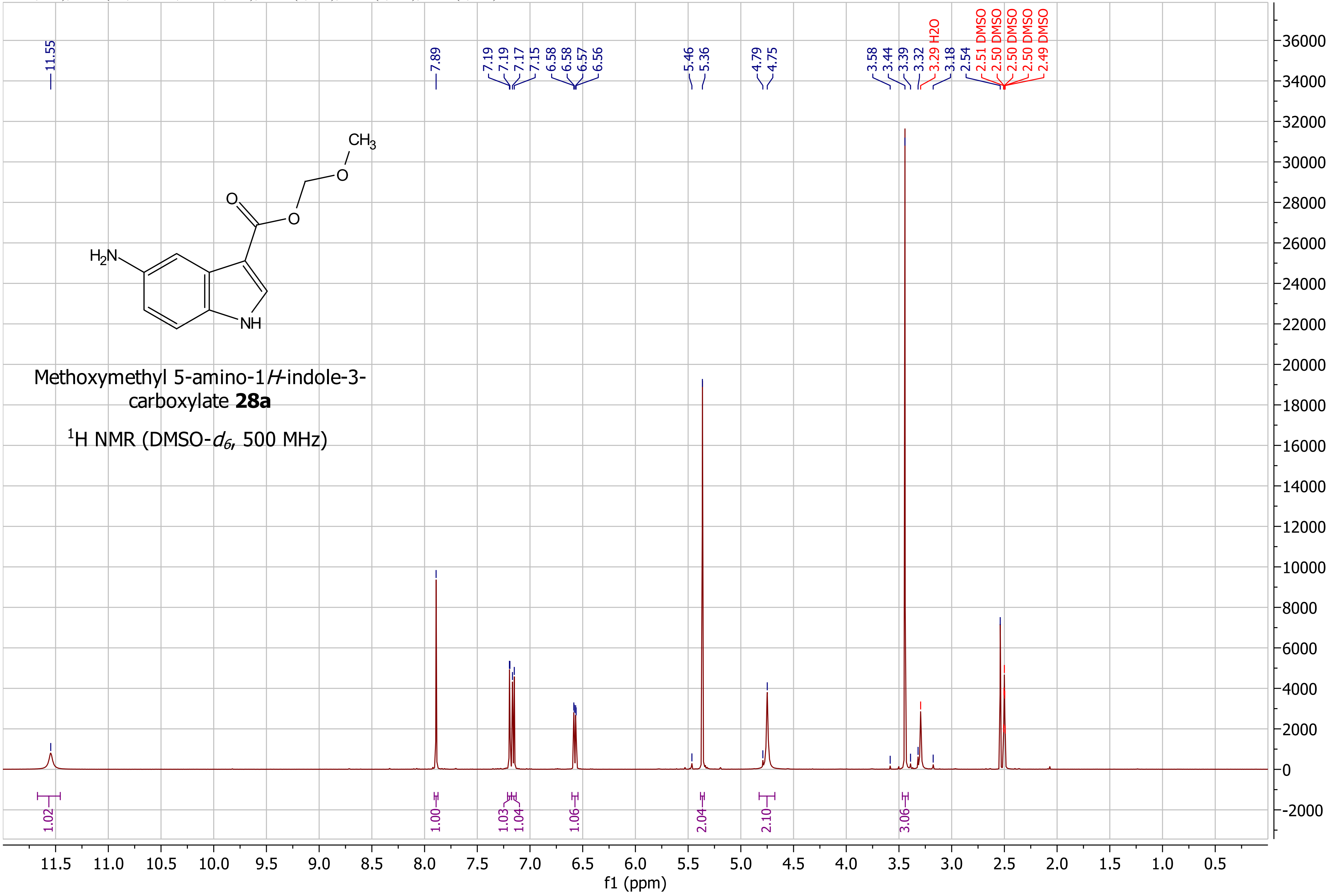

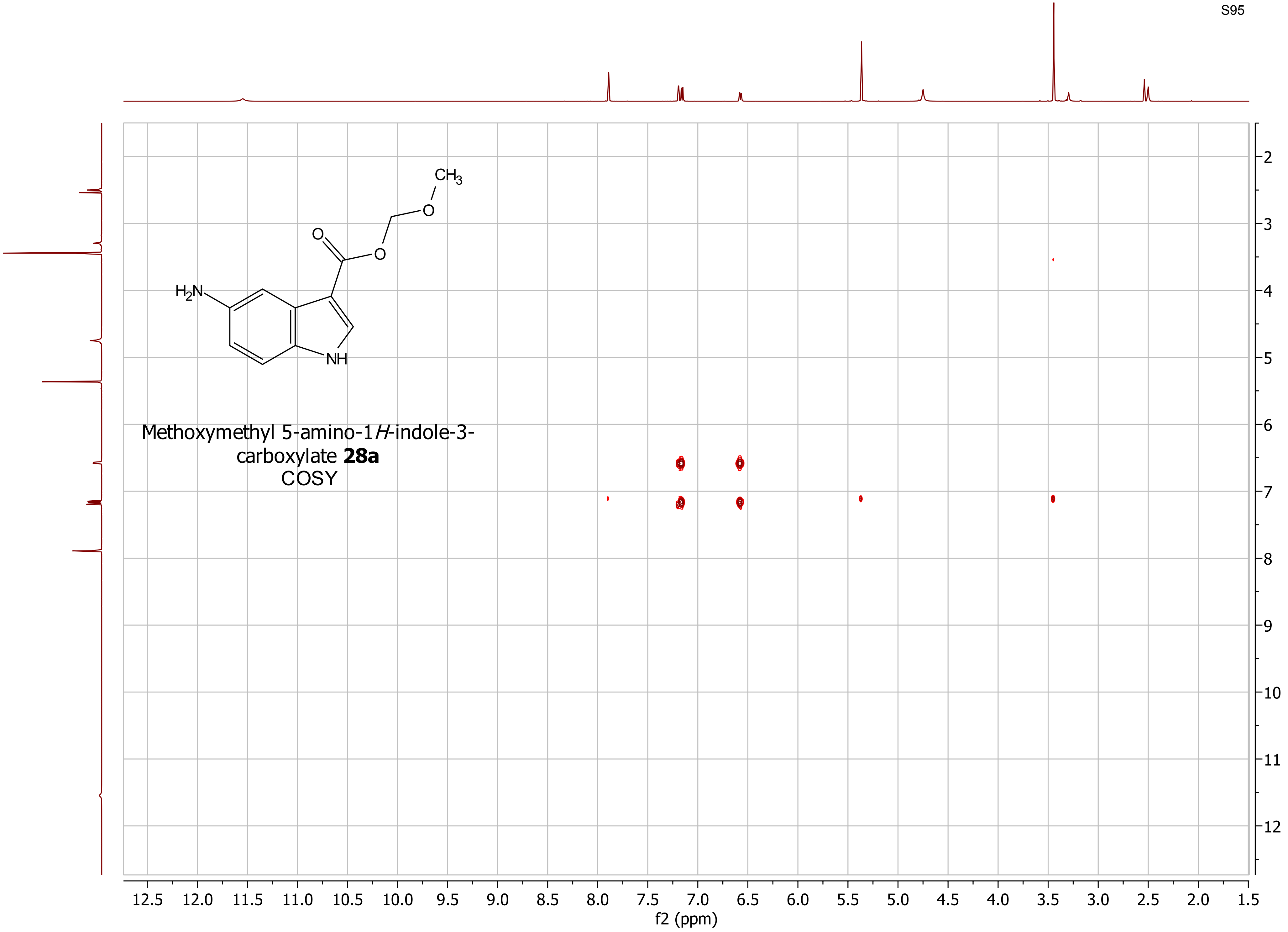
CH3
O
O
O
H2N
NH
Methoxymethyl 5-amino-1H-indole-3-carboxylate 28a
COSY
f1 (ppm)
f2 (ppm)
12.5 12.0 11.5 11.0 10.5 10.0 9.5 9.0 8.5 8.0 7.5 7.0 6.5 6.0 5.5 5.0 4.5 4.0 3.5 3.0 2.5 2.0 1.5
2 3 4 5 6 7 8 9 10 11 12

^{13}C NMR (126 MHz, DMSO-d_6) δ 163.8, 143.6, 131.9, 129.6, 127.1, 112.6, 112.4, 104.5, 103.4, 88.4, 56.6.

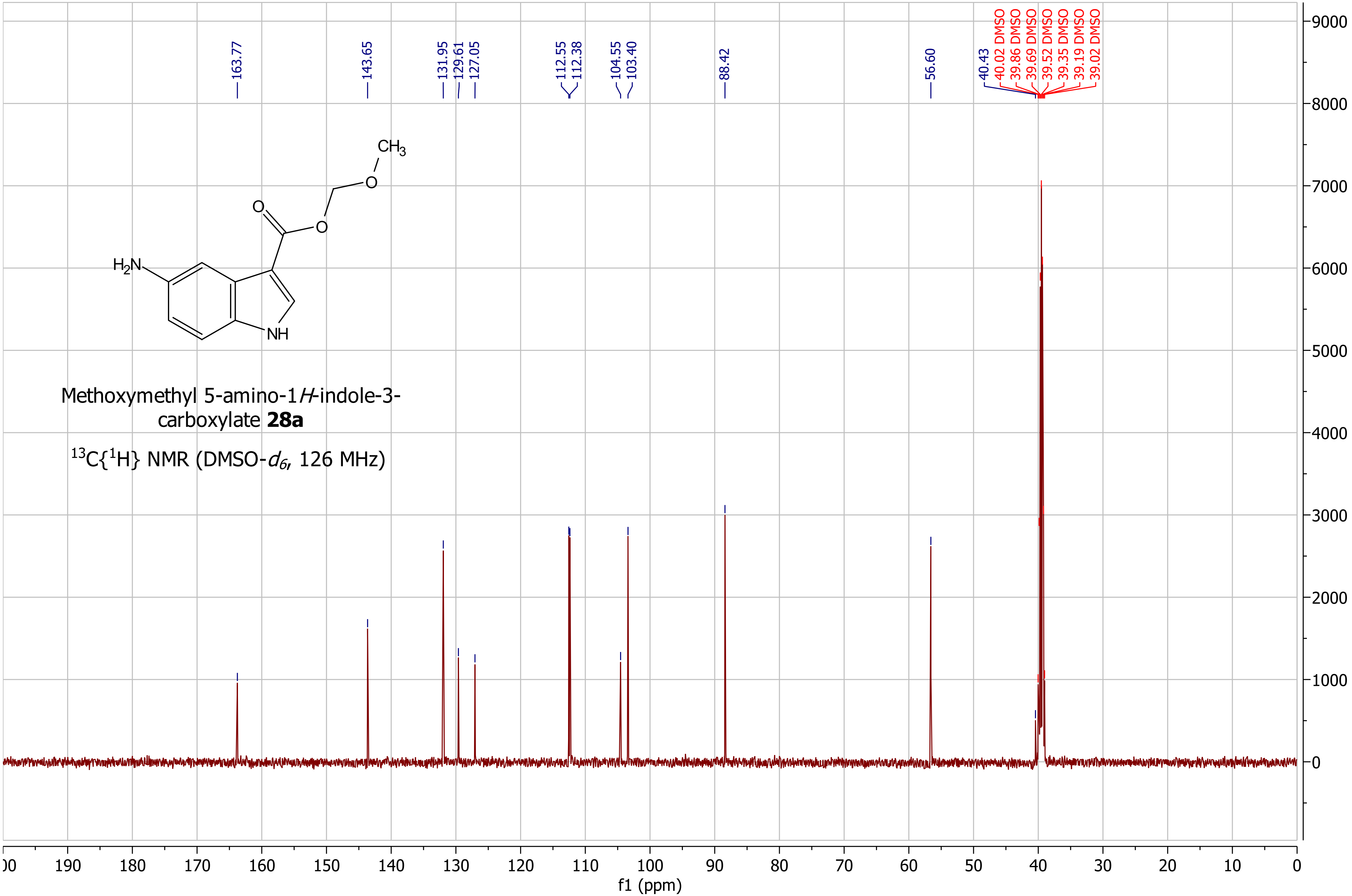

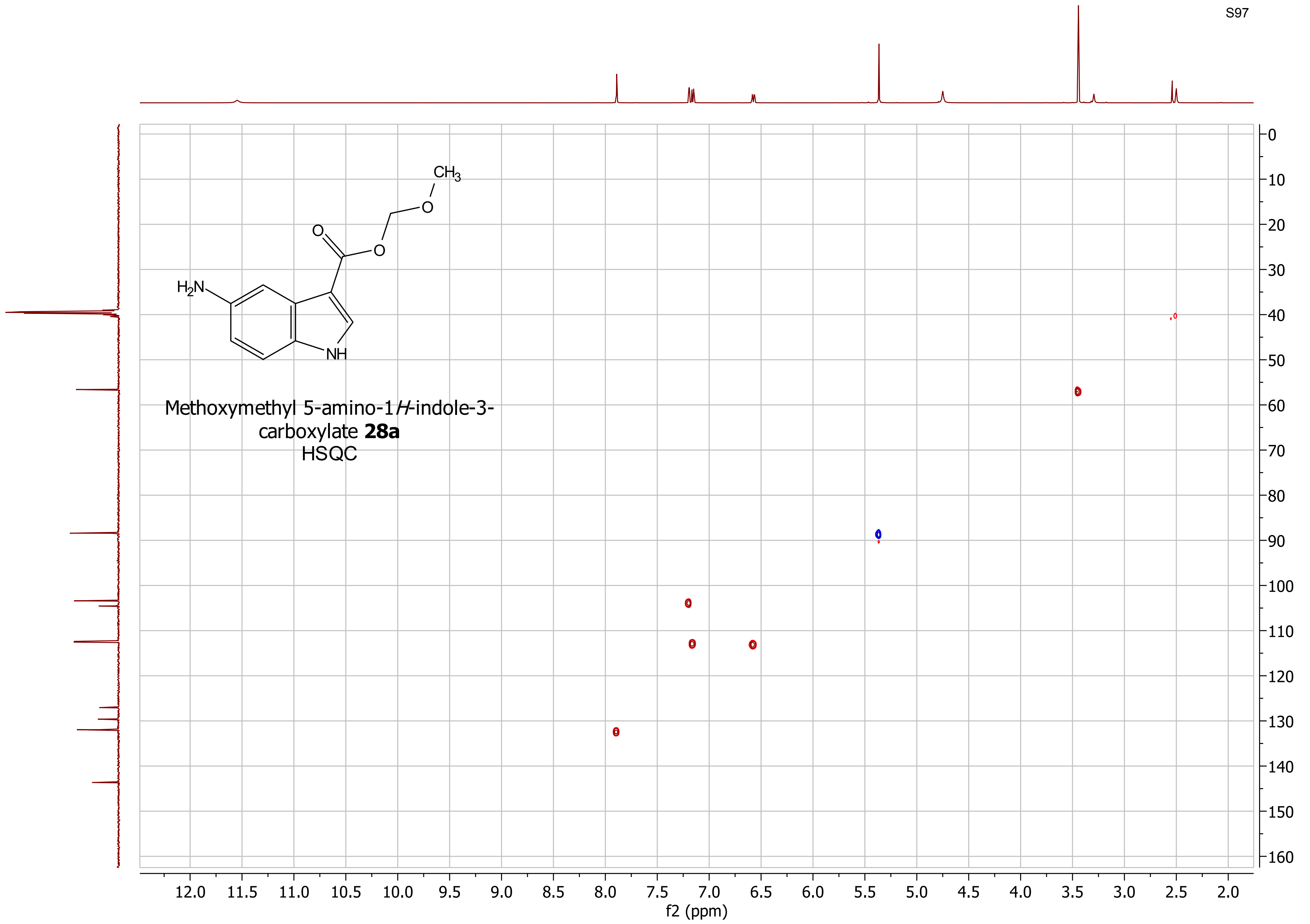
CH3
O
O
O
H2N
NH
Methoxymethyl 5-amino-1H-indole-3-carboxylate 28a
HSQC
f2 (ppm)
f1 (ppm)

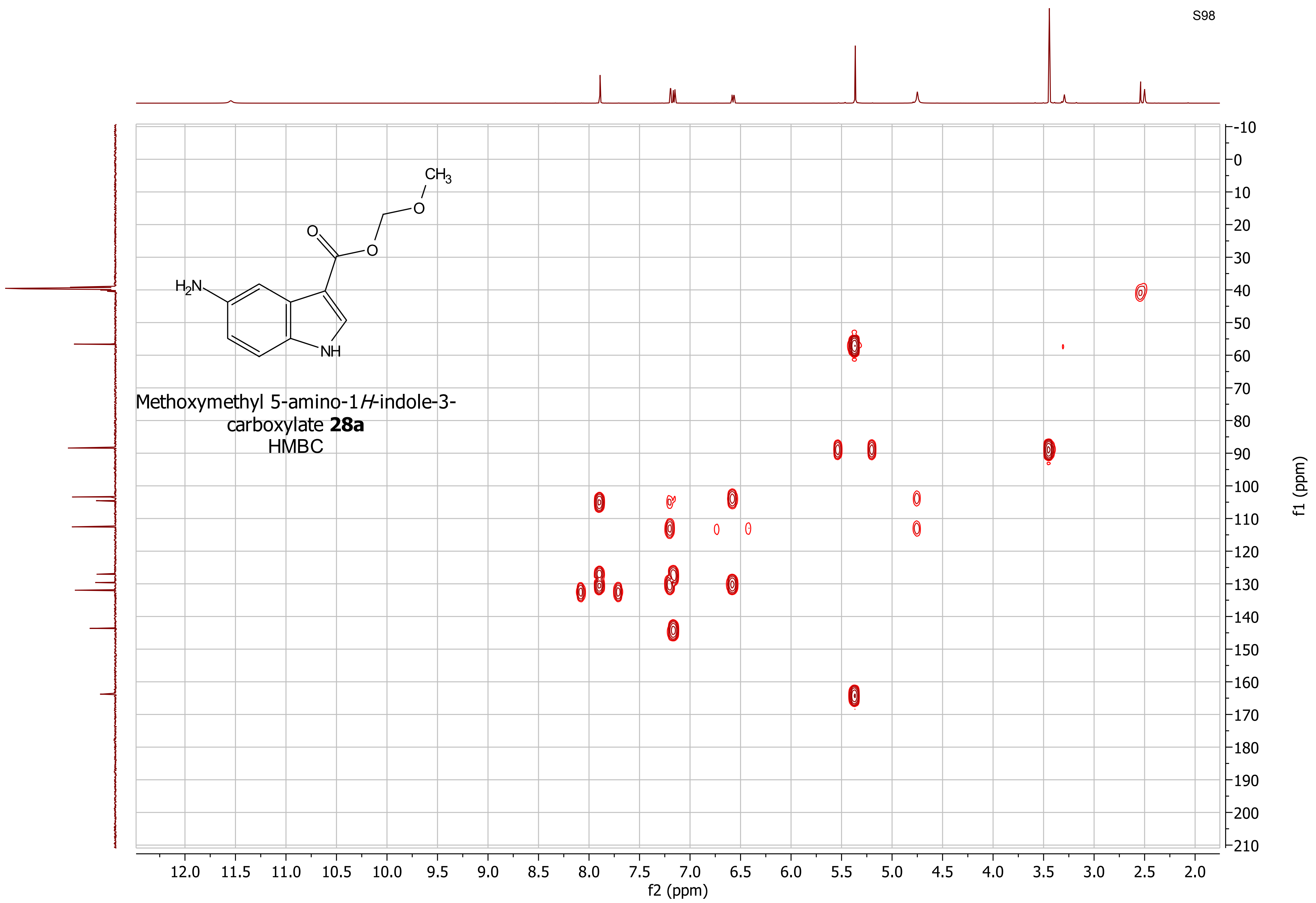

Methoxymethyl 5-amino-1*H*-indole-3-carboxylate **28a**
HMBC

^{1}H NMR (500 MHz, DMSO-d_6) δ 11.38 (s, 1H), 7.80 (s, 1H), 7.63 (d, J = 8.5 Hz, 1H), 6.63 (d, J = 1.9 Hz, 1H), 6.56 (dd, J = 8.4, 2.0 Hz, 1H), 5.36 (s, 2H), 4.89 (s, 2H), 3.43 (s, 3H).

Methoxymethyl 6-amino-1*H*-indole-3-carboxylate **28b**

^{1}H NMR (DMSO-d_6, 500 MHz)

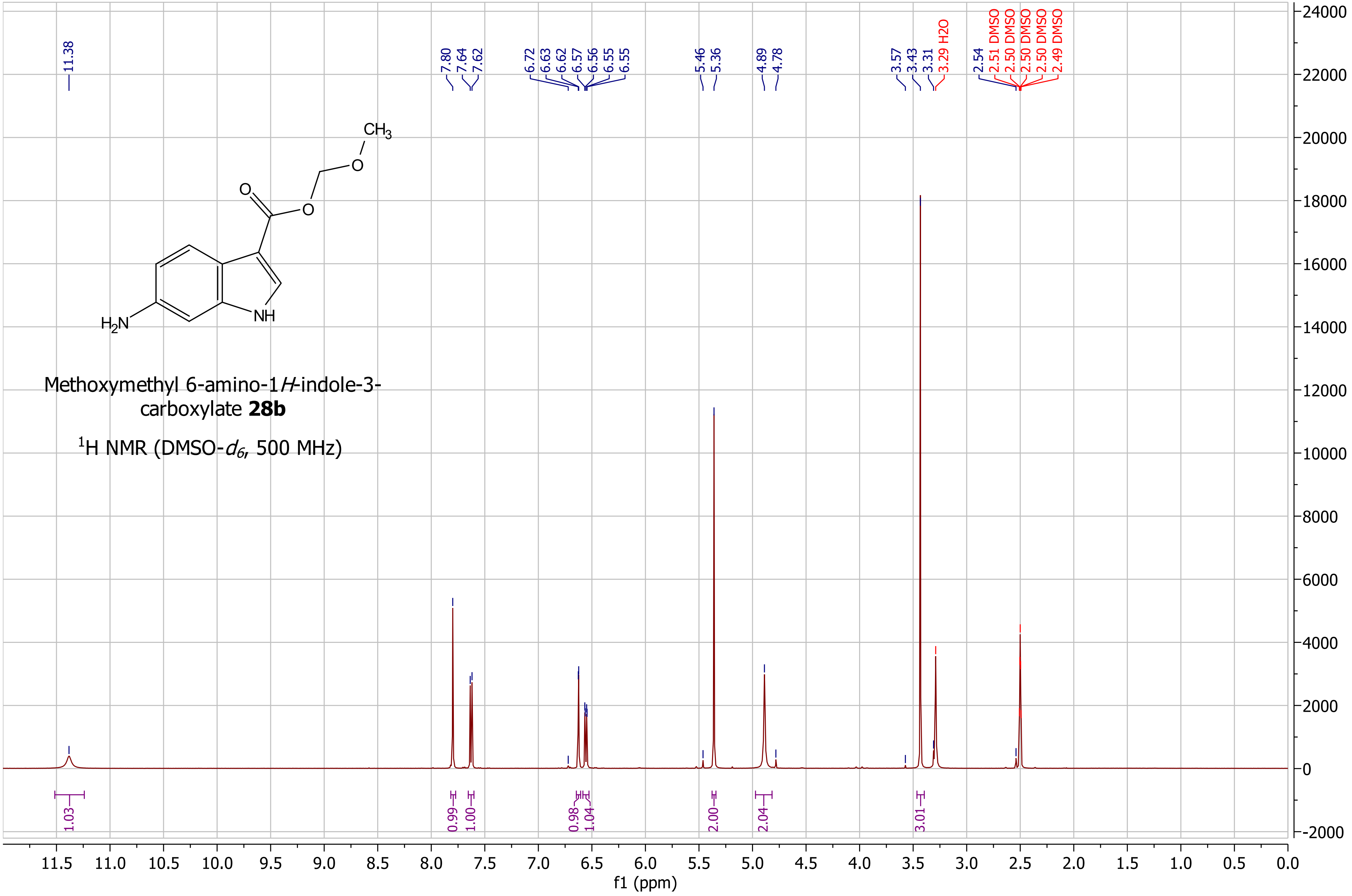

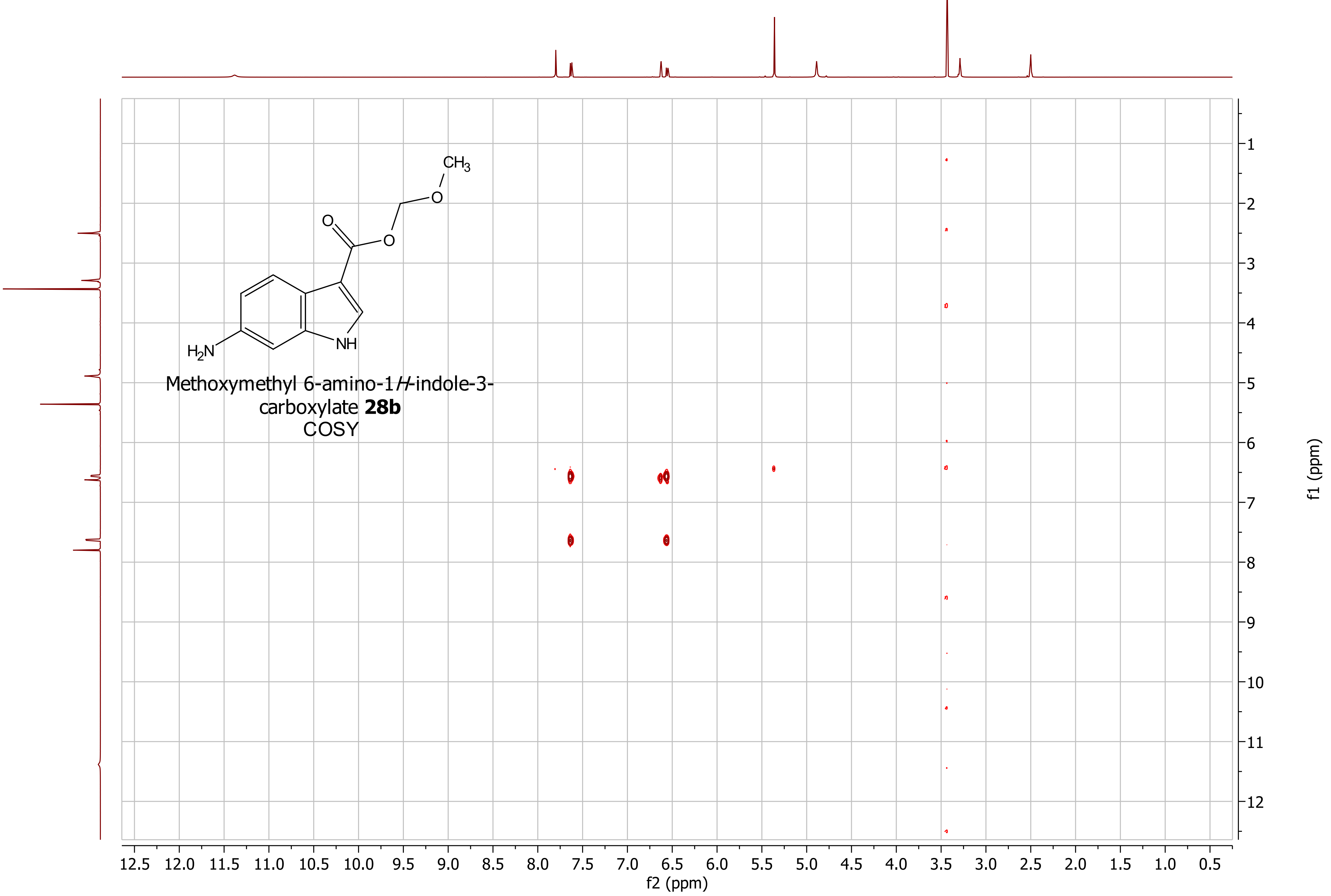

Methoxymethyl 6-amino-1*H*-indole-3-carboxylate **28b**
COSY

^{13}C NMR (126 MHz, DMSO-d_6) δ 163.7, 145.0, 138.1, 130.1, 120.5, 117.0, 111.8, 106.0, 95.5, 88.5, 56.6.

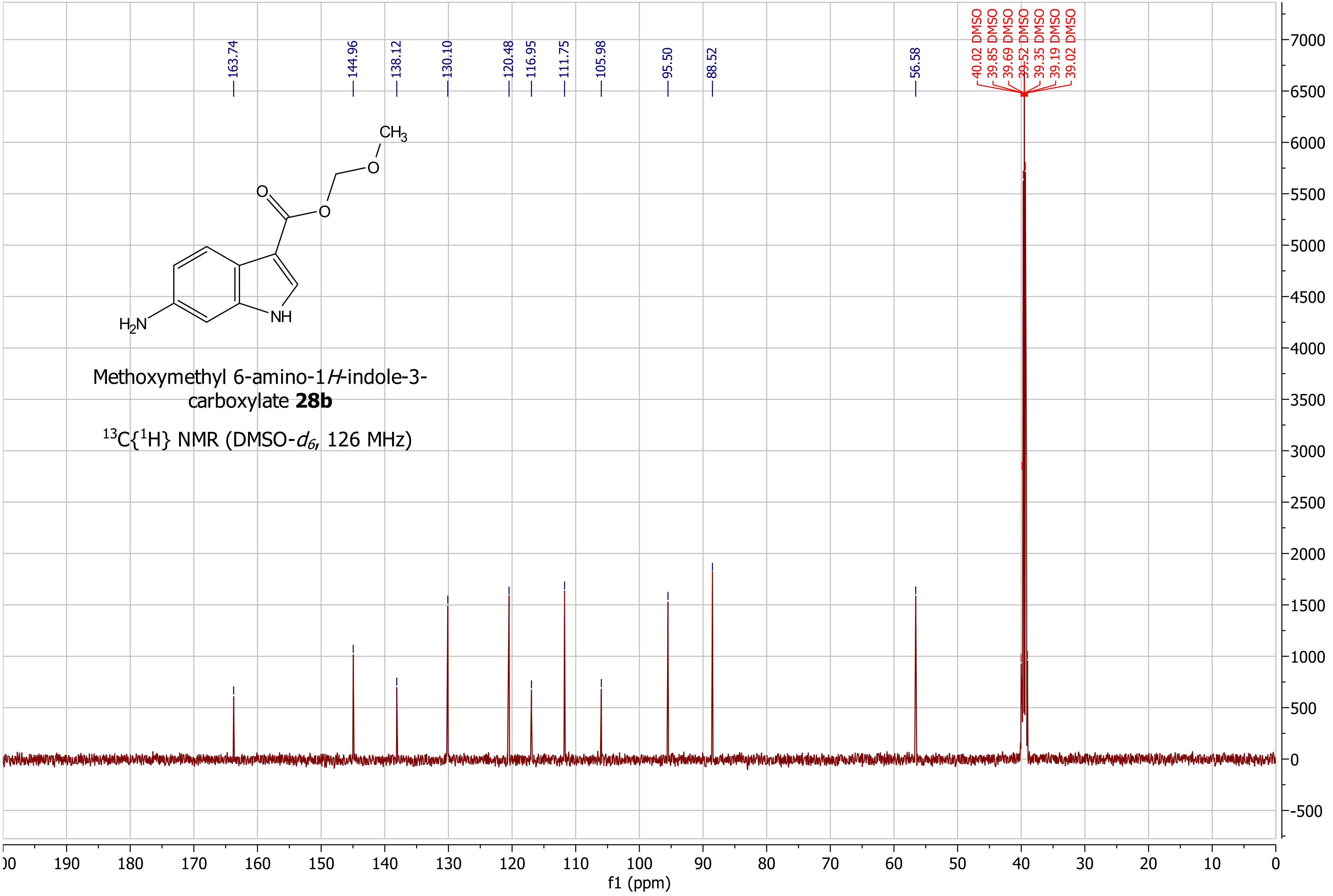

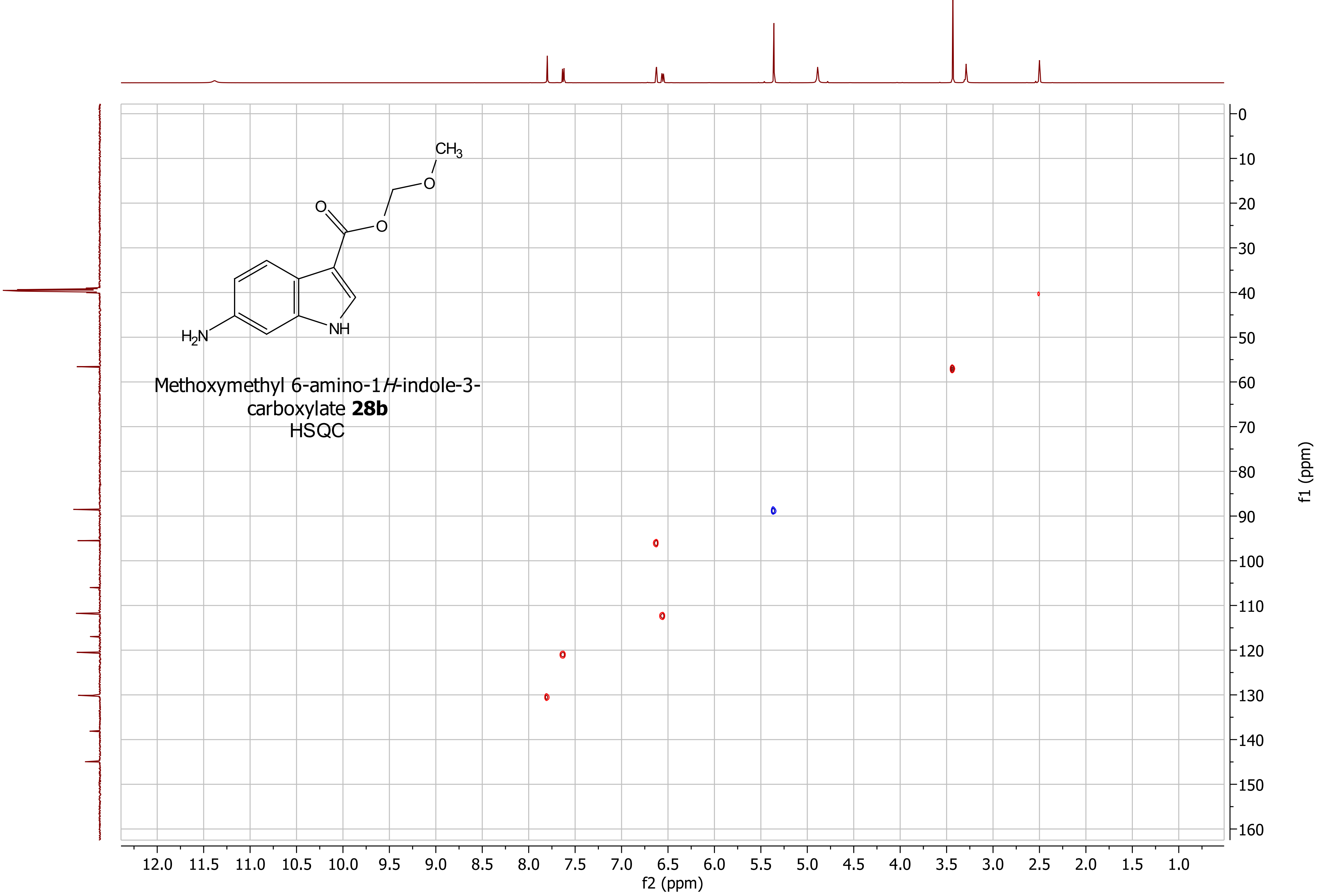

Methoxymethyl 6-amino-1*H*-indole-3-carboxylate **28b**
HSQC

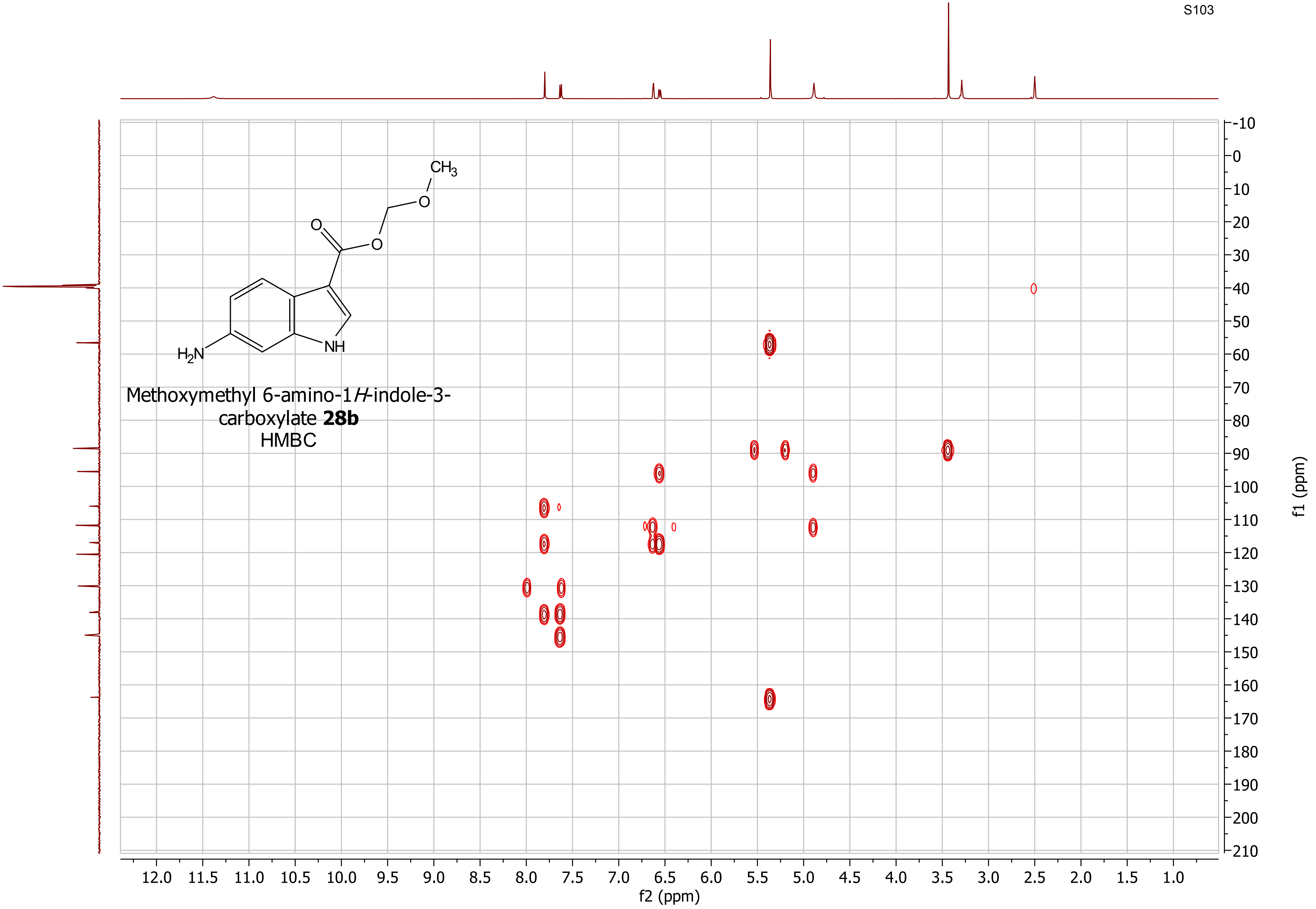
CH3
O
O
O
H2N
NH
Methoxymethyl 6-amino-1H-indole-3-carboxylate 28b
HMBC
f2 (ppm)
f1 (ppm)

^{1}H NMR (500 MHz, DMSO-d_6) δ 11.56 (s, 1H), 8.07 (s, 1H), 7.26 (d, J = 7.9 Hz, 1H), 6.91 (t, J = 7.7 Hz, 1H), 6.43 (d, J = 7.5 Hz, 1H), 5.39 (s, 2H), 5.19 (s, 2H), 3.45 (s, 3H).

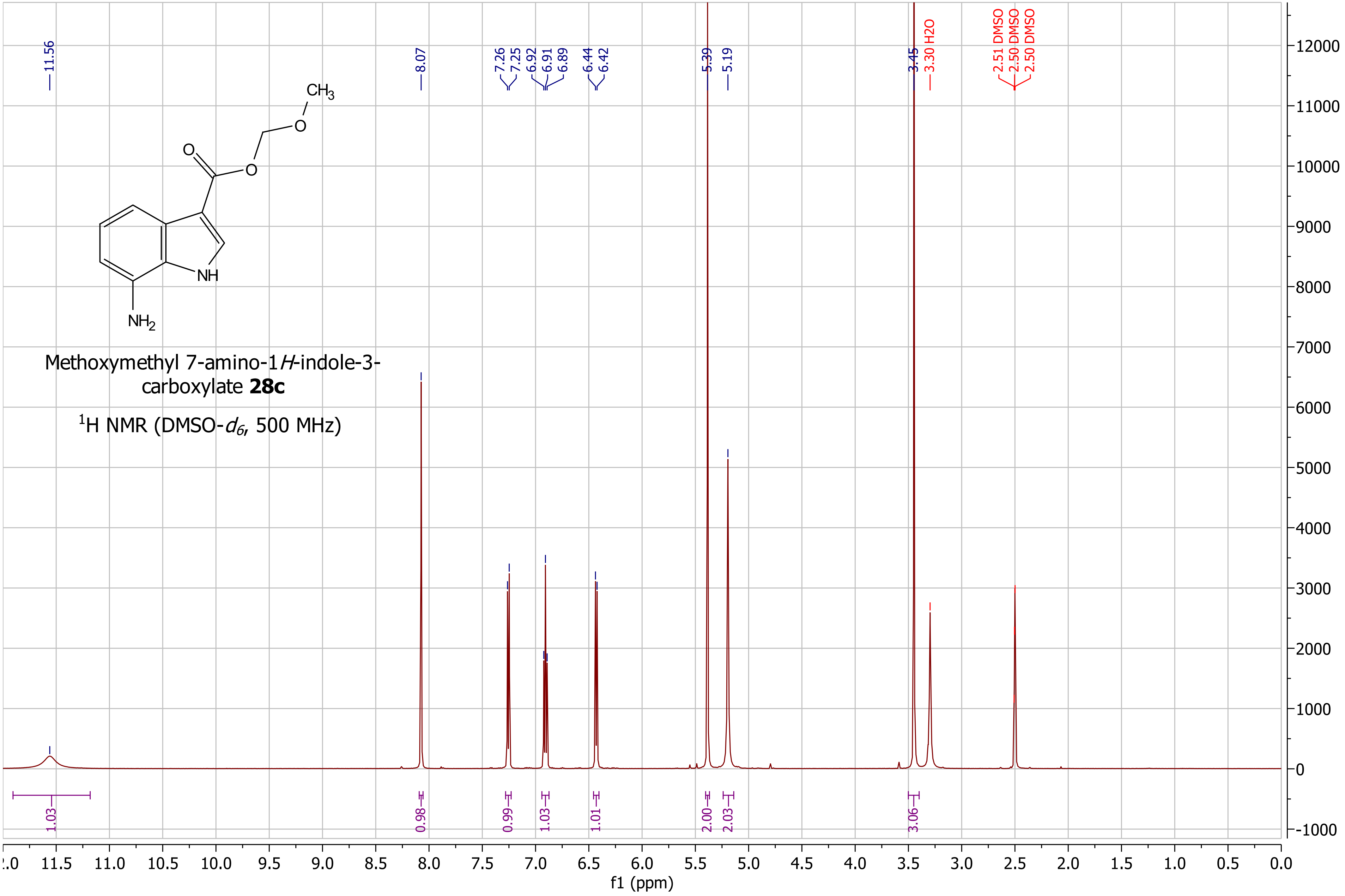

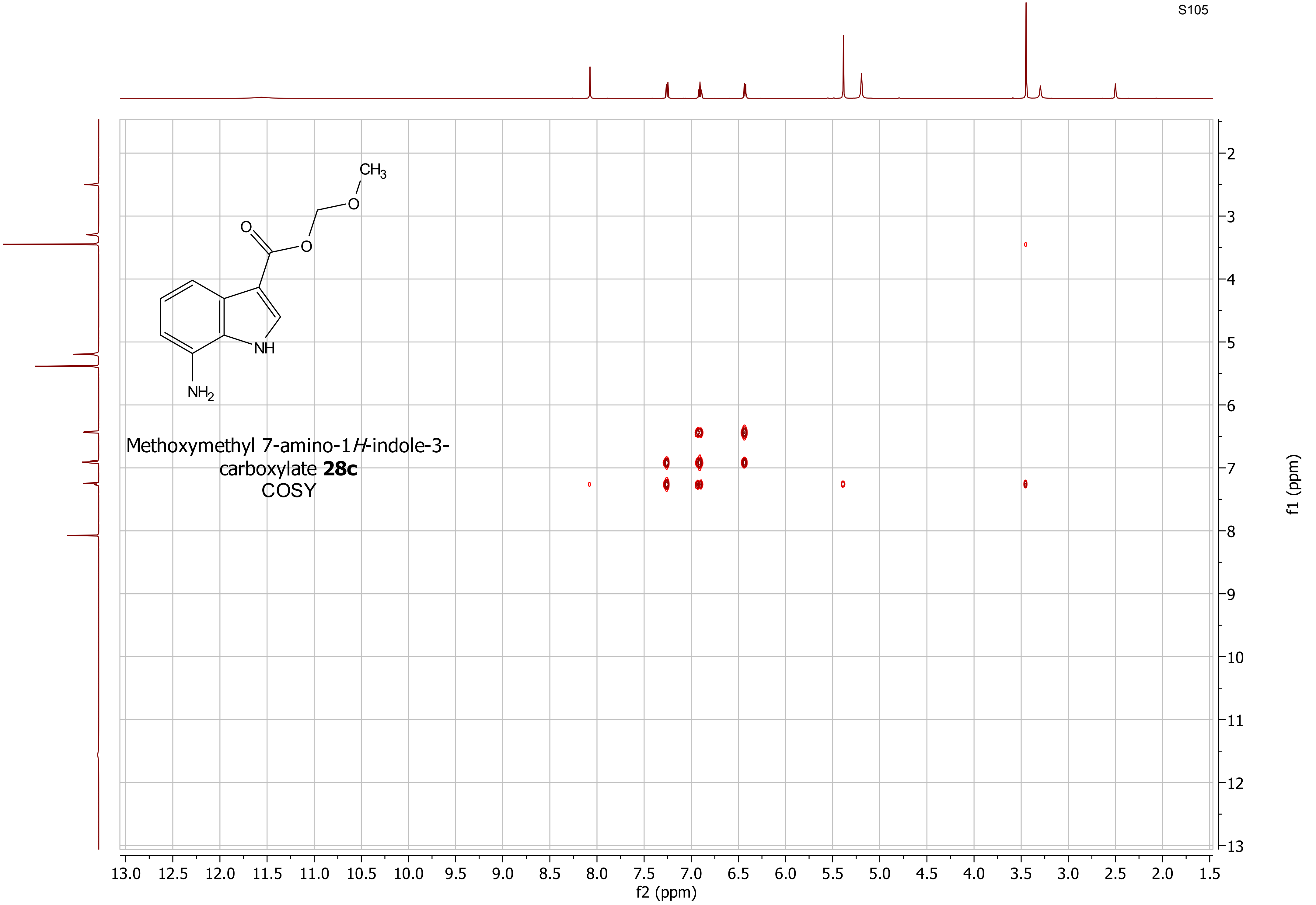
CH3
O
O
O
NH
NH2
Methoxymethyl 7-amino-1H-indole-3-carboxylate 28c
COSY
f2 (ppm)
f1 (ppm)
13.0 12.5 12.0 11.5 11.0 10.5 10.0 9.5 9.0 8.5 8.0 7.5 7.0 6.5 6.0 5.5 5.0 4.5 4.0 3.5 3.0 2.5 2.0 1.5
2 3 4 5 6 7 8 9 10 11 12 13

^{13}C NMR (126 MHz, DMSO-d_6) δ 164.3, 134.9, 132.1, 127.1, 126.2, 123.1, 109.1, 106.8, 106.6, 89.2, 57.2.

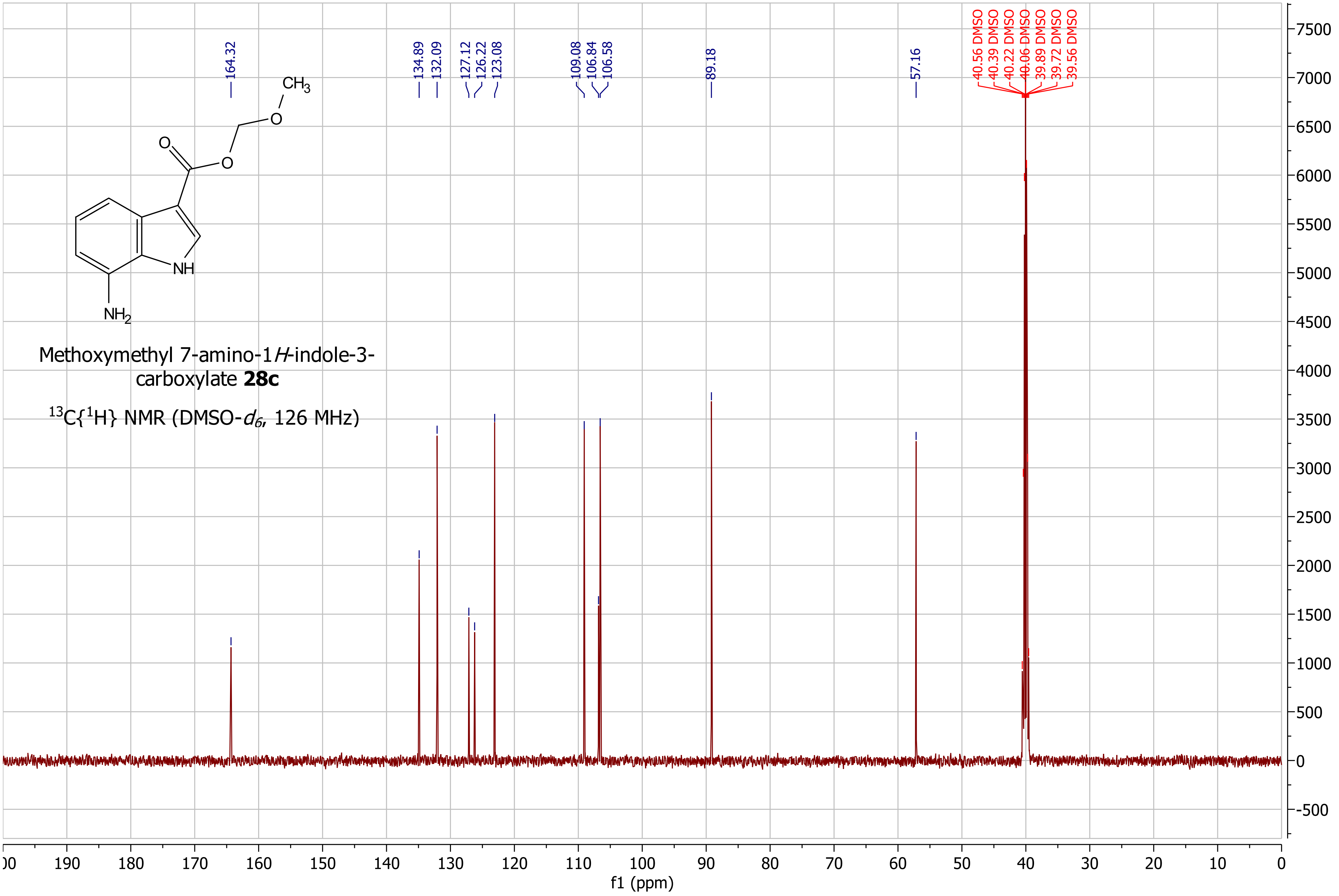

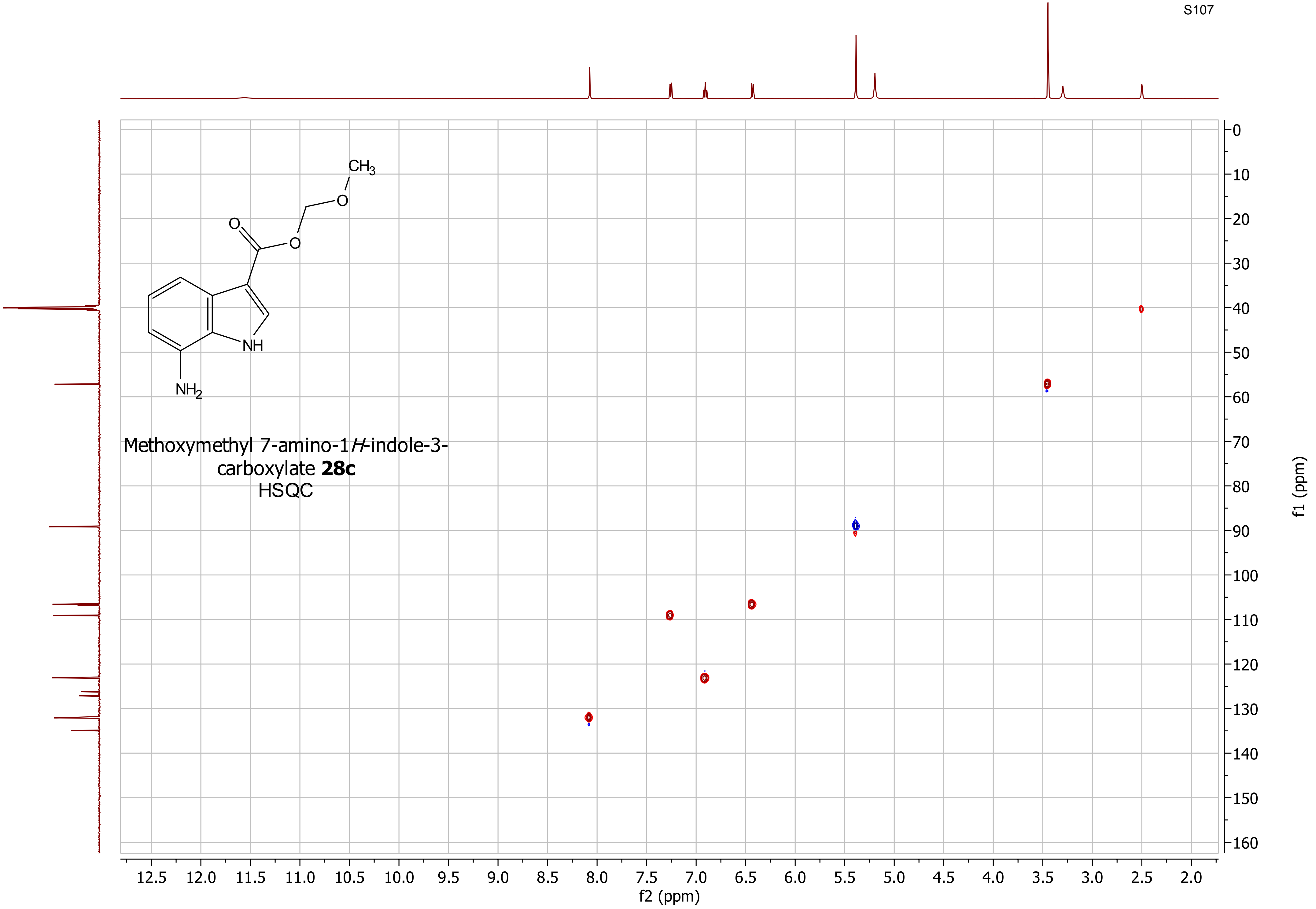
CH3
O
O
O
NH
NH2
Methoxymethyl 7-amino-1H-indole-3-carboxylate 28c
HSQC
f2 (ppm)
f1 (ppm)

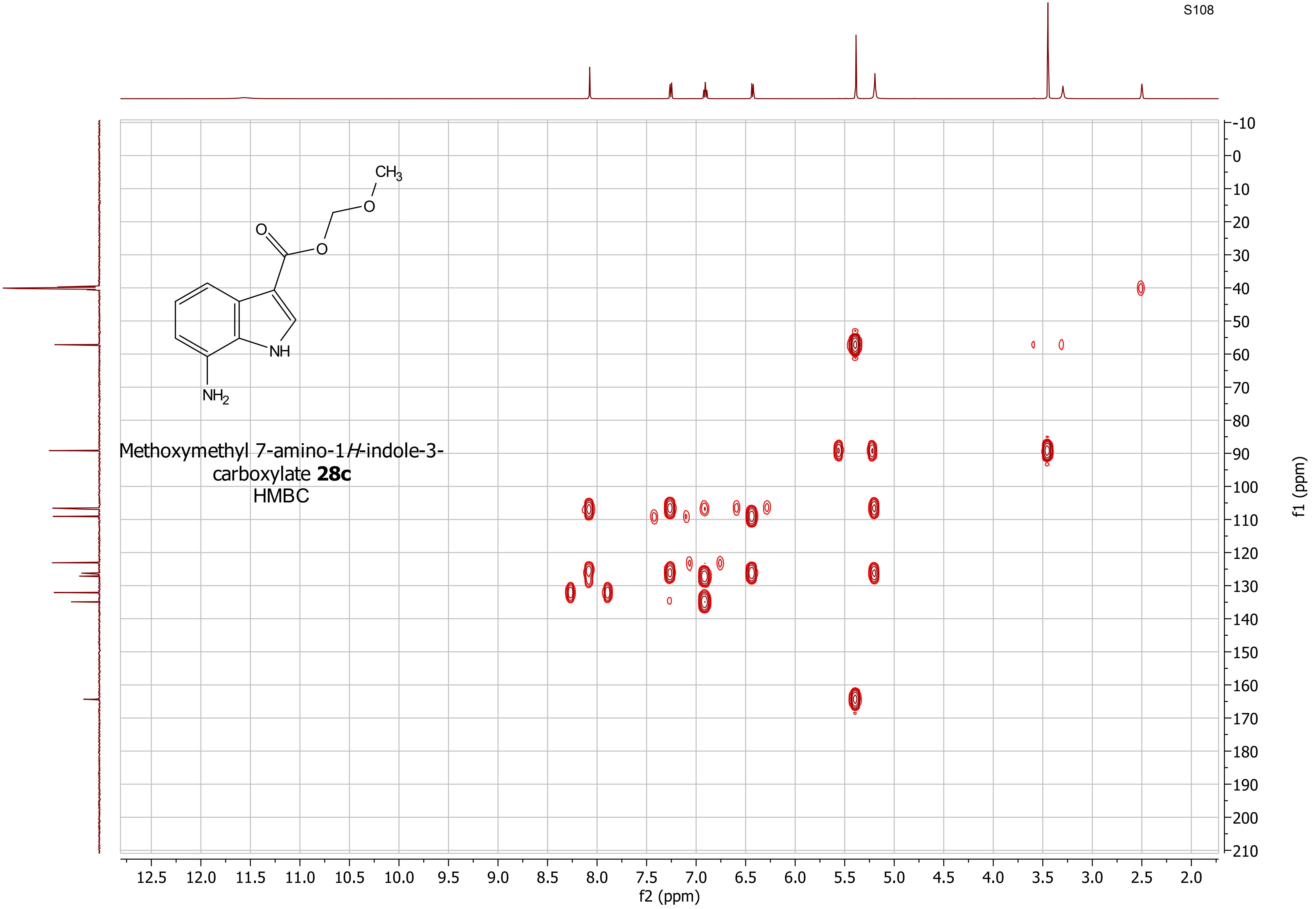
CH3
O
O
O
NH
NH2
Methoxymethyl 7-amino-1H-indole-3-carboxylate 28c
HMBC
12.5 12.0 11.5 11.0 10.5 10.0 9.5 9.0 8.5 8.0 7.5 7.0 6.5 6.0 5.5 5.0 4.5 4.0 3.5 3.0 2.5 2.0
f2 (ppm)
-10 0 10 20 30 40 50 60 70 80 90 100 110 120 130 140 150 160 170 180 190 200 210
f1 (ppm)

^{1}H NMR (500 MHz, DMSO-d_6) δ 11.89 (s, 1H), 9.63 (s, 1H), 8.21 (s, 1H), 8.09 (s, 1H), 7.44 (d, J = 6.9 Hz, 2H), 7.42 – 7.37 (m, 3H), 7.36 – 7.29 (m, 2H), 5.40 (s, 2H), 5.16 (s, 2H), 3.47 (s, 3H).

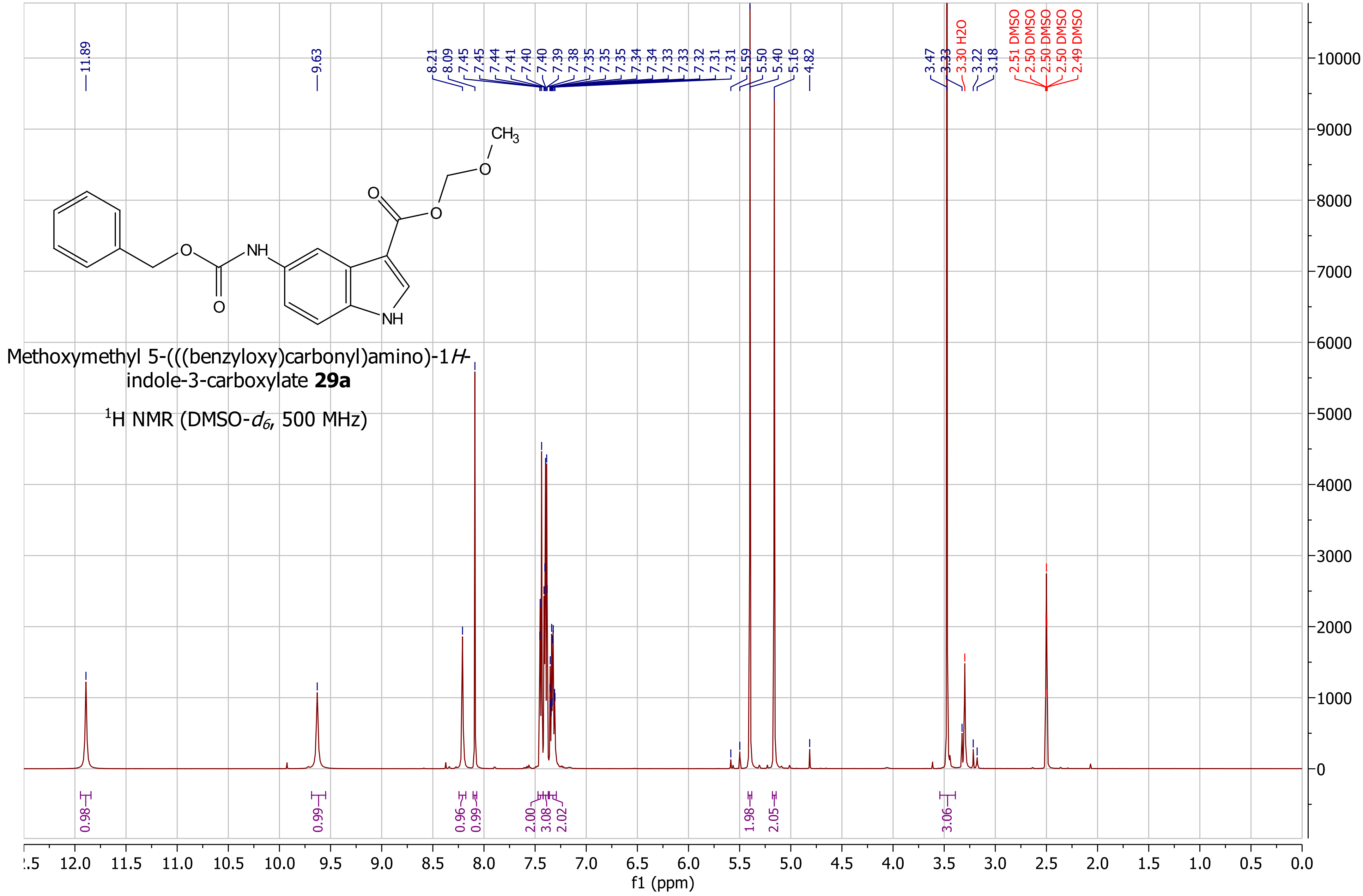

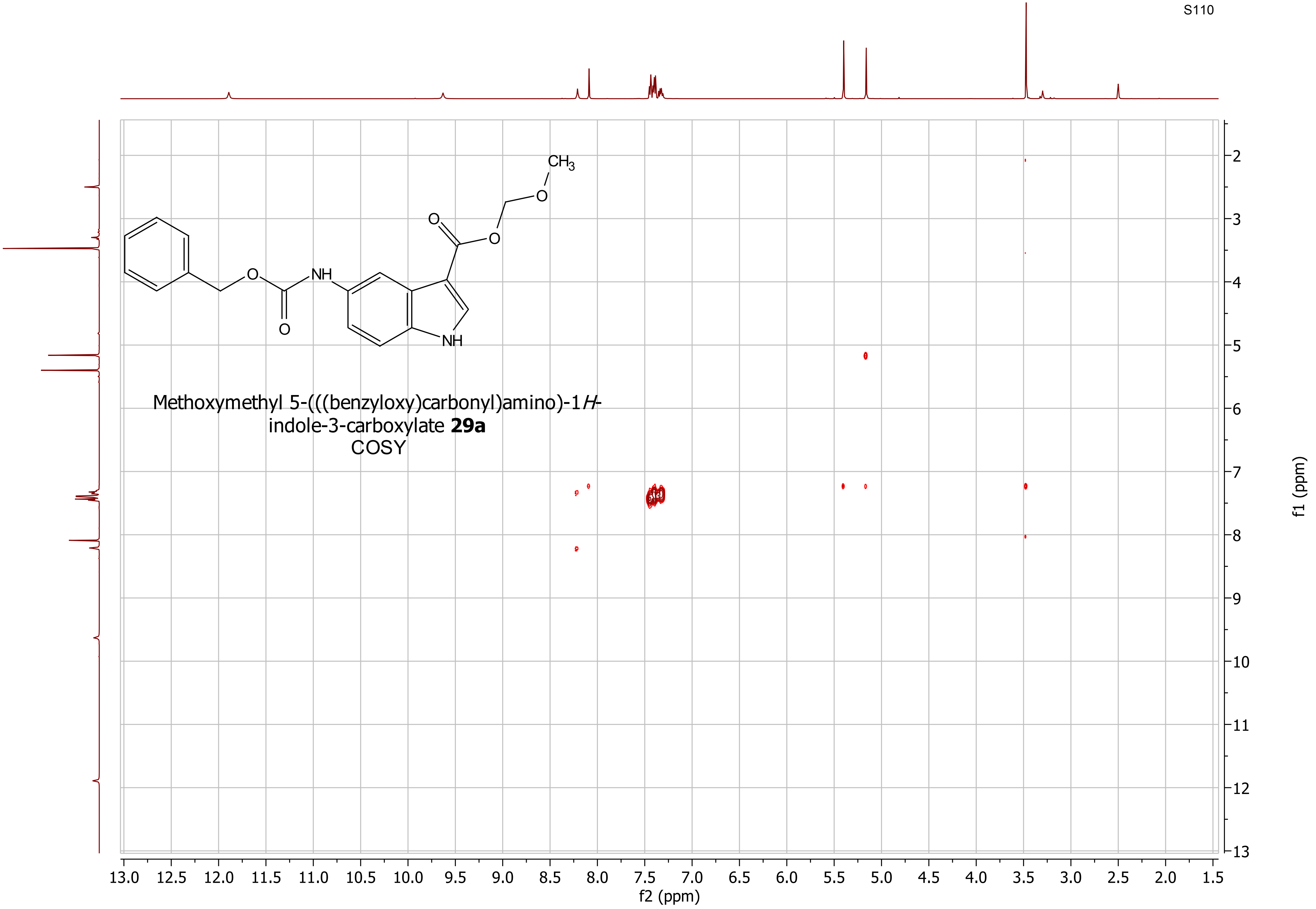
Methoxymethyl 5-(((benzyloxy)carbonyl)amino)-1*H*-indole-3-carboxylate **29a**
COSY
f2 (ppm)
f1 (ppm)

^{13}C NMR (126 MHz, DMSO-d_6) δ 164.1, 154.2, 137.4, 133.9, 133.3, 128.9, 128.5, 128.4, 126.4, 116.0, 112.8, 110.6, 106.4, 89.3, 66.0, 57.3.

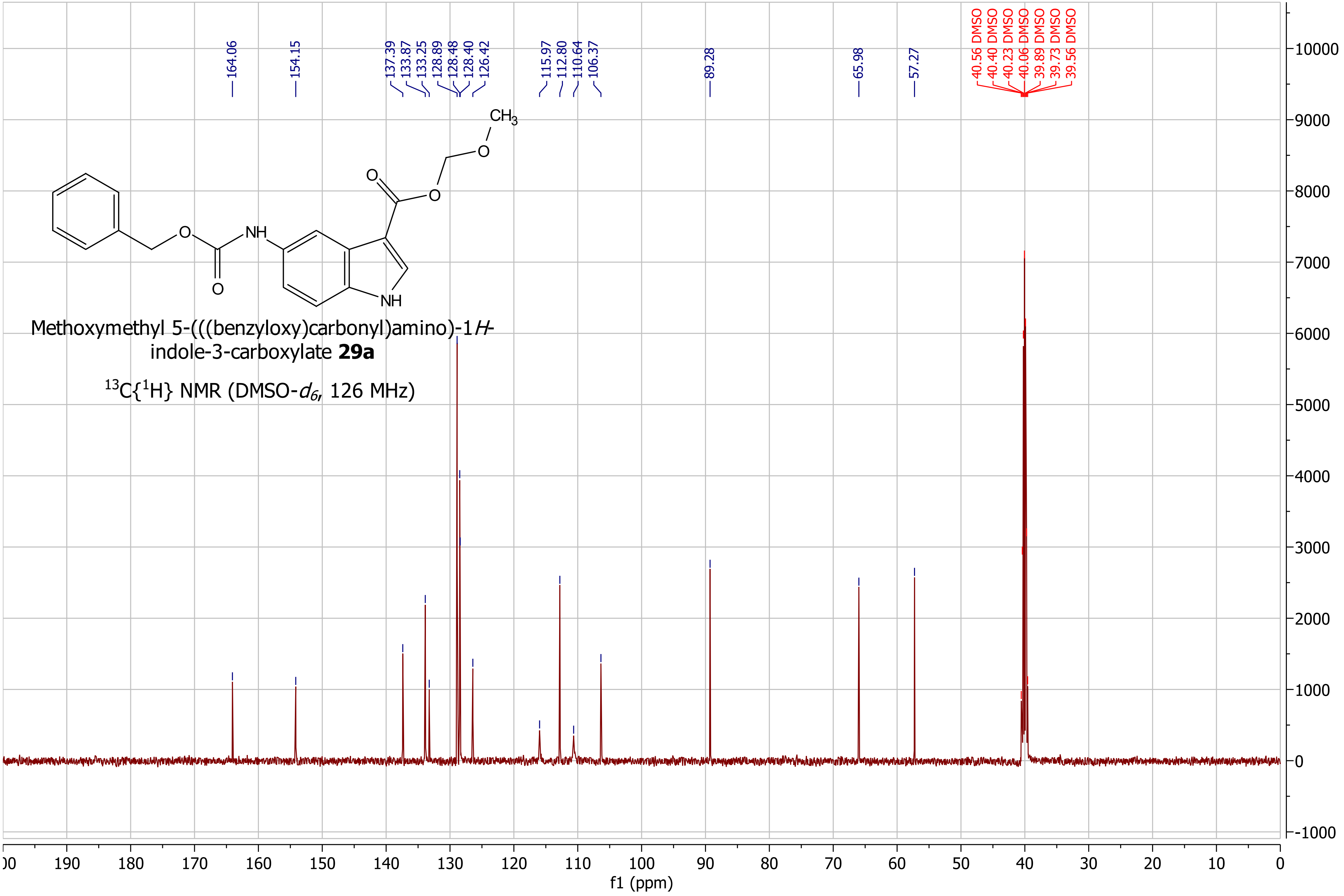

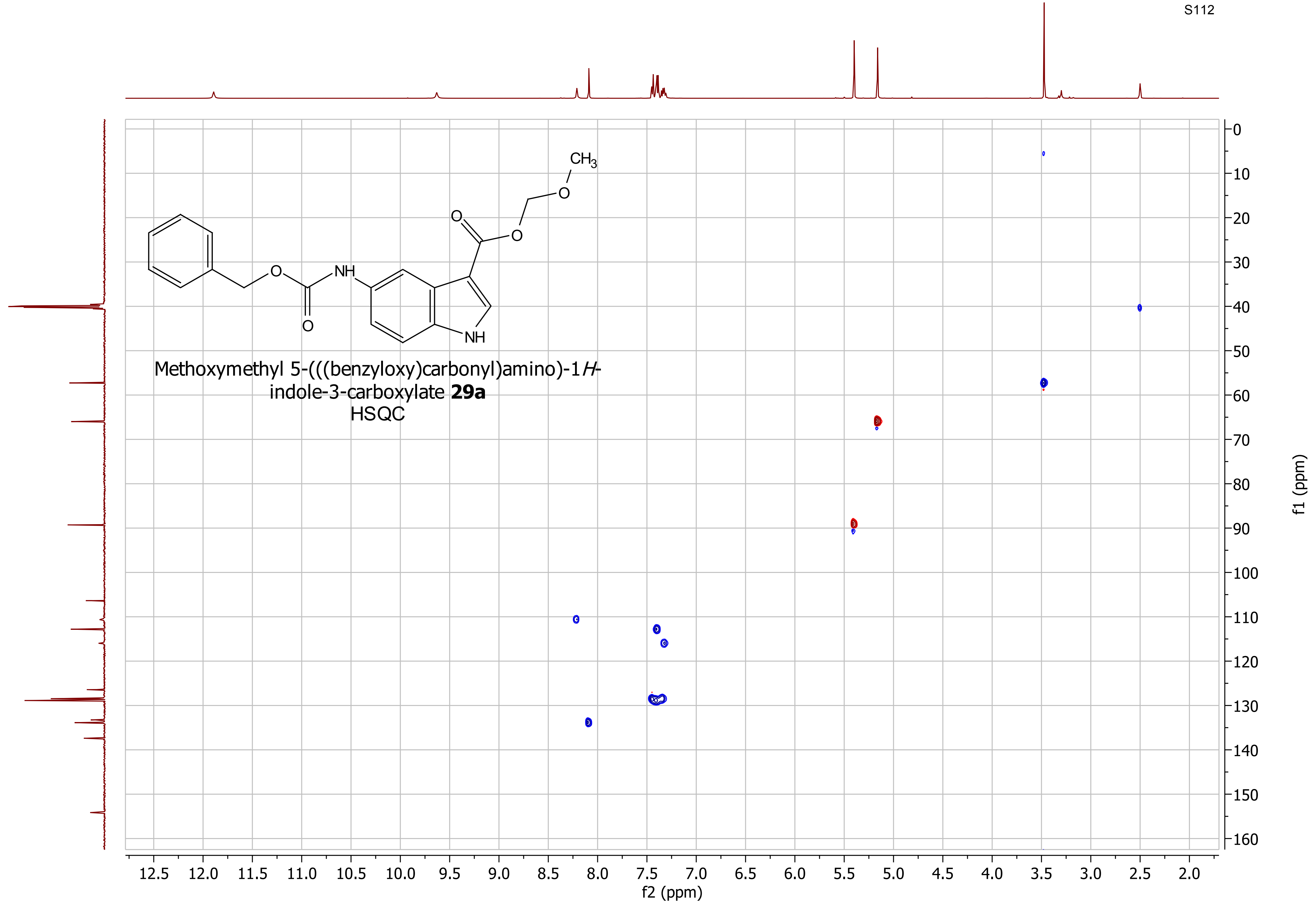
CH3
O
O
O
NH
O
O
NH
Methoxymethyl 5-(((benzyloxy)carbonyl)amino)-1H-indole-3-carboxylate 29a
HSQC
f1 (ppm)
f2 (ppm)

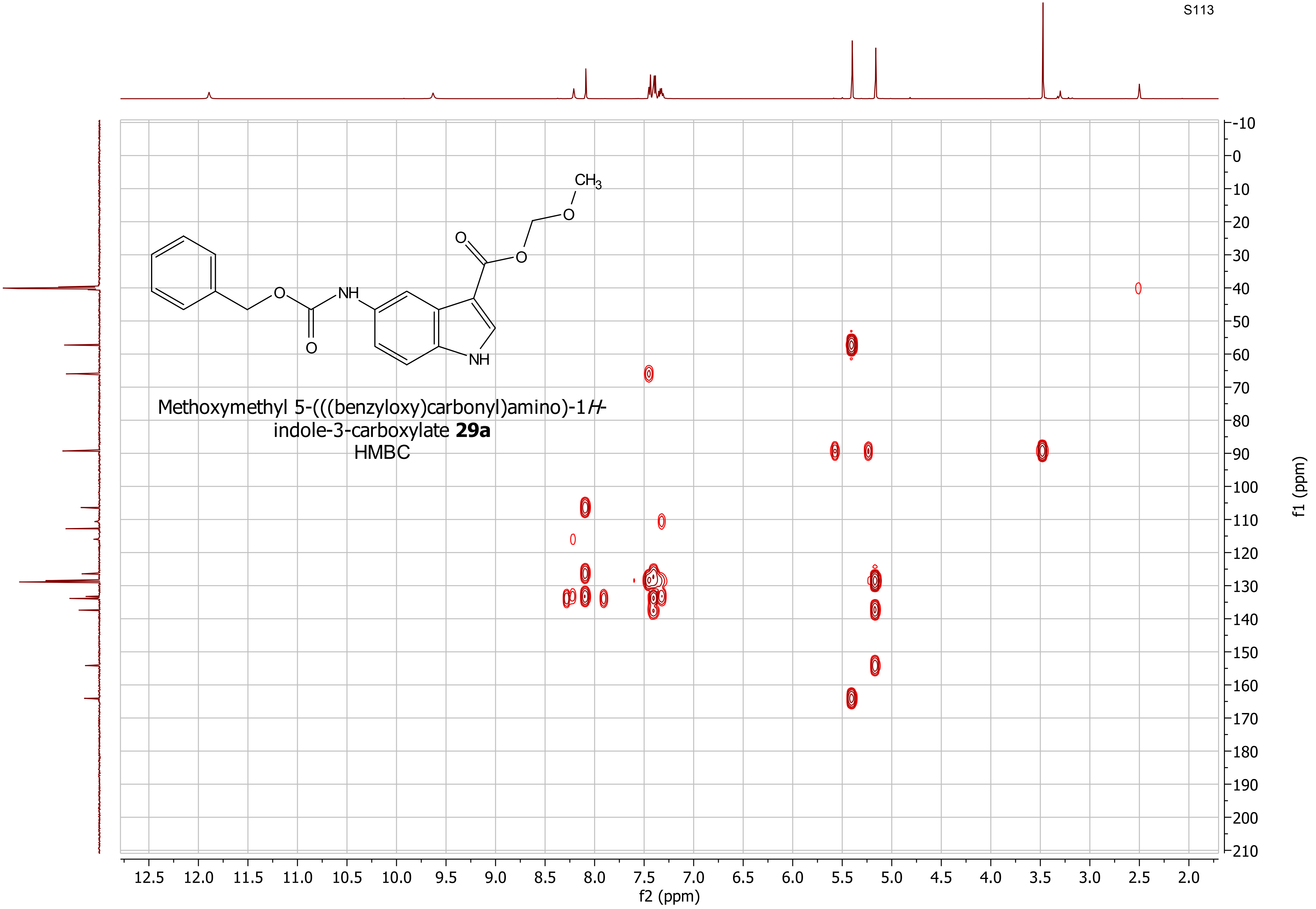

Methoxymethyl 5-(((benzyloxy)carbonyl)amino)-1*H*-indole-3-carboxylate **29a**
HMBC

^{1}H NMR (500 MHz, DMSO-d_6) δ 11.86 (s, 1H), 9.74 (s, 1H), 8.05 (s, 1H), 7.88 – 7.82 (m, 2H), 7.45 (d, J = 7.0 Hz, 2H), 7.42 – 7.37 (m, 2H), 7.37 – 7.31 (m, 1H), 7.21 (dd, J = 8.6, 1.9 Hz, 1H), 5.39 (s, 2H), 5.17 (s, 2H), 3.45 (s, 3H).

Methoxymethyl 6-(((benzyloxy)carbonyl)amino)-1*H*-indole-3-carboxylate **29b**

^{1}H NMR (DMSO-d_6, 500 MHz)

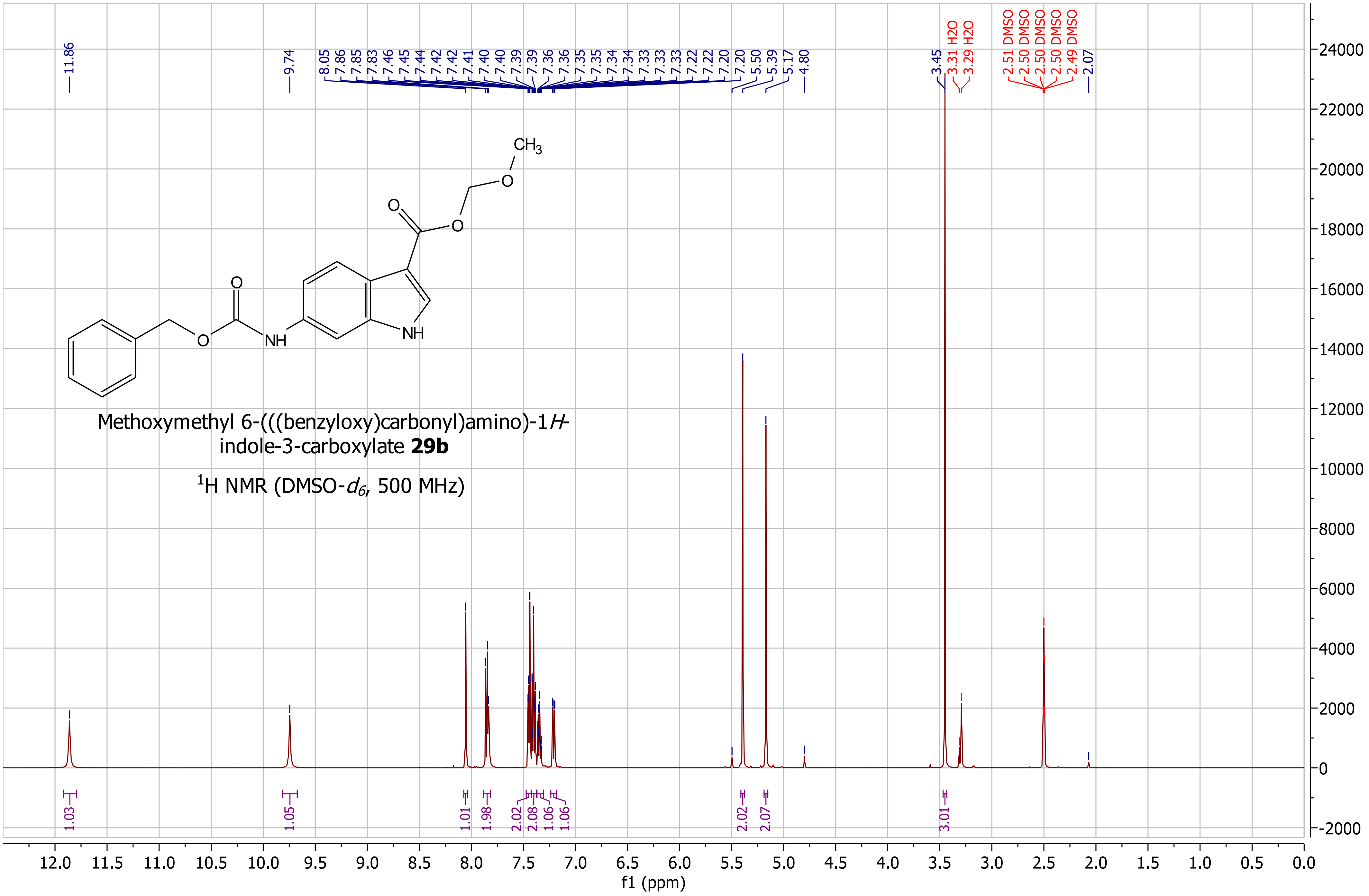

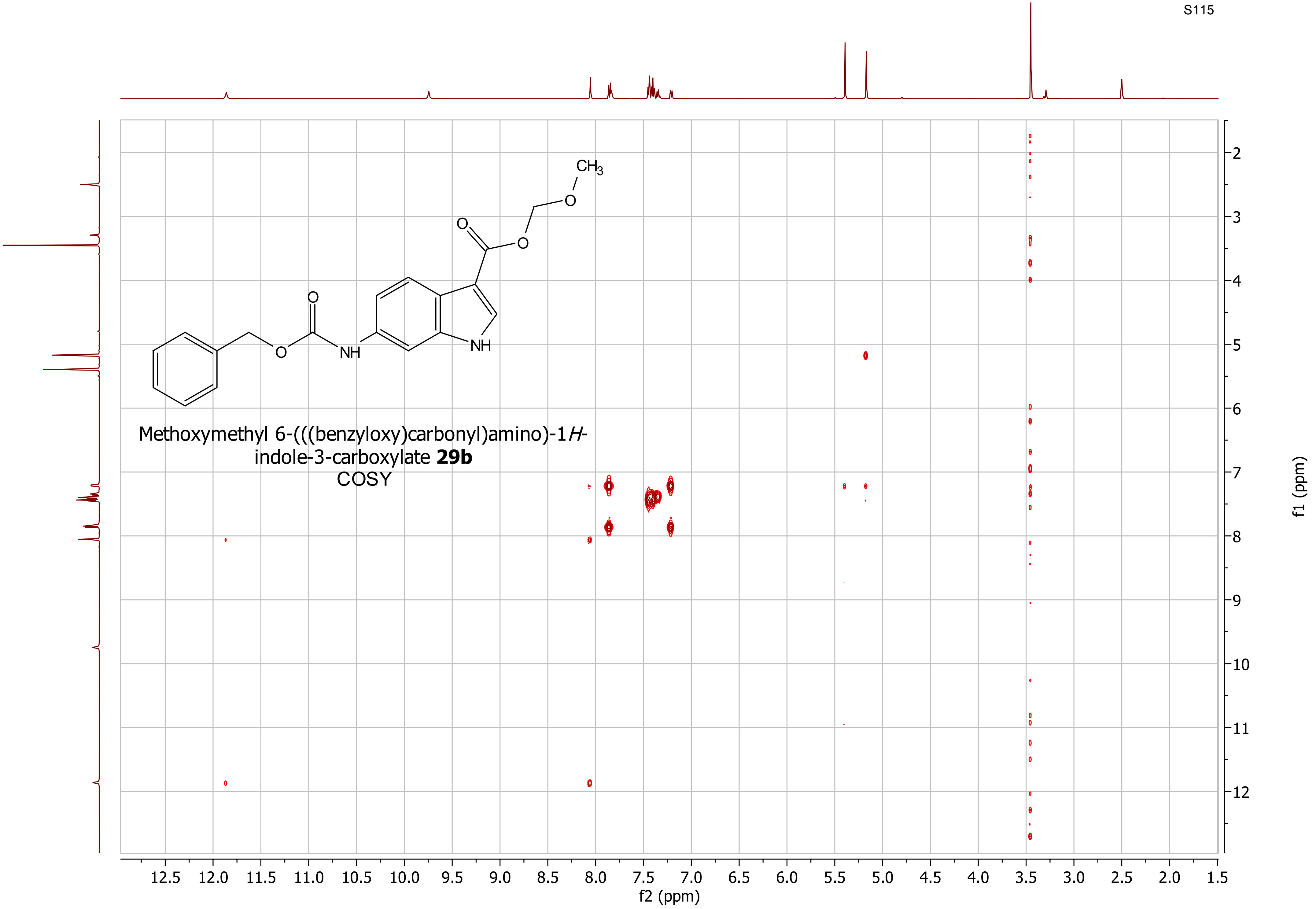

Methoxymethyl 6-(((benzyloxy)carbonyl)amino)-1*H*-indole-3-carboxylate **29b**
COSY

^{13}C NMR (126 MHz, DMSO-d_6) δ 163.6, 153.5, 136.7, 136.7, 134.5, 132.5, 128.4, 128.0, 127.9, 121.2, 120.3, 113.9, 106.0, 101.6, 88.8, 65.6, 56.7.

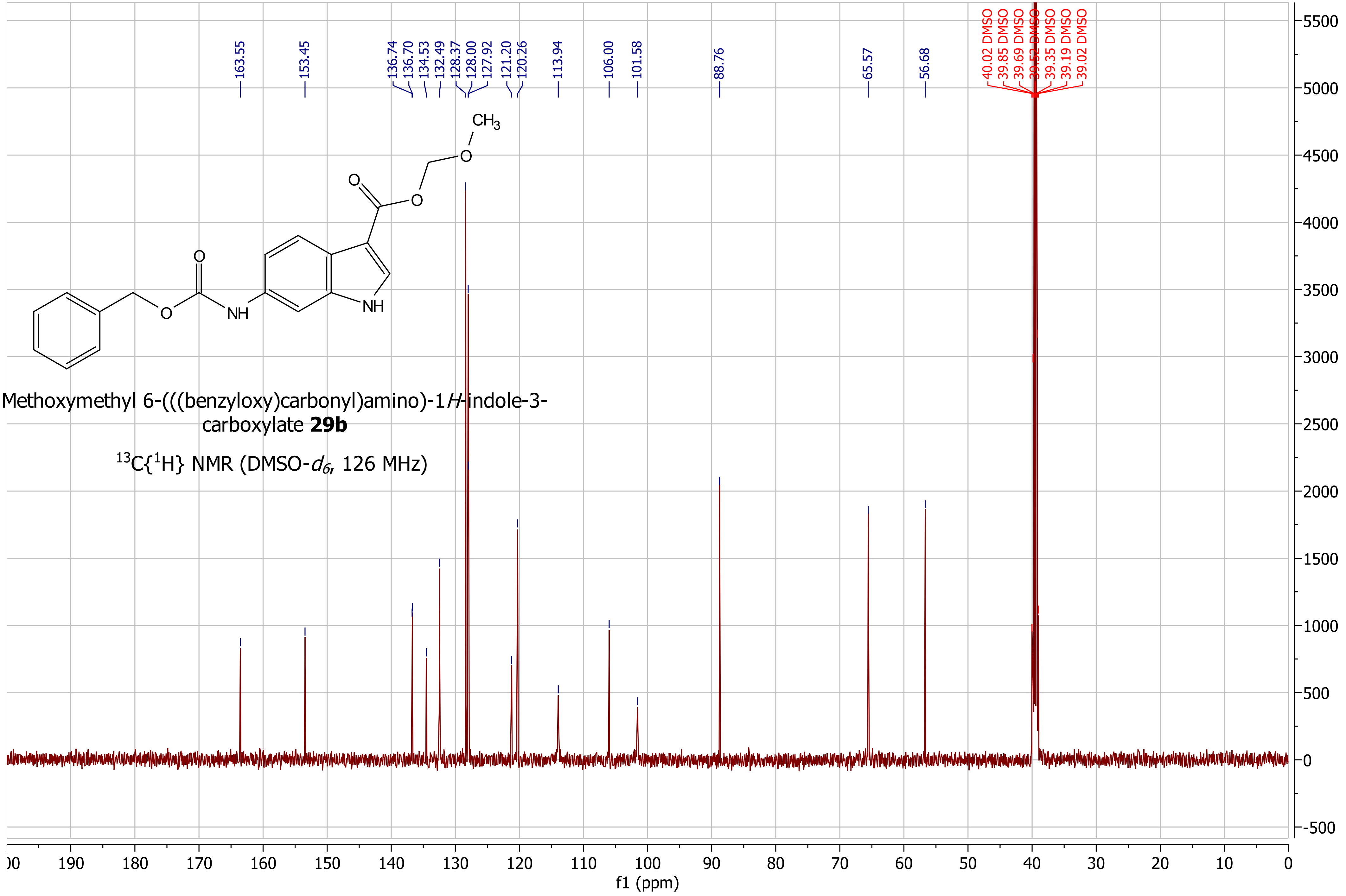

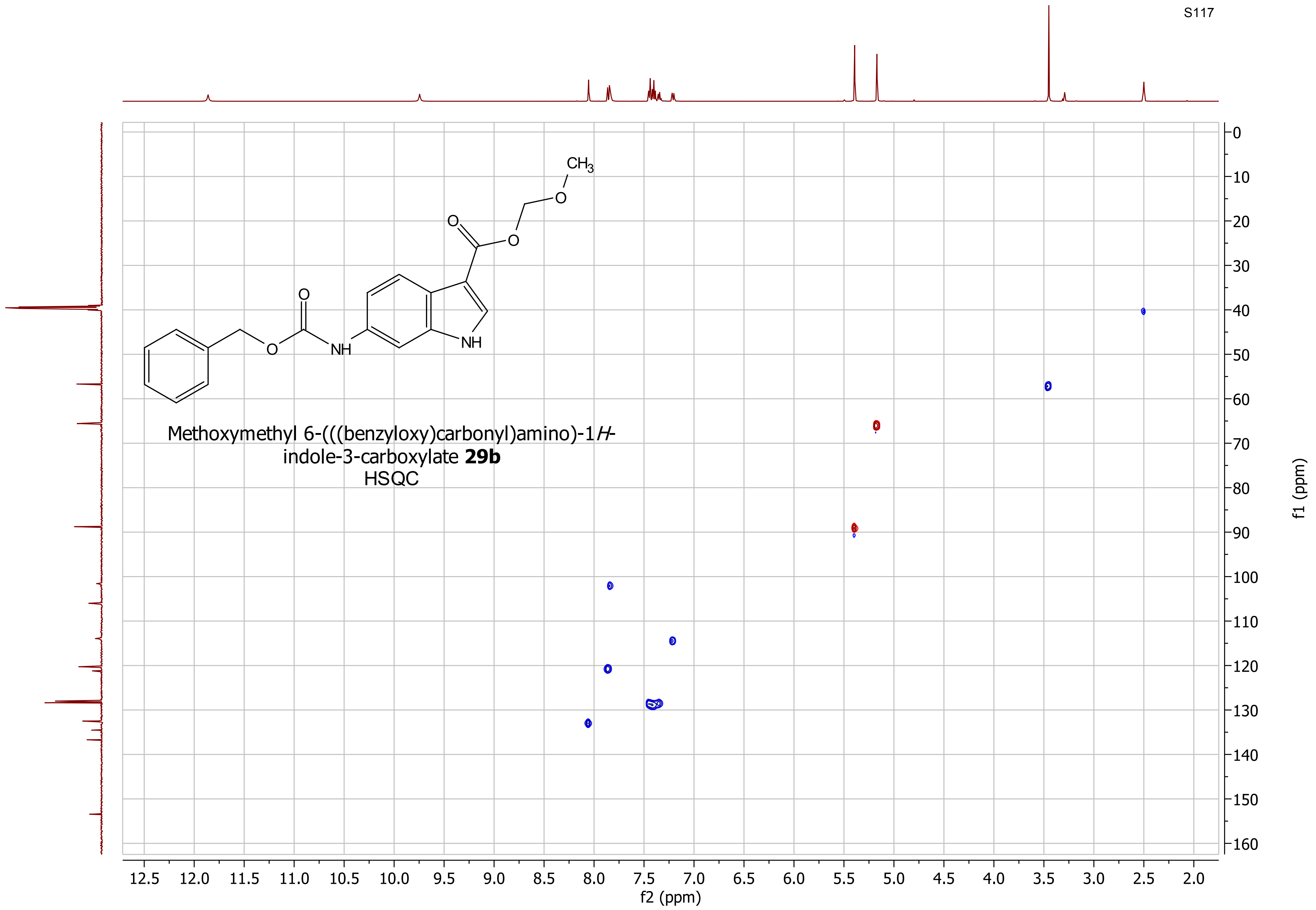
CH3
O
O
O
O
O
NH
NH
Methoxymethyl 6-(((benzyloxy)carbonyl)amino)-1H-
indole-3-carboxylate 29b
HSQC
f2 (ppm)
f1 (ppm)
12.5 12.0 11.5 11.0 10.5 10.0 9.5 9.0 8.5 8.0 7.5 7.0 6.5 6.0 5.5 5.0 4.5 4.0 3.5 3.0 2.5 2.0
0 10 20 30 40 50 60 70 80 90 100 110 120 130 140 150 160

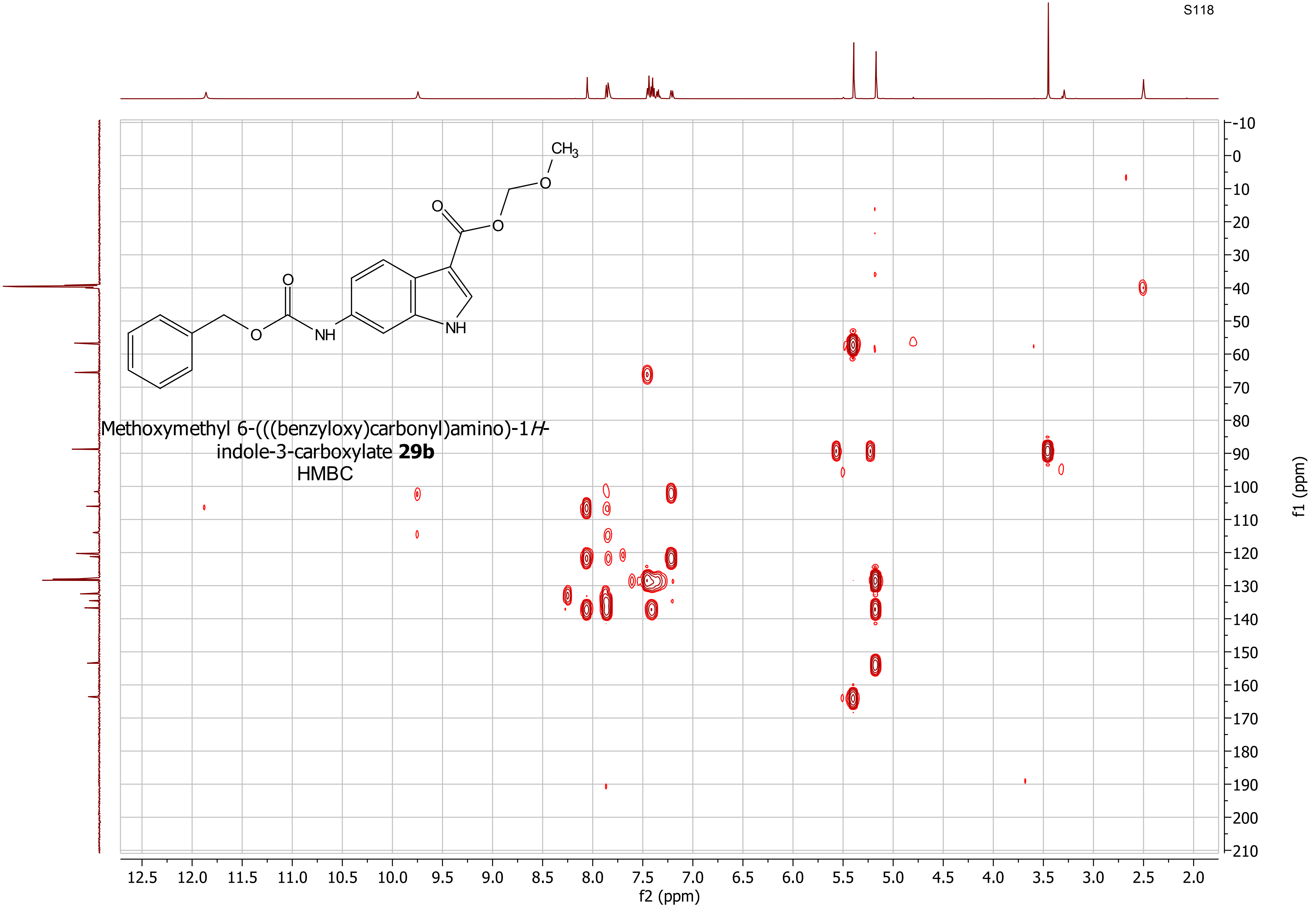
Methoxymethyl 6-(((benzyloxy)carbonyl)amino)-1H-indole-3-carboxylate 29b
HMBC
f2 (ppm)
f1 (ppm)

^{1}H NMR (500 MHz, DMSO-d_6) δ 11.70 (s, 1H), 9.53 (s, 1H), 8.14 (s, 1H), 7.80 – 7.75 (m, 1H), 7.53 – 7.44 (m, 3H), 7.41 (t, J = 7.4 Hz, 2H), 7.35 (t, J = 7.2 Hz, 1H), 7.16 (t, J = 7.9 Hz, 1H), 5.41 (s, 2H), 5.21 (s, 2H), 3.46 (s, 3H).

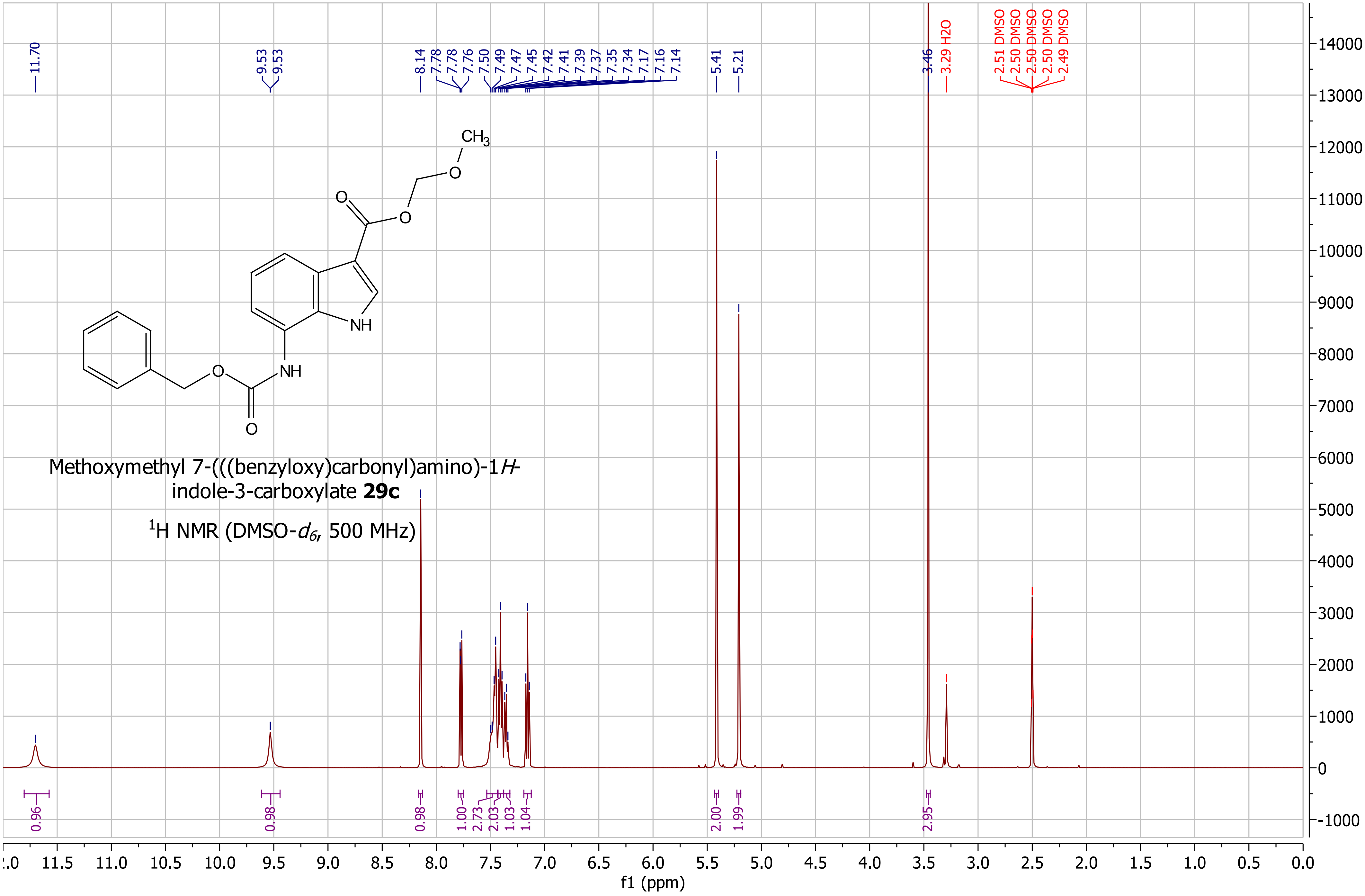

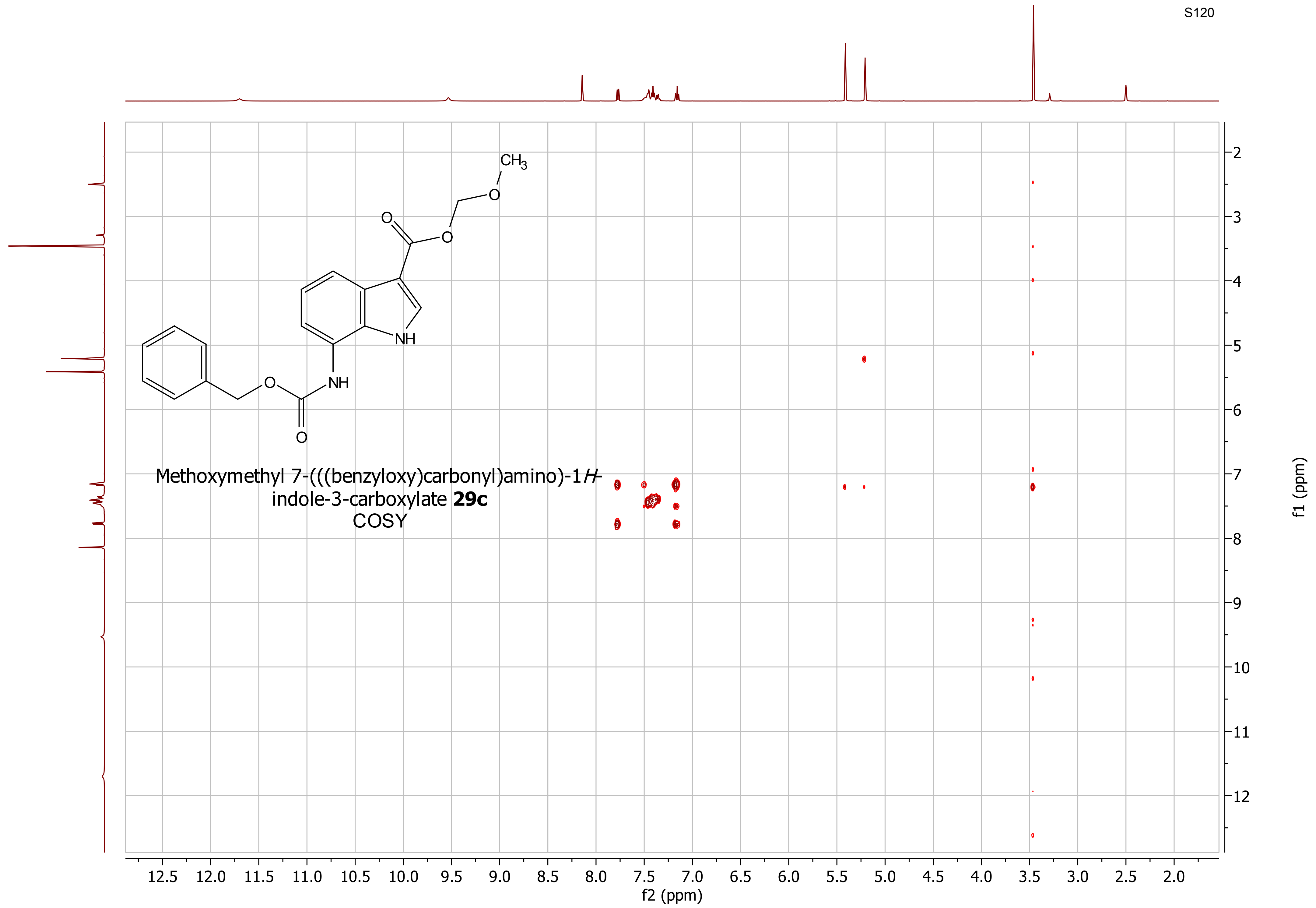
Methoxymethyl 7-(((benzyloxy)carbonyl)amino)-1H-indole-3-carboxylate 29c
COSY
f2 (ppm)
f1 (ppm)

^{13}C NMR (126 MHz, DMSO-d_6) δ 163.5, 153.7, 136.4, 132.7, 128.4, 128.1, 128.0, 127.0, 124.1, 121.7, 116.0, 113.7, 106.4, 88.9, 66.1, 56.7.

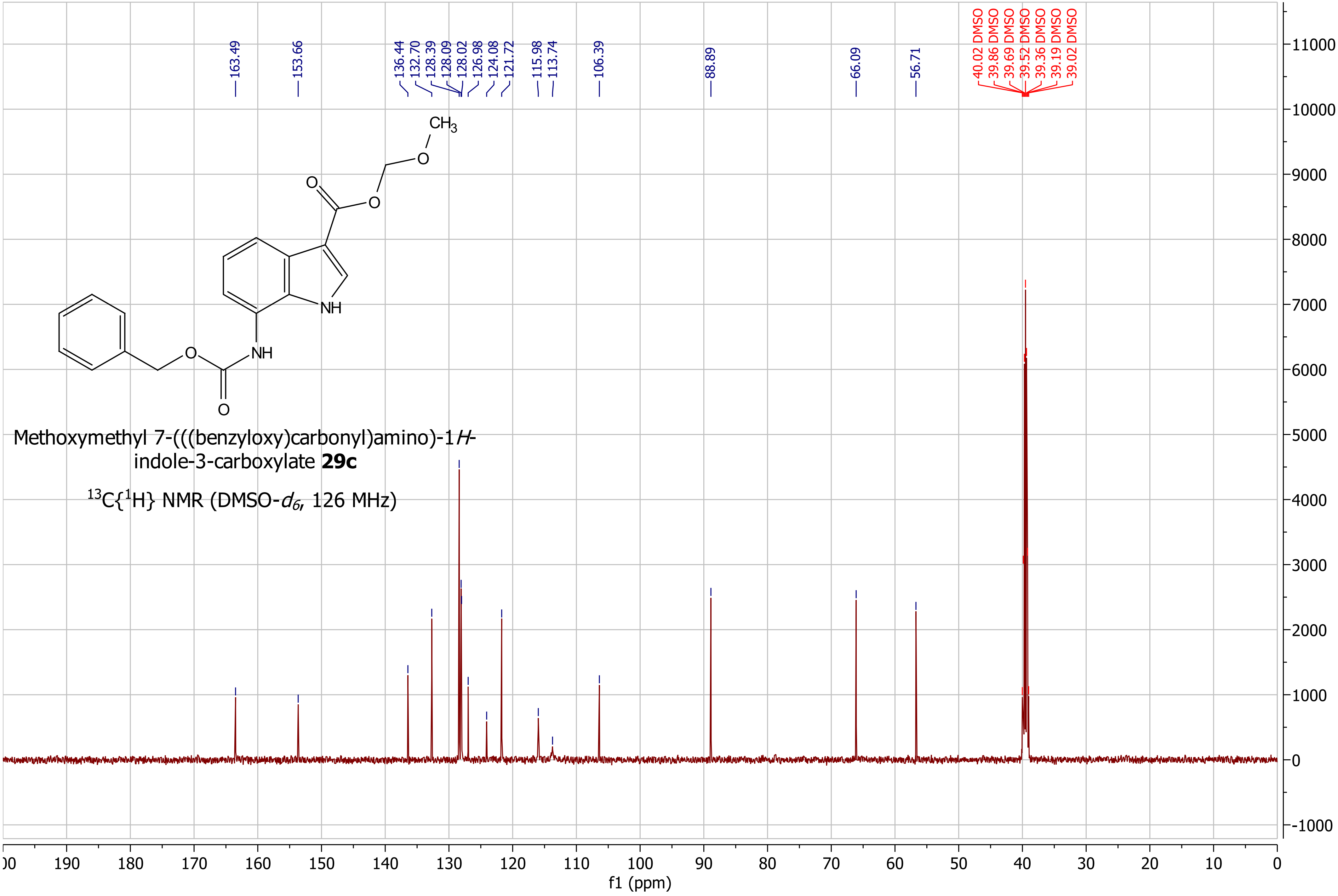

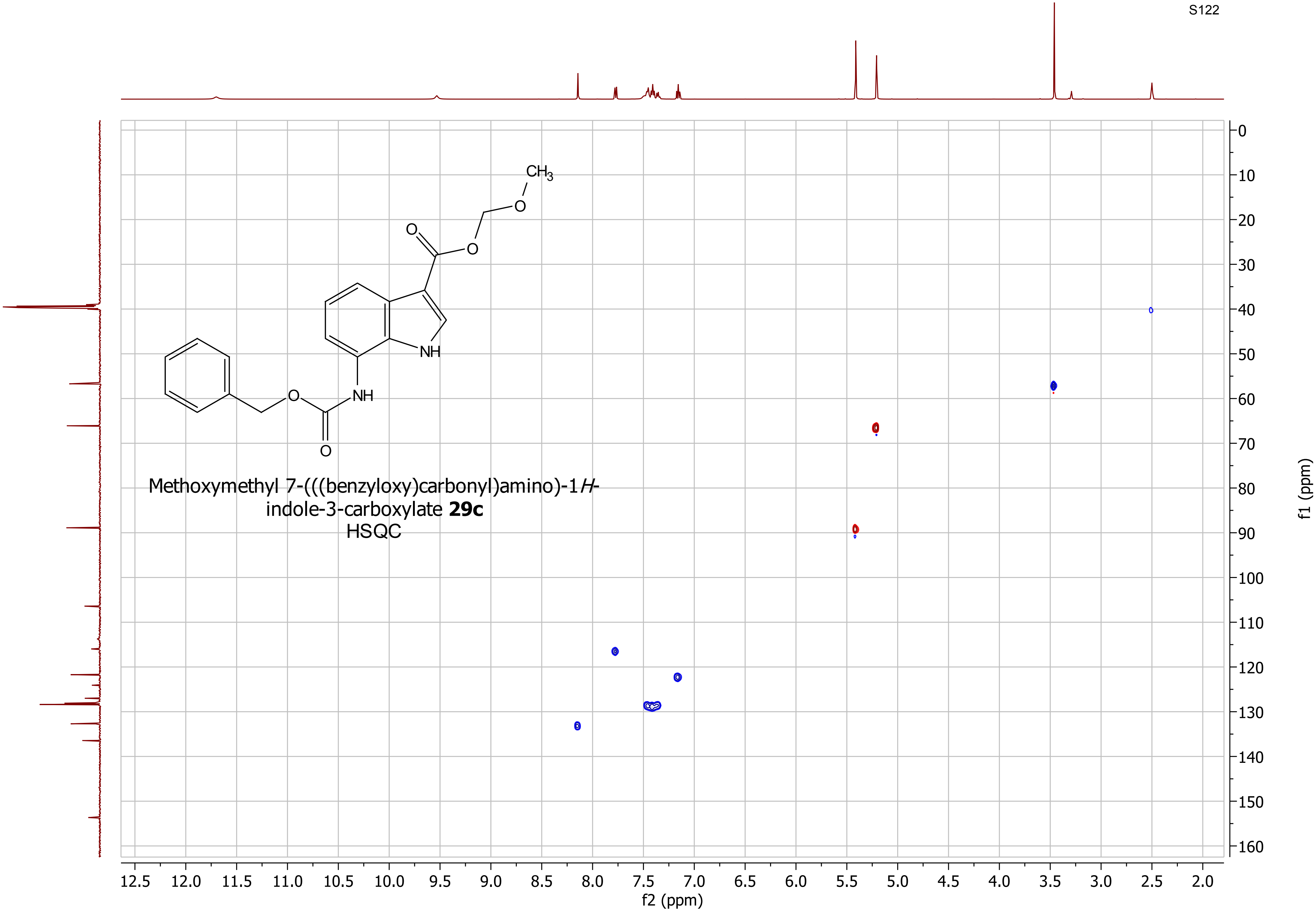
Methoxymethyl 7-(((benzyloxy)carbonyl)amino)-1*H*-indole-3-carboxylate **29c**
HSQC
f2 (ppm)
f1 (ppm)

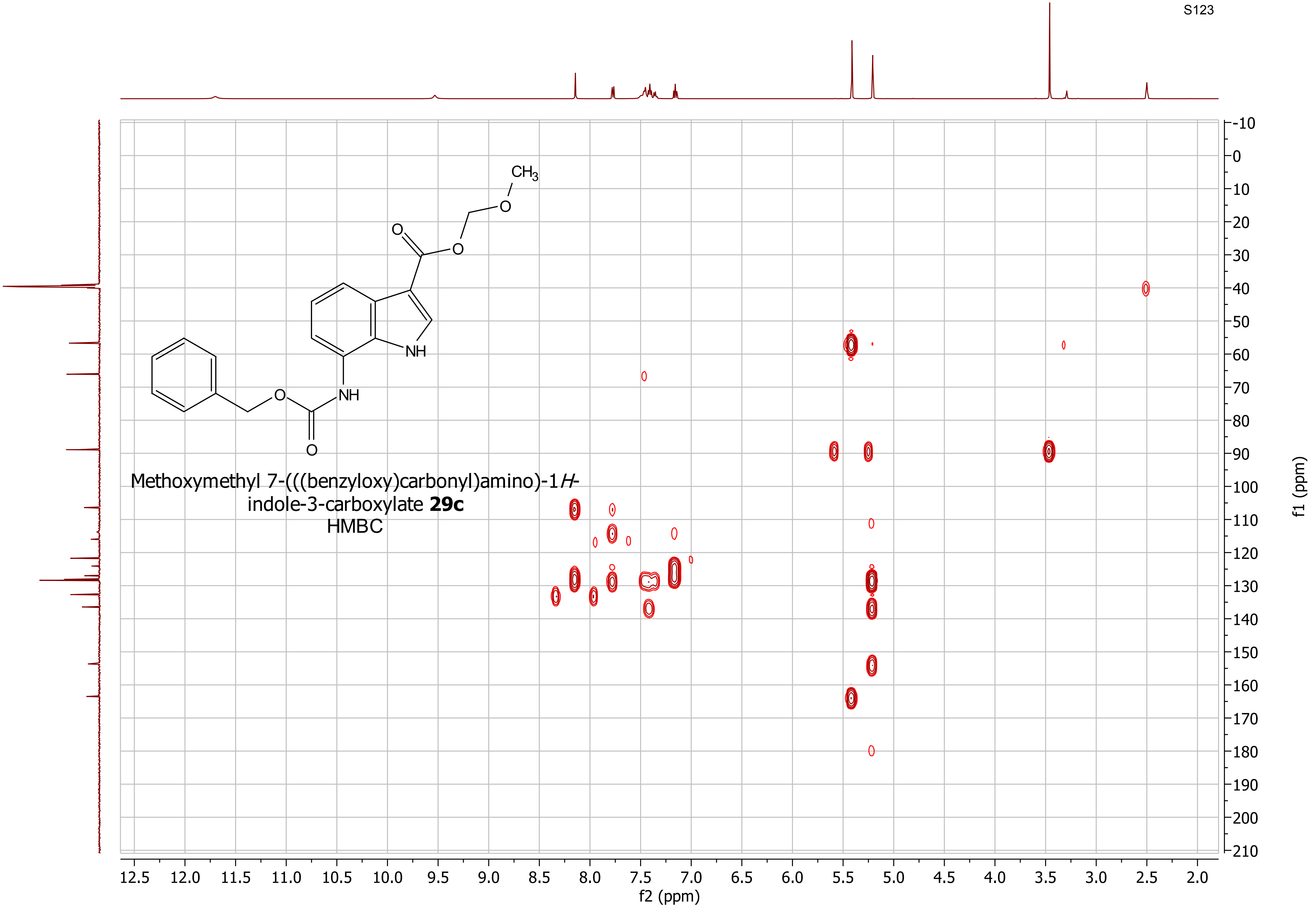

Methoxymethyl 7-(((benzyloxy)carbonyl)amino)-1*H*-indole-3-carboxylate **29c**
HMBC

^{1}H NMR (500 MHz, DMSO-d_6) δ 11.80 (s, 1H), 11.67 (s, 1H), 9.55 (s, 1H), 8.19 (s, 1H), 7.93 (d, J = 2.9 Hz, 1H), 7.44 (d, J = 7.0 Hz, 2H), 7.40 (t, J = 7.4 Hz, 2H), 7.36 – 7.30 (m, 2H), 7.26 (d, J = 8.7 Hz, 1H), 5.15 (s, 2H).

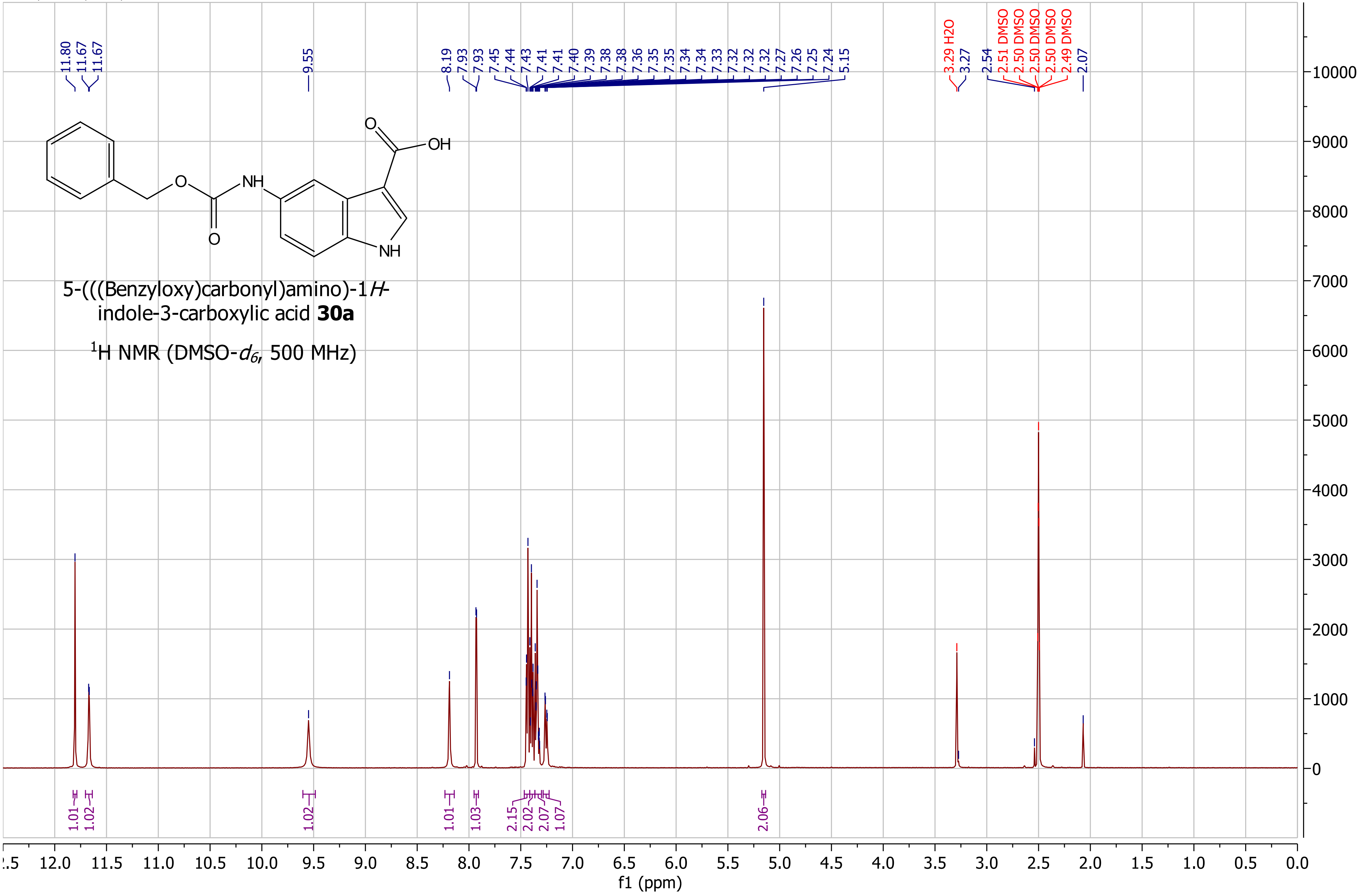

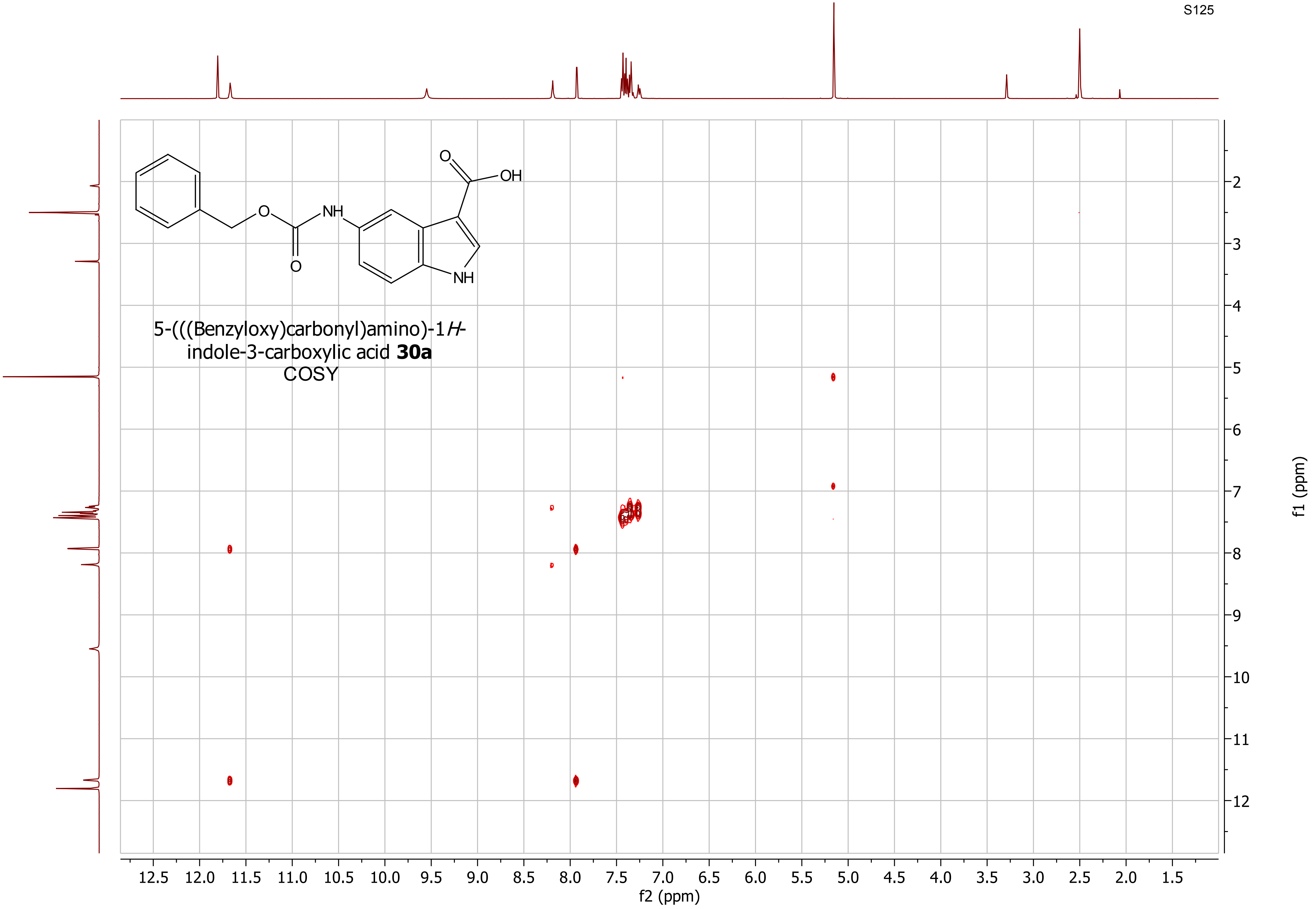

O
OH
NH
O
O
NH
5-(((Benzyloxy)carbonyl)amino)-1H-indole-3-carboxylic acid **30a**
COSY
12.5 12.0 11.5 11.0 10.5 10.0 9.5 9.0 8.5 8.0 7.5 7.0 6.5 6.0 5.5 5.0 4.5 4.0 3.5 3.0 2.5 2.0 1.5
f2 (ppm)
2 3 4 5 6 7 8 9 10 11 12
f1 (ppm)

^{13}C NMR (126 MHz, DMSO-d_6) δ 165.8, 153.6, 136.9, 132.9, 132.7, 132.6, 128.3, 127.9, 127.8, 126.1, 115.2, 112.0, 110.4, 107.2, 65.4.

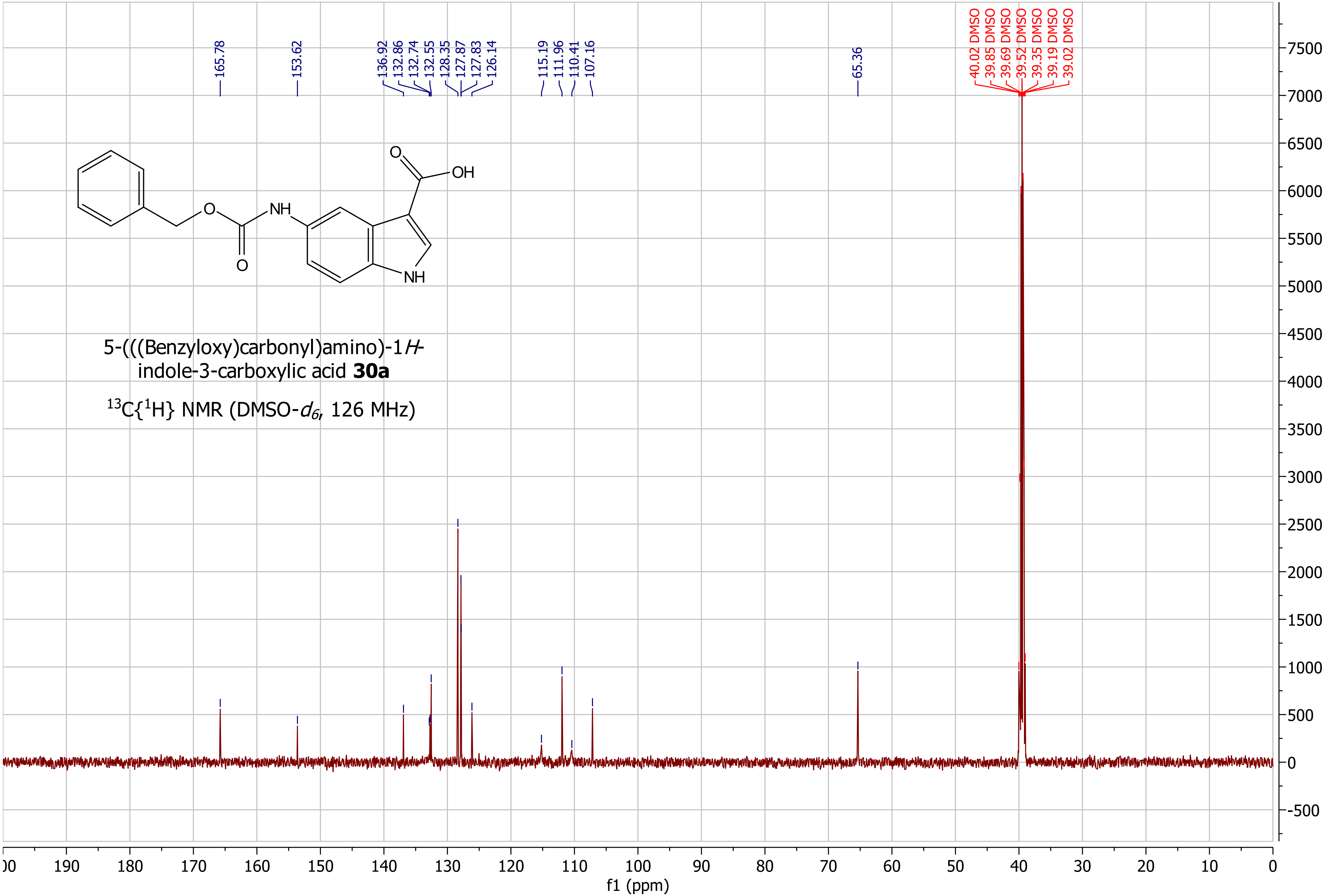

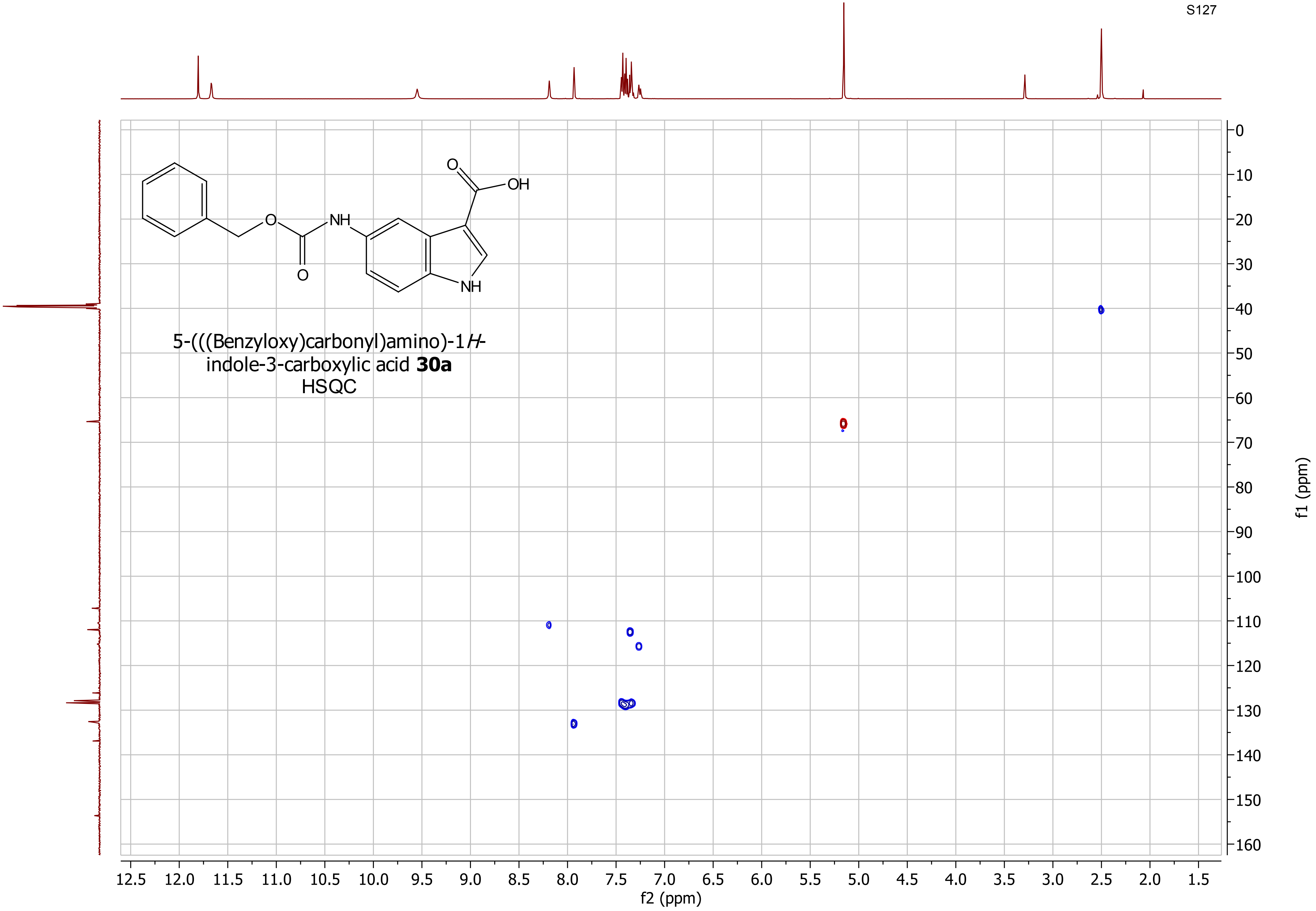
O
OH
O
NH
O
NH
5-(((Benzyloxy)carbonyl)amino)-1*H*-indole-3-carboxylic acid **30a**
HSQC
f1 (ppm)
f2 (ppm)
0
10
20
30
40
50
60
70
80
90
100
110
120
130
140
150
160
12.5
12.0
11.5
11.0
10.5
10.0
9.5
9.0
8.5
8.0
7.5
7.0
6.5
6.0
5.5
5.0
4.5
4.0
3.5
3.0
2.5
2.0
1.5

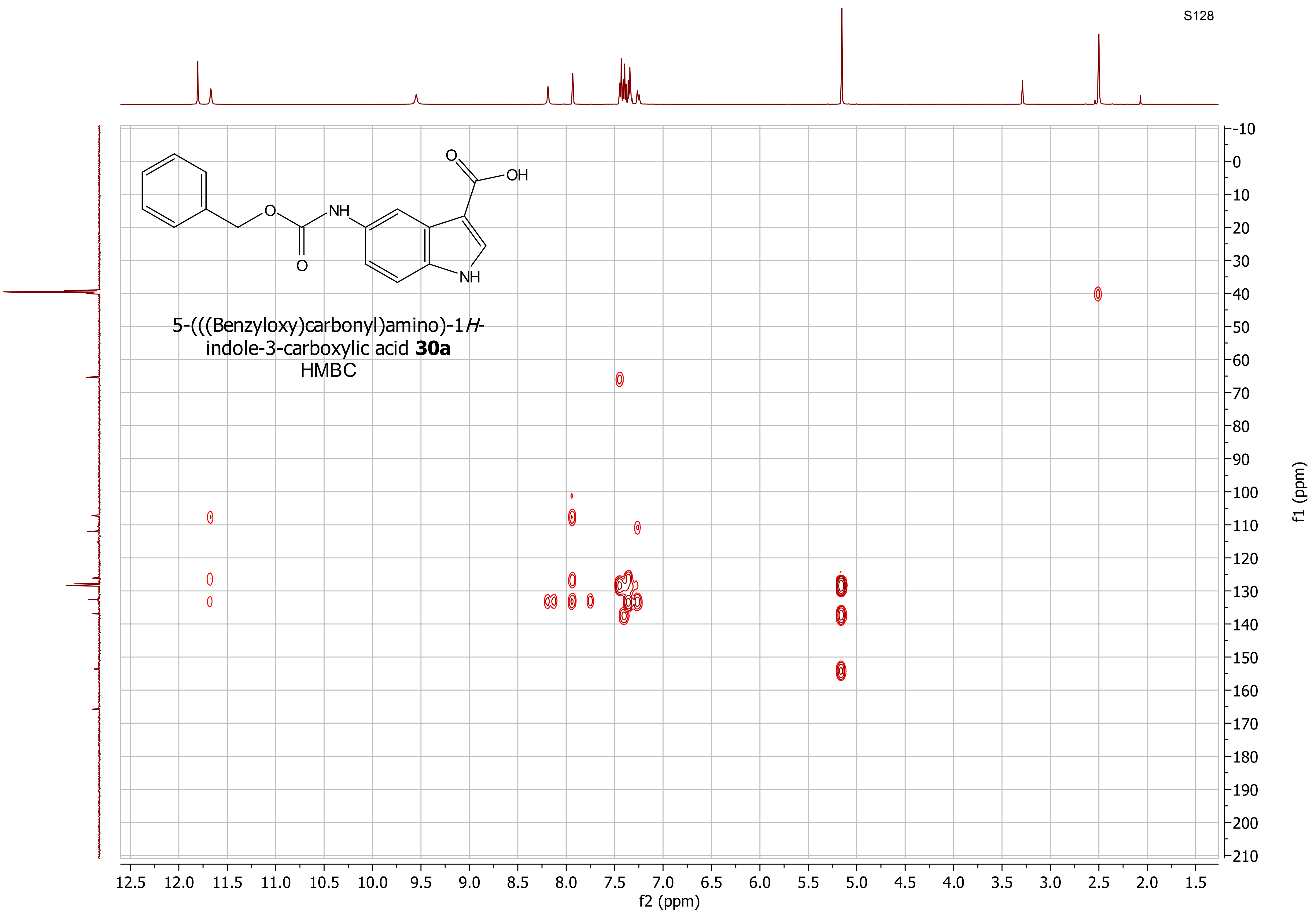
O
OH
O
NH
O
NH
5-(((Benzyloxy)carbonyl)amino)-1*H*-
indole-3-carboxylic acid **30a**
HMBC
f2 (ppm)
f1 (ppm)

^{1}H NMR (500 MHz, DMSO-d_6) δ 11.81 (s, 1H), 11.64 (d, J = 3.0 Hz, 1H), 9.70 (s, 1H), 7.89 (d, J = 2.8 Hz, 1H), 7.84 (d, J = 8.6 Hz, 1H), 7.79 (s, 1H), 7.44 (d, J = 7.0 Hz, 2H), 7.40 (t, J = 7.4 Hz, 2H), 7.37 – 7.31 (m, 1H), 7.16 (dd, J = 8.6, 1.9 Hz, 1H), 5.16 (s, 2H).

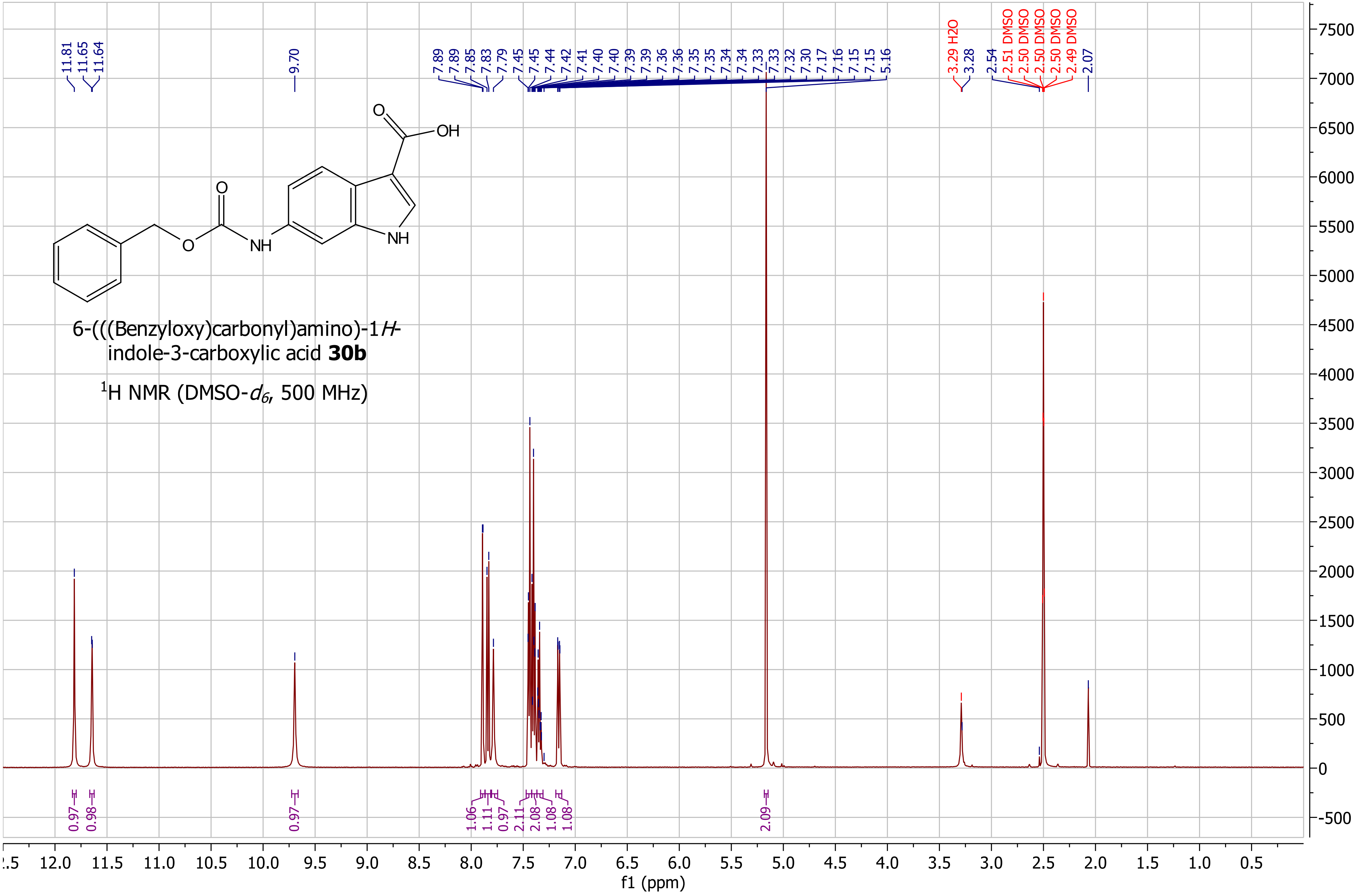

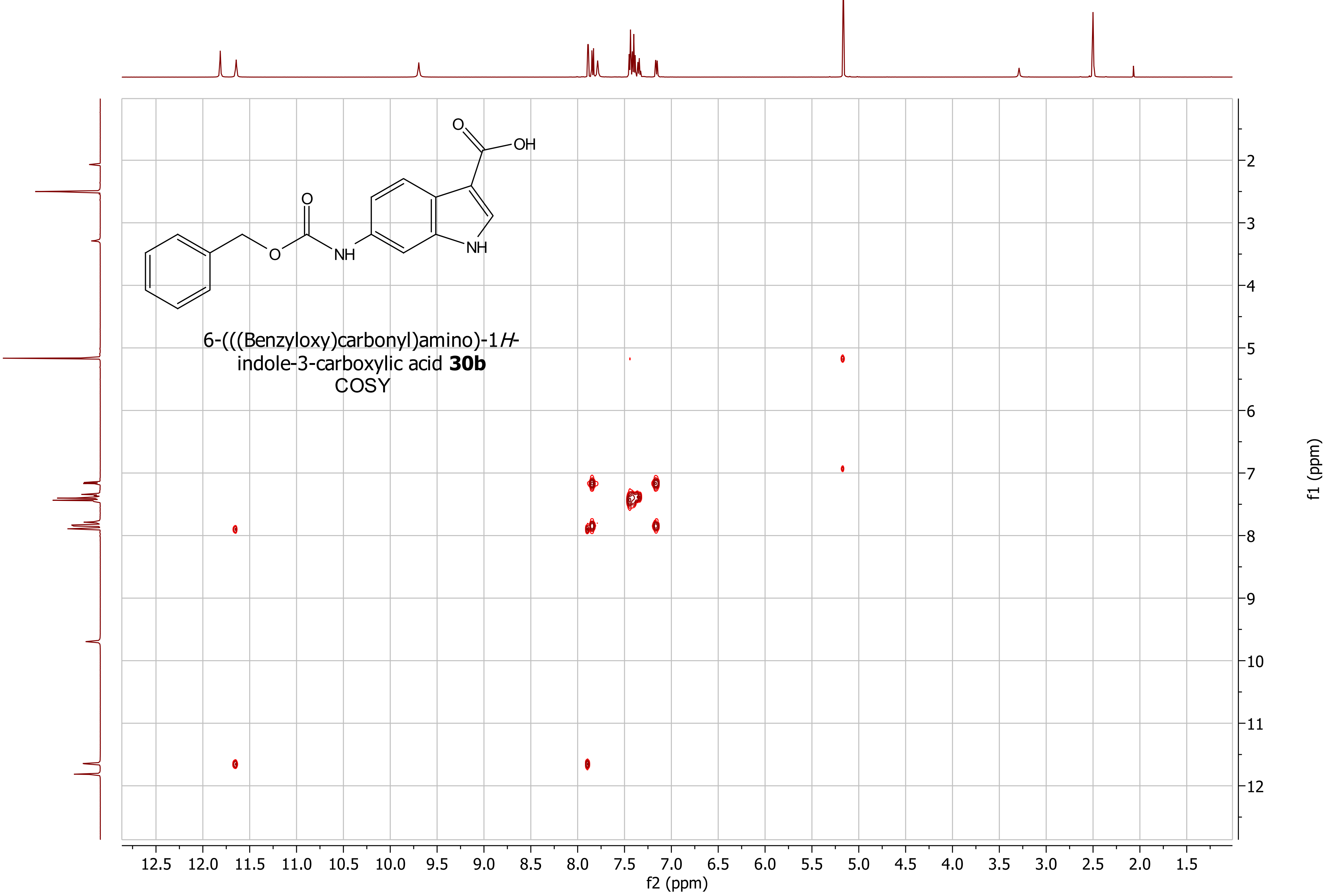

O
OH
O
NH
O
NH
6-(((Benzyloxy)carbonyl)amino)-1*H*-indole-3-carboxylic acid **30b**
COSY
12.5 12.0 11.5 11.0 10.5 10.0 9.5 9.0 8.5 8.0 7.5 7.0 6.5 6.0 5.5 5.0 4.5 4.0 3.5 3.0 2.5 2.0 1.5
f2 (ppm)
2 3 4 5 6 7 8 9 10 11 12
f1 (ppm)

^{13}C NMR (126 MHz, DMSO-d_6) δ 165.8, 153.5, 136.7, 136.7, 134.2, 131.6, 128.4, 128.0, 127.9, 121.6, 120.4, 113.5, 107.3, 101.4, 65.5.

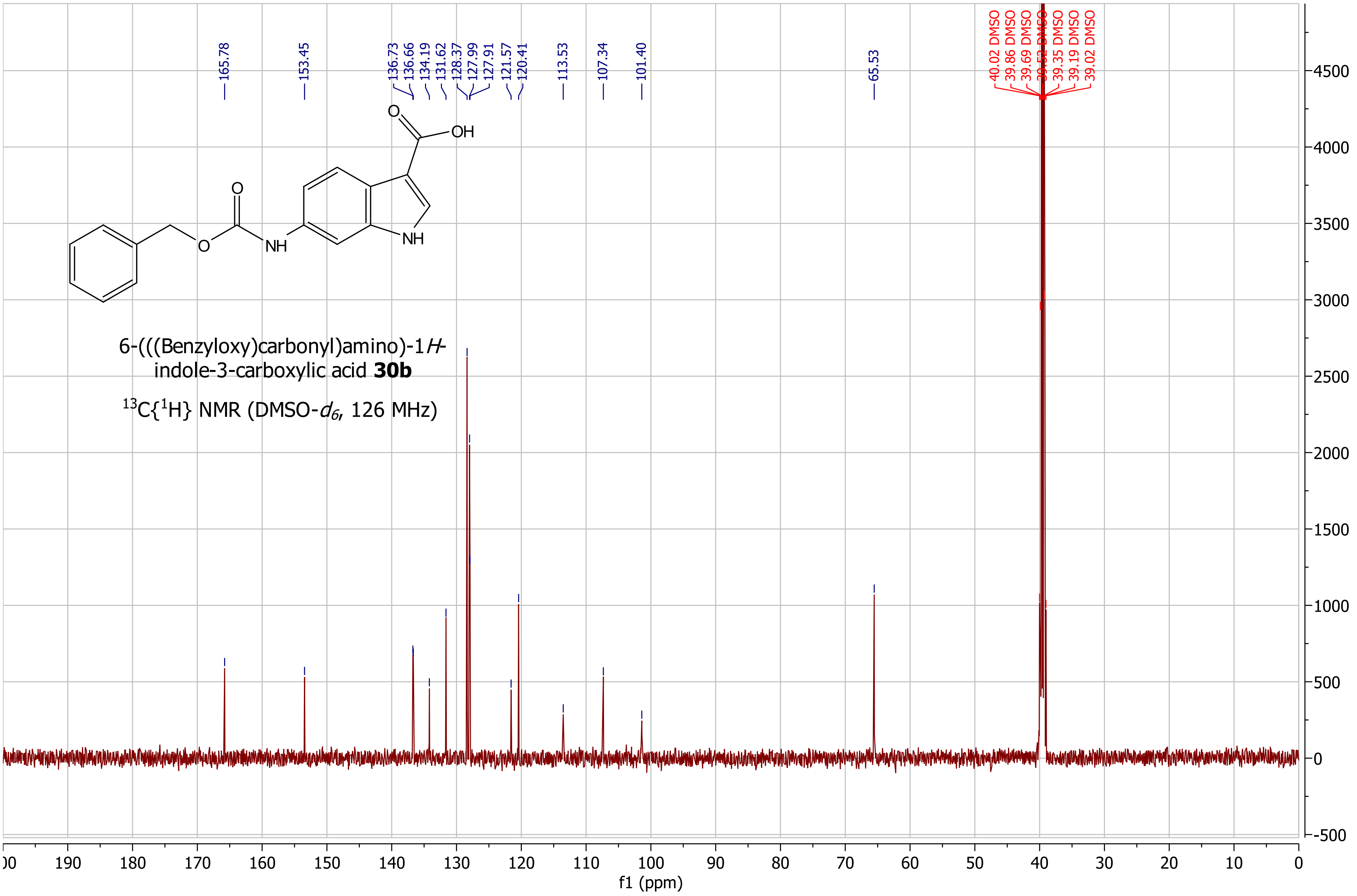

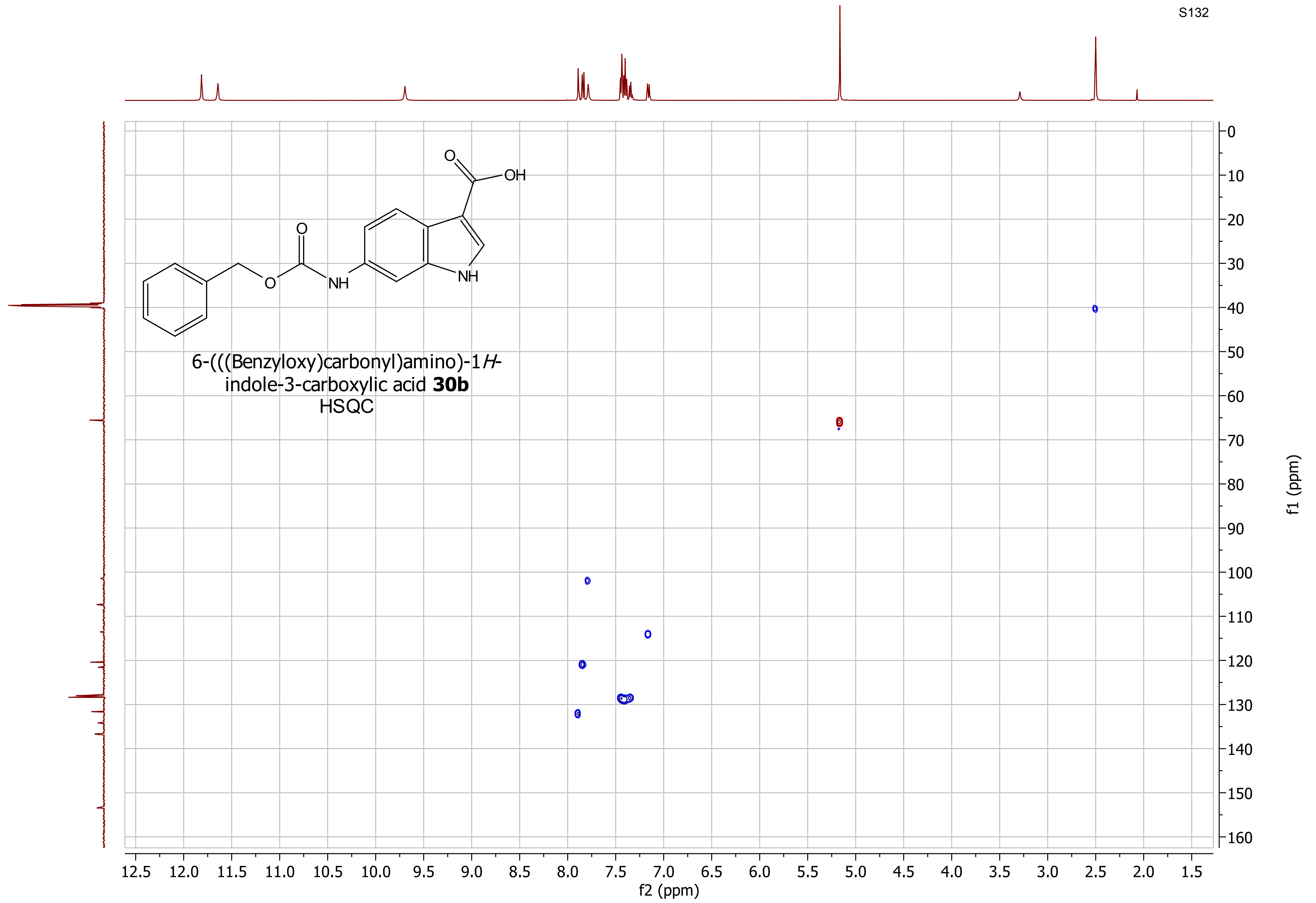
O
OH
O
O
NH
NH
6-(((Benzyloxy)carbonyl)amino)-1H-indole-3-carboxylic acid 30b
HSQC
f1 (ppm)
f2 (ppm)
12.5 12.0 11.5 11.0 10.5 10.0 9.5 9.0 8.5 8.0 7.5 7.0 6.5 6.0 5.5 5.0 4.5 4.0 3.5 3.0 2.5 2.0 1.5
0 10 20 30 40 50 60 70 80 90 100 110 120 130 140 150 160

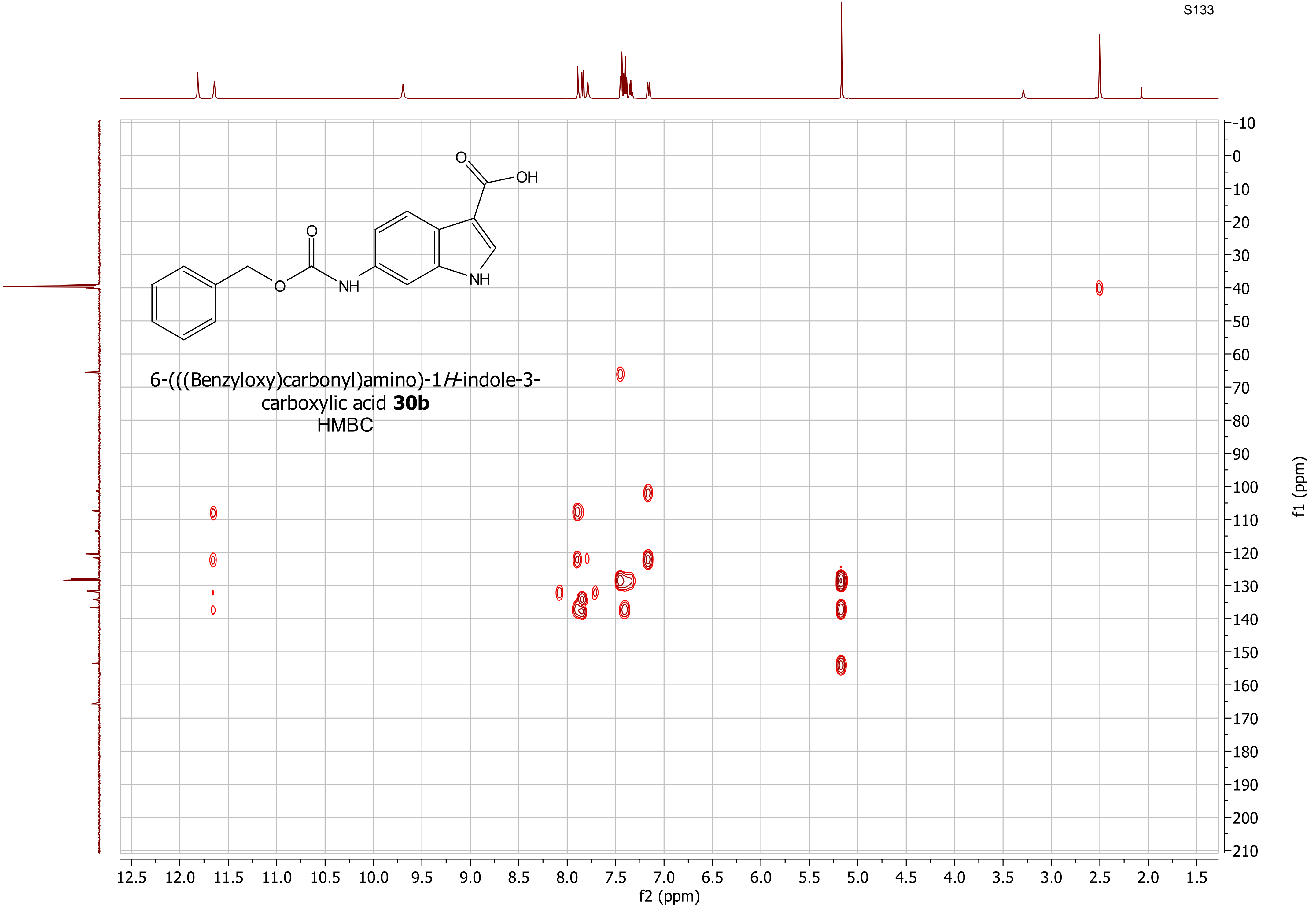
6-(((Benzyloxy)carbonyl)amino)-1H-indole-3-carboxylic acid 30b
HMBC
O
OH
NH
O
NH
O
f2 (ppm)
f1 (ppm)
12.5 12.0 11.5 11.0 10.5 10.0 9.5 9.0 8.5 8.0 7.5 7.0 6.5 6.0 5.5 5.0 4.5 4.0 3.5 3.0 2.5 2.0 1.5
-10 0 10 20 30 40 50 60 70 80 90 100 110 120 130 140 150 160 170 180 190 200 210

^{1}H NMR (500 MHz, DMSO-d_6) δ 11.91 (s, 1H), 11.49 (s, 1H), 9.49 (s, 1H), 7.98 (d, J = 3.0 Hz, 1H), 7.76 (d, J = 7.9 Hz, 1H), 7.49 – 7.33 (m, 6H), 7.10 (t, J = 7.9 Hz, 1H), 5.20 (s, 2H).

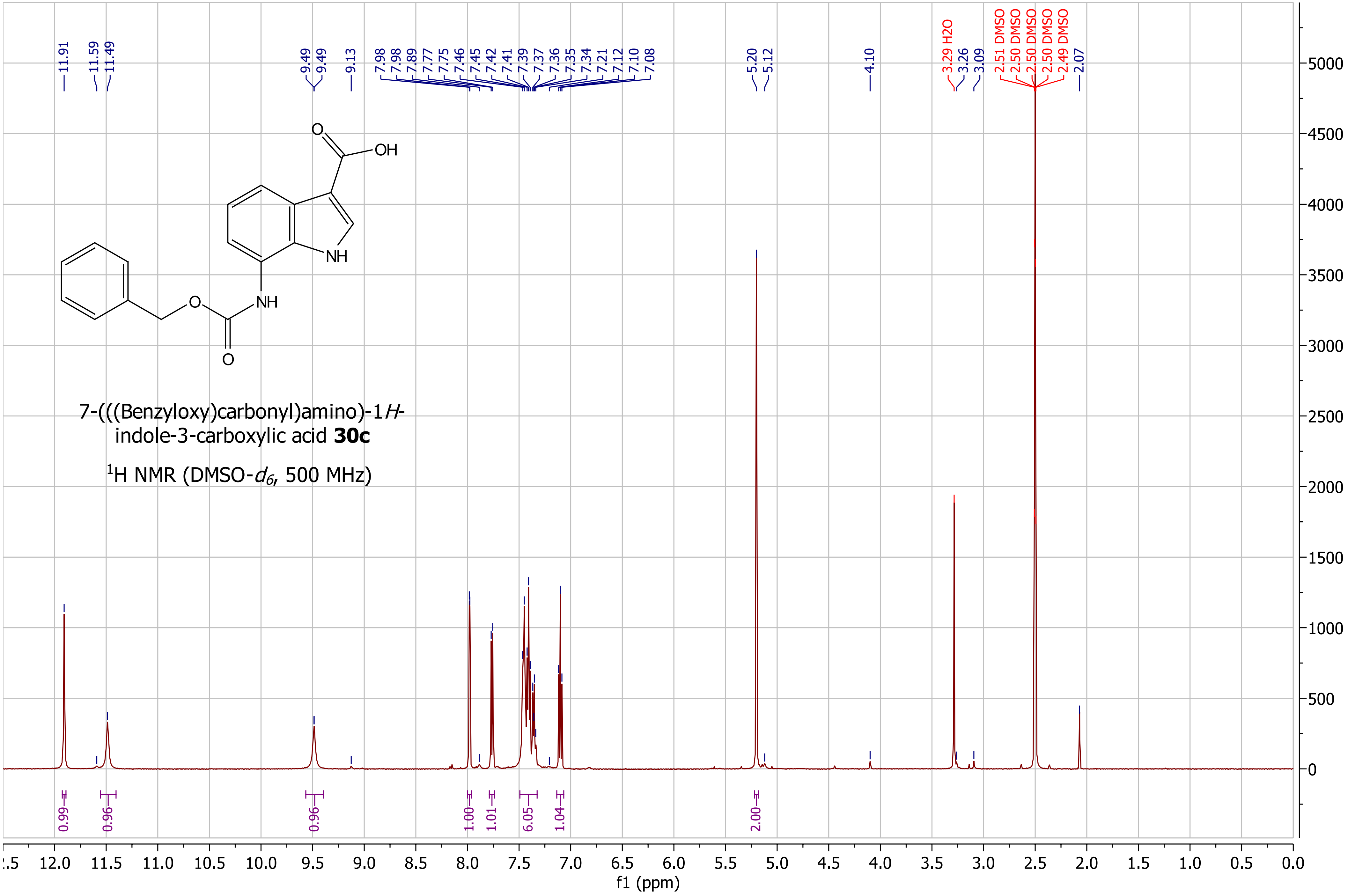

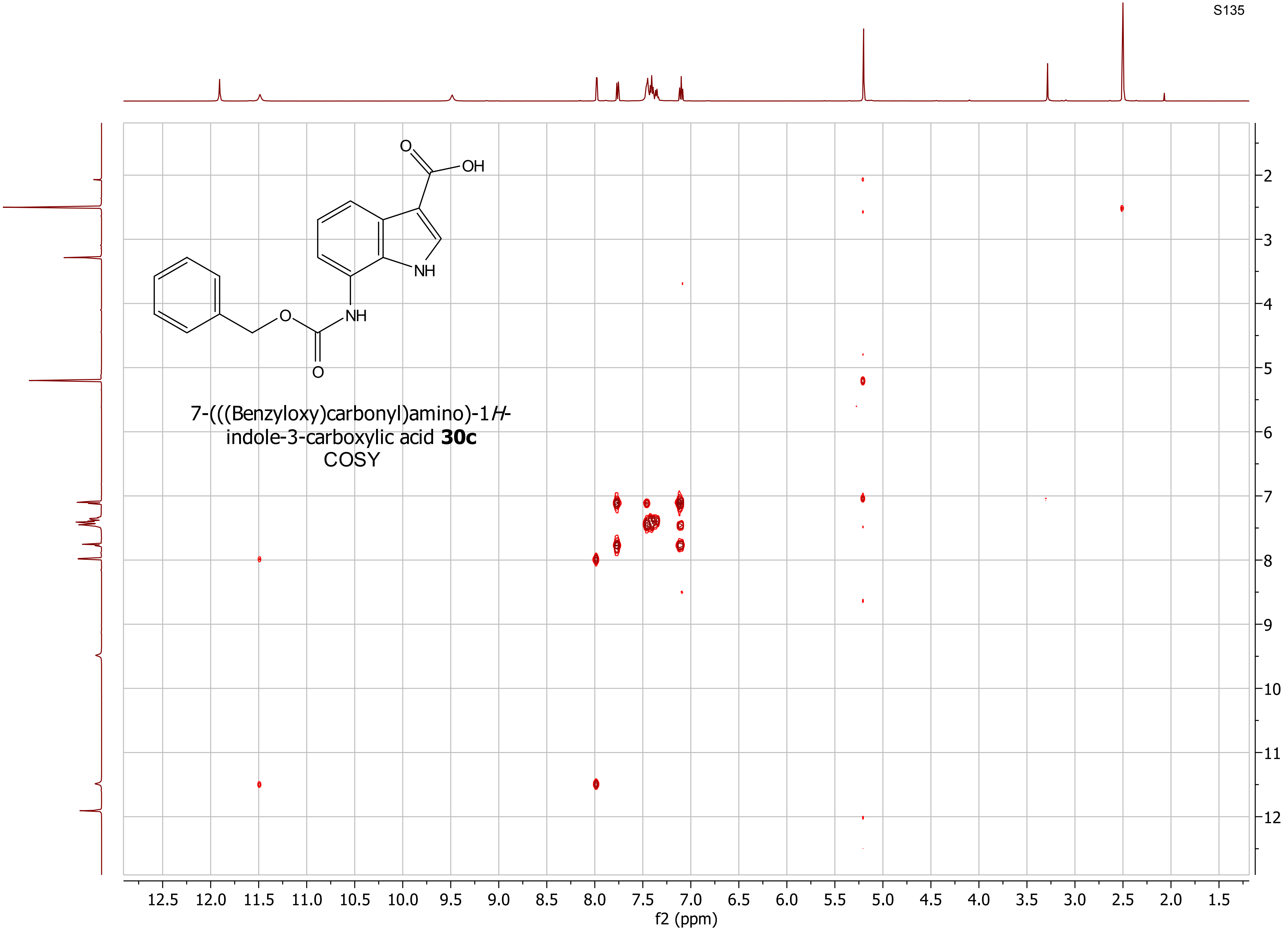

7-(((Benzyloxy)carbonyl)amino)-1*H*-indole-3-carboxylic acid **30c**
COSY

^{13}C NMR (126 MHz, DMSO-d_6) δ 165.7, 153.7, 136.5, 131.9, 128.4, 128.1, 128.0, 127.3, 123.8, 121.2, 116.2, 107.7, 66.0.

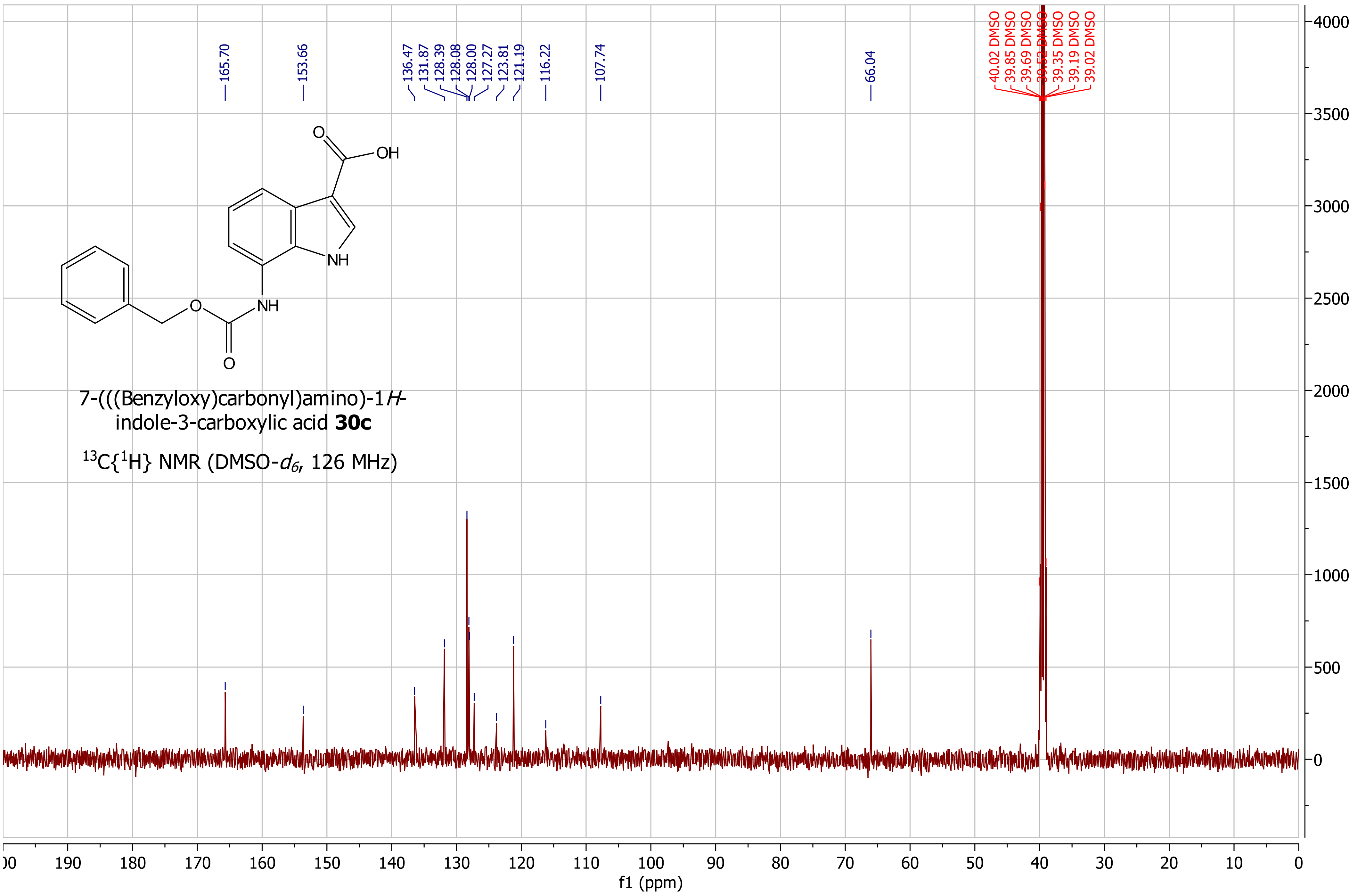

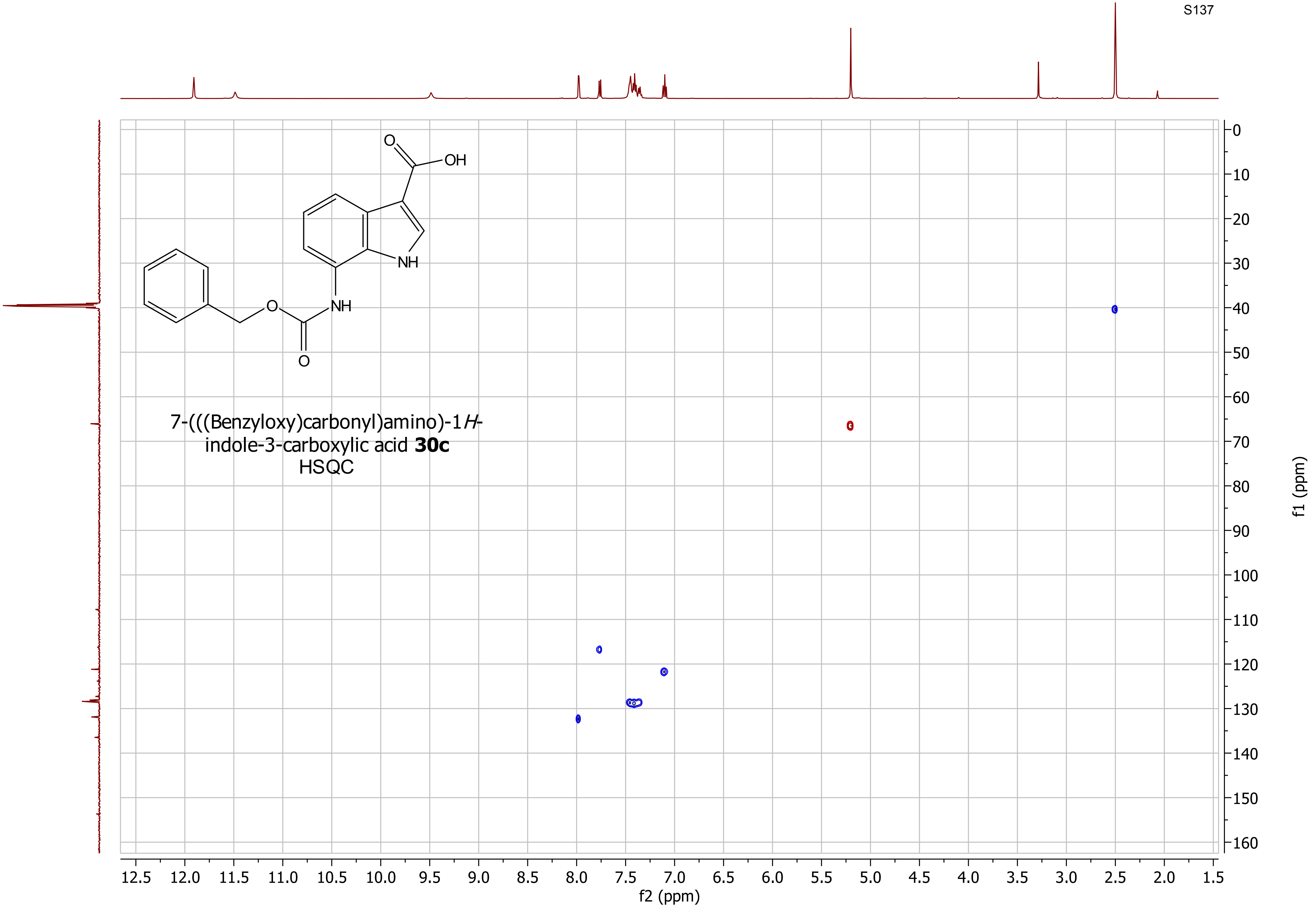

7-(((Benzyloxy)carbonyl)amino)-1*H*-indole-3-carboxylic acid **30c**
HSQC
O
OH
NH
O
NH
O
f2 (ppm)
f1 (ppm)
12.5 12.0 11.5 11.0 10.5 10.0 9.5 9.0 8.5 8.0 7.5 7.0 6.5 6.0 5.5 5.0 4.5 4.0 3.5 3.0 2.5 2.0 1.5
0 10 20 30 40 50 60 70 80 90 100 110 120 130 140 150 160

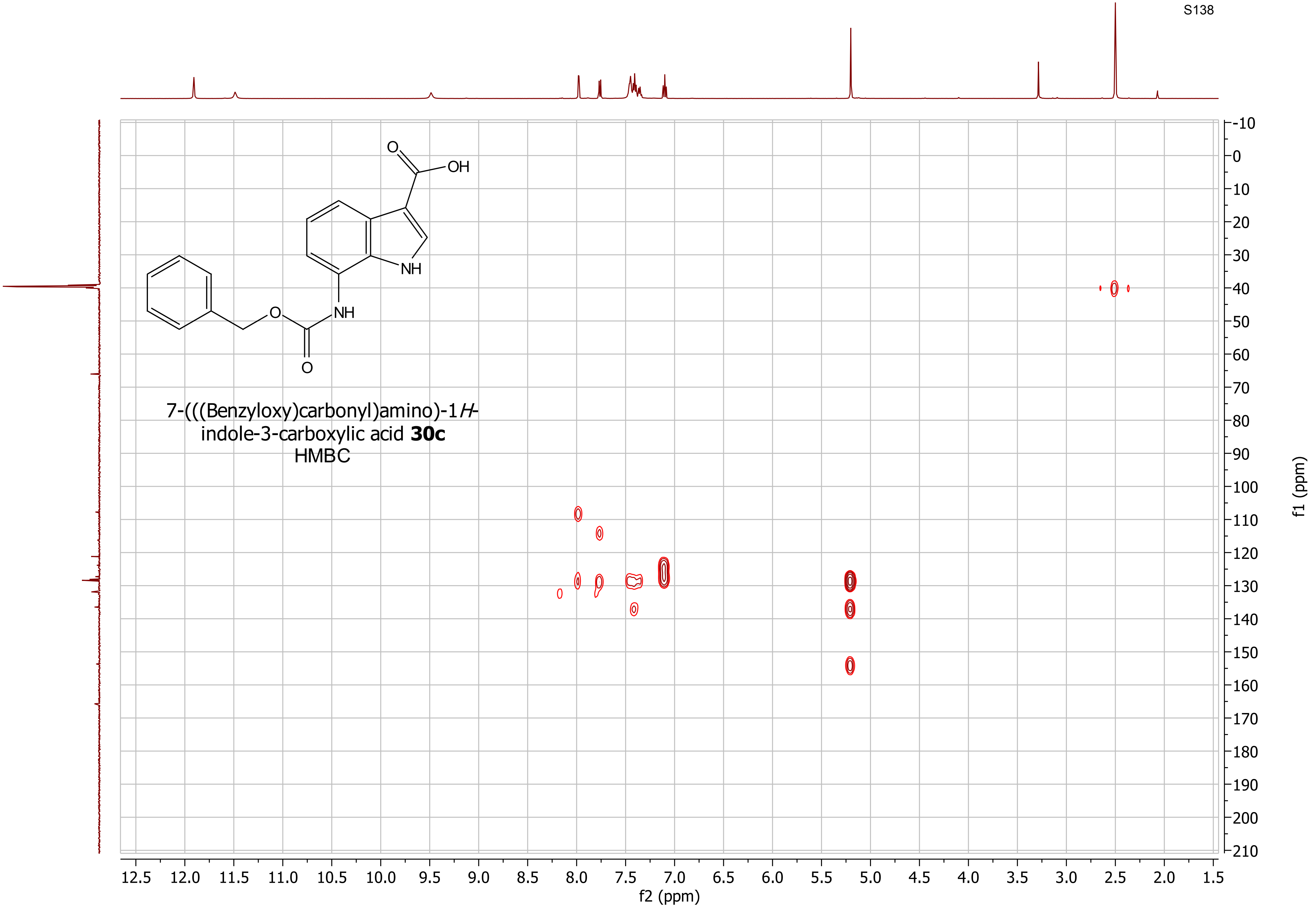
O
OH
NH
O
NH
O
7-(((Benzyloxy)carbonyl)amino)-1H-indole-3-carboxylic acid **30c**
HMBC
f1 (ppm)
f2 (ppm)

^{1}H NMR (500 MHz, DMSO-d_6) δ 10.60 (s, 1H), 9.39 (s, 1H), 8.88 (s, 1H), 7.79 (s, 1H), 7.46 – 7.37 (m, 4H), 7.37 – 7.26 (m, 2H), 7.24 – 7.14 (m, 1H), 7.12 – 7.02 (m, 1H), 5.15 (s, 2H), 1.48 (s, 9H).

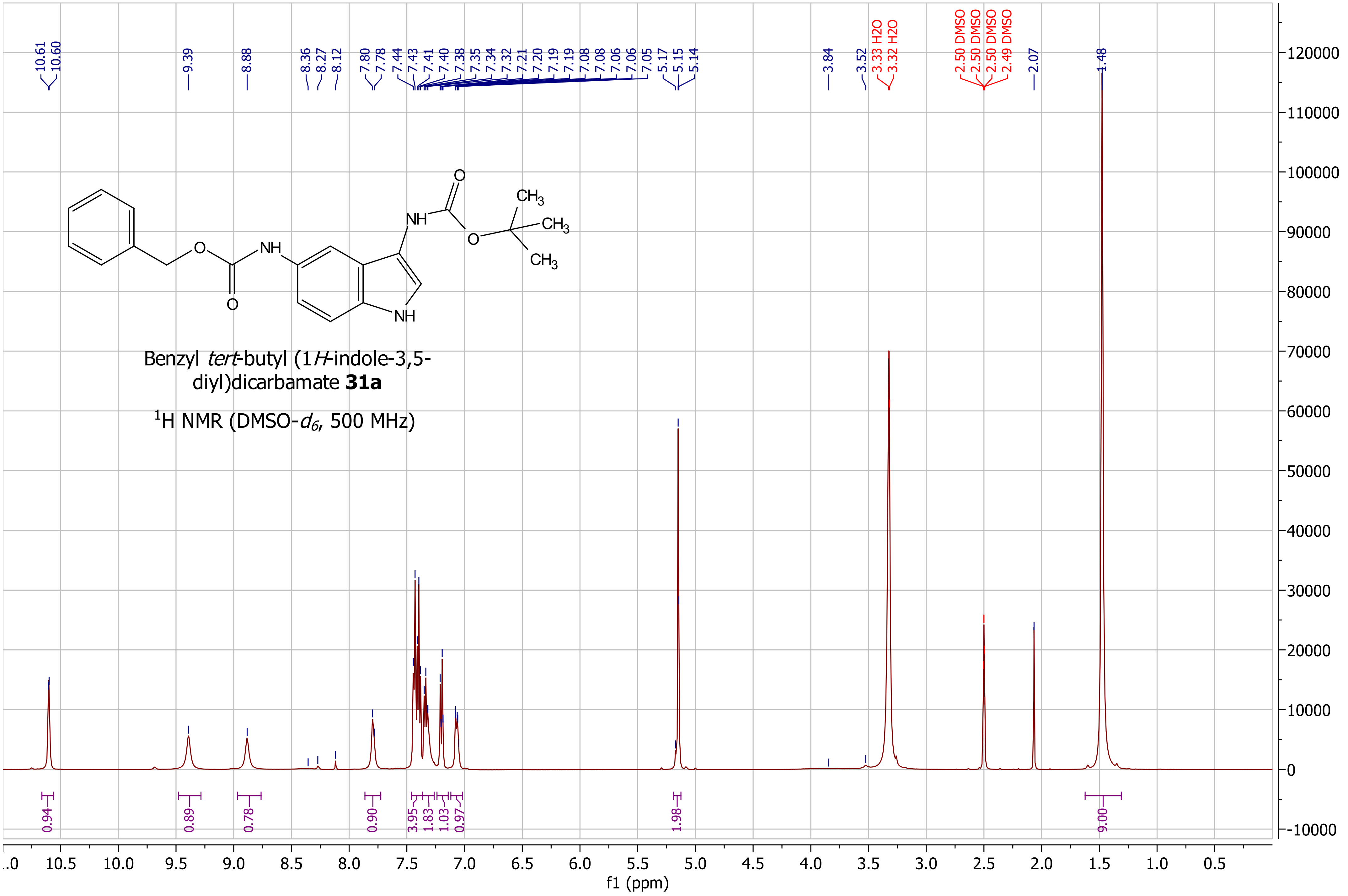

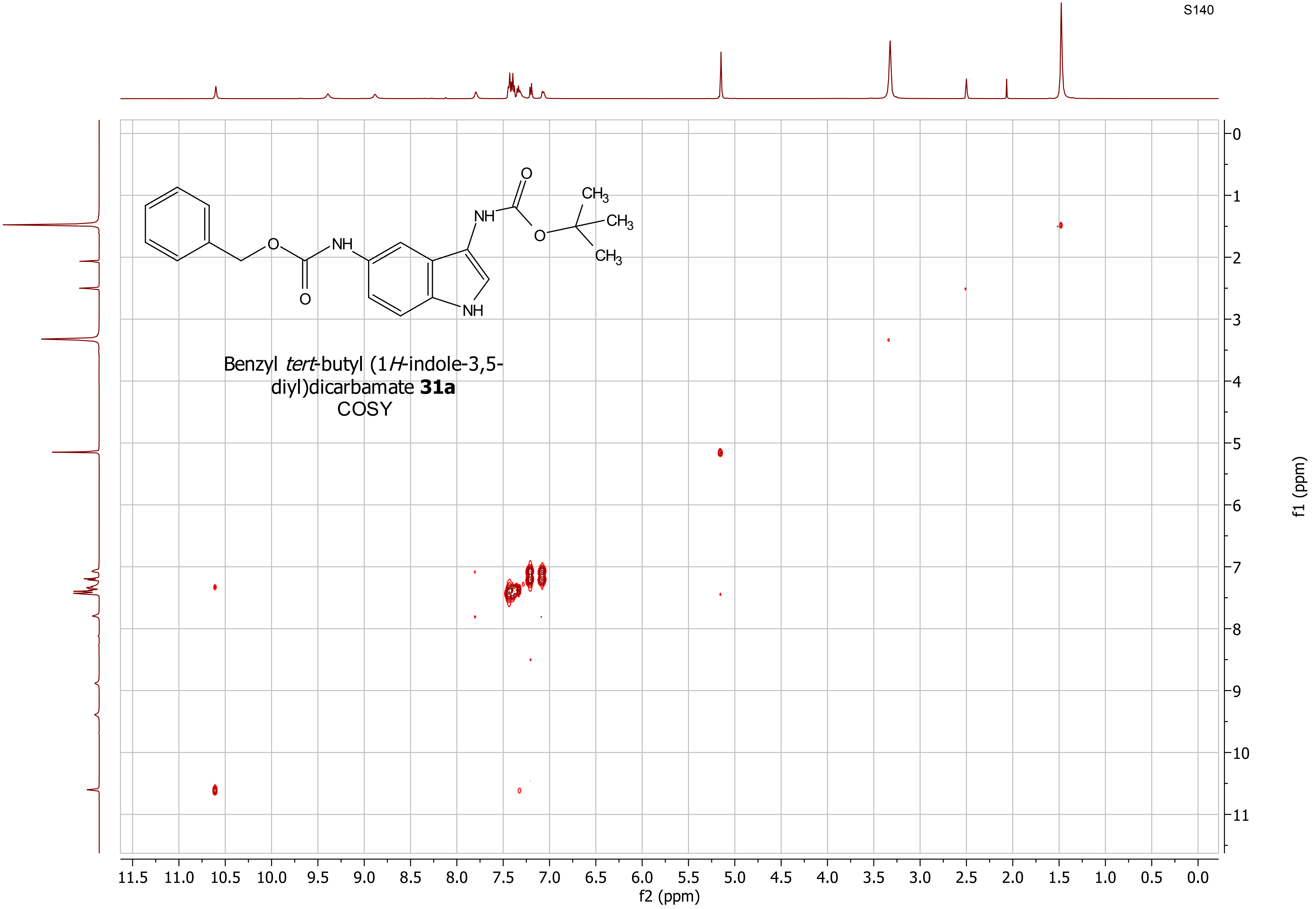

Benzyl *tert*-butyl (1*H*-indole-3,5-diyl)dicarbamate **31a**
COSY
NH
O
O
NH
O
O
CH_3
CH_3
CH_3
NH
f2 (ppm)
f1 (ppm)

^{13}C NMR (126 MHz, DMSO-d_6) δ 153.8, 137.0, 130.8, 130.0, 128.4, 127.9, 127.8, 121.6, 116.5, 115.2, 114.9, 111.1, 108.8, 78.1, 65.3, 28.2.

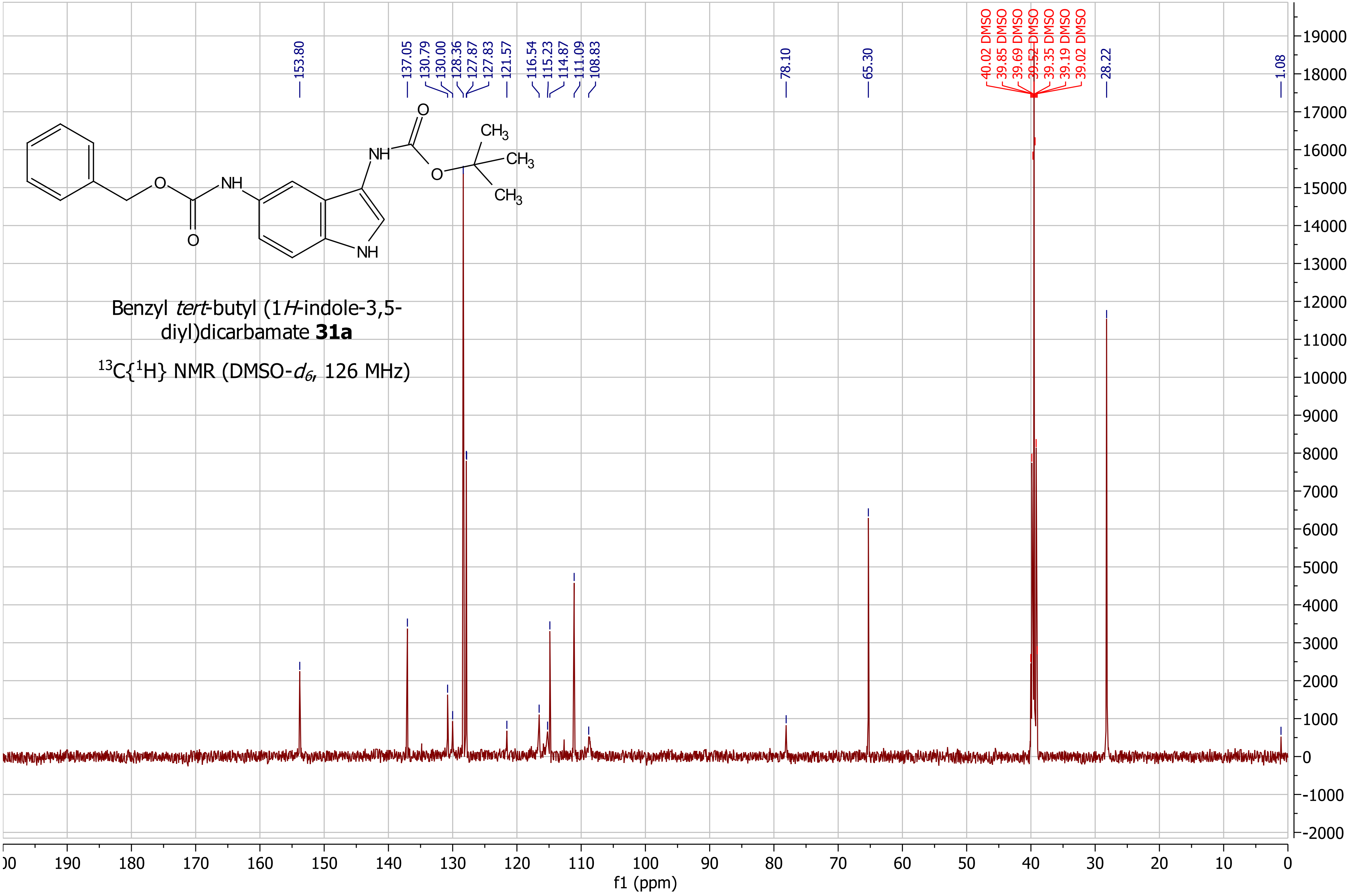

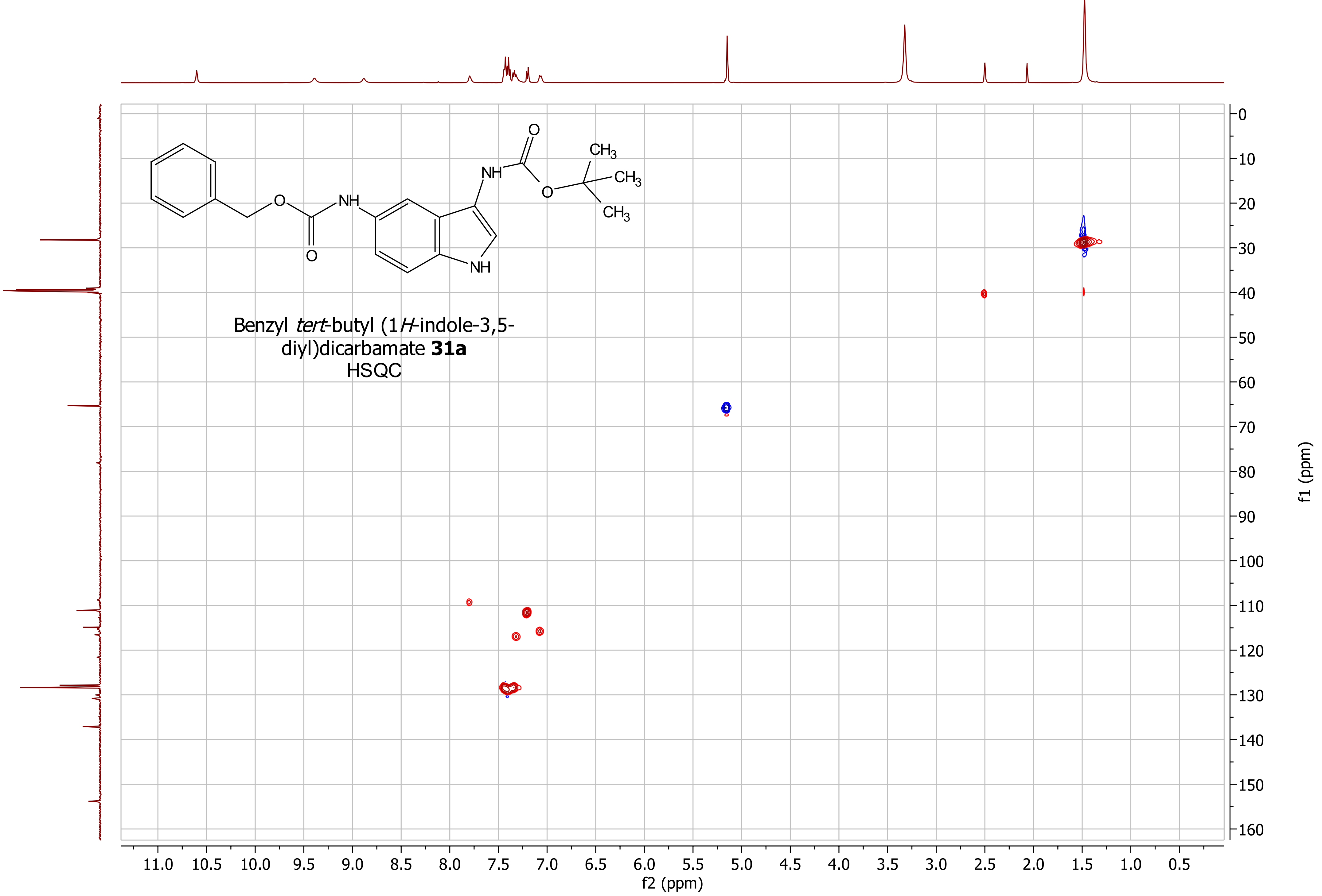

Benzyl *tert*-butyl (1*H*-indole-3,5-diyl)dicarbamate **31a**
HSQC
f2 (ppm)
f1 (ppm)

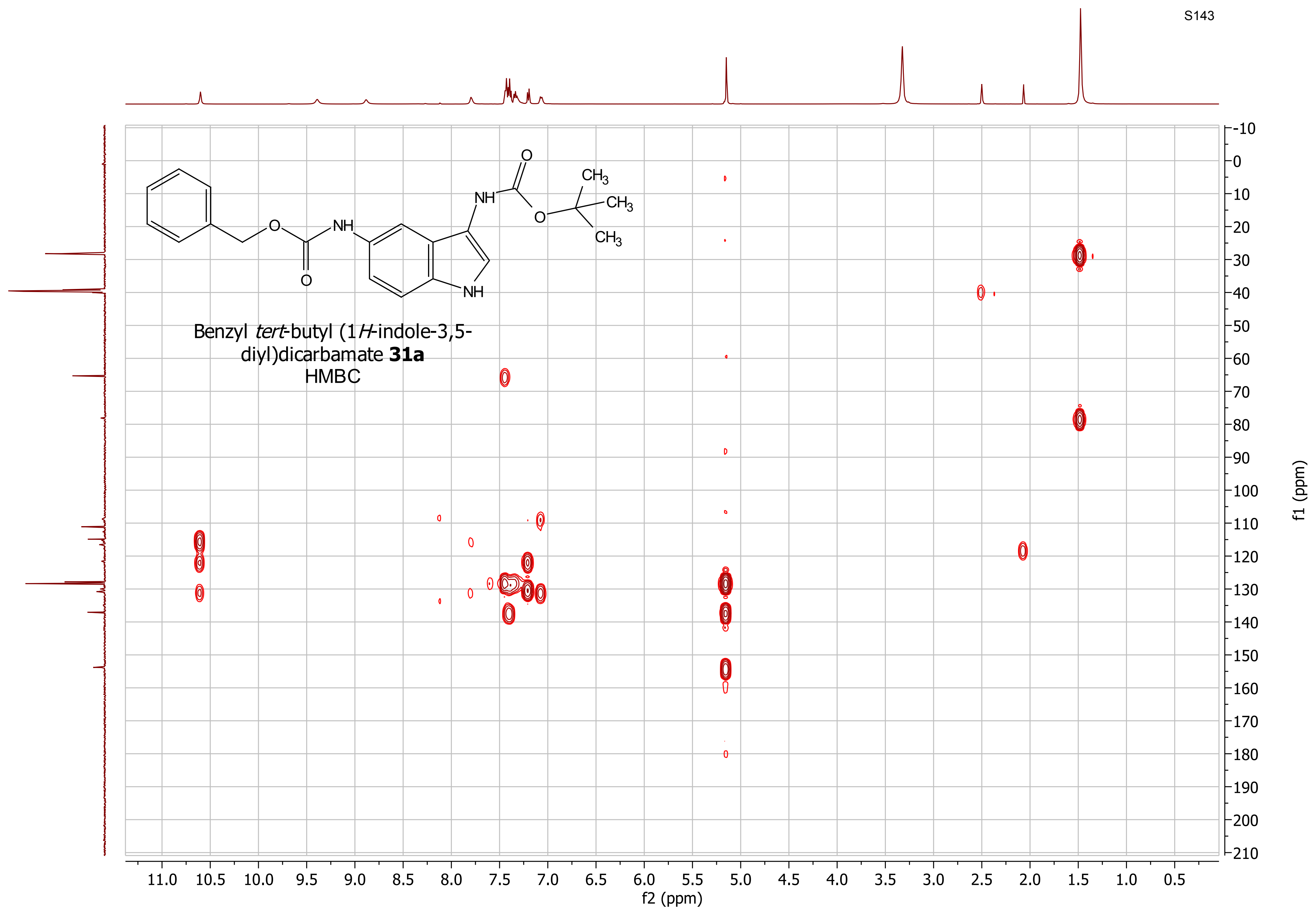
O
NH
O
CH3
CH3
CH3
O
NH
O
NH
Benzyl tert-butyl (1H-indole-3,5-diyl)dicarbamate 31a
HMBC
f2 (ppm)
f1 (ppm)

^{1}H NMR (500 MHz, DMSO-d_6) δ 10.53 (s, 1H), 9.55 (s, 1H), 8.99 (s, 1H), 7.57 (t, J = 7.2 Hz, 2H), 7.47 – 7.37 (m, 4H), 7.37 – 7.31 (m, 1H), 7.29 (s, 1H), 6.95 (dd, J = 8.6, 1.9 Hz, 1H), 5.15 (s, 2H), 1.48 (s, 9H).

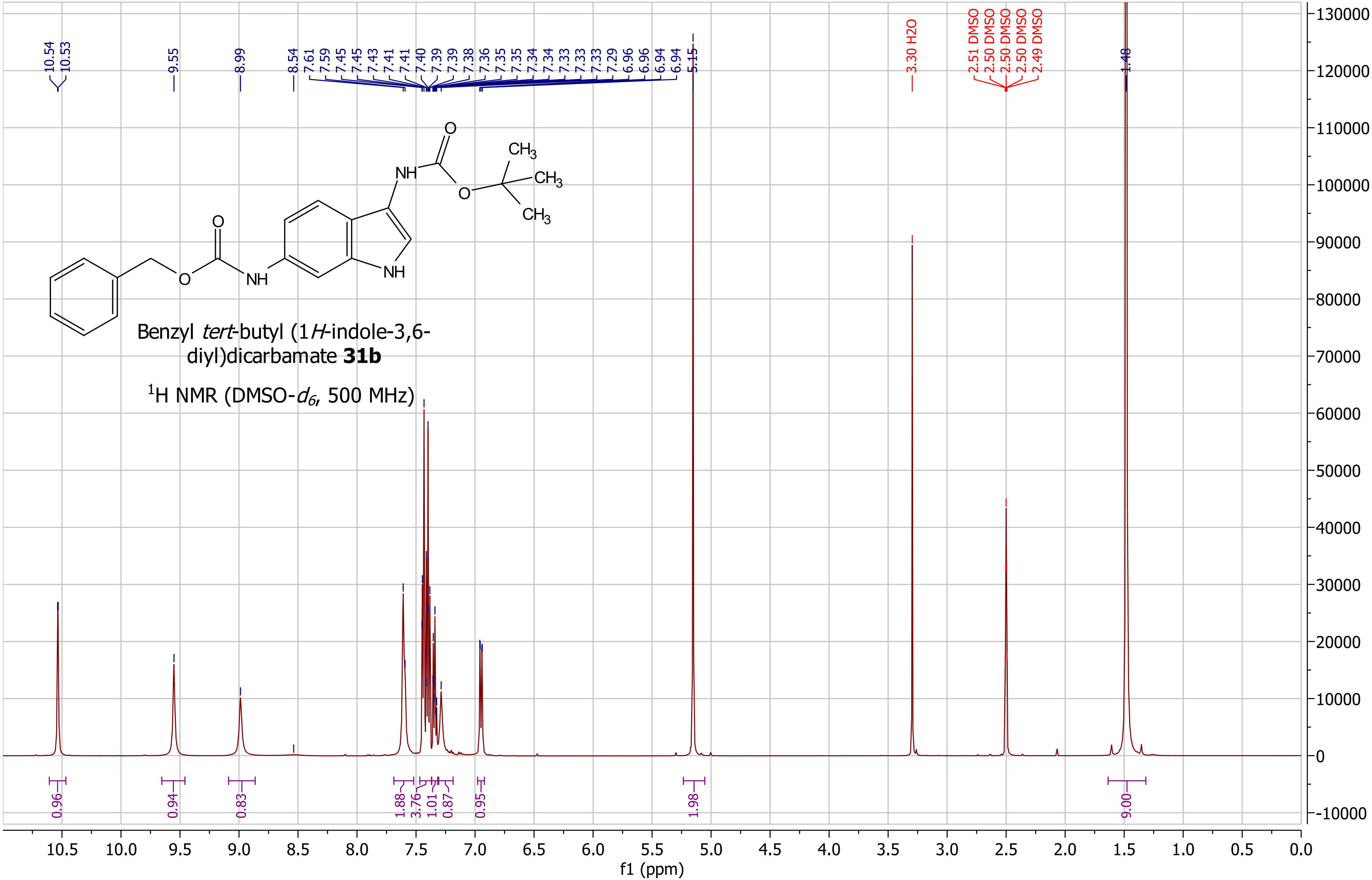

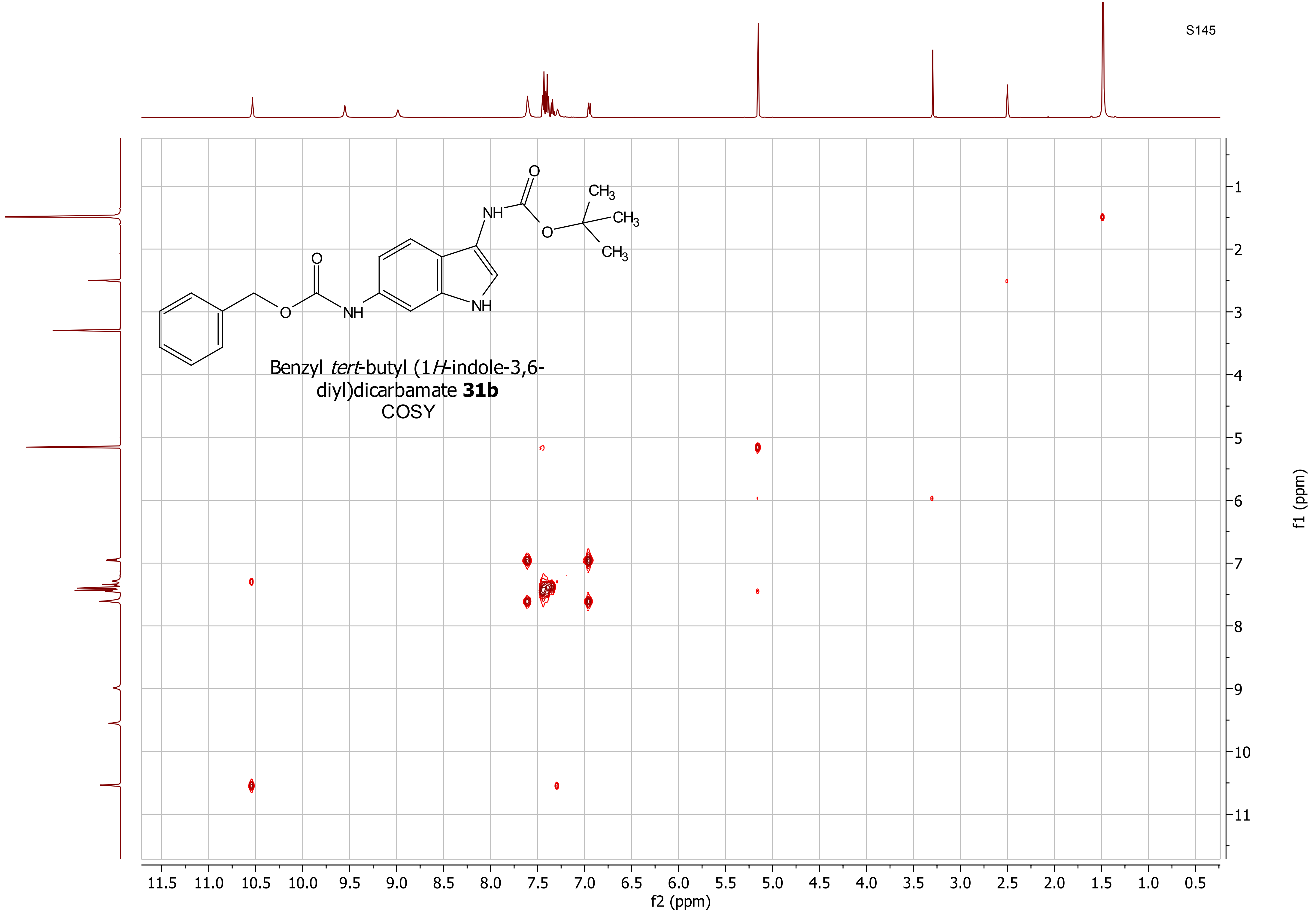
Benzyl *tert*-butyl (1*H*-indole-3,6-diyl)dicarbamate **31b**
COSY
f2 (ppm)
f1 (ppm)

^{13}C NMR (126 MHz, DMSO-d_6) δ 153.4, 153.3, 136.8, 134.1, 133.4, 128.4, 128.0, 127.9, 118.1, 116.9, 115.2, 113.5, 110.9, 100.7, 78.2, 65.4, 28.2.

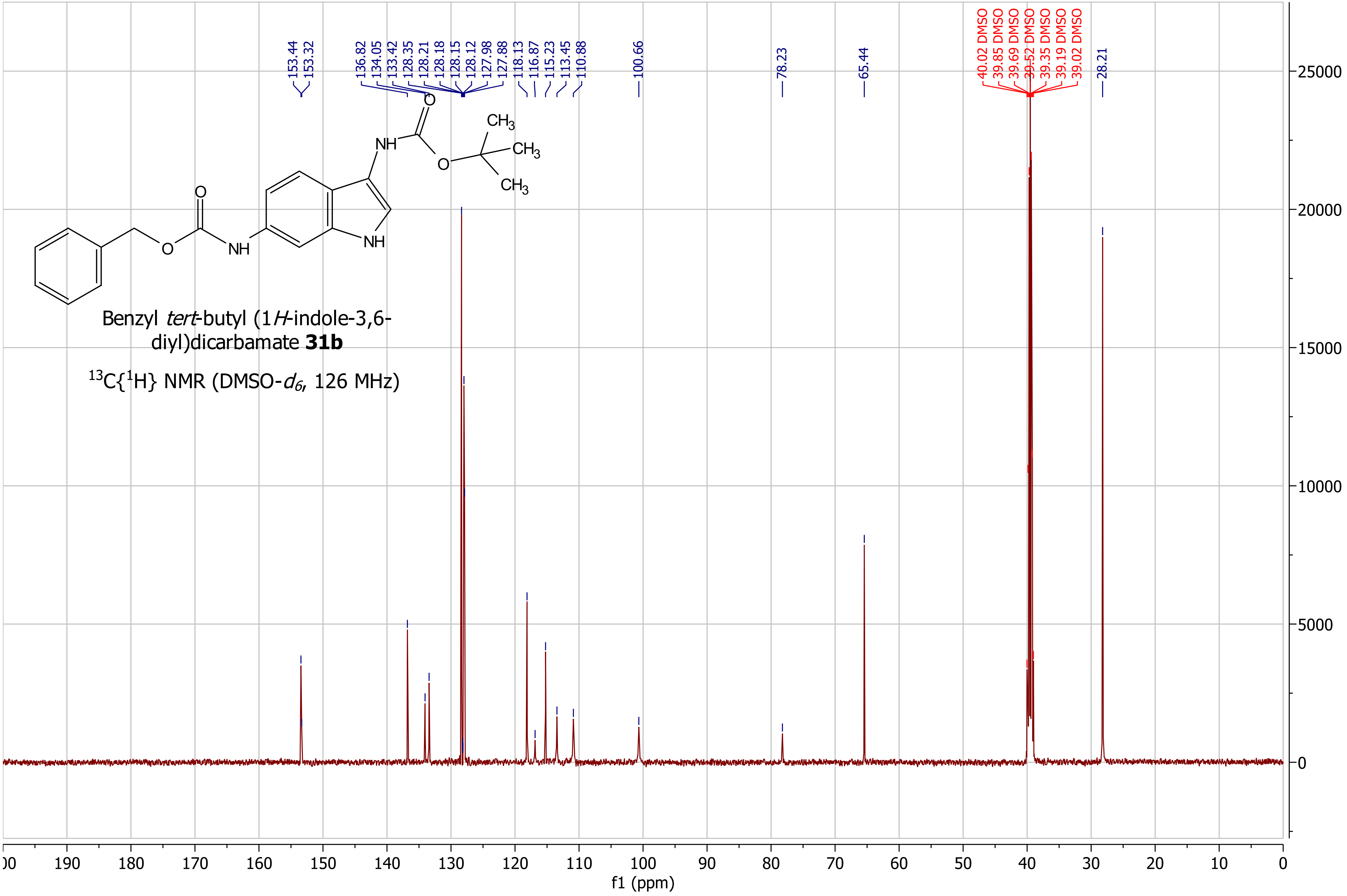

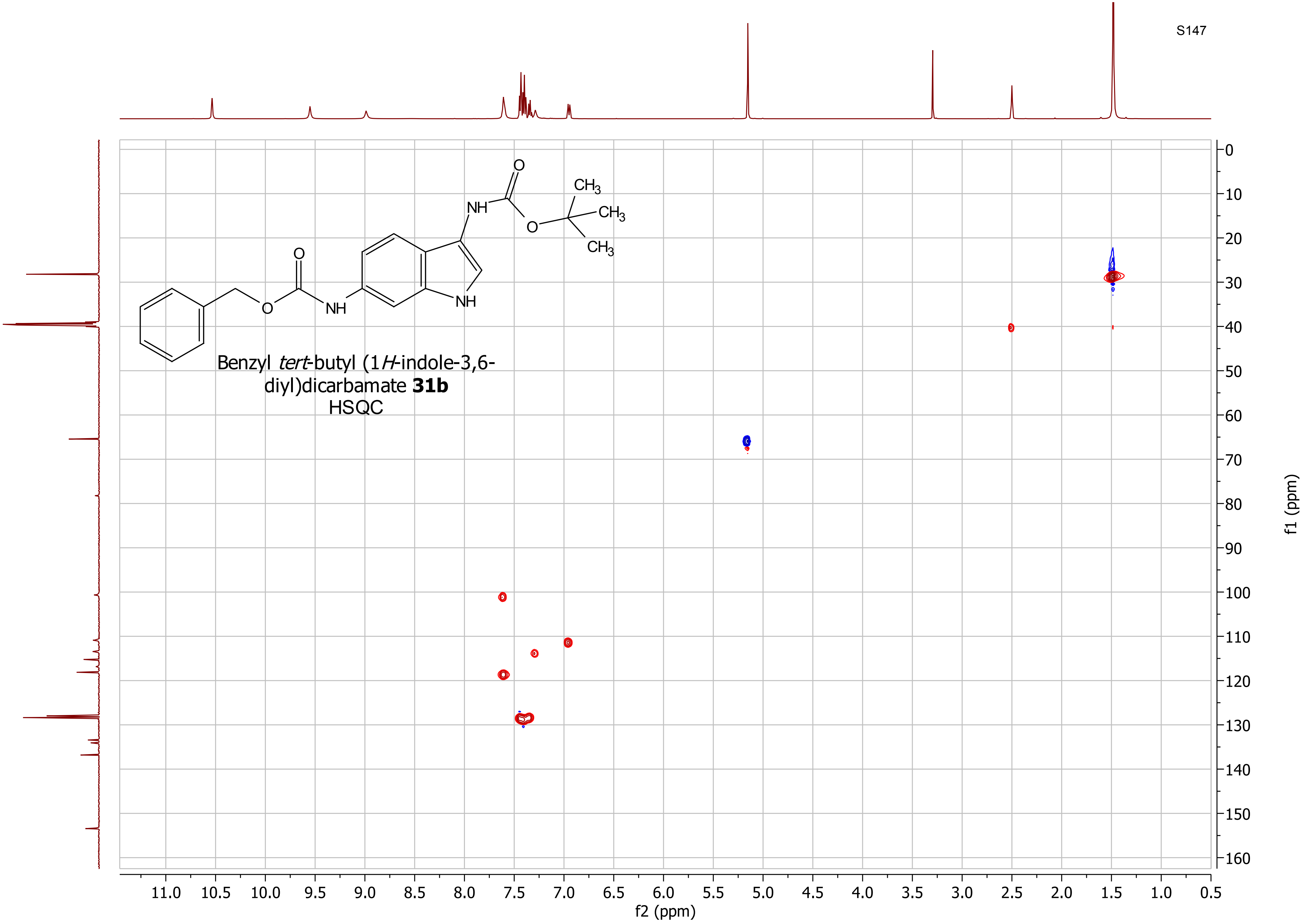
O
NH
CH3
CH3
CH3
O
O
NH
NH
O
Benzyl tert-butyl (1H-indole-3,6-diyl)dicarbamate 31b
HSQC
f1 (ppm)
f2 (ppm)
0
10
20
30
40
50
60
70
80
90
100
110
120
130
140
150
160
11.0
10.5
10.0
9.5
9.0
8.5
8.0
7.5
7.0
6.5
6.0
5.5
5.0
4.5
4.0
3.5
3.0
2.5
2.0
1.5
1.0
0.5

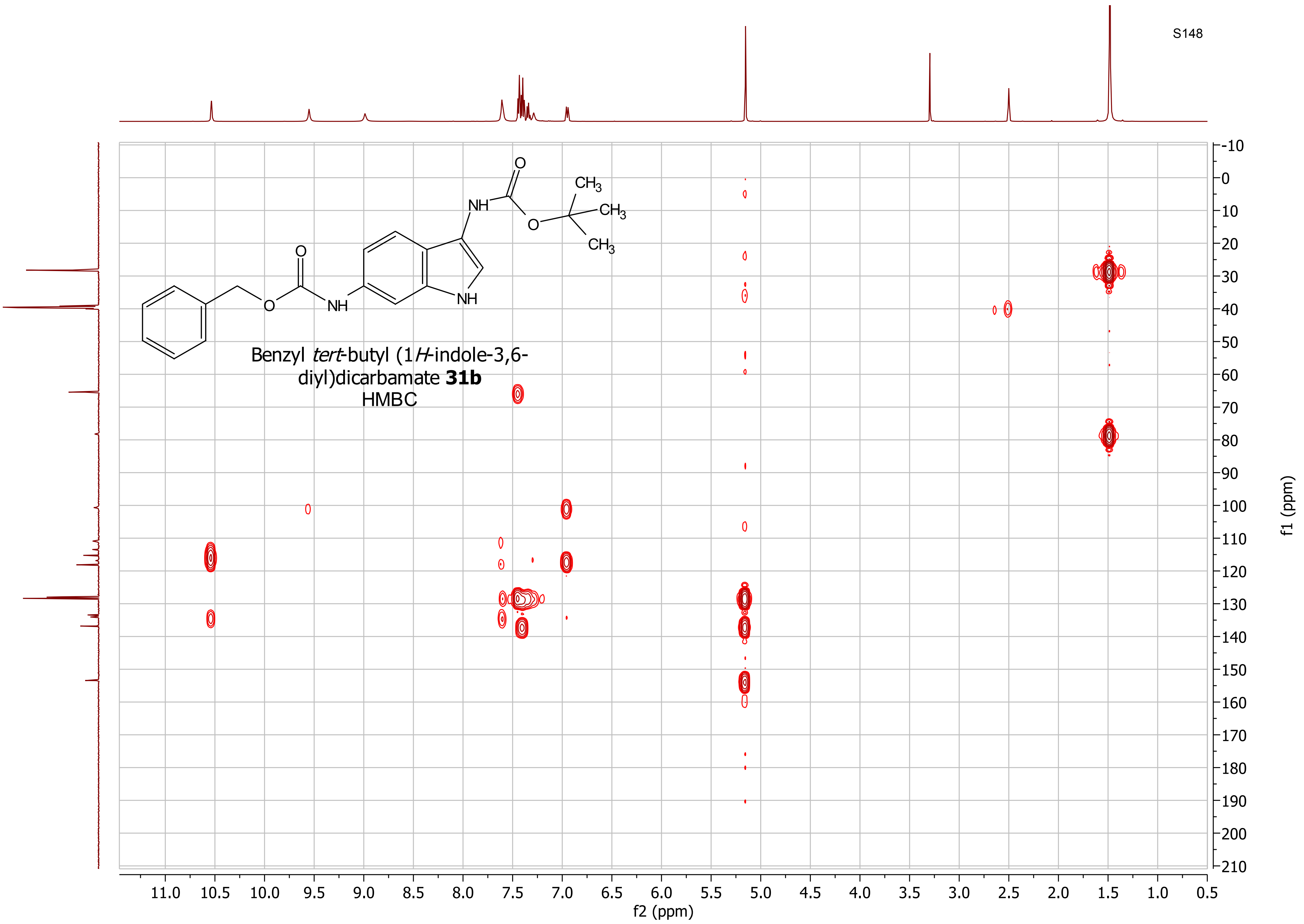
Benzyl *tert*-butyl (1*H*-indole-3,6-diyl)dicarbamate **31b**
HMBC
f2 (ppm)
f1 (ppm)

^{1}H NMR (500 MHz, DMSO-d_6) δ 10.44 (s, 1H), 9.38 (s, 1H), 9.05 (s, 1H), 7.58 – 7.29 (m, 8H), 6.93 (t, J = 7.8 Hz, 1H), 5.21 (s, 2H), 1.50 (s, 9H).

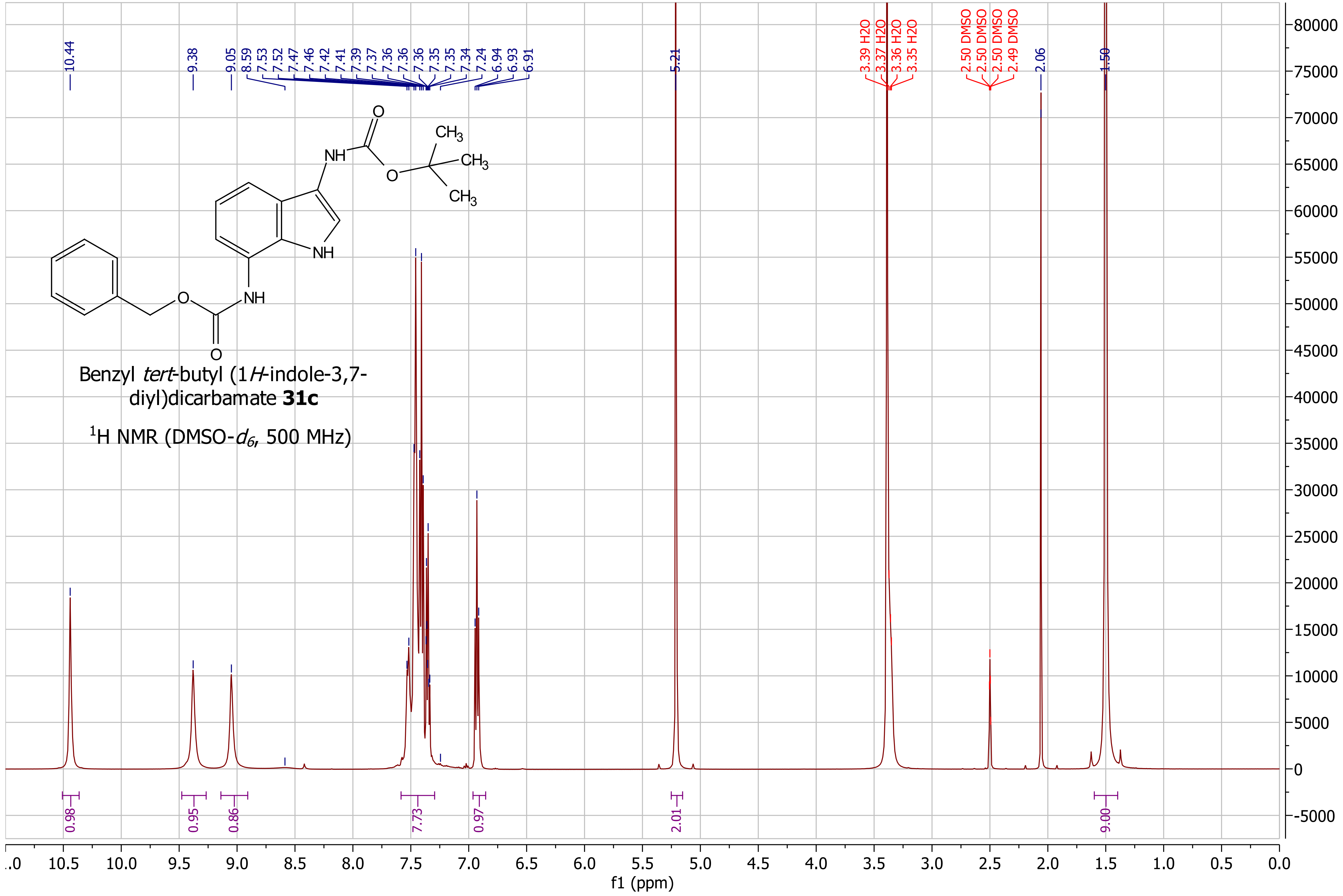

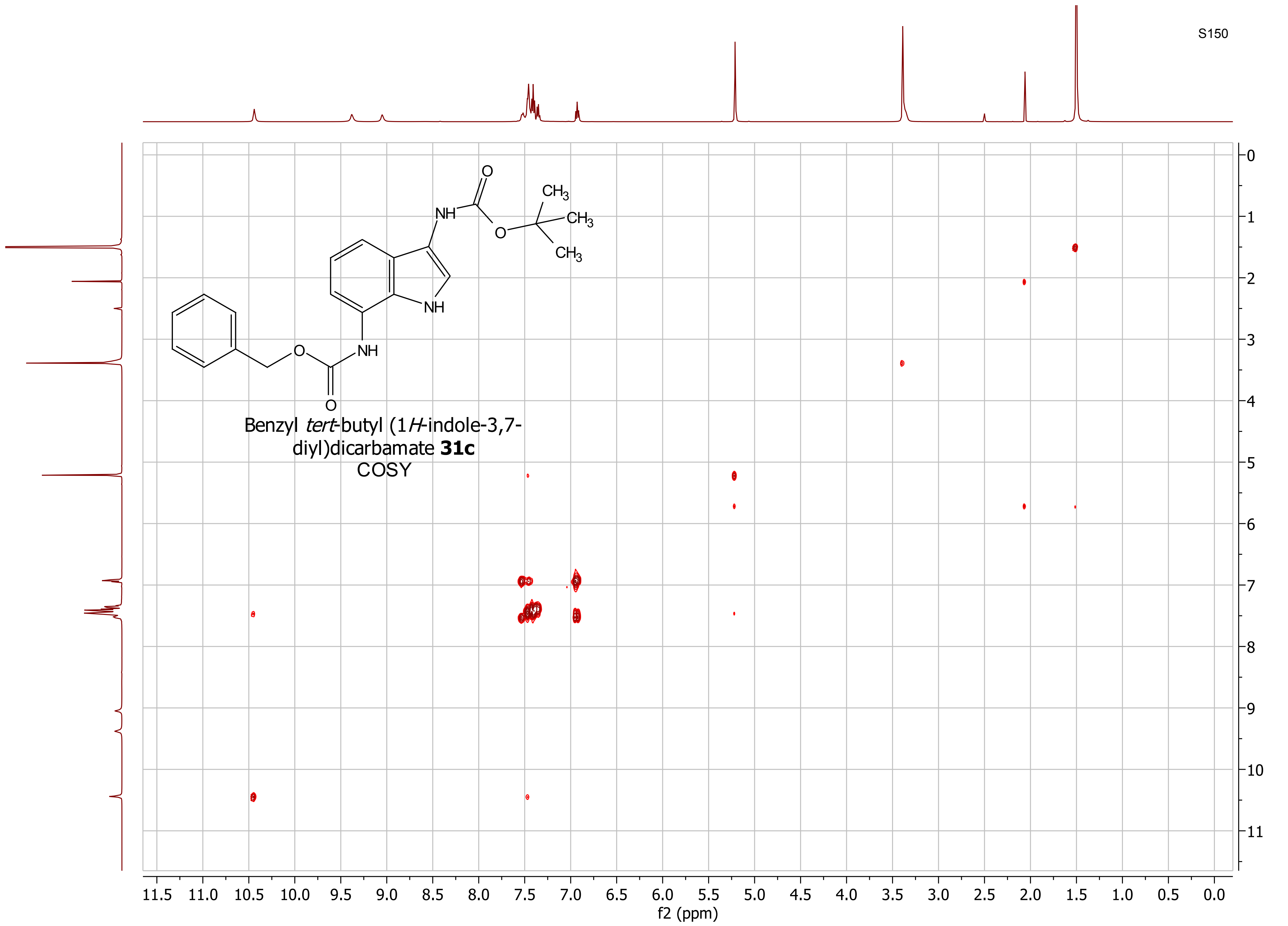

O
NH
CH3
CH3
CH3
O
NH
O
NH
O
Benzyl tert-butyl (1H-indole-3,7-diyl)dicarbamate 31c
COSY
f2 (ppm)
f1 (ppm)

^{13}C NMR (126 MHz, DMSO-d_6) δ 153.8, 153.5, 136.6, 128.5, 128.1, 128.0, 125.9, 123.1, 122.3, 118.2, 115.8, 114.4, 114.0, 112.3, 78.4, 66.0, 28.3.

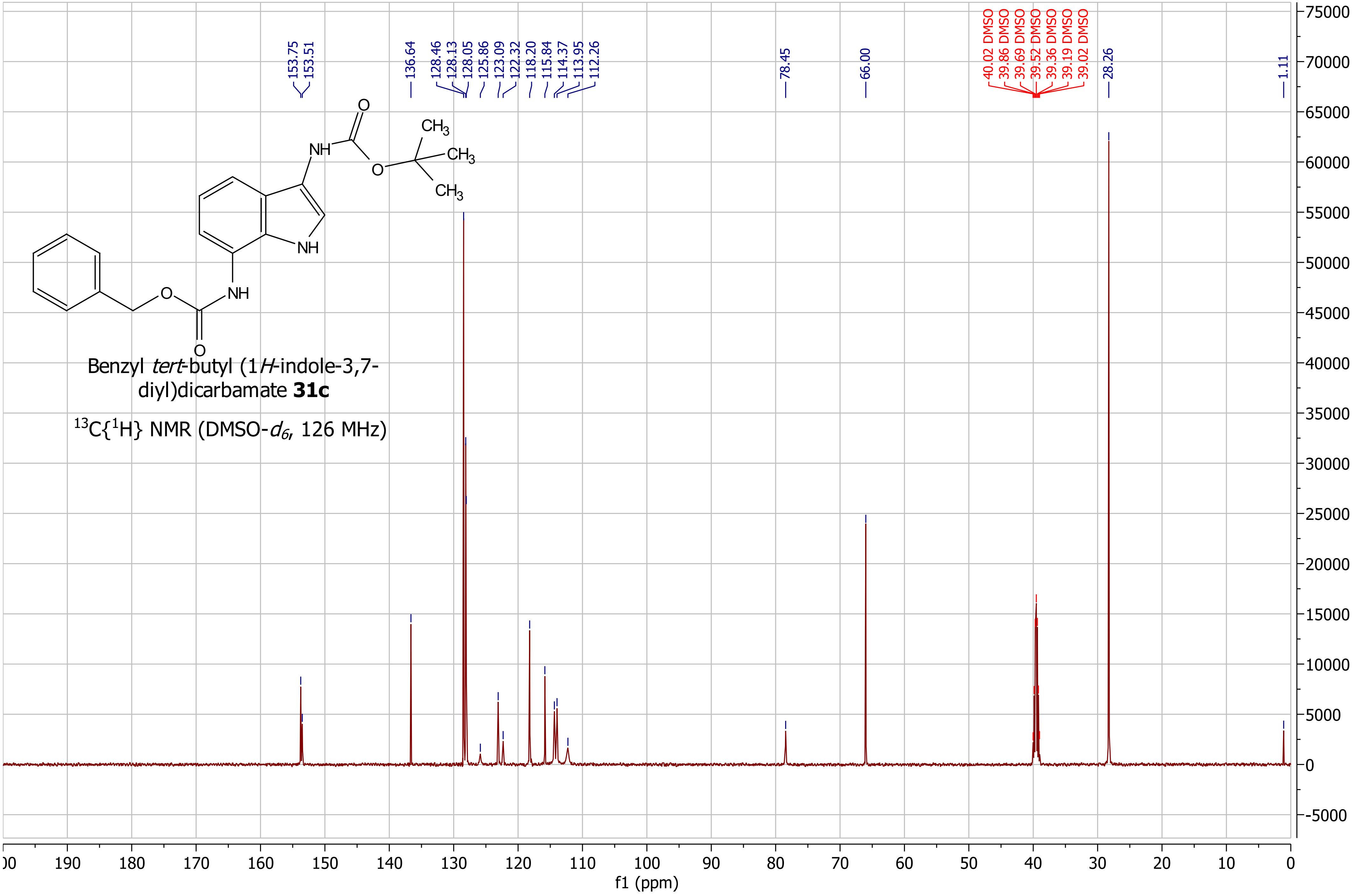

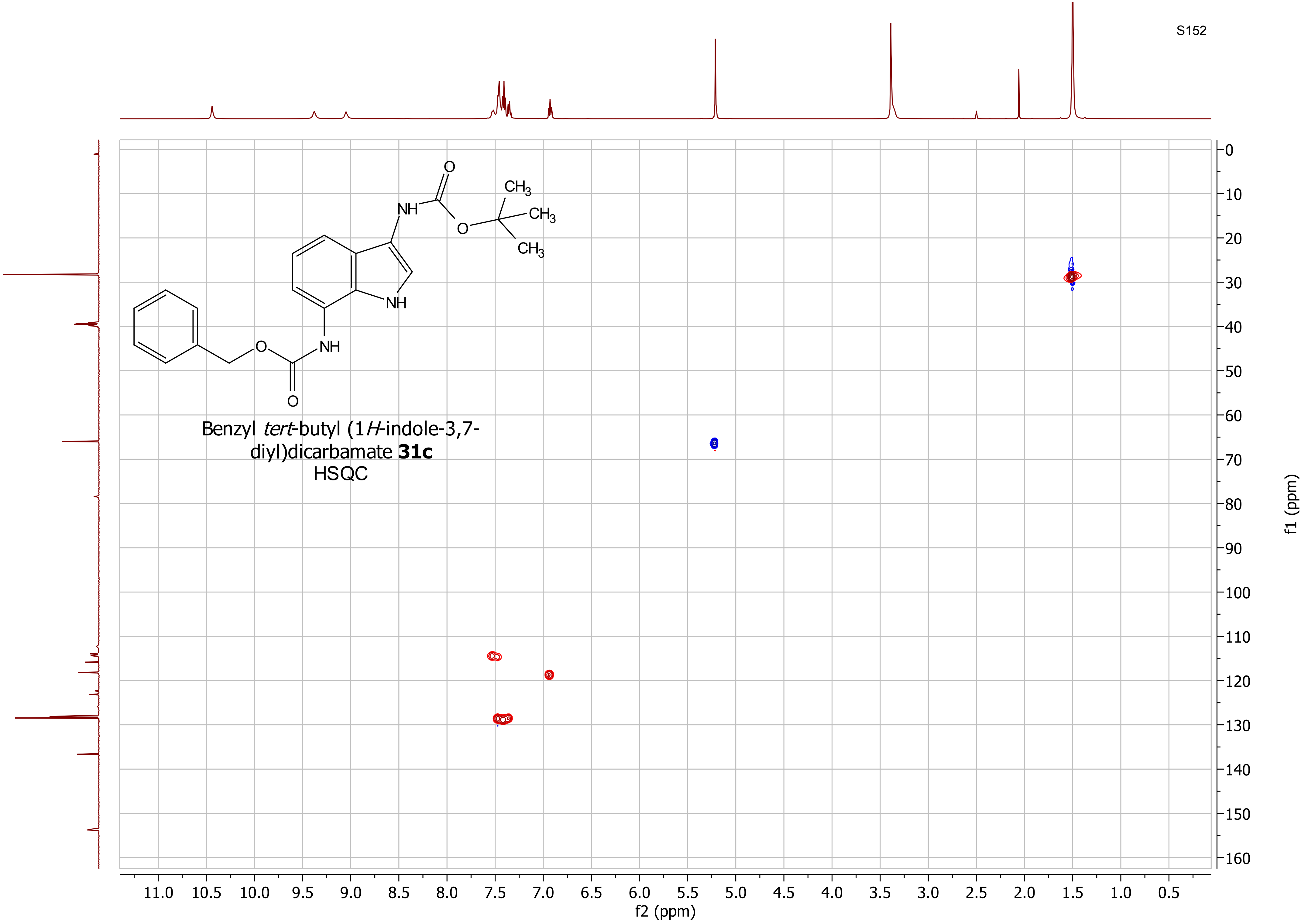
Benzyl *tert*-butyl (1*H*-indole-3,7-diyl)dicarbamate **31c**
HSQC
f2 (ppm)
f1 (ppm)

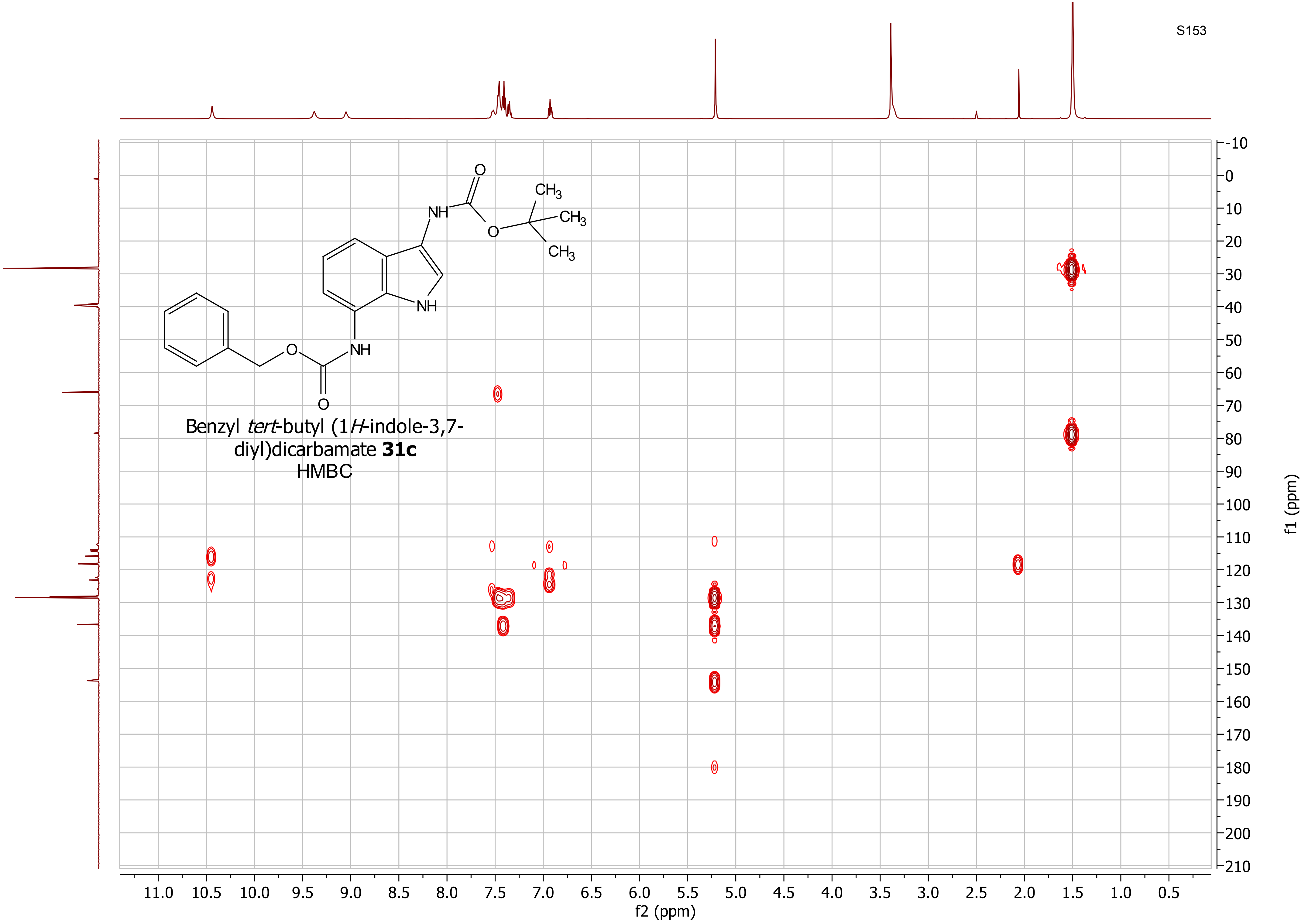
Benzyl *tert*-butyl (1*H*-indole-3,7-diyl)dicarbamate **31c**
HMBC
f2 (ppm)
f1 (ppm)
